# Transfer of structural units through imine exchanges, in solution or without solvent: successive transiminations, stimuli (pH)-modulated covalent switches, and mathematical models

**DOI:** 10.3389/fchem.2025.1241625

**Published:** 2026-04-13

**Authors:** Juan Ramirez, Adrian-Mihail Stadler

**Affiliations:** 1 Faculté de Chimie, Université de Strasbourg, Strasbourg, France; 2 University of Strasbourg Institute for Advanced Study (USIAS), Strasbourg, France; 3 Institut de Science et d’Ingénierie Supramoléculaires (ISIS), Unité Mixte de Recherche (UMR) 7006, Centre National de la Recherche Scientifique (CNRS) - Université de Strasbourg, Strasbourg, France; 4 Institut für Nanotechnologie (INT), Karlsruhe Institut für Technologie (KIT), Karlsruhe, Germany

**Keywords:** imines, transimination, dynamic chemistry, pH, solvent-free reaction, acid-base stimuli, mathematical models, exchange

## Abstract

Transfer of structural units through covalent constitutional (non mechanical) switches based on (bis-)imine/amine exchanges (transiminations), can reversibly be modulated by external stimuli, namely by pH changes through alternate additions of acid and base. This is illustrated in this work, in solution, by means of mono- and dialdehydes derived from pyridine. For example, in the case of a dialdehyde Ald, its reaction with an aromatic amine Am1, produces a first bis-imine Im1. Reaction of bis-imine Im1 with an aliphatic amine Am2 produces the aliphatic bis-imine Im2. Addition of acid regenerates the first bis-imine Im1. Subsequent addition of base produces again the second bis-imine Im2. The aromatic amines Am1 used in this work are aniline-based ones, while the aliphatic ones Am2 are primary alkylamines. Trifluoroacetic acid (TFA) and triethylamine (TEA) are used as pH-triggers (external stimuli) in solution. No metal ions are needed to perform the exchanges. In several cases, an excess of aromatic amine Am1, and further, correlatively, an excess of amine Am2 were used to increase the yields. It is also shown that it is possible to invert the steps and to start with aliphatic amine Am2, to continue with aromatic amine Am1 and acid, then, after appropriate adjustments, with base. (Bis-, tris-)imine/(di)aldehyde exchanges in solution were investigated as well. In addition, in a green chemistry approach, under solvent-free conditions, were performed formation of imines, (bis-)imine/amine exchanges and (bis-)imine/(di)aldehyde exchanges, as well as multistep sequences of successive transiminations. In the solvent-free imine/aldehyde-type sequence of successive exchanges, a monoimine is converted into a tris-imine, the tris-imine, into a bis-imine, which finally generates a new monoimine. In the solvent-free imine/amine-type sequence of transiminations, three monoimines derived from the same aldehyde, are successively generated. The experimental work is complemented with an equilibrium-constants-based, mathematical treatment of exchanges between amines and dialdehyde-based bis-imines at equilibrium, in solution: calculation of the composition at equilibrium, modeling of the pH-adaptive behavior of small dynamic libraries of imines, as well as water-dependent distribution curves. Under particular conditions, a simplified mathematical approach to exchanges (transiminations) can be used, where equilibriums involving di- or monoaldehydes do not appear directly.

## Graphical Abstract

**Figure:**
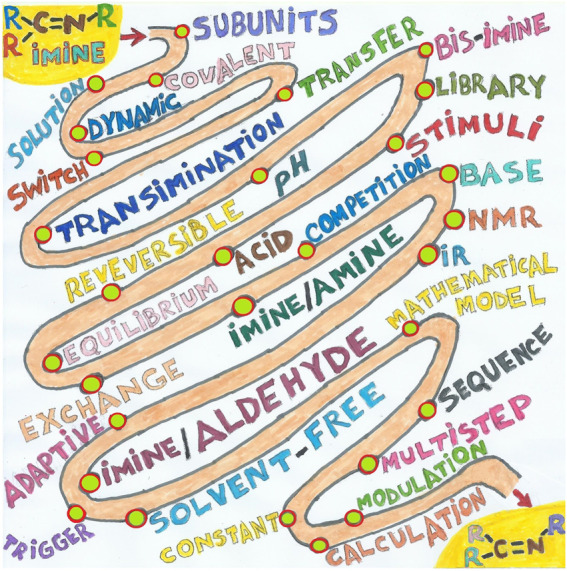

## 1 Introduction

**FIGURE 1:**
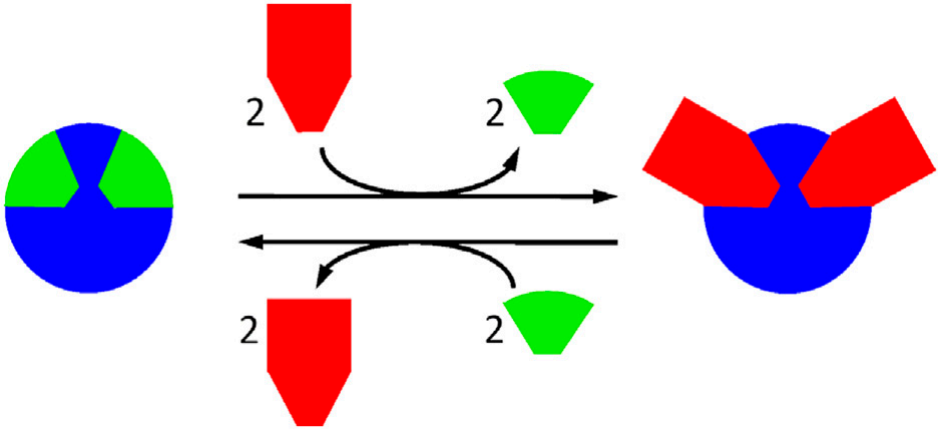
Stylized representation of the displacement of structural units from an architecture of supramolecular and/or covalent nature, possible when key chemical bonds involved herein are, under defined conditions, enough labile. This results in a transfer of structural units.

The structural tuning of chemical species can be performed at covalent and/or supramolecular level, when the concerned bonds are made enough dynamic. It may appear that one or more of the constitutive units of a given molecule can play a structural and/or a functional role in other species and it would be advantageous to transfer it from the first to the second molecule (Figure 1). Such displacement processes can be made possible in the framework of dynamic covalent chemistry (DCC) (Corbett et al., 2006; Lehn, 2007; Rowan et al., 2002), where imines are of much interest (Belowich and Stoddart, 2012; Zentner et al., 2019; You, 2023). In the area of biological catalysts, transiminations take place in reactions catalyzed by pyridoxal phosphate-dependent enzymes (John, 1995; Soniya et al., 2019). Imine exchanges may occur in drug release processes, as well (Dizdarevic et al., 2019).

### 1.1 Di- and oligoaldehydes in dynamic chemistry

**FIGURE 2:**
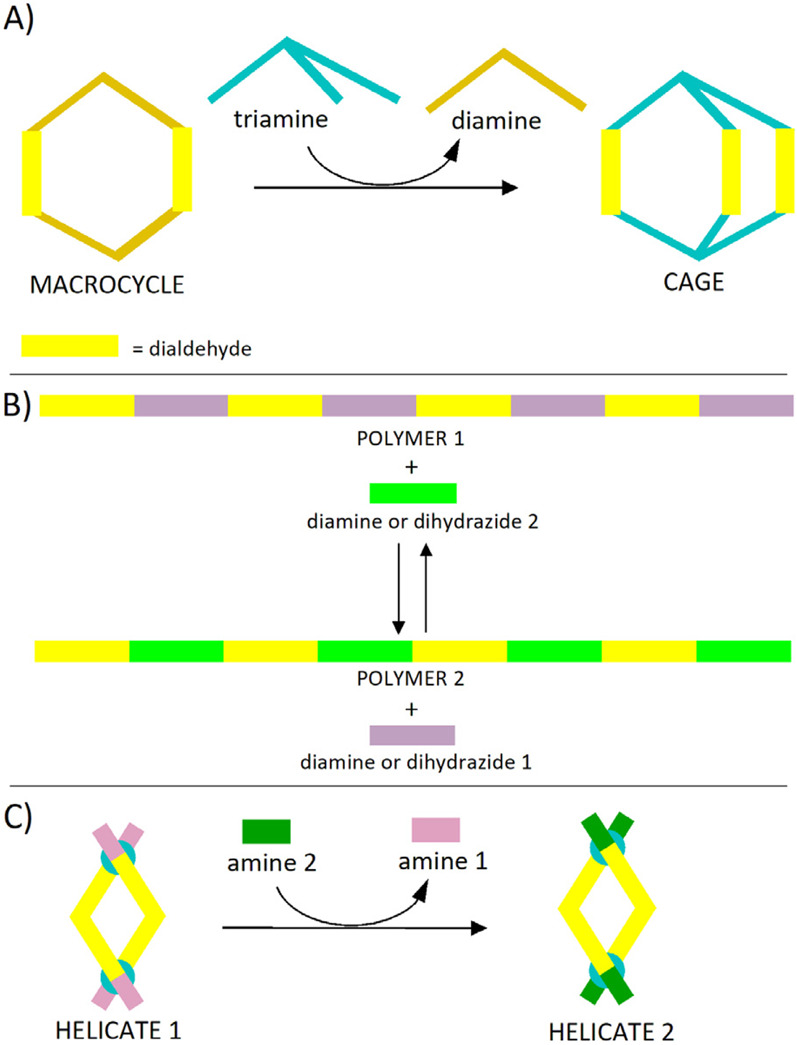
Stylized representation of transiminations where a transfer of dialdehyde-based structural units takes place: **(A)** Conversion of a classical [2 + 2] macrocycle into a cage (Yang and Lehn, 2020); **(B)** Replacement of structural units from polymers (Skene and Lehn, 2004); **(C)** Conversion of a helicate into another one (Schultz and Nitschke, 2006).

Molecules bearing two or more functional groups, like di- and oligoaldehydes and di- and oligoamines, are good candidates for generation of imine-based complex structures. Constitutional exchanges based on imines derived from dialdehydes may be needed and implemented in cases such as the passage from a macrocycle to a cage-like structure (Figure 2A; Yang and Lehn, 2020), the replacement of a co-monomer in a given polymer (Figure 2B) (Skene and Lehn, 2004) or the transfer of a monoamine between helicates based on bis-imine ligands (Figure 2C) (Schultz and Nitschke, 2006). In the area of dynamic imine chemistry, dialdehyde-based building blocks can act as structural units of dynamic polyimine macrobicyclic cryptands (Kolodziejski et al., 2019), of imine-linked covalent organic frameworks (Wang et al., 2023), of cages (Schick et al., 2020), of recyclable polymers (Deng et al., 2022) or of polymeric architectures from imine-based covalent adaptable networks (Schoustra et al., 2021). They can also be exchanged between imine-based open circular helicates or between closed-loop pentafoil knots (Ayme et al., 2019). Moreover, recently, in the domain of oligoaldehydes, an imine cage derived from a trialdehyde was transformed, through transimination, into a covalent organic framework film (Giri et al., 2023).

### 1.2 General motivation for alternate transiminations

The study of such dynamic processes can be motivated by, for example.

- The wish to explore new dynamic covalent assemblies and/or to solve synthetic problems with help of the DCC;
- The wish to design and synthesize versatile chemical systems with alternate properties and functions, associated to structures which can alternately be generated in the same pot;
- The saving of starting materials and reagents in a green chemistry approach or due to their limited availability (difficult to synthesize or to recover, expensive). Indeed, in the case of stimuli-triggered switches, the reagents and products of transimination can be reused in a certain number of cycles, without isolation and loss;
- The saving of solvents, the sequence of reactions being done in the same pot or, when possible, under solvent-free conditions;
- The development of methods (e.g. separation, purification) or (nano)materials based on exchanges between amines and imines (Diez-Castellnou et al., 2021) derived from immobilized (Krishnamoorthy and Grubbs, 2020) aldehydes.

### 1.3 Exchanges involving dialdehyde-based bis-imines

In the typical and illustrative example of dialdehyde-based bis-imines (which are investigated in the main part of this work), for transiminations through reaction with primary monoamines, a first (or initial) bis-imine should form from the corresponding dialdehyde and a first amine, according to the reaction

Dialdehyde+2 First amine ⇌ First bis−imine+2 Water

This first bis-imine reacts with a second amine that replaces the first amine. In this way is produced, through transimination (for transiminations, see: Ciaccia et al., 2013; Ciaccia et al., 2014; Madec et al., 1997; Vitaku and Dichtel, 2017; Wang et al., 2019; Ye et al., 2012), the second bis-imine:

First bis−imine+2 Second amine ⇌ Second bis−imine+2 First amine

An important question related to these processes, is if there are some general conditions that could ensure a good yield of transimination reactions. Another point is whether there is a quite fast and simple means that can reverse this situation at equilibrium (thus leading to the formation of the first imine) and that could be generalized and applied without isolation of the products. And once this equilibrium is reversed, one may wish to find a quite conveniently to implement means to go back again and to regenerate the second imine.

### 1.4 pH-modulated, imine-involving exchanges

It was shown that selection in libraries of imines can be modulated through pH changes (Giuseppone and Lehn, 2006). An aliphatic diamine incorporated into the bis-imine ligand of a Cu(I) mononuclear complex, was displaced on treatment of the complex with an aromatic diamine dihydrochloride (Schultz and Nitschke, 2005). Sulfanilic acid produced, by protonation and transimination, displacedment of aliphatic amines from bis-imines acting as ligands in Cu(I) dinuclear helicates (Nitschke et al., 2004). We have previously shown (Stadler, 2013) that the selectivity of Schiff base formation in mixtures of 1 equiv. of aromatic amine and 1 equiv. of aliphatic amine upon reaction with 1 equiv. of aldehyde, can significantly be modulated between the absence and the presence of 1 equiv. of acid. Indeed, in the absence of acid, mainly forms the aliphatic imine, while in its presence, the main product is the aromatic one. Similarly, gold nanoparticles bearing immobilized aldehydes react, in the presence of equal amounts of aliphatic and aromatic amine, preferentially with the aliphatic amine, while in the presence of acid, the preference is reversed; it is reestablished on addition of base (Diez-Castellnou et al., 2021).

Such processes of reorganization of the molecular constitution (here, at the covalent level) upon alternate pH changes can also be seen as an adaptive response of dynamic libraries of aldehydes, amines and imines to external stimuli (here, the pH).

### 1.5 Preliminary overview

With the above considerations in mind, we report herein (Section 2) on the formation of (di)aldehyde-based aromatic and aliphatic (bis-)imines, and on (bis-)imine/amine exchanges in solution, as well as on the synthesis of a mono- and a dialdehyde. The key point consists of pH-modulated transiminations where an aromatic amine works as first (initial) amine, and an aliphatic one, as second amine. The difference of basicity and nucleophilicity between the two amines should make possible a better selectivity and its modulation through the pH. The aim of the first part is rather to illustrate this kind of covalent switch (Zou et al., 2019) based on transiminations, than to produce a study with a considerable number of examples.

We further wished to extend the investigation to other types of exchanges (namely, to imine/aldehyde exchanges) and to other conditions (namely, without solvent). A number of (tris-, bis-)imine/(di)aldehyde exchanges (Section 3) are also presented here, although a way to trigger them through external stimuli (e.g. pH changes) is not yet available.

Imine formation and transiminations, including sequential multistep processes, were tried under free-solvent conditions, as well (Section 4).

The first four parts of this work are complemented by a fourth one. The last part (Section 5) deals with calculations based on equilibrium constants, which include the determination of the composition at equilibrium starting from initial concentrations of amines and aldehydes, equilibrium-constants-based modeling of the pH-adaptive behavior of small dynamic libraries of imines and calculation of water-dependent distribution curves.

This work is mainly focused on dialdehydes. Monoaldehydes can serve as models or as competing aldehydes. A trialdehyde was used in a sequence of successive, multistep exchanges, and the corresponding tris-imine was used for an exchange in solution.

With one exception (ketimine **bzphim**; vide infra), all Schiff bases used in this work are imines derived from aldehydes, namely (mono-, bis- or tris-)aldimines. In the framework of this report, where all aldehydes are aromatic ones, “aromatic” imine means an imine derived from an aromatic amine, and an “aliphatic” imine is one derived from an aliphatic amine.

### 1.6 Experimental considerations

As preliminary experimental observations, one may notice that reactions in solution (except those where the imine products are isolated) are done in NMR tubes (0.5–1 mL of solution), on milligram scale. The starting mono- or bis-imine may directly be prepared in the tube and used without purification, or may be isolated by precipitation, or prepared in another convenient way (like the solvent-free methods, vide infra). In most cases of dialdehyde-based bis-imines prepared in the tube, we used an excess of monofunctional reagent (monoamine) to increase the yield of starting aromatic bis-imine (vide infra).

For the switches in solution, the reagents are usually added in small volumes of solvent, with appropriate microsyringes. In practice, depending on factors like the amount of water from the solvent, the amount of water from the reagents and the uncertainties associated to the preparation of the solutions, it might be necessary for the theoretically calculated volumes of reagent solutions, to be slightly adjusted. In order to keep the volume of the reaction mixture as constant as possible and to limit the external (undesired) inputs of water, one may, amongst other possibilities: i) use concentrated solutions of reagents, which lead to very small volumes of solution to add, so that the overall volume of the reaction mixture can be seen as constant; ii) add the reagent in a small volume of solvent, then, under Ar or N_2_ flow, without heating, evaporate the corresponding volume of solvent from the reaction mixture.

Chloroform-d3 (main NMR solvent) may contain traces of acid (Teipel et al., 2022). It is usually filtered through basic alumina; the kinetics may be affected by possible traces of acid, which may act as a catalyst.

The reaction mixtures in solution are generally equilibrated at room temperature. The equilibration can be accelerated by heating.

The working concentrations before reaction are usually between 3 × 10^-3^M and 2 × 10^-2^M. The yields and conversions were determined by ^1^H NMR (integration uncertainties being, on average, of about ±5%).

The equilibrium constants associated to imine formation or exchange were roughly estimated through NMR spectroscopy. For reactions involving water, the concentration of water in the reaction mixture at equilibrium was included in the calculation of the constants. In our experiments, the concentration of water (before and after equilibration) is usually below the solubility (of about 7.2 × 10^-2^M at 25°C) of water in chloroform (Horwath et al., 1995).

Chemicals from commercial sources were used without purification.

## 2 Exchanges between (bis-)imines and amines in solution. pH-modulated covalent switches

### 2.1 General principles. Choice of aldehydes and amines. Main equilibriums

Such (bis-)imine/amine transiminations are covalent constitutional interconversions between two architectures, which occur through breaking and formation of C=N dynamic covalent bonds (Chakma and Konkolewicz, 2019; Wanasinghe et al., 2022), generally under equilibrium conditions. As covalent (non mechanical) constitutional switches, they should operate in a way that can be made reversible, should not be too slow, should be triggered by stimuli and it should be possible to modulate and implement them in a relatively accessible way.

#### 2.1.1 A short classification of (bis-)imine/(di)amine exchanges

This part of our work is limited to exchanges which are based on mono- or difunctional compounds and does not deal with macrocyclic or polymeric imines. An attempt of systematic presentation of the exchanges between (bis-)imines and (di)amines can be done on the basis of a short classification that includes the following types of reactions (Am = amine, Im = imine, Diam = diamine, Diim = bis-imine).

- imine I with amine II (formation of amine I and of target imine II)
$${\text{Im}}_{I}+{\text{Am}}_{\text{II }}⇌{\text{ Im}}_{\text{II}}+{\text{Am}}_{I}$$
- dialdehyde-based bis-imine I with amine II (formation of amine I and of target bis-imine II)
$${\text{Diim}}_{I}+2\;{\text{Am}}_{\text{II }}⇌{\text{ Diim}}_{\text{II}}+2\;{\text{Am}}_{I}$$
- diamine-based bis-imine I with amine II (formation of diamine I and of target imine II)
$${\text{Diim}}_{I}+2\;{\text{Am}}_{\text{II }}⇌\;2\;{\text{Im}}_{\text{II}}+{\text{Diam}}_{I}$$
- diamine-based bis-imine I with diamine II (formation of diamine I and of target bis-imine II)
$${\text{Diim}}_{I}+{\text{Diam}}_{\text{II }}\;⇌{\text{ Diim}}_{\text{II}}+{\text{Diam}}_{I}$$
- imine I with diamine II (formation of amine I and of target diamine-based bis-imine II)
$$2\;{\text{Im}}_{I}+{\text{Diam}}_{\text{II }}⇌{\text{ Diim}}_{\text{II}}+2\;{\text{Am}}_{I}$$
- macrocyclic bis-imine I (from diamine I and dialdehyde) with diamine II (formation of diamine I and of target macrocyclic bis-imine II or of oligomeric or polymeric structures).

In terms of number of imine groups per imine-containing-molecule, between the starting (bis-)imine and the target one, there are cases where this number remains constant (e.g. amine/dialdehyde-based-bis-imine exchanges, namely from 2 (bis-imine) to 2 (bis-imine)), and cases where it changes (e.g. amine/diamine-based-bis-imine exchanges, namely from 2 (bis-imine) to 1 (imine)). This classification is of interest also for the choice of the mathematical model which is to be applied to the system.

One deals in this section with exchanges, in solution, between monoamines and bis-imines derived from dialdehydes (point ii of the above classification), and between monoimines and monoamines (point i of the classification). In the examples presented here, I from the previous classification corresponds to the aromatic nature of the amine, and II, to its aliphatic nature.

#### 2.1.2 Choice of aldehydes

**FIGURE 3:**
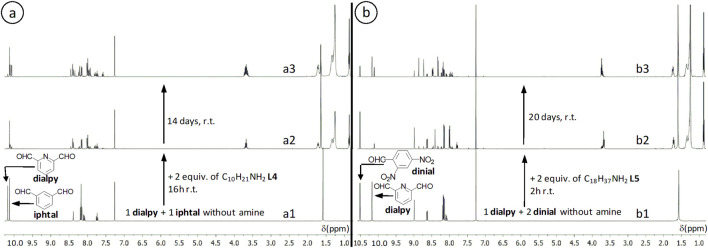
^1^H NMR spectra (CDCl_3_, 400 MHz) for **(a)** the reaction of a mixture consisting of 1 equiv. of 2,6-pyridinedicarboxaldehyde **dialpy** and 1 equiv. of isophthalaldehyde **iphtal** (a1), with 2 equiv. of decylamine **L4** (a2, a3) and for **(b)** the reaction of a mixture of 1 equiv. of 2,6-pyridinedicarboxaldehyde and 2 equiv. of 2,4-dinitrobenzaldehyde **dinial** (b1), with 2 equiv. of octadecylamine **L5** (b2, b3).

Reaction of an equimolar mixture of 1 equiv. of 2,6-pyridinedicarboxaldehyde **dialpy** and 1 equiv. of isophtalaldehyde **iphtal** with 2 equiv. of decylamine **L4** produces a reaction mixture where 2,6-pyridinedicarboxaldehyde is consumed, under kinetic conditions, faster than isophtalaldehyde (Figure 3a). A similar behavior is observed when a mixture of 1 equiv. of 2,6-pyridinedicarboxaldehyde **dialpy** and 2 equiv. of 2,4-dinitrobenzaldehyde **dinial** reacts with 2 equiv. of octadecylamine **L5** (Figure 3b). The bis-imine from pyridine-derived dialdehyde is the main kinetic product (yield >67%, depending on the reaction time). This seems however to be a rather kinetic situation, that may be different from that at equilibrium.

Reaction of mixtures consisting of 1 equiv. of a benzene-derived aldehyde and 1 equiv. of a homologous pyridine-derived one (3-bromobenzaldehyde and 6-bromo-2-pyridinecarboxaldehyde; salicylaldehyde and 3-hydroxy-2-pyridinecarboxaldehyde; 2-naphtaldehyde and 2-quinolinecarboxaldehyde) with p-toluidine, suggests that, for the same kinds of substituents, similarly disposed, the pyridine-derived aldehyde reacts faster than the homologous benzene-derived one (see Supplementary Material = SM), file synt-char, p. 129).

On the basis of these results and with the aim of reaching reasonable reaction rates, we decided to use, for the syntheses of (bis-) imines involved in the exchanges performed in this part of the work, (di)aldehydes derived from pyridine (or quinoline), rather than (di)aldehydes derived from benzene.

#### 2.1.3 Choice of aromatic amines

In the framework of a qualitative screening, the choice of aromatic amines was done in several steps. We worked mainly with benzene-based amines and we tried to select, amongst a limited set of compounds, most of which are commercially available, those which would react faster.

Aldehydes may generate, as autoxidation products with oxygen (Liao et al., 2022), acids. Chloroform-d_3_, which is the main solvent for this work (although several reactions were done in acetonitrile-d_3_), may contain traces of hydrochloric acid (Teipel et al., 2022). In order to diminish the influence of such potential acid sources on the comparison of reaction rates, we decided to put two or three amines in competition for a same aldehyde, in order to purely qualitatively compare their reactivities.

**FIGURE 4:**
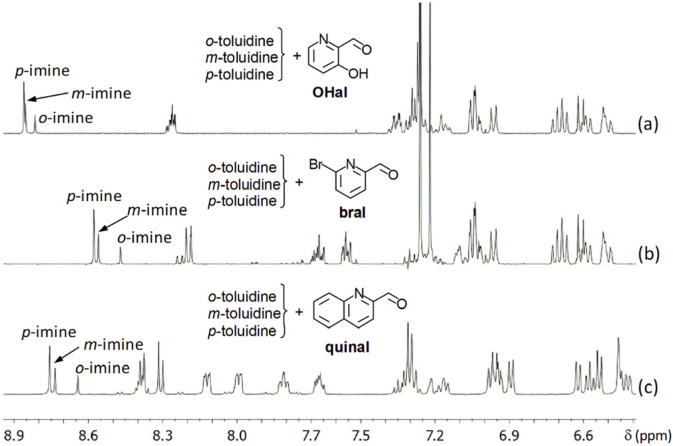
Part of ^1^H NMR spectra (400 MHz) of reaction mixtures of 1 equiv. of o-, 1 equiv. of m- and 1 equiv. of p-toluidine with 1 equiv. of 3-hydroxy-2-pyridinecarboxaldehyde **OHal** (a; CD_3_CN), 1 equiv. of 6-bromo-2-pyridinecarboxaldehyde **bral** (b; CDCl_3_) or 1 equiv. of 2-quinolinecarboxaldehyde **quinal** (c; CDCl_3_).

**FIGURE 5:**
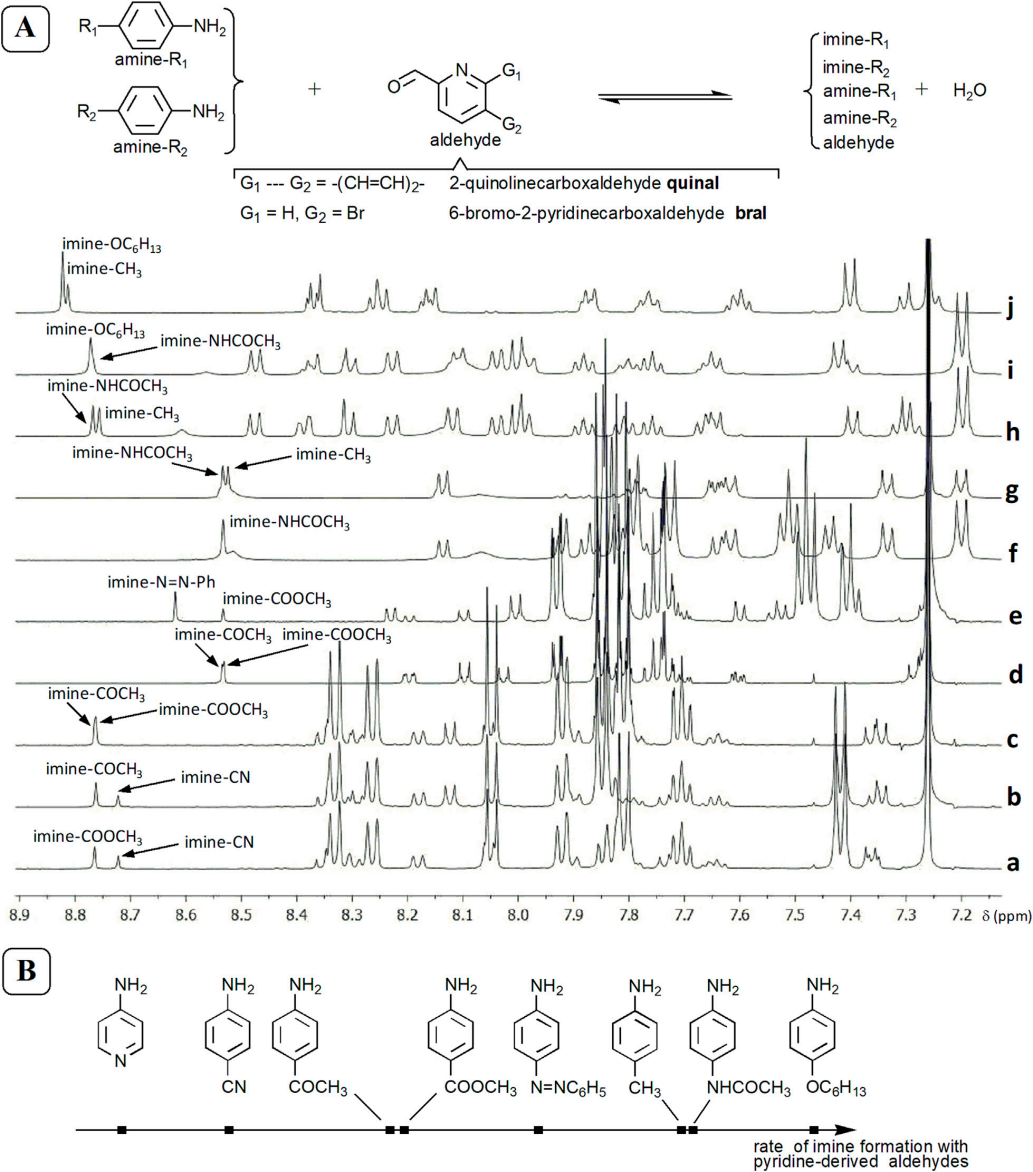
**(A)** ^1^H NMR spectra (500 MHz) of reaction mixtures of 1 equiv. of p-R_1_-aniline and 1 equiv. of p-R_2_-aniline with 1 equiv. of aldehyde: (a) R_1_ = COOCH_3_, R_2_ = CN, **quinal**, CDCl_3_; (b) R_1_ = COCH_3_, R_2_ = CN, **quinal**, CDCl_3_; (c) R_1_ = COOCH_3_, R_2_ = COCH_3_, **quinal**, CDCl_3_; (d) R_1_ = COOCH_3_, R_2_ = COCH_3_, **bral**, CDCl_3_; (e) R_1_ = N=N-Ph, R_2_ = COOCH_3_, **bral**, CDCl_3_; (f) R_1_ = NHCOCH_3_, R_2_ = N=N-Ph, **bral**, CD_3_CN; (g) R_1_ = NHCOCH_3_, R_2_ = CH_3_, **bral**, CD_3_CN; (h) R_1_ = NHCOCH_3_, R_2_ = CH_3_, **quinal**, CD_3_CN; (i) R_1_ = NHCOCH_3_, R_2_ = OC_6_H_13_, **quinal**, CD_3_CN; (j) R_1_ = CH_3_, R_2_ = OC_6_H_13_, **quinal**, CDCl_3_. Working concentrations between 6 mM and 7 mM. The mixtures are not equilibrated, because one focuses on relative reaction rates. **(B)** Rough, qualitative order of aromatic amines (several p-substituted-anilines and 4-aminopyridine) based on qualitative comparisons of the rates of their reactions with aldehydes, in chloroform.

- Choice of positional isomers. Reaction of equimolar mixtures consisting of one equivalent of o-, m- and p-toluidine with one equivalent of 3-hydroxypyridine-2-carboxaldehyde **OHal**, 2-quinolinecarboxaldehyde **quinal** or 6-bromo-2-pyridinecarboxaldehyde **bral** (Figure 4) shows that p-toluidine is the most reactive. Reaction of a mixture of 1 equiv. of o- and 1 equiv. of p-anisidine, with 1 equiv. of 6-bromo-2-pyridinecarboxaldehyde, shows that p-anisidine reacts faster than o-anisidine.
- Choice of primary aromatic amines. Further, we comparatively tested seven p-substituted-anilines (4-acetylaniline, 4-aminoacetanilide, 4-aminoazobenzene, 4-aminobenzonitrile, methyl-4-aminobenzoate, 4-(hexyloxy)aniline **R4** and p-toluidine **R3**), by making subsets of two amines. To this purpose, equimolar mixtures of 1 equiv. of a first p-substituted-aniline and 1 equiv. of a second one were reacted with 1 equiv. of aldehyde (2-quinolinecarboxaldehyde **quinal** or 6-bromo-2-pyridinecarboxaldehyde **bral**; for a selection of spectra, see Figure 5A). These qualitative comparisons are mainly based on the relative intensity of peaks corresponding to the protons from CH=N imine groups. They lead to a qualitative and relative estimation of the order of p-substituted-anilines based of the rate of their reaction with pyridine- and quinoline-derived aldehydes (Figure 5B; preliminary results suggest that the same order would hold for the formation constants of the corresponding imines). 4-Aminopyridine was also tested, in chloroform-d_3_. These results (Figure 5B) together with those from reactions of 2,6-pyridinedicarboxaldehyde with p-substituted-anilines possessing OC_6_H_13_, CH_3_, CN, COCH_3_ and COOCH_3_ at the para position (see SM, file synt-char, pp. 111–115) led us to consider p-toluidine and p-(hexyloxy)aniline as good aromatic amine candidates, especially when starting imines are intended to be prepared in the NMR tube and to be further used without isolation.
  The nucleophilicity of these amines can, in most cases, be correlated with the inductive (I) and mesomeric (M) effects of substituents: CN, COCH_3_, COOCH_3_ (−I and −M), CH_3_ (+I) and NHCOCH_3_, OC_6_H_13_ (−I < +M).

Considering these results, we focused our work mainly on p-anilines. Only a few experiments with m-substituted anilines were performed.

#### 2.1.4 Motivation for dialdehydes **A** and **dialpy**

**FIGURE 6:**
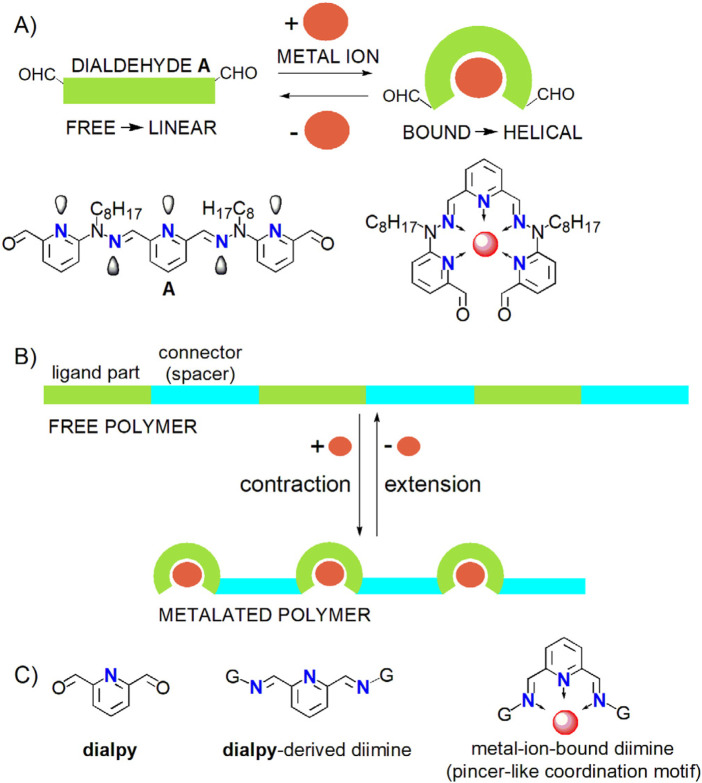
**(A)** Dialdehyde **A** (Figure 7) possesses a metal-ion-dependent mechanical motional function: as a free ligand, it adopts a linear conformation, which becomes helical on reaction with metal ions (red circles), thus resulting in a contraction. **(B)** The polymers generated with **A** as a comonomer should undergo reversible contraction on reaction with metal ions. Stylized representations. **(C)** Structural formulae of dialdehyde **dialpy**, of its bis-imine (general case) and of the corresponding pincer-like coordination motif.

**FIGURE 7:**
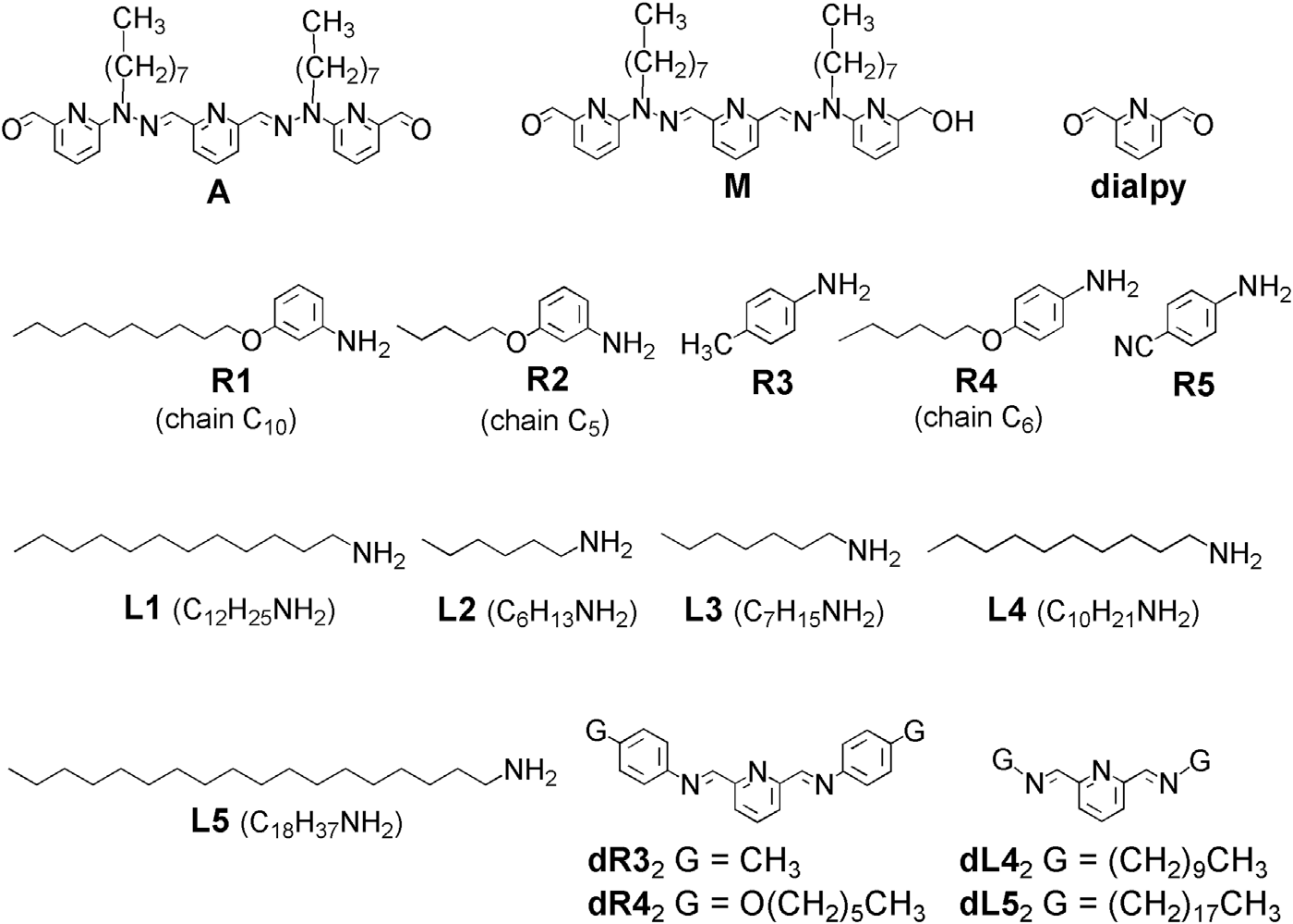
Structural formulae of monoaldehyde **M**, dialdehydes **A** and **dialpy**, aromatic amines **R1-R5**, aliphatic amines **L1-L5** and bis-imines **dR3**_2_, **dR4**_2_, **dL4**_2_ and **dL5**_2_.

- In the dialdehyde **A** (Figures 6A, 7), the sequence generated by pyridine rings together with the hydrazone groups adopts a linear conformation (Figures 6, 7). Such sequences can contract and become helical on reaction with metal ions (Figures 6, 7) (Stadler and Lehn, 2014), like Pb^2+^. The contraction is reversible upon removal of the metal ions. This could provide our system with a potential mechanical motional function (not investigated here), which will be the basis of a future work dealing with contractile polymeric architectures (Figure 6B; see (Dattler et al., 2020)).
- Dialdehyde **A** incorporates, as its central moiety, the **dialpy**-derived sequence 2,6-bis(imino)pyridine (Figure 6C), that may act as a tridentate ligand and produce pincer like-coordination motifs or complexes (Gibson et al., 2007), a fact that motivates our interest in exploring such **dialpy**-derived bis-imines.

The study of the dicarbonyl species **A** is advantageously preceded by that of the model monoaldehyde **M**.

#### 2.1.5 Overview of equilibriums

Control of imine exchanges through protonation/deprotonation of amines is a convenient one, given that acids and bases are easily available. The simplified sequence of exchanges for a molecule Ald bearing n aldehyde groups (when Ald = **A**, than n = 2) can be written as follows.

- Formation of the first imine Im_1_ from aldehyde Ald and the first amine Am_1_:
$$\text{Ald}+n\;{\text{Am}}_{1}\;⇌{\text{ Im}}_{1}+n\;H_{2}O\;K_{e1}\;\left(\text{equilibrium constant}\right)$$
- First exchange:
$${\text{Im}}_{1}+n\;{\text{Am}}_{2}\;⇌{\text{ Im}}_{2}+n\;{\text{Am}}_{1}\;K_{e3}$$
- Acid-triggered exchange:
$${\text{Im}}_{2}+n\;{\text{Am}}_{1}+n\;H_{3}O^{+}\;⇌{\text{ Im}}_{1}+n\;{\text{Am}}_{2}H^{+}+n\;H_{2}O\;K_{e4}$$
  If the protonation of amine Am_1_ is considered, the reaction is as follows:
$${\text{Im}}_{2}+n\;{\text{Am}}_{1}H^{+}\;⇌{\text{ Im}}_{1}+n\;{\text{Am}}_{2}H^{+}\;K_{e4p}$$
- Base-triggered exchange:

$${\text{Im}}_{1}+n\;{\text{Am}}_{2}H^{+}+n\text{ Base }⇌{\text{ Im}}_{2}+n\;{\text{Am}}_{1}+n\;{\text{BaseH}}^{+}\;K_{e5}$$

Considering the formation of the second imine

$$\text{Ald}+n\;{\text{Am}}_{2}\;⇌{\text{ Im}}_{2}+n\;H_{2}O\;K_{e2}$$

and the acidity constants:

$${\text{Am}}_{1}H^{+}+H_{2}O\;⇌{\text{ Am}}_{1}+H_{3}O^{+}\;K_{a1}$$

$${\text{Am}}_{2}H^{+}+H_{2}O\;⇌{\text{ Am}}_{2}+H_{3}O^{+}\;K_{a2}$$

$${\text{BaseH}}^{+}+H_{2}O\;⇌\text{ Base}+H_{3}O^{+}\;K_{\text{aB}}$$

the following equalities can be written

$$K_{e3}=K_{e2}/K_{e1}$$

$$K_{e4p}=\left(1/K_{e3}\right)\times {\left(K_{a1}/K_{a2}\right)}^{n}$$

and

$$K_{e5}=K_{e3}\times {\left(K_{a2}/K_{\text{aB}}\right)}^{n}$$

We notice that if $K_{a2}=K_{\text{aB}}$ , than $K_{e5}=K_{e3}$ .

In this simplified approach, equilibriums like dissociation of the acid, intermediate reactions (formation of monoimine from dialdehyde, of hemiaminals) or side reactions (formation of aminals, of aldehyde hydrates), possible partial protonation of the N_pyridine_ atom, have not been written.

In the case of a dialdehyde (n = 2), starting from a 1:2 mixture of dialdehyde Ald and amine Am_1_, and assuming the only source of water would be that generated in this reaction, for a conversion of 90% of Ald into bis-imine Im_1_, with 5% of monoimine (or aldehyde-imine, not shown), K_e1_ should be of about 2.7 × 10^3^. Further, if to the previous equilibrated mixture one adds 2 equiv. of amine Am_2_, then, for the same conversion of 90% of Im_2_ with respect to the starting dialdehyde Ald and with 5% of mixed bis-imine (derived from Am_1_, Am_2_ and Ald; not shown) and 1% of monoimine (from Am_2_ and Ald), K_e2_ should be of about 1.1 × 10^7^ (here, one assumes that the other Ald-based species in the equilibrated reaction mixture are only Ald itself (if still any), the bis-imine Im_1_ and the aldehyde-amine from Am_1_). Under similar conditions, but with conversions of 99% instead of 90%, 0.5% instead of 5% and 0.1% instead of 1%, one should have K_e1_ ≈ 3.5 × 10^6^ and K_e2_ ≈ 1.7 × 10^13^ (see SM, file l-simul-Ke1-Ke2).

pK_a_ values of primary alkylamines and of tertiary trialkylamines in water are generally between 10 and 11 (Hall, 1957). p-Toluidine has a pK_a_ of 5.07, p-methoxy-aniline one of 5.29 and m-methoxy-aniline, one of 4.20 (Brown et al., 1955). These values, although for water, suggest that aliphatic amines **L1-L5**, aromatic ones **R1-R4** (Figure 7) and triethylamine TEA should be, thank to the differences between their pK_a_ values, good candidates for the implementation of such acid/base-triggered switches. In addition, in an organic solvent like CH_3_CN, primary aliphatic amines, such as n-propylamine and n-butylamine are more nucleophilic than p-anilines, like p-toluidine and p-anisidine. Indeed, in acetonitrile, n-butylamine has an N_parameter_ of 15.27 and n-propylamine, one of 15.11 (Kanzian et al., 2009), while p-toluidine displays 13.19 and p-anisidine, 13.42 (Brotzel et al., 2007). The nucleophilicity of amines is, of course, relevant here, because the mechanism of imine formation (Ciaccia and Di Stefano, 2015), as well as that of imine/amine exchange starts with a nucleophilic attack of the amine to the C=O or C=N bond. One may however notice that the nucleophilicity is not always in correlation with the basicity.

### 2.2 Synthesis

**FIGURE 8:**
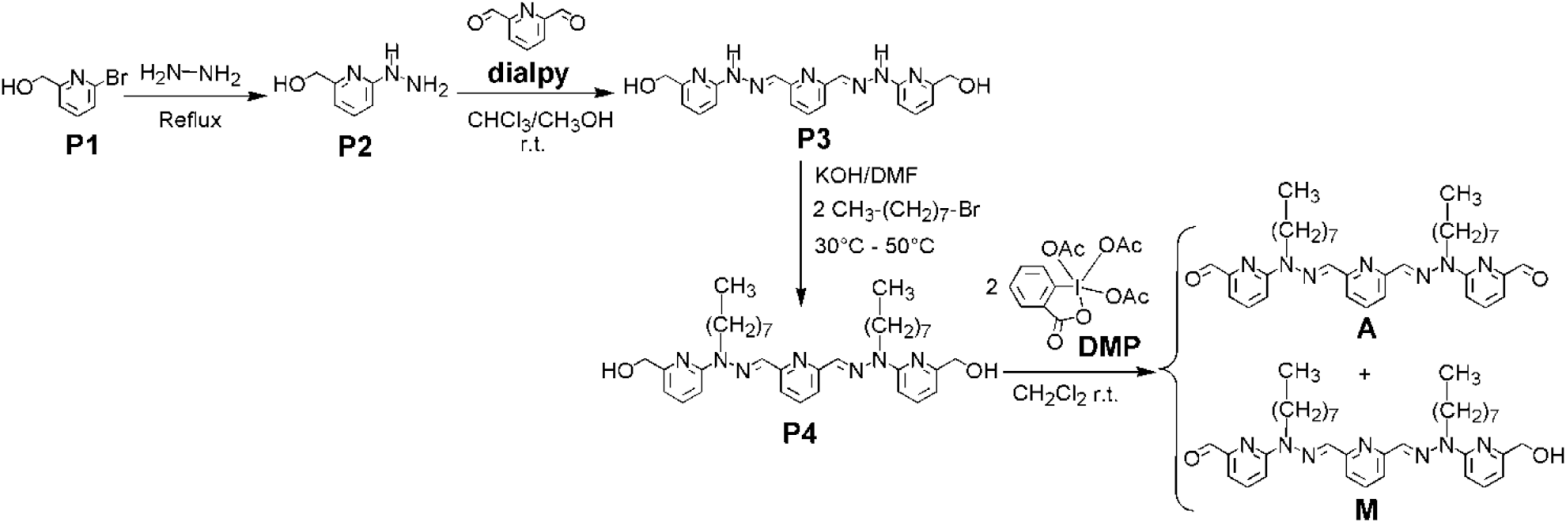
Synthesis of monoaldehyde **M** and of dialdehyde **A**. **DMP** = Dess-Martin periodinane.

2,6-Pyridinedicarboxaldehyde **dialpy** was obtained through oxidation of 2,6-di(hydroxymethyl)pyridine with selenium dioxide SeO_2_ (Alam, 2011). Reaction of 2-bromo-6-hydroxymethyl-pyridine **P1** with hydrazine under reflux produces the corresponding hydrazino-alcohol **P2**. 2 equiv. of compound **P2** react with 1 equiv. of 2,6-pyridinedicarboxaldehyde and generate the corresponding dihydrazone **P3**. Its NH groups are further alkylated with n-octylbromide (in the presence of KOH in DMF), in order to ensure a good solubility. The resulting diol **P4** (for a related compound, see (Lobo et al., 2019)) was reacted with the Dess-Martin periodinane (Dess and Martin, 1983) in order to oxidize the CH_2_OH groups to aldehydes (Ulrich et al., 2009). This produced a mixture of monoaldehyde **M** and dialdehyde **A** (Figure 8; see SM, file synt-char, pp. 3–108).

Amines **R1**, **R2** and **R4** were prepared from 4- or 3-hydroxyacetanilide according to methods described in the literature (Bartulin et al., 1986; Sharma et al., 2017).

### 2.3 Imine formation

The reactions, mainly performed in CDCl_3_ for the sake of the solubility (and only in a few cases in CD_3_CN), were monitored through ^1^H NMR.

Aromatic (bis-)imines used as first (starting) bis(imines) can be beforehand synthesized and isolated (by precipitation, like in the case of **AR3**_**2**_) or obtained in the NMR tube and used without isolation. In the last situation, an excess of aromatic amine may be added in order to increase the yield of target (bis-)imine (like for the reaction of dialdehyde **dialpy** with amines **R3** or **R4**, vide infra). This excess can be calculated (vide infra, the calculations on constants) or added by titration until a satisfactory yield of (bis-)imine is reached. For example, a reaction mixture of **dialpy** (about 6.2 mM in CDCl_3_; r.t.) with **R3** (2 equiv., 1 week) contains about 61–64% of bis-imine, while that with **R4** (2 equiv., about 3 days) has about 80% of bis-imine. Addition of an excess of amine (2.5 equiv. of **R3**; 0.75 equiv. of **R4**; about 12 days at r.t.), produces yields of bis-imine higher than 90%.

The yields of imine formation depend, of course, amongst other factors, also on the amount of water (vide infra, Section 5.2.4).

Uncertainties associated to equilibrium constants K_1_ and K_2_ were, roughly, estimated to be, on average, of about ±20% to ±40% and they should be higher for constants K_e1_.

#### 2.3.1 Imines from aldehyde **M**

**FIGURE 9:**
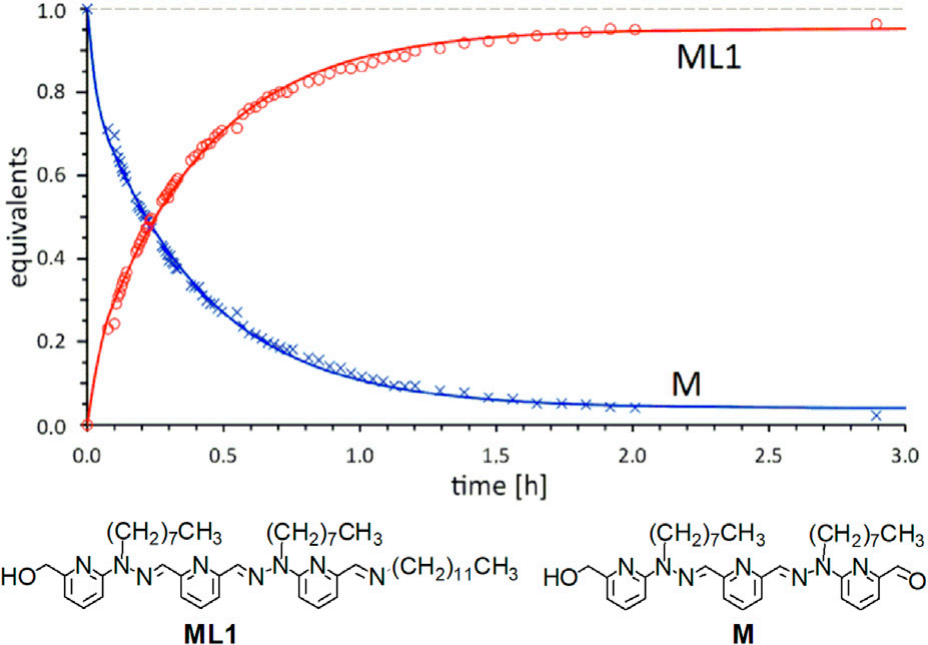
Kinetic study through ^1^H NMR (CDCl_3_, 500 MHz) of the reaction of monoaldehyde **M** with aliphatic amine **L1**.

Reaction of monoaldehyde **M** (Figure 7) with 1 equiv. of aromatic amine **R1** produces the imine **MR1** with K_e1_ = 10 (yield: 57%). With the aliphatic amine **L1**, the reaction (Figure 9) of the monoaldehyde **M** is relatively fast (t_1/2_ = 15 min) and almost quantitative (98%), for a much higher equilibrium constant K_e1_ = 9.8 × 10^3^. For examples of reactions between aliphatic amines and pyridinealdehydes, see (French and Bruice, 1964; Green and Rogerson, 1968).

#### 2.3.2 Bis-imines from dialdehyde **A**

Dialdehyde **A** reacted with 2 equiv. of aromatic amine **R2** to form the corresponding bis-imine **AR2**_2_ with a roughly estimated K_e1_ of 55 (yield: 66%) and a half-reaction time t_1/2_ of about 4 h. Reactions of dialdehyde **A** with aliphatic amines **L1** (n-dodecylamine) and **L2** (n-hexylamine) were much faster (t_1/2_ of about 10–15 min). The conversion to bis-imine was significantly higher (96%–97%), where K_e1_ for **L1** is of 5.7 × 10^6^, and that for **L2**, of 2.8 × 10^6^. These data are in agreement with the idea that the aliphatic amines react faster than the aromatic ones and their imines have formation constants higher than those of the aromatic ones.

#### 2.3.3 Bis-imines from dialdehyde **dialpy**

A good precursor of Schiff bases is 2,6-pyridinedicarboxaldehyde **dialpy** (vide supra). For the formation of bis-imine **dL4**_2_ from **dialpy** and decylamine **L4**

$$\text{dialpy}+2L4\;⇌\;\text{dL}4_{2}+2H_{2}O$$

K_e1_ is greater than 10^7^, while for the reaction of **dialpy** with p-toluidine **R3**

$$\text{dialpy}+2R3\;⇌\;\text{dR}3_{2}+2H_{2}O$$

one has K_e1_ = 10.9 × 10^3^, and for the reaction of **dialpy** with 4-(hexyloxy)aniline **R4**

$$\text{dialpy}+2R4\;⇌\;\text{dR}4_{2}+2H_{2}O$$

one has K_e1_ = 14.4 × 10^4^.

For the formation of bis-imines from dialdehyde **dialpy** and amines **L4** and **R4**, the formation constant of the bis-imine from aldehyde-imine (monoimine) and amine is lower than the formation constant of the aldehyde-imine (monoimine) from dialdehyde and amine, as suggested by the following reactions

$$\text{dialpy}+R4\;⇌\text{ aldehyde}-\text{imine}+H_{2}O\;\left(K_{1}=9.0\;x\;10^{2}\right)$$

$$R4+\text{aldehyde}-\text{imine }⇌\;\text{dR}4_{2}+H_{2}O\;\left(K_{2}=1.6\;x\;10^{2}\right)$$

and with amine **L4**

$$\text{dialpy}+L4\;⇌\text{ aldehyde}-\text{imine}+H_{2}O\;\left(K_{1}=15.0\;x\;10^{3}\right)$$

$$L4+\text{aldehyde}-\text{imine }⇌\;\text{dL}4_{2}+H_{2}O\;\left(K_{2}=1.8\;x\;10^{3}\right)$$

### 2.4 Transfer of structural units through transiminations (exchanges)

**FIGURE 10:**
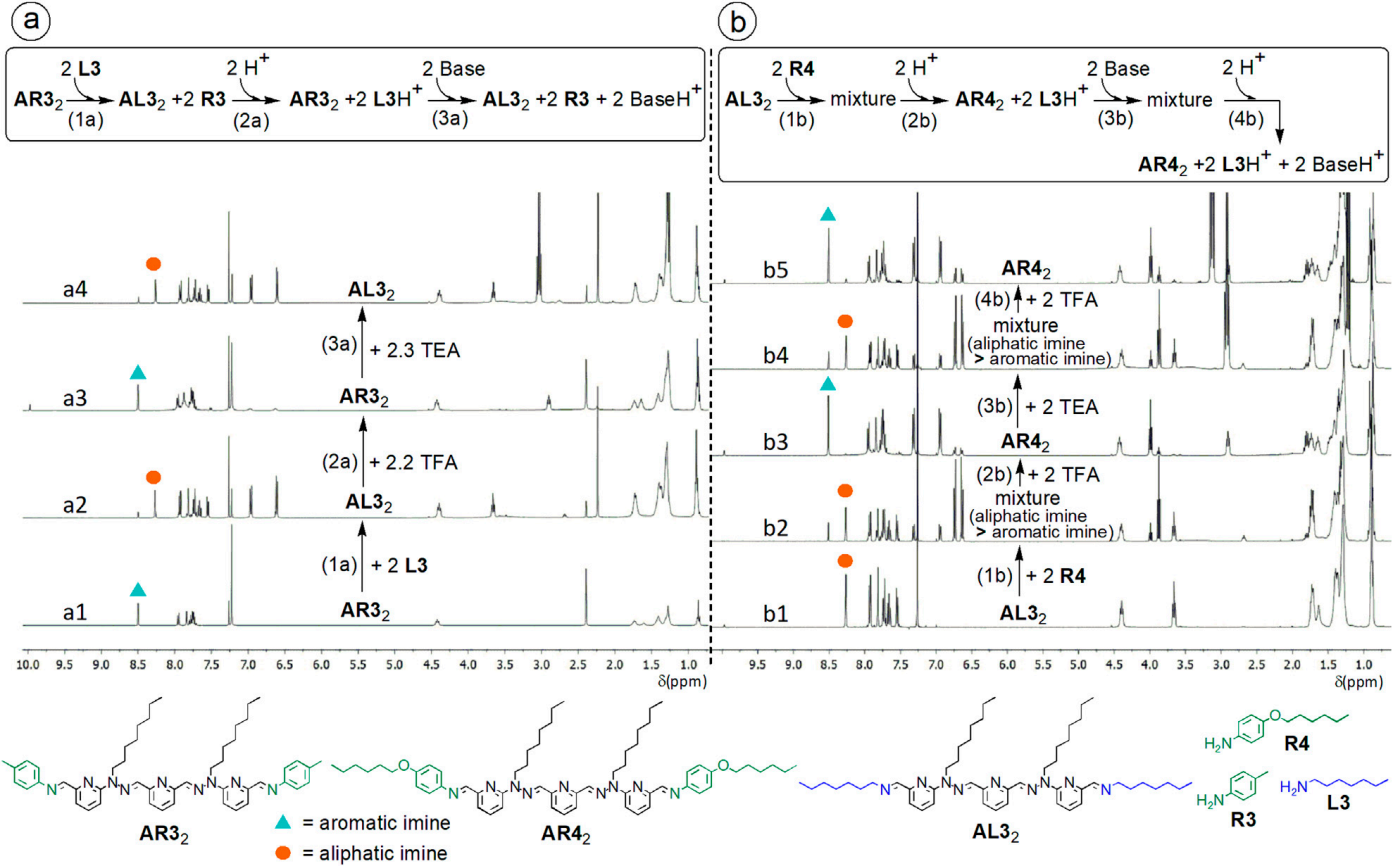
^1^H NMR-monitoring (CDCl_3_, 400 MHz) of **(a)** covalent constitutional switches between bis-imines **AR3**_2_ and **AL3**_2_ (addition (1a) of aliphatic amine **L3** (a2) to bis-imine **AR3**_2_ (a1) followed by addition (2a) of trifluoroacetic acid (TFA) (a3), then by addition (3a) of base (triethylamine TEA) (a4)), and of **(b)** covalent constitutional switches between bis-imines **AL3**_2_ and **AR4**_2_ (addition (1b) of aromatic amine **R4** (b2) to bis-imine **AL3**_2_ (b1) followed by addition (2b) of TFA (b3), then by addition (3b) of TEA (b4), and again by addition (4b) of TFA (b5)). In this example, compounds **AL3**_2_ and **AR3**_2_ were not prepared in the NMR tube, but they were beforehand synthesized and isolated.

**FIGURE 11:**
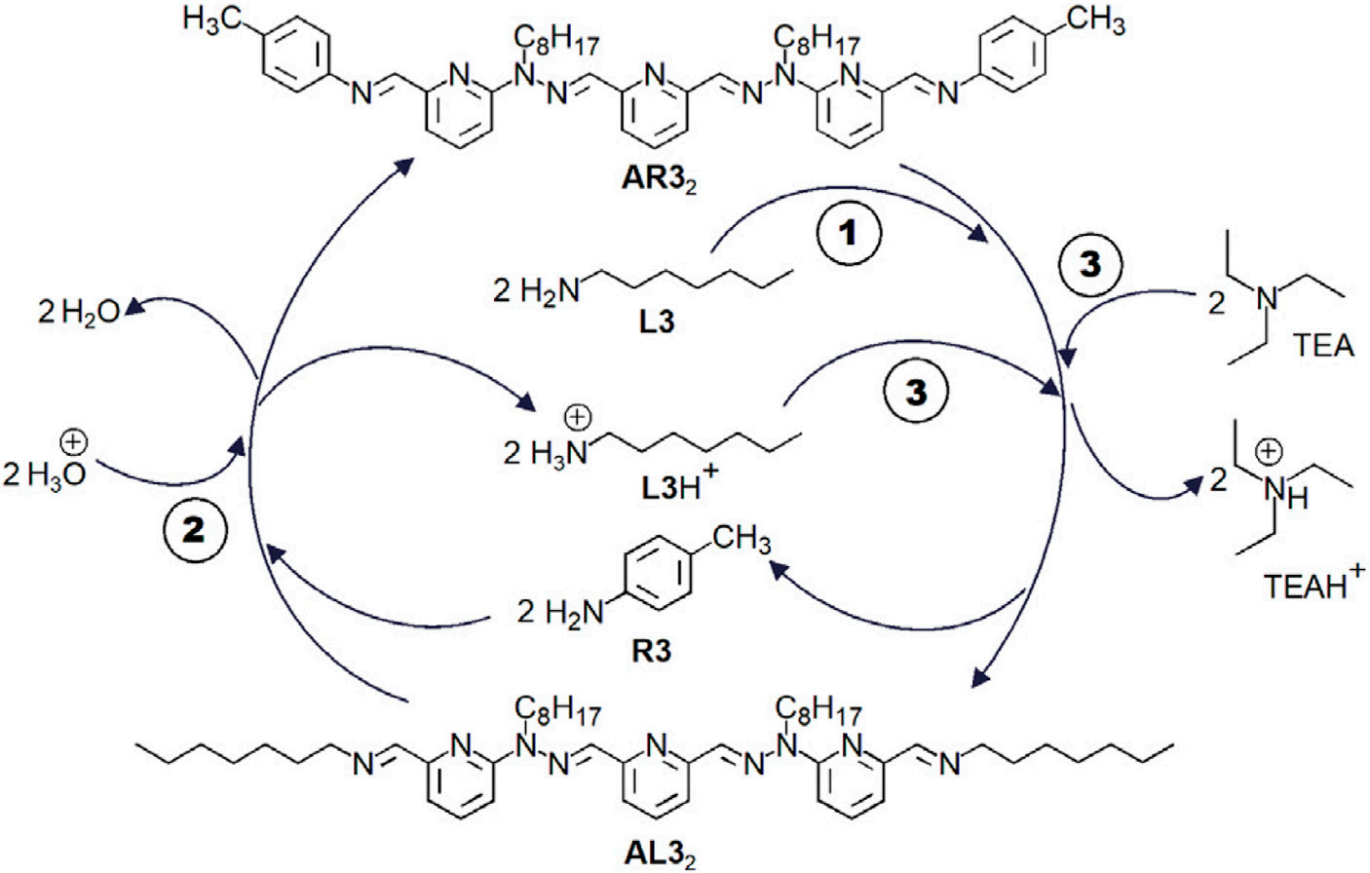
Cyclic representation of the switch presented in Figure 10a. In the first step (1), reaction of the bis-imine **AR3**_2_ with the aliphatic amine **L3** (n-heptylamine) produces the bis-imine **AL3**_2_ and the aromatic amine **R3** (p-toluidine). In the second step (2), addition of trifluoroacetic acid (TFA) to the reaction mixture regenerates the bis-imine **AR3**_2_. In the third step (3), addition of triethylamine (TEA) to the reaction mixture produces the bis-imine **AL3**_2_ together with amine **R3** (p-toluidine).

**FIGURE 12:**
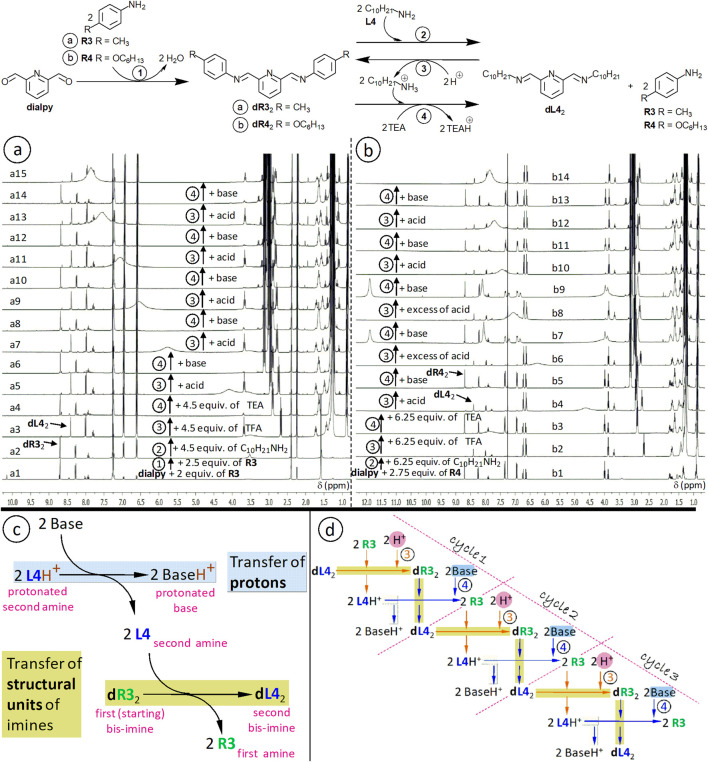
**(a,b)** ^1^H NMR spectra (CDCl_3_, 400 MHz) corresponding to the exchanges from the scheme at the top: transiminations between monoamines and dialdehyde-derived bis-imines, as covalent switches triggered by alternate additions of acid and base **(a,b)**. Here, the starting bis-imines (**dR3**_2_ and **dR4**_2_) are prepared (1) without being isolated, from 2,6-pyridinedicarboxaldehyde **dialpy** and an excess of aromatic amine, p-toluidine **R3** (a1, a2) or 4-(hexyloxy)aniline **R4** (b1); aromatic bis-imine yields (after addition of excesses of aromatic amines) > 90%; spectra a2 and b1 were recorded after about 12 days of equilibration. The aliphatic amine is the n-decylamine **L4** (2; a3, b2); aliphatic bis-imine yields (after exchange, but before addition of acid and base) > 85%. There are 6 acid-base cycles (12 alternate acid/base additions, (3) and (4), spectra a3-a15 and b2-b14; yields between 65% and 90%). An accidentally added excess of acid (spectra b7 and b9) can be corrected by addition of the appropriate amount of base in the next step. **(c)** Representation of the reactions triggered by addition of base, showing that the transfer of protons from the primary aliphatic ammonium ion to the base, generates the aliphatic amine and, so, it is subsequently associated to, here, a transfer of dialdehyde-derived structural units. **(d)** Sequential representation of 3 of the 6 acid-base cycles from **(a)**. Starting concentrations: about 6.2 mM.

The work done in this subsection is focused mainly on the starting aromatic-aromatic- (e.g. **AR**_2_) and final aliphatic-aliphatic-bis-imines (e.g. **AL**_2_) from each reaction (Figures 10–12), rather than on the likely intermediate mixed aliphatic-aromatic-bis-imines (**ALR**).

The exchanges can be followed by ^1^H DOSY NMR (see SM, file synt-char, pp. 120–121).

#### 2.4.1 Bis-imines with amines

The global equation of the reaction of 2 equiv. of n-heptylamine **L3** with 1 equiv. of bis-imine **AR3**_2_ (beforehand synthesized from dialdehyde **A** and 2 equiv. of p-toluidine **R3**) and that with 1 equiv. of bis-imine **AR4**_2_ (obtained from A and 2 equiv. of p-(hexyloxy)aniline **R4**), can be written as follows:

$$\text{AR}3_{2}+2\;L3\;⇌\;\text{AL}3_{2}+2\;R3\;K_{e3}=263$$

$$\text{AR}4_{2}+2\;L3\;⇌\;\text{AL}3_{2}+2\;R4\;K_{e3}=25$$

On that basis, one can calculate the equilibrium constant for the reaction

$$\text{AR}3_{2}+2\;R4\;⇌\;\text{AR}4_{2}+2\;R3\;K_{e3}\approx 11$$

This example confirms a fact that should generally be true: considering the same bis-imine (here **AR3**_2_), the equilibrium constant for the cases where the displacing amine is an aliphatic one (like here, **L3** *n*-C_7_H_15_NH_2_), is higher than where the displacing amine is an aromatic one (here, p-(hexyloxy)aniline **R4**).

The equilibration of such exchange reactions (aliphatic amine + aromatic (bis-)imine) usually requires several hours.

The increase of the amount of displacing amine, produces an increase, in agreement with Le Chatelier’s principle, of the yield. For example, for the reaction

$$\text{AR}3_{2}+2\;L3\;⇌\;\text{AL}3_{2}+2\;R3\;K_{e3}$$

the yield was of about 95% for 3.6 equiv. of *n*-heptylamine **L3**, while it was of about 87% for 2 equiv. of *n*-heptylamine.

#### 2.4.2 Addition of acid

In the last situation, subsequent addition of 2.2 equiv. of trifluoroacetic acid (TFA; Figure 10a), partly regenerates the starting bis-imine, according to the chemical equation:

$$\text{AL}3_{2}+2\;R3+2\;H_{3}O^{+}\;⇌\;\text{AR}3_{2}+2\;L3H^{+}+2\;H_{2}O\;K_{e4}$$

or, considering the protonation of aromatic amines (although-under these conditions, where **R3** is an aromatic and **L3** an aliphatic one-very weak)

$$\text{AL}3_{2}+2\;R3H^{+}\;⇌\;\text{AR}3_{2}+2\;L3H^{+}\;K_{e4p}=10^{2\left(\text{pKa}2-\text{pKa}1\right)}/K_{e3}$$

with a yield of 68%, the reaction being quite fast (a few minutes). The protonation inverts the sense of the selectivity. Indeed, without acid, the aliphatic imine forms preferentially, while in presence of acid, the favored imine is the aromatic one (Stadler, 2013). For the amines used in our work, the difference ΔpK_a_ = pKa_2_-pKa_1_ is of about 5–6 units, in water. The pK_a_ values may be different in the cases herein reported, where the solvent is CDCl_3_.

Under these conditions, trifluoroacetic acid (TFA) not only acts as a protonating agent of - mainly - aliphatic amines, but it also catalyzes the exchanges. Indeed, the equilibration after addition of acid is particularly fast. Partial protonation of species (e.g. primary aromatic amines, pyridines) other than the primary aliphatic amine might also occur. This can, together with the chemical exchanges, affect the NMR chemical shifts of the species in solution. This should explain the slight differences between the ^1^H NMR spectrum of the starting bis-imine and that obtained after addition of TFA (Figure 10a). The yield of this step may often be lower than those of other steps, and this can be due to acid-catalyzed hydrolytic processes. The excess (if any) of aromatic amine added in the initial steps can limit such hydrolytic effects.

#### 2.4.3 Addition of base

Further addition of an organic base, triethylamine (TEA; in practice, usually, between 1 and 1.4 equiv. of base per equiv. of acid), regenerates the aliphatic bis-imine

$$\text{AR}3_{2}+2\;L3H^{+}+2\text{ TEA }⇌\;\text{AL}3_{2}+2\;R3+2\;{\text{TEAH}}^{+}\;K_{e5}$$

with a yield of 92%. The sequence of pH-triggered switches can be represented in a cyclic manner (Figure 11). For two related examples, see Figures 12a, b.

The transfer of protons from the protonated second amine (e.g. **L4**H^+^) to the base (e.g. Et_3_N = TEA) makes possible—through transimination—the transfer of dialdehyde-derived structural units. This is represented here (Figure 12c) for the conversion of bis-imine **dR3**_2_ into bis-imine **dL4**_2_. The selectivity of imine formation is alternately reversed through addition of acid, than of base.

The aliphatic amines (e.g. n-hexyl-, n-heptyl- and n-dodecylamine) used as second amine to replace the first amine, have similar pK_a_ values (10.56 (Patel and Higuchi, 1980), 10.66 (Carlson et al., 1988) and 10.63 (Zhou et al., 2018)), which are close to that of TEA (10.75) (Tao et al., 2021) (values for water). In our previous work (Stadler and Lehn, 2014) we already used TEA as a basic trigger.

Acid and base are added as solutions in small volumes of solvent (chloroform-d_3_). One may first add 90%–95% of the calculated amount, then progressively, by titration, a bit less or a bit more than the remaining amount, until a convenient yield is reached. A same mixture of components (here, the one obtained after addition of the second amine) can be used to reversibly generate—upon addition of two different, alternate stimuli—two constitutional states, namely two alternate mixtures of constituents (which may be associated to two alternate properties). In this way, the mixture can be “reused” during a certain number of cycles. Such imine-based, pH-responsive switches, which undergo constitutional reorganization, can be seen as dynamic libraries with pH-adaptive behavior.

Similar sequences of covalent switches can also be performed even in reaction mixtures where the formation of the starting bis-imine is not complete, like in the example of dialdehyde **A **with m-(pentyloxy)aniline **R2** as a first amine, and hexylamine **L2**, as a second amine (see SM, file synt-char, p. 130).

#### 2.4.4 Number of acid-base cycles

It should be possible to perform pH-modulated transiminations a significant number of times. The switches fuelled through alternate additions of acid (CF_3_COOH = TFA) and base (Et_3_N = TEA) between **dR3**_**2**_ and **dL4**_**2**_, and those between **dR4**_**2**_ and **dL4**_**2**_ have been performed 12 times (6 cycles; only the acid-base cycles are considered here) (Figures 12a,b). The starting aromatic bis-imines **dR3**_**2**_ and **dR4**_**2**_ were prepared in the NMR tubes. Aromatic and aliphatic amines are added in excess with respect to the dialdehyde **dialpy**. These are typical examples of the use of an excess of amine for the preparation of dialdehyde-derived bis-imines in NMR tubes.

If too much acid or base is accidentally added in a step, this excess can be corrected in the subsequent step (Figure 12b, spectra b7-b10).

The successive pH-triggered interconversions can be represented in a cyclic manner (Figure 11), as well as in a non-cyclic way (Figure 12d).

### 2.5 Inversion of steps

The first step of the above presented switches is the formation of the aromatic imine or, if it has previously been isolated, its reaction with the aliphatic amine. So, usually, the first (bis-)imine is the aromatic one (Figures 11, 12).

It is however possible to start with the aliphatic amine (or imine). The interest of this procedure is that aliphatic imines generally forms faster than aromatic ones and so, the starting aliphatic imine can rapidly be obtained in the NMR tube. Moreover, the subsequent step of the covalent switch (formation of the aromatic amine in the presence of TFA through transimination) is quite fast, thanks to acid catalysis.

**FIGURE 13:**
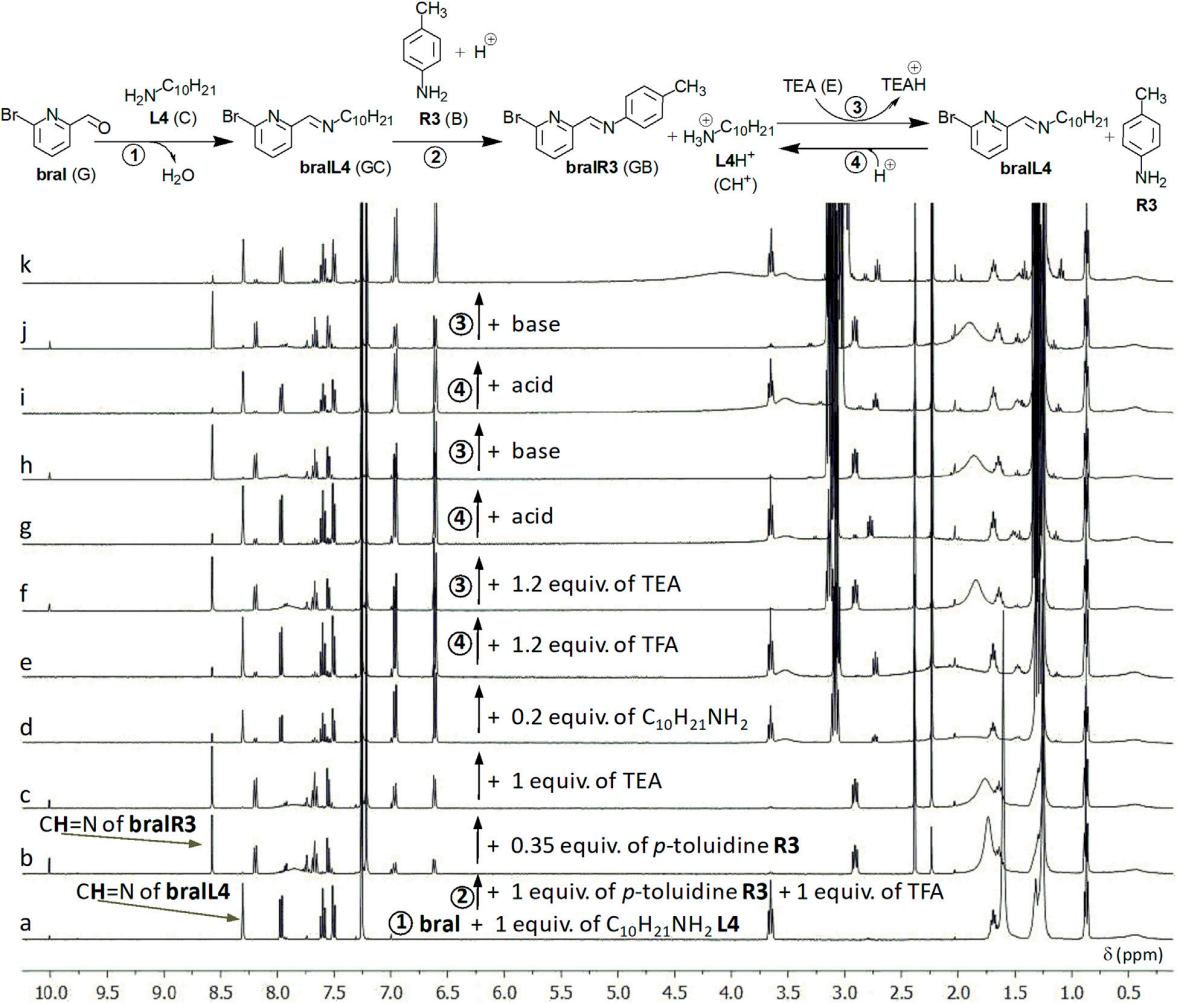
^1^H-NMR-monitored (CDCl_3_, 400 MHz) inversion of steps for an imine/amine exchange. After formation (1) of the aliphatic imine **bralL4** from the aromatic aldehyde **bral** and decylamine **L4** [first step, **(a)**], aromatic imine **bralR3** (2) forms on addition of p-toluidine in the presence of the acid TFA **(b,c)**. After neutralization of the acid with the base TEA **(d)**, an excess of aliphatic amine **L4** leads to the desired amount of aliphatic imine **(e)**. Further, alternate additions of acid (4) and base (3) modulate the switch (6 alternate additions, 3 cycles; spectra e-**k**). With respect to the text (Section 2.5), the notations are: G = **bral**, B = **R3** (p-toluidine), C = **L4** (decylamine) and E = TEA (N(C_2_H_5_)_3_). Here: β = 0.35 equiv. and γ = 0.2 equiv. See text, Section 2.5.

In the example from Figure 13, an aromatic aldehyde G (here, **bral**) (1 equiv.) reacts with one equivalent of aliphatic amine C (here C_10_H_21_NH_2_ = **L4**) and forms the imine GC (=**bralL4**, Figure 13a). After equilibration, one equivalent of aromatic amine B (here, p-toluidine **R3**) is added, followed by one equivalent of acid (TFA, Figure 13b). After a new equilibration, one adds a supplementary amount of B (β equiv.) until the desired amount of imine GB (=**bralR3**) is reached at equilibrium. Then, one adds one equivalent of base E (here, TEA = triethylamine). After equilibration (Figure 13d), one adds a supplementary amount of amine C (γ equiv.), until the desired amount of imine GC is reached (Figure 13e). To the equilibrated mixture, one adds an amount of acid that corresponds to the total amount of amine C (namely, in principle, 1 + γ equiv.; Figure 13f). After subsequent equilibration, one adds the amount of base E required to neutralize the previous amount of acid and to reach the previous amount of imine GC (Figure 13g). In our example, the alternate additions of acid and base were continued, each of them being done two more times, and, in this way, 2 supplementary cycles were performed (Figures 13g–k).

A simplified inversion of steps was used for the pH-triggered switch between the bis-imines **AL3**_**2**_ and **AR4**_**2**_ (Figure 10b), where the starting bis-imine was the aliphatic one, **AL3**_**2**_. This bis-imine was beforehand synthesized and isolated, and no excess of amine was added in the NMR tube.

## 3 Exchanges between (mono-, bis-, tris-)imines and (di)aldehydes in solution

### 3.1 Overview and examples

Such exchanges correspond, in the case of a monoimine Im_1_ (obtained from an amine Am and an aldehyde Ald_1_) and an aldehyde Ald_2_, to the global equation:

$${\text{Im}}_{1}+{\text{Ald}}_{2}\;⇌{\text{ Im}}_{2}+{\text{Ald}}_{1}$$

They should most likely occur through the intermediate hydrolysis of the starting imine, followed by the subsequent reaction of the resulting amine with the second aldehyde

$${\text{Im}}_{1}+H_{2}O\;⇌\text{ Am}+{\text{Ald}}_{1}$$

$$\text{Am}+{\text{Ald}}_{2}\;⇌{\text{ Im}}_{2}+H_{2}O$$

thus leading to an overall process of transimination.

In an amine/imine exchange (vide supra), takes place the transfer of the aldehyde-derived moiety, while in an aldehyde/imine exchange, the moiety that is transferred, is the amine-derived one.

We explored such transiminations by performing the following kinds of (bis-, tris-)imine/(di)aldehyde exchanges in solution.

**FIGURE 14:**
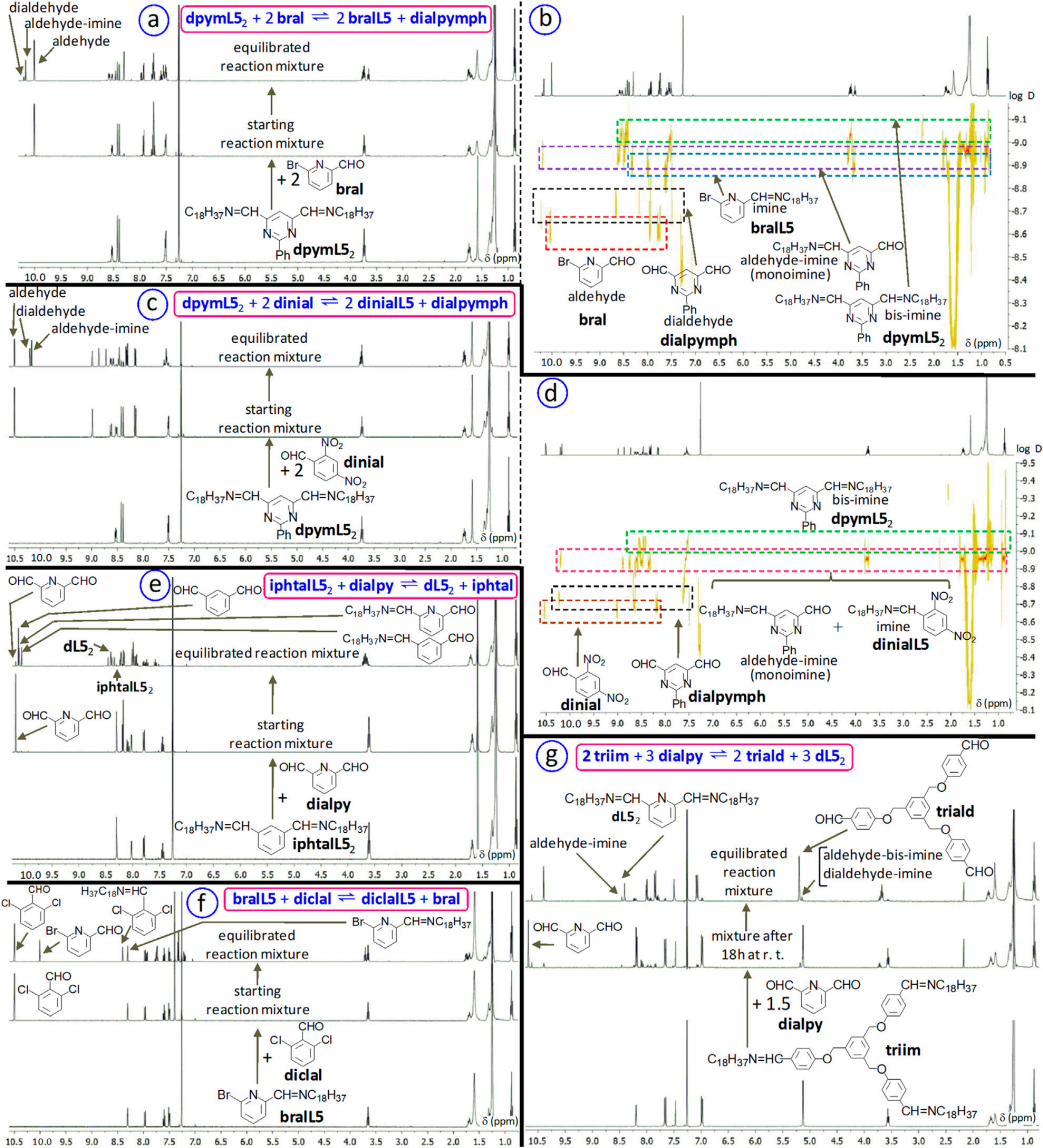
Exchanges between (bis-)imines and (di)aldehydes in solution. **(a,c,e,f,g)** ^1^H NMR spectra (CDCl_3_, 400 MHz) of the reaction mixtures of: **(a, c)** the bis-imine **dpymL5**_2_ with 2 equiv. of 6-bromo-2-pyridinecarboxaldehyde **bral** **(a)** or with 2 equiv. of 2,4-dinitrobenzaldehyde **dinial** **(c)**; **(e)** the bis-imine **iphtalL5**_2_ with 1 equiv. of 2,6-pyridinedicarboxaldehyde **dialpy**; **(f)** the imine **bralL5** with 1 equiv. of 2,6-dichlorobenzaldehyde **diclal**; **(g)** the tris-imine **triim** (from trialdehyde **triald** and octadecylamine) with 1.5 equiv. of dialdehyde **dialpy**. **(b,d)** ^1^H DOSY NMR spectra (CDCl_3_, 500 MHz) of the equilibrated reaction mixtures from **(a)** and **(c)**: a → b, c → d. D is the diffusion coefficient and its unit of measurement is m^2^s^-1^. The percentages of starting (bis-, tris-)imines which remain unreacted at room temperature are, roughly: **(a)** 48% after 20 days, **(c)** 18% after 20 days, **(e)** 5% after 2 months, **(f)** 56% after 20 days and **(g)** < 1% after 20 days. Starting concentrations: between 3 mM and 6.6 mM.

- dialdehyde-based bis-imine with aldehyde, exemplified here by the reaction of the bis-imine **dpymL5**_**2**_ (from 2-phenyl-4,6-pyrimidinedicarboxaldehyde **dialpymph** and octadecylamine **L5**), with i.a) 2 equiv. of 6-bromo-2-pyridinecarboxaldehyde **bral**, where forms the target imine **bralL5** (30% after 20 days; Figures 14a,b):
$$\text{dpymL}5_{2}+2\;\text{bral }⇌\;2\;\text{bralL}5+\text{dialpymph}$$
  or with i.b) 2 equiv. of 2,4-dinitrobenzaldehyde **dinial**, where is generated the target imine **dinialL5** (57% after 20 days; Figures 14c,d):
$$\text{dpymL}5_{2}+2\;\text{dinial }⇌\;2\;\text{dinialL}5+\text{dialpymph}$$
- dialdehyde-based bis-imine with dialdehyde, illustrated here by the reaction of the bis-imine **iphtalL5**_2_ (from isophtalaldehyde and octadecylamine), with 1 equiv. of 2,6-pyridinedicarboxaldehyde **dialpy**, with formation of target bis-imine **dL5**_2_ (41% after 2 months; Figure 14e):
$$\text{iphtalL}5_{2}+\text{dialpy}⇌\text{dL}5_{2}+\text{iphtal}$$
- imine with aldehyde, the example presented here being the reaction of the imine **bralL5** (from 6-bromo-2-pyridinecarboxaldehyde **bral** and octadecylamine **L5**), with 1 equiv. of 2,6-dichlorobenzaldehyde **diclal**, the target imine being **diclalL5** (44% after 20 days; Figure 14f; see SM, file synt-char, p. 123):
bralL5+diclal⇌diclalL5+bral
- trialdehyde-based tris-imine with dialdehyde, exemplified here by the reaction of the starting tris-imine **triim** (from trialdehyde **triald** (Rajakumar et al., 2006) and octadecylamine **L5**; see SM, file synt-char, p. 100), with 2,6-pyridinedicarboxaldehyde **dialpy** (Figure 14g):
$$2\;\text{triim}+3\;\text{dialpy}⇌2\;\text{triald}+3\;\text{dL}5_{2}$$

The target bis-imine is, here, **dL5**_2_ (65%–70% after 20 days). In the equilibrated reaction mixture of **triim** with 1.5 equiv. of **dialpy**, the intermediates between **triim** et **triald**, namely the aldehyde-bis-imine (about 5%) and the dialdehyde-imine (about 23%), are also present along with the trialdehyde **triald** (about 70%–72%); they were roughly quantified through ^1^H NMR thanks to the resonances of CH_2_O groups. Trialdehydes (Nakada et al., 2017) are promising candidates for imine-based dynamic architectures, including cages (Giri et al., 2023; Pattillo and Moore, 2019) and fluorodynamers (Si et al., 2020).

Starting (mono-, bis-, tris-)imines were synthesized through a simple solvent-free procedure, where (mono-, di-, tri-)aldehydes were reacted, under solvent-free conditions, at 65°C–95°C, for, usually, 18–22 h, with the corresponding amines (vide infra, Section 4; see Supplementary Material, file synth-char, p. 4).

Exchange reactions were followed by 1D ^1^H NMR and, quite often, by ^1^H DOSY NMR (Figures 14b,d), that is a useful tool for the characterization of multicomponent reaction mixtures.

The differential of the number of imine groups between the starting imine-containing species and the target one is, here, 0, for reactions (ii) (bis-imine → bis-imine) and (iii) (imine → imine), or 1, for reactions (i) (bis-imine → imine) and (iv) (tris-imine → bis-imine). If the N atom of each imine bears a moiety possessing one of more (for example, n) functional groups, then the passage from a compound with p imines per molecule to one with q imines per molecule, leads to a differential (absolute value) of |p-q| imines and |n(p-q)| functional groups between the starting compound and the target one. This aspect can be of significance if the functional group plays a particular role, like a biological or an analytical one.

**FIGURE 15:**
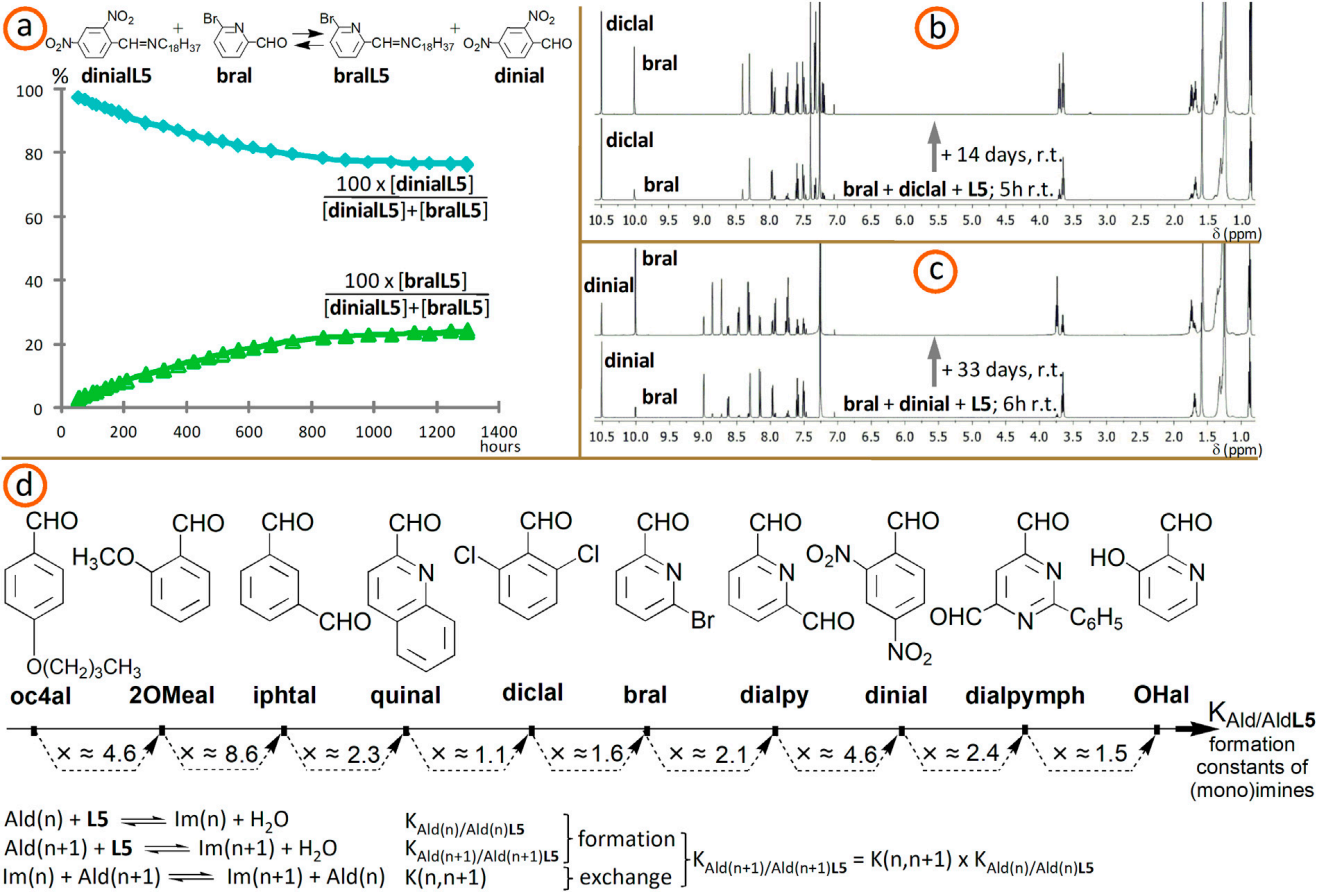
**(a)** Time-dependent representation of molar percentages of **dinialL5** and **bralL5** for the imine/aldehyde exchange between **dinialL5** (1 equiv.; obtained from **dinial** and 1 equiv. of **L5**, without solvent; 18 h, 70°C, >90%) and **bral** (1 equiv.) in CDCl_3_. **(b)** ^1^H NMR spectra (CDCl_3_, 500 MHz) for the reaction of 1 equiv. of **bral** and 1 equiv. of **diclal** with 1 equiv. of **L5**. **(c)** ^1^H NMR spectra (CDCl_3_, 500 MHz) for the reaction of 1 equiv. of **bral** and 1 equiv. of **dinial** with 1 equiv. of **L5**. Starting concentrations for **(a–c)**: 0.013-0.014 M; experiments done at r.t. **(d)**. Aromatic aldehydes roughly ordered according to formation constants of their aliphatic imines or, for dialdehydes, monoimines (amine: **L5**; solvent: chloroform-d_3_). The values given under the main arrow are those of exchange constants K(n,n+1).

Results obtained in solution at room temperature suggest that the equilibration generally requires days, weeks or even months (Figure 15a), at room temperature (r.t.), without catalyst (of course, acid traces which may form in CDCl_3_ may affect the reaction rate).

In preliminary attempts, we tested dihexylammonium p-toluenesulfonate (prepared from the corresponding amine and acid), as catalyst for imine/aldehyde exchanges, on the reaction between **dinialL5** and **bral** (that requires, without catalyst, about 6 weeks to reach the equilibrium at r.t.; Figure 15a). The promising results thus obtained will be deepened in further experiments.

The means to modulate these exchanges through external stimuli (like the variations of the pH) remains to be found. For an example of supramolecular modulation of imine dynamic libraries, see Gambaro et al. (2020).

### 3.2 Order of aldehydes according to the formation constants of their aliphatic imines

The design of successive transiminations (switches or other related processes under equilibrium conditions) requires the knowledge of an at least qualitative and relative order of amines and aldehydes according to the formation constants of their imines. To this purpose, one can use exchange experiments, like in the following example.

In a typical example, the equilibrium constant for the exchange equilibrium

bralL5+diclal⇌bral+diclalL5

was found, through NMR spectroscopy, to be K_iii_ = 6.2 × 10^−1^.

If K_bral/bralL5_ is associated to the equilibrium

$$\text{bral}+L5⇌\text{bralL}5+H_{2}O$$

and K_diclal/diclalL5_ is associated to the equilibrium

$$\text{diclal}+L5⇌\text{diclalL}5+H_{2}O$$

then one has K_iii_ = K_diclal/diclalL5_/K_bral/bralL5_, which gives K_diclal/diclalL5_ = K_iii_K_bral/bralL5_ = 0.62K_bral/bralL5_. The relative uncertainties associated to the constants were estimated to be, roughly, of about ±20%.

One determined the constants K_ia1_ = 5.0 × 10^−1^ for the equilibrium

$$\text{dpymL}5_{2}+\text{bral }⇌\text{aldehyde}-\text{imine}+\text{bralL}5$$

and K_ia2_ = 4.0 × 10^−2^ for the equilibrium

bral+aldehyde−imine⇌dialpymph+bralL5.

Let K_dialpymph/monoim_ be associated to the equilibrium

$$L5+\text{dialpymph}⇌\text{aldehyde}-\text{imine}+H_{2}O$$

and K_monoim/dpymL52_, to the equilibrium

$$L5+\text{aldehyde}-\text{imine}⇌\text{dpymL}5_{2}+H_{2}O$$

One obtains K_ia1_ = K_bral/bralL5_/K_monoim/dpymL52_ and K_ia2_ = K_bral/bralL5_/K_dialpymph/monoim_, or K_monoim/dpymL52_ = K_bral/bralL5_/K_ia1_ ≈ 2K_bral/bralL5_ and K_dialpymph/monoim_ = K_bral/bralL5_/K_ia2_ ≈ 25K_bral/bralL5_.

For the reaction

$$\text{dinial}+\text{dpymL}5_{2}⇌\text{aldehyde}-\text{imine}+dinialL5,$$

the equilibrium constant is K_ib1_ = 4.5, and for the equilibrium

dinial+aldehyde−imine⇌dialpymph+dinialL5,

one has the constant K_ib2_ = 4.2 × 10^−1^. If K_dinial/dinialL5_ is associated to the equilibrium

$$d\text{inial}+L5⇌dinialL5+H_{2}O,$$

then, one obtains K_ib1_ = K_dinial/dinialL5_/K_monoim/dpymL52_ and K_ib2_ = K_dinial/dinialL5_/K_dialpymph/monoim_, or K_dinial/dinialL5_ = K_ib1_K_monoim/dpymL52_ and K_dinial/dinialL5_ = K_ib2_K_dialpymph/monoim_.

With the above results K_monoim/dpymL52_ ≈ 2K_bral/bralL5_ and K_dialpymph/monoim_ ≈ 25K_bral/bralL5_, one obtains K_dinial/dinialL5_ = 4.5 × 2K_bral/bralL5_ ≈ 9K_bral/bralL5_ and K_dinial/dinialL5_ = 0.42 × 25K_bral/bralL5_ ≈ 10.5K_bral/bralL5_, expressions which lead to the average, rounded result K_dinial/dinialL5_ ≈ 9.8K_bral/bralL5_.

Now, taking into account the above results where K_diclal/diclalL5_ (=0.62K_bral/bralL5_), K_monoim/dpymL52_ (≈2K_bral/bralL5_), K_dinial/dinialL5_ (≈9.8K_bral/bralL5_) and K_dialpymph/monoim_ (≈25K_bral/bralL5_) are expressed as functions of K_bral/bralL5_, the following order of the constants of imine formation can be established:

$$K_{\text{diclal}/\text{diclalL}5}<K_{\text{bral}/\text{bralL}5}<K_{\text{monoim}/\text{dpymL}52}<K_{\text{dinial}/\text{dinialL}5}<K_{\text{dialpymph}/\text{monoim}.}$$

One notices that, for aliphatic amine **L5**, the constant of formation of the monoimine (aldehyde-imine) from the dialdehyde **dialpymph** is bigger than that of formation of bis-imine from monoimine. A similar trend, namely K_monoimine/bis-imine_ < K_dialdehyde/monoimine_ (the constant of the formation of the bis-imine from monoimine is lower than that of the formation of monoimine from dialdehyde), was observed for **dialpy** and amines **L4** and **R4** (vide supra). For dialdehydes **iphtal**, **dialpy** and **dialpymph**, the ratio K_dialdehyde/monoimine_/K_monoimine/bis-imine_ lies, when reacted with aliphatic amines, between 8 and 13 (average value of about 10.5).

During competing experiments (2 aldehydes + 1 amine; e.g. **bral** + **diclal** + **L5**, Figure 15b; **diclal** + **quinal** + **L5**; **bral** + **dinial** + **L5**; **dialpy** + **dinial** + **L5**, Figure 15c), we noticed that pyridine- and quinoline-derived aldehydes react faster than benzene-derived ones (with “kinetic” yields usually greater than 70%). The trend from this kinetic situation may be maintained (Figure 15b) or reversed (Figure 15c) at equilibrium.

On the basis of exchanges of this kind, as well as of competing experiments done on sets of two aldehydes and one amine (decyl- or octadecylamine), in CDCl_3_, and through calculations like those presented above, done on average values, we established a rough, qualitative order of some aldehydes, corresponding to the increase of formation constants of their aliphatic imines (for dialdehydes, is considered the formation of the monoimine; Figure 15d). Here, one looks at the thermodynamic aspect of imine formation, while in the precedent Section 2.1.3 (vide supra), one focused rather on the kinetic aspect.

Thus, exchange constants K(n,n+1) associated to the equilibrium

Ald(n+1)+Im(n)⇌Ald(n)+Im(n+1)

have been obtained and their average, rough values rounded to one digit after the decimal separator (relative uncertainties of about ±20%), are given under the main arrow from Figure 15d. n corresponds to an aldehyde Ald(n) from the ordered set (Figure 15d), and n+1, to the immediately next one Ald(n+1), in the increasing sense of formation constants. If K_Ald(n)/Ald(n)**L5**_ is the formation constant associated to the equilibrium

$$\text{Ald}(\text{n})+\mathrm{L5}⇌\text{Im}(\text{n})+{\text{H}}_{2}\text{O}$$

then one has K_Ald(n+1)/Ald(n+1)**L5**_ = K_Ald(n)/Ald(n)**L5**_ × K(n,n+1).

One obtained (Figure 15d): K_2OMeal/2OMealL5_ ≈ 4.6K_oc4al/oc4alL5_, K_iphtal/monoim_ ≈ 8.6K_2OMeal/2OMealL5_, K_quinal/quinalL5_ ≈ 2.3K_iphtal/monoim_, K_diclal/diclalL5_ ≈ 1.1K_quinal/quinalL5_, K_bral/bralL5_ ≈ 1.6_Kdiclal/diclalL5_, K_dialpy/monoim_ ≈ 2.1K_bral/bralL5_, K_dinial/dinialL5_ ≈ 4.6K_dialpy/monoim_, K_dialpymph/monoim_ ≈ 2.4K_dinial/dinialL5_ and K_OHal/OHalL5_ ≈ 1.5K_dialpymph/monoim_. Of all formation constants, only K_oc4al/oc4alL5_ (for the reaction of aldehyde **oc4al** with amine **L5**) has been determined directly; it has a value of, roughly, about 52 (uncertainties of about, roughly, ±20%).

## 4 Solvent-free imine formation and exchanges

**FIGURE 16:**
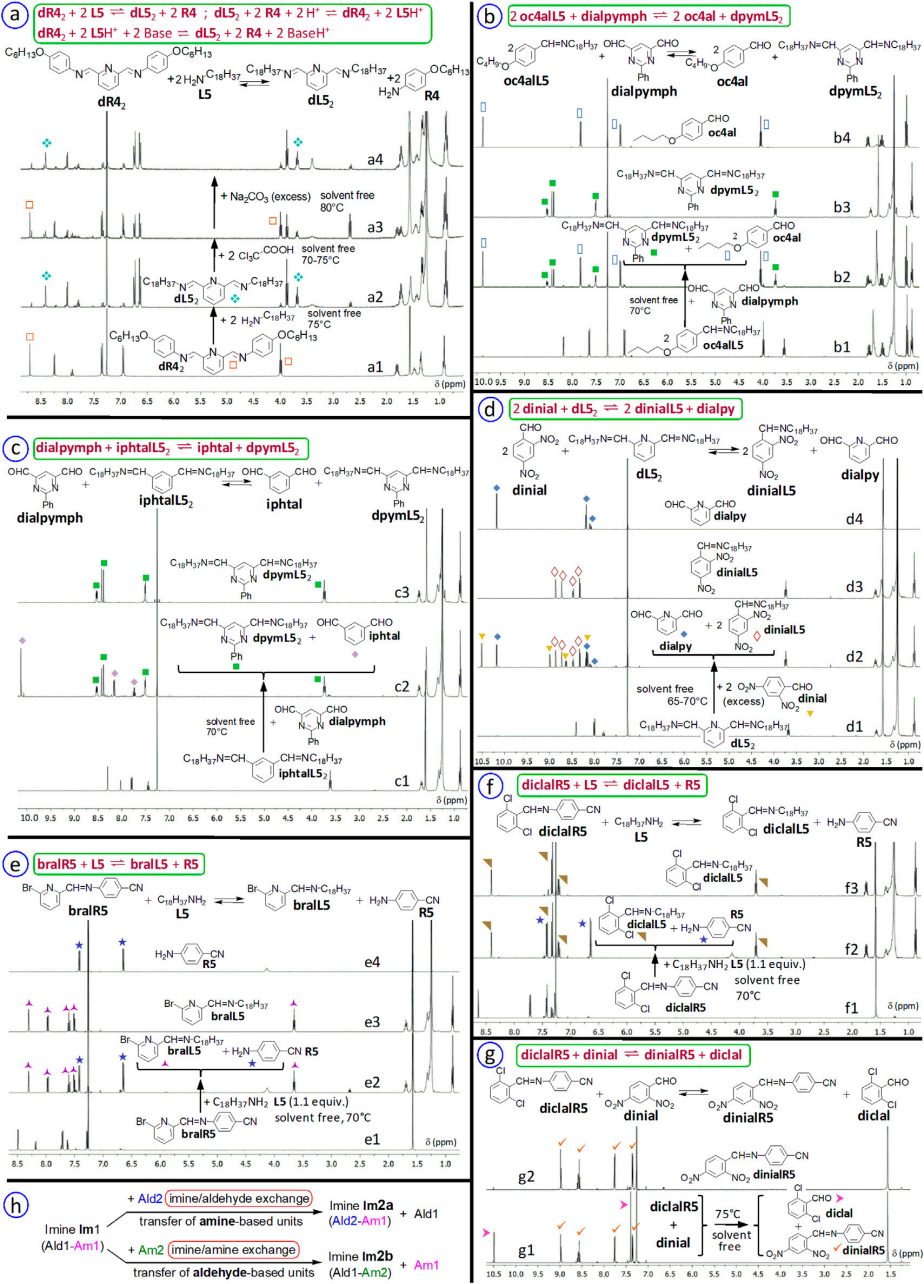
Solvent-free exchanges between (bis-)imines and (di)aldehydes or amines. ^1^H NMR spectra (400 MHz, CDCl_3_) of samples recorded a few minutes after their dissolution. **(a)** Reaction mixture of bis-imine **dR4**_2_ (obtained from 2,6-pyridinedicarboxaldehyde **dialpy** and 2 equiv. of 4-(hexyloxy)aniline **R4**, 24h, 70°C, >95%; a1) with 2 equiv. of octadecylamine **L5** (a2, 18h, 75°C, 60-74%), followed by addition of trichloroacetic acid TCA (a3; 2 equiv. of acid, 16h, 70°C–75°C, 33–49%) and of sodium carbonate (a4; 9 equiv. of carbonate, 18h, 80°C, 66%); **(b)** Reaction mixture of imine **oc4alL5** (from 4-butoxybenzaldehyde **oc4al** and 1 equiv. of octadecylamine **L5**, 18h, 75°C, >90%; b1) with 0.5 equiv. of 2-phenyl-4,6-pyrimidinedicarboxaldehyde **dialpymph** (b2; 18h, 70°C, ≈80%) compared with bis-imine **dpymL5**_2_ (from 2-phenyl-4,6-pyrimidinedicarboxaldehyde and 2 equiv. of octadecylamine, 18h, 70°C, >95%; b3) and with 4-butoxybenzaldehyde **oc4al** (b4); **(c)** Reaction mixture of bis-imine **iphtalL5**_2_ (from isophtalaldehyde and 2.05 equiv. of octadecylamine, 22 h, 80°C–85°C, >95%; c1) with 1 equiv. of 2-phenyl-4,6-pyrimidinedicarboxaldehyde (16h, 70°C, 65–85%; c2) compared with bis-imine **dpymL5**_2_ (c3); **(d)** Reaction mixture of the bis-imine **dL5**_2_ (from 2,6-pyridinedicarboxaldehyde and 2 equiv. of octadecylamine, 6h, 70°C, >95%; d1) with an excess of 2,4-dinitrobenzaldehyde **dinial** (4 equiv., 24 h, 65°C–70°C, >95%; d2) compared with the imine **dinialL5** (obtained from 2,4-dinitrobenzaldehyde and 1 equiv. of octadecylamine, 18 h, 70°C, >90%; d3) and with 2,6-pyridinedicarboxaldehyde **dialpy** (d4). ^1^H NMR spectra (500 MHz, CDCl_3_). **(e)** Reaction mixture of imine **bralR5** (from 4-aminobenzonitrile **R5** and 1 equiv. of 6-bromo-2-pyridinecarboxaldehyde **bral**, 24h, 85°C, >90%; aminal 4-8%; e1) with octadecylamine **L5** (1.1 equiv., 20h, 70°C, >90%; e2), compared with imine **bralL5** (obtained from 6-bromo-2-pyridinecarboxaldehyde **bral** and 1 equiv. of octadecylamine **L5**, 20h, 70°C, >95%; e3) and with 4-aminobenzonitrile **R5** (e4); **(f)** Reaction mixture of imine **diclalR5** (from 4-aminobenzonitrile **R5** and 1 equiv. of 2,6-dichlorobenzaldehyde **diclal**, 48h, 80°C, 80-90%; aminal 4-12%; f1) with octadecylamine **L5** (1.1 equiv., 20h, 70°C, >90%; f2), compared with imine **diclalL5** (obtained from 2,6-dichlorobenzaldehyde **diclal** with 1 equiv. of octadecylamine **L5**, 18h, 70°C, >90%; f3); **(g)** Reaction mixture of imine **diclalR5** with 2,4-dinitrobenzaldehyde **dinial** (1 equiv., 18h, 75°C, > 90%; g1), compared with imine **dinialR5** (from **dinial** and 1 equiv. of **R5**, 48h, 80°C, > 90%; g2); **(h)** Two possibilities of transimination and transfer of structural units illustrated for a monoimine: imine/amine and imine/aldehyde exchanges. All starting imines were synthesized without solvent.

**FIGURE 17:**
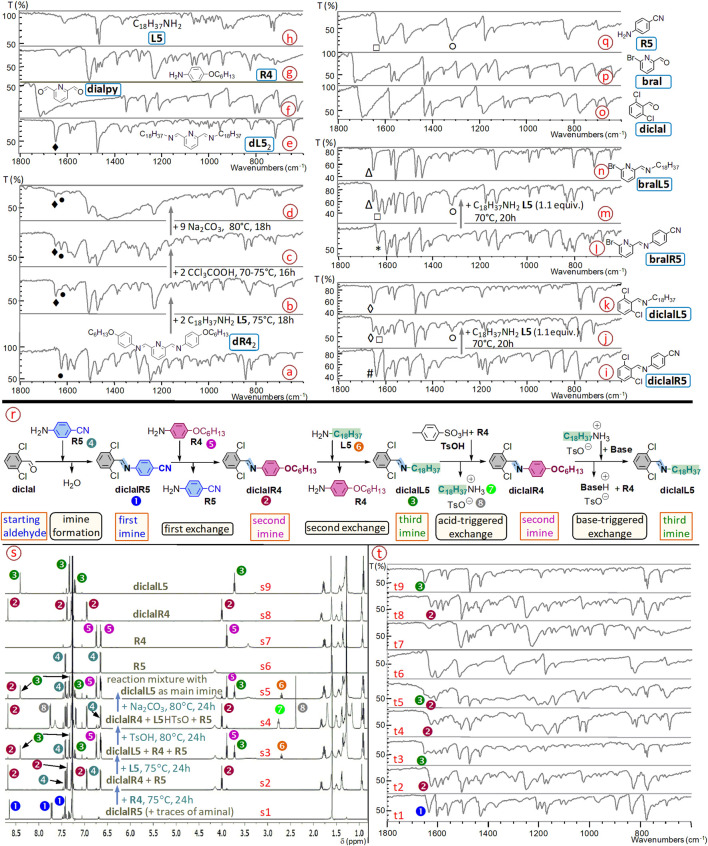
**(a–h)** Part of IR spectra (by Attenuated Total Reflectance = ATR) of the solvent-free reaction mixture of bis-imine **dR4**_2_ (from 2,6-pyridinedicarboxaldehyde **dialpy** and 4-(hexyloxy)aniline **R4**) **(a)** with 2 equiv. of octadecylamine **L5** **(b)**, then with acid (TCA; **(c)**) and base (Na_2_CO_3_; **(d)**), as well as those of starting materials **(f–h)** and of the bis-imine **dL5**_2_ from 2,6-pyridinedicarboxaldehyde and octadecylamine **(e)**. ♦ denotes stretching bands of C=N bond in aliphatic imines ν_C=Nalkyl_ (here, 1,647–1,648 cm^-1^) and •, those of the C=N bond in aromatic imines ν_C=Naryl_ (here, 1,623–1,626 cm^-1^). One may notice that in **(b)** the height of ♦ (ν_C=Nalkyl_) is greater than that of • (ν_C=Naryl_), while in **(c)** the height of • (ν_C=Naryl_) is greater than that of ♦ (ν_C=Nalkyl_); in **(d)** the height of ♦ (ν_C=Nalkyl_) is again greater than that of • (ν_C=Naryl_). **(i-k)** Part of IR spectra (by ATR) of the reaction mixture of imine **diclalR5** **(i)** with n-octadecylamine **L5** **(j)**, compared with that of imine **diclalL5** **(k)**; **(l-n)** Part of IR spectra (by ATR) of the reaction mixture of imine **bralR5 (l)** with n-octadecylamine **L5 (m)**, compared with that of imine **bralL5 (n)**; **(o-q)** Part of IR spectra (by ATR) of aldehyde **diclal (o)**, aldehyde **bral (p)** and 4-aminobenzonitrile **R5 (q)**. # and * denote imine stretching bands ν_C=Naryl_, ◊ and △ denote imine stretching bands ν_C=Nalkyl_. □ denotes the scissoring mode β_s_(NH_2_), and ○ denotes the stretching mode ν_C-NH2_ coupled with the bending δ(NH_2_) in 4-aminobenzonitrile (Palafox et al., 2006). **(r,s)** A multistep sequence based on imine/amine exchanges: scheme **(r)** represents the whole process illustrated through the ^1^H NMR spectra (500 MHz, CDCl_3_; **s)** of: the starting (first) imine **diclalR5** (s1), the mixture of the first exchange (s2), the mixture of the second exchange (s3), the mixture of the acid-triggered exchange **(s4)**, the mixture of the base-triggered exchange **(s5)**, the two aromatic amines **R5** (s6) and **R4** (s7), and imines **diclalR4** (s8) and **diclalL5** (s9). The numbers associated to peaks correspond to compounds from **(r)**. **(t)** t1 to t9 are IR spectra which correspond to NMR spectra s1 to s9. Only several key IR bands are denoted with numbers corresponding to compounds. T = transmittance.

**FIGURE 18:**
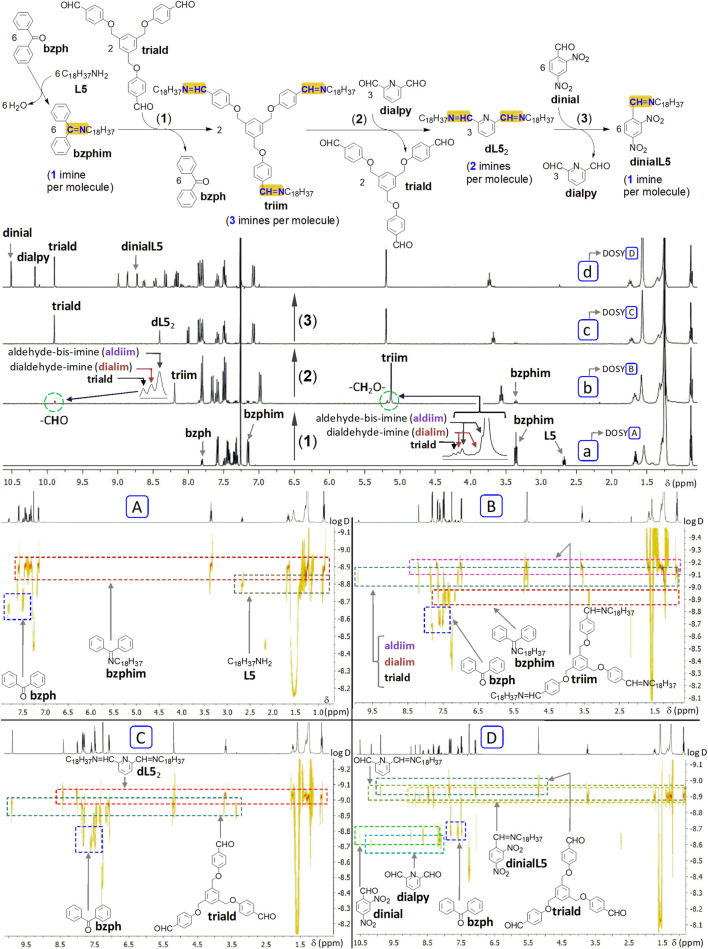
A sequential multistep process: solvent-free successive transiminations (top scheme). (a-d) ^1^H NMR spectra (400 MHz, CDCl_3_) of reaction mixtures, recorded a few minutes after dissolution of samples, and **(A–D)** the corresponding ^1^H DOSY NMR spectra (500 MHz, CDCl_3_): reaction of benzophenone **bzph** with octadecylamine **L5** (a, **A**), followed by addition (1) of trialdehyde **triald** (b, **B**), then (2) of 2,6-pyridinedicarboxaldehyde **dialpy** (c, **C**) and, in the final step (3), of 2,4-dinitrobenzaldehyde **dinial** (d, **D**). During this process, one passes from a monoimine (**bzphim**) to a tris-imine (**triim**), then to a bis-imine (**dL5**_2_), and, finally, again to a monoimine (**dinialL5**). For conditions and discussion, see the text. D is the diffusion coefficient, measured in m^2^s^−1^.

We performed several reactions of imine formation and exchange in the absence of solvents (Figures 16–18).

### 4.1 Overview and examples

Amongst the advantages of such solvent-free reactions are the saving of organic solvents and the reduction of organic wastes (in a green chemistry, sustainable approach), the saving of the energy and time required to evaporate the solvents, a possible simplification of the work-up. Absence of solvent also means higher concentrations of starting materials, absence of the water that would have been contained in the solvent and therefore, limitation of hydrolysis and shift of equilibriums. In addition, without solvent (particularly when both water and imine reaction product are soluble in it), a better separation of the organic phase from the water generated in the reaction is achieved.

Such reactions have already been performed in the field of imine chemistry (Alvarez-Santamaría et al., 2019; Crawford et al., 2015; Crawford et al., 2017; Ferguson et al., 2014; León et al., 2023; Leoni et al., 2019; Shaw et al., 2018; Shaw et al., 2019; Shaw et al., 2024; Singh et al., 2021; Suzuki et al., 2018; Suzuki et al., 2019; Wang et al., 2016; Zuo et al., 2022). Here, we applied them to imine synthesis, to (bis-)imine/amine and (bis-)imine/(di)aldehyde exchanges (Figures 16, 17), as well as, in a multistep process, to a sequence of successive transiminations involving mono-, bis- and tris-imines (Figure 18).

The solvent-free transiminations performed in one step, which are reported herein, are of following types (Figure 16):

- dialdehyde-derived bis-imine with amine, here, aromatic bis-imine **dR4**_2_ with 2 equiv. of aliphatic amine **L5** (Figures 16a, 17a, b):
$$\text{dR}4_{2}+2\;L5⇌\text{dL}5_{2}+2\;R4$$
- dialdehyde-derived bis-imine with dialdehyde, namely bis-imine **iphtalL5**_2_ with 1 equiv. of dialdehyde **dialpymph** (Figure 16c; for the corresponding DOSY spectrum, see SM, file synt-char, p. 126):
$$\text{dialpymph}+\text{iphtalL}5_{2}⇌\text{iphtal}+\text{dpymL}5_{2}$$
- dialdehyde-derived bis-imine with aldehyde, here, bis-imine **dL5**_2_ with 2 equiv. (in practice we used 4 equiv.) of 2,4-dinitrobenzaldehyde **dinial** as competing aldehyde (Figure 16d; for the corresponding DOSY spectrum, see SM, file synt-char, p. 125):
$$2\;\text{dinial}+\text{dL}5_{2}⇌2\;dinialL5+\text{dialpy}$$
- imine with dialdehyde, namely 2 equiv. of imine **oc4alL5** with 1 equiv. of **dialpymph** (Figure 16b; for the corresponding DOSY spectrum, see SM, file synt-char, p. 127):
$$2\;oc4alL5+\text{dialpymph }⇌\;2\;oc4al+\text{dpymL}5_{2}$$
- imine with amine, e. g. imines **bralR5** or **diclalR5** with 1 equiv. of octadecylamine **L5** (Figures 16e,f; 17i-q):
bralR5+L5⇌bralL5+R5
diclalR5+L5⇌diclalL5+R5
- imine with aldehyde, here, imine **diclalR5** with 1 equiv. of 2,4-dinitrobenzaldehyde **dinial** (Figure 16g):
diclalR5+dinial⇌dinialR5+diclal

The starting materials were roughly ground. Solvent-free reactions, usually on 2–20 mg of starting aldehyde or imine, were done in small glass vials with good caps.

The ^1^H NMR spectra of samples of solvent-free reaction mixtures were recorded a few (generally, between 2 and 5) minutes after the dissolution of the samples. It is assumed (except for acid-triggered exchanges) that, during this short time and in the absence of (acid) catalysts, the transiminations do not (significantly) progress in solution and that, in this way, the composition of the mixture remains almost unchanged between the solvent-free sample and the corresponding solution in CDCl_3_.

Two of the amines used in solvent-free syntheses and exchanges are 4-(hexyloxy)aniline **R4** and octadecylamine **L5**, which are already molten under the reaction conditions (65°C-95°C; reaction time, depending on the case, between 6 h and 8 days; vide infra section 4.3.1 and Figure 16), a fact that improves the homogenization in the course of the reaction. The reaction times are, usually, not optimized. The yields were determined by ^1^H NMR. Small amounts of compounds like 2,6-dichlorobenzaldehyde, may deposit, during the reaction, on the wall of the vial. The yields of syntheses of starting (bis-)imines **dR4**_**2**_, **dL5**_**2**_, **iphtalL5**_**2**_ and **oc4alL5** can be seen as very good (>90%). Most of the exchanges we tested (e.g. Figure 16 a2, b2, c2) worked rather satisfactorily, with yields between 70% and 85%. An appropriate excess of competing reagent, for example, aldehyde **dinial** (Figure 16 d2), may be used to improve the yield.

#### 4.1.1 IR spectroscopy as an analytical tool for solvent-free reactions

The solvent-free final reaction mixtures were also investigated through infrared (IR) spectroscopy (Figures 17a–q, t), that can provide useful structural information for characterization of such mixtures. The IR data are in agreement with the NMR ones (vide infra, Sections 4.2 and 4.4).

For example, the stretching bands ν_C__=Naryl_ observed in imines **diclalR5** (1,636 cm^−1^; Figure 17i) and **bralR5** (1,629 cm^−1^; Figure 17l) are replaced, upon reaction with n-octadecylamine **L5**, by bands with higher wavenumbers—the stretching bands ν_C=Nalkyl_—in **diclalL5** (1,648–1,651 cm^−1^; Figures 17j, k) and **bralL5** (1,651 cm^−1^; Figure 17m, n). In addition to these changes, these imine/amine exchanges without solvent are also confirmed through the formation of amine **R5**, which is suggested, amongst other bands, by the scissoring mode of the NH_2_ group (β_s_) at about 1,621–1,625 cm^−1^, as well as by the stretching mode ν_C-NH2_ coupled with the bending δ(NH_2_), at about 1,315–1,317 cm^−1^. For the attributions of the IR bands in amine **R5**, see (Palafox et al., 2006).

#### 4.1.2 Two kinds of exchanges per imine group

We are mentioning here only imine/amine and imine/aldehyde exchanges, namely exchanges between imines and compounds possessing one (i.e., here, amine or aldehyde) of the functional groups which react together to form imines (and water). Indeed, an imine group (Figure 16h) offers the possibility of imine/amine exchanges associated to a transfer of aldehyde-derived units, as well as the possibility of imine/aldehyde exchanges associated to a transfer of amine-derived units. In the context of the work reported herein, both possibilities are illustrated, under solvent-free conditions, for imine **diclalR5**: the imine/amine exchange upon reaction with amine **L5** (Figure 16f), and the imine/aldehyde exchange upon reaction with aldehyde **dinial** (Figure 16g).

For pyridine-based, dialdehyde-derived bis-imines, were performed not only solvent-free bis-imine/amine exchanges (bis-imine **dR4**_2_ with amine **L5**; Figure 16, spectra a1,a2), but also solvent-free bis-imine/aldehyde exchanges (bis-imine **dL5**_2_ with aldehyde **dinial**; Figure 16, spectra d1,d2).

### 4.2 Acid/base-modulated covalent switches

For the solvent-free acid/base-triggered switches between bis-imines **dR4**_**2**_ and **dL5**_**2**_ (Figure 16 a3, a4)

$$\text{dL}5_{2}+2\;R4+2\;H^{+}⇌\text{dR}4_{2}+2\;L5H^{+}$$

$$\text{dR}4_{2}+2\;L5H^{+}+2\text{ Base}⇌\text{dL}5_{2}+2\;R4+2\;{\text{BaseH}}^{+}$$

we used trichloroacetic acid (TCA) and sodium carbonate (both being solid at room temperature), as acidic and basic triggers, respectively. TCA (CCl_3_COOH) was chosen here because it is less volatile than TFA (CF_3_COOH). The yields are lower than those of the corresponding solvent-free reactions without triggers (Figures 16a1, a2), and a possible optimization would be a better homogenization of the solvent-free solid reaction mixtures. These preliminary results will be further investigated.

The switches between bis-imines **dR4**_2_ and **dL5**_2_ (Figure 16a) were also followed by IR (infrared) spectroscopy on solvent-free, solid samples (Figure 17), thanks to, for example, the stretching bands of the imine C=N bond which are, here, at 1,623–1,626 cm^-1^ (ν_C=Naryl_, aromatic imine) and 1,647–1,648 cm^-1^ (ν_C=Nalkyl_, aliphatic imine). See also SM, file synt-char, p. 128. Here, the starting bis-imine is **dR4**_2_ (Figure 17a). In the IR spectrum of the mixture obtained after the first transimination (Figure 17b), the two stretching bands ν_C=Naryl_ and ν_C=Nalkyl_ have different intensities, which move - after each subsequent transimination step - in opposite directions (Figures 17c,d). IR spectra suggest, together with the NMR results, that the switch globally operates as expected. The IR spectra corresponding to the steps of the switch (Figures 17a–d), can be compared with that of bis-imine **dL5**_2_ (Figure 17e) and with those of starting reagents (Figures 17f–h).

### 4.3 A multistep sequence of transiminations based on imine/aldehyde exchanges

The series of successive transiminations previously presented (vide supra, Sections 2.4, 2.5) were performed in a switch-like manner, between two (bis-)imines (Im1→Im2→Im1→Im2→Im1 …), and they were modulated by additions of acid and base. The example presented hereafter shows that successive transiminations can also be implemented as a multistep process, namely from an imine to a second one, then to a third one, and, in the final step, to a fourth one, in just one direction (Im1→Im2→Im3→Im4), without modulation through stimuli (pH changes). The solvent-free multistep sequence presented hereafter was designed on the basis of the order from Figure 15. For another example of multistep process in imine chemistry (but performed in solution), see (Schultz and Nitschke, 2006).

#### 4.3.1 Operation

Solvent-free reaction of benzophenone **bzph** with octadecylamine **L5** (1 equiv., 5 days, 75–80°C) produces the corresponding ketimine N-octadecyl-1,1-diphenylmethanimine **bzphim** (72%–82%) (Figure 18A a). This reaction mixture, used without purification, was treated with trialdehyde **triald** (for its synthesis, see (Rajakumar et al., 2006); 1/3 equiv. of **triald** with respect to the total initial amount of amine **L5**; 6–8 days, 80 °C–90 °C) and produced the tris-imine **triim** (yield of 66%–80% with respect to the total initial amount of **L5**; Figure 18B b). Intermediate aldehyde-bis-imine and dialdehyde-imine are also present. Treatment of this mixture with dialdehyde **dialpy** (1/2 equiv. with respect to the total amount of **L5**; 1–1.5 days, 70 °C–75 °C) produces the expected bis-imine **dL5**_2_ (about 80–90%; Figure 18C c). This mixture was reacted with 2,4-dinitrobenzaldehyde **dinial** (4 equiv., so an excess of 2 equiv. with respect to **L5**, 1–1.5 days, 65 °C–70 °C) to produce the corresponding imine **dinialL5** (77%–85% with respect to **L5**; Figure 18D d), together with observable traces of the intermediate aldehyde-imine (monoimine). Reagents like benzophenone and dinitrobenzaldehyde may partly deposit on the vial wall. The reactions were followed by 1D ^1^H NMR (recorded a few minutes after the dissolution of the samples) and by ^1^H DOSY NMR (Figure 18); chemical exchanges in solution may affect the values of the diffusion coefficients.

#### 4.3.2 Change of the number of imine groups per molecule

In this sequence of successive transiminations (although not with quantitative yields), the amine-derived unit is transferred from an imine-containing species to another. The multistep process consists of three subsequent solvent-free reactions done in the same pot. Correlatively, the number of imine groups per imine-containing species changes from a step to another (Figure 18), namely from 1 (**bzphim**) to 3 (**triim**), then to 2 (**dL5**_2_) and, finally, again to 1 (**dinialL5**). Vide supra, Section 3.1.

### 4.4 A multistep sequence of transiminations based on imine/amine exchanges

In this solvent-free sequence, the **R5**-derived unit from imine **diclalR5** is replaced by a unit derived from 4-(hexyloxy)aniline **R4**. Further, the **R4**-derived unit from **diclalR4** is replaced by that derived from n-octadecylamine **L5**, when forms imine **diclalL5** (Figure 17r). In this way takes place a 2-step transfer of the unit derived from aldehyde **diclal**.

Practically, solvent-free reaction of the first monoimine **diclalR5** of the sequence (obtained from **diclal** and **R5**, without solvent; 48 h, 80°C, 80-90%; Figure 17sl) with 1.1 equiv. of 4-(hexyloxy)aniline **R4** (24 h, 75°C, Figure 17s2) generates the second imine **diclalR4** (> 90%). Reaction of the mixture obtained in this way, with 1.25 equiv. of n-octadecylamine **L5** (24 h, 75°C, Figure 17s3) produces **diclalL5**, the third imine of the sequence (92%), with about 8% of imine **diclalR4**. Comparison with spectra of amines **R4** and **R5** (Figures 17s4,s5) and with those of imines **diclalR4** (from 2,6-dichlorobenzaldehyde **diclal** and amine **R4**, without solvent; 24 h, 75°C, > 90%; Figure 17s6) and **diclalL5** (from **diclal** with 1 equiv. of **L5**, without solvent; 18 h, 70°C, > 90%; Figure 17s7), confirms the formation of expected imines and amines.

Addition of p-toluenesulfonic acid monohydrate (TsOH·H_2_O; 1.25 equiv.) to this reaction mixture (80 °C, 24 h), leads to compound **diclalR4** as main imine (92%; acid-triggered exchange; Figure 17 r,s4). Subsequent treatment with an excess of Na_2_CO_3_ (5 equiv.) under similar conditions, generates **diclalL5** as main imine (84%; base-triggered exchange; Figure 17r,s5).

The whole process is one of type Im1→Im2→Im3→Im2→Im3, namely a combined (mixed) one consisting of a multistep sequence of exchanges without stimuli (Im1→Im2→Im3) and a stimuli-triggered switch (Im3→Im2→Im3).

One may wish to reuse, in new acid-base cycles, the imines and amines from the final solvent-free reaction mixture. To this end, they can be extracted in accordance with the following steps, thanks to the low solubility of NaTsO and Na_2_CO_3_ in chloroform: addition of chloroform to the final mixture, stirring, centrifugation, filtration, removal of the solvent.

The sequence was monitored by ^1^H NMR (the spectra being recorded 2-5 min after the dissolution of samples; Figures 17s1–s9) and IR spectroscopy (Figures 17t1–t9).

In all intermediate steps of sequences presented in Sections 4.2–4.4, the reagents are added as ground solids, without solvents. Before heating, the solid reaction mixtures are ground again with the added reagents.

## 5 Mathematical models

This part is focused on calculations with and simulations of equilibrium constants in solution, for the reaction of a dialdehyde with a monoamine, followed by the addition of a second amine.

In this way, these calculations deal with the formation and exchange of dialdehyde-based bis-imines and they are intended to complement the experimental work. One can, for example:

- Estimate the composition at equilibrium starting from initial concentrations of reagents and from the corresponding equilibrium constants. See, for example, (Crerar, 1975) and references cited herein;
- Calculate the excess of a reagent required to reach a desired yield;
- Calculate concentration-versus-pH distribution curves, which represent the composition of an equilibrated reaction mixture as a function of the pH, and are relevant for pH-adaptive dynamic libraries;
- Estimate/simulate the values of equilibrium constants starting from desired yields. From such constants, one can estimate (but this was not done here) the corresponding Gibbs free energies (ΔG = -RTlnK), which could further be used in the framework of structure-free energy relationships (Liu et al., 1994). One notices also that, on the basis of quantitative determination of relationships between the rate and equilibrium constants, in the framework of structure-reactivity relationships for Schiff base formation, was developed a model to predict the macroscopic behavior of dynamic covalent materials (hydrogels) (Morgan et al., 2024);
- Quantitatively investigate the effect of the amount of water on the concentration of imine-containing compounds;
- Generalize or extend such calculations to other classes of chemical reactions similar to those discussed herein (i.e. reaction of a difunctional compound with a complementary monofunctional one, followed by an exchange with a second, competing, monofunctional compound).

Key points would be: the quantitative description of reaction mixtures at equilibrium, the solving of the corresponding systems of (polynomial) equations and the possible simplifications of the mathematical treatment.

For the sake of simplification, we assumed that, in this mathematical model, molar concentrations are equal to activities, and that the volume of solution does not significantly change on addition of various reagents. Hydration of aldehydes, formation of (hemi)aminals, protonation of Nsp^2^ atom of pyridines or other possible protonation equilibriums (except the protonation of NH_2_ groups of amines B and C), dissociation of water, and other reactions, except those written herein, were not taken into account for the calculations.

One deals here with an (aromatic) dialehyde A, that reacts with a first primary (aromatic) amine B. To this reaction mixture is added a second primary amine C, then acid. A, B and C denote also, in calculations, parameters.

For simplification, all kinds of hydrated or solvated protons are denoted H^+^.

The meaning of the term “yield” is the ratio between the number of moles of (aldehyde- or bis-)imine of interest, and the number of moles of dialdehyde A present initially, i.e., for a derivative of A, the molar fraction of A that generated that derivative.

For the calculations, the only notations which keep their meaning from a subsection to another one, are those for the initial concentrations of dialdehyde A (a), amine B (b), amine C (c) and water (w) and those for the following concentrations at equilibrium: x, y, z, t, u, v, s and h. The other notations may change their meanings from a subsection to another. In such cases, they are explicitly associated to their respective new formulae or meanings.

One assumes that the reactions between aldehydes and amines take place in the sense of formation of imines, so that x, y, z, t, u, v and s should be positive. The multiplication sign was omitted in the literal expressions.

### 5.1 Formation of the first bis-imine

Dialdehyde A (that may be, but is not only the dialdehyde **A**), of initial molar concentration in the reaction mixture a, reacts with the primary (aromatic) monoamine B (of concentration b) to form, as a final target product, the bis-imine AB_2_. The equilibrium constants being K_1_ and K_2_, the reactions are:

$$A+B⇌\text{AB}+H_{2}O\;K_{1}=x\left(w+x+2y\right)/\left[\left(a-x-y\right)\left(b-x-2y\right)\right]$$

$$\text{AB}+B⇌{\text{AB}}_{2}+H_{2}O\;K_{2}=y\left(w+x+2y\right)/\left[x\left(b-x-2y\right)\right]$$

where the initial concentrations (prior to formation of any imine) are [A]_in_ = a, [B]_in_ = b, [AB]_in_ = 0, [AB_2_]_in_ = 0, [H_2_O]_in_ = w, and the concentrations at equilibrium are [A]_eq_ = a-x-y, [B]_eq_ = b-x-2y, [AB]_eq_ = x, [AB_2_]_eq_ = y, [H_2_O]_eq_ = w + x + 2y. The concentrations x and y at equilibrium, the excess of B required to obtain a desired concentration of AB_2_, and equilibrium constants from yields, can be calculated as follows.

#### 5.1.1 Calculation of K_1_ and K_2_ from yields

If the ratios (yields at equilibrium) [AB]_eq_/[A]_in_ = x/a = m and [AB_2_]_eq_/[A]_in_ = y/a = n are known, then we have x = ma, y = na and, finally, K_1_ = [m/(1-m-n)]{[w/a + (m + 2n)]/[b/a-(m + 2n)]}, K_2_ = (n/m){[w/a + (m + 2n)]/[b/a-(m + 2n)]} and K_e1_ = K_1_K_2_ = [n/(1-m-n)]{[w/a + (m + 2n)]/[b/a-(m + 2n)]}^2^. See SM, file l-simul-K1-K2.

#### 5.1.2 Composition at equilibrium

If the initial concentrations (before formation of any imine) [A]_in_ = a, [B]_in_ = b, [AB]_in_ = [AB_2_]_in_ = 0, [H_2_O]_in_ = w, and K_1_ and K_2_ are known, one may wish to calculate the concentrations of species at equilibrium. One needs x and y. From K_1_/K_2_ = x^2^/[y (a-x-y)] one obtains the equation in x, K_2_x^2^ + K_1_yx + K_1_ (y-a)y = 0, with the discriminant Δ = K_1_(K_1_-4K_2_)y^2^ + 4K_1_K_2_ay and x_1,2_ = (-K_1_y±Δ^1/2^)/(2K_2_). One notices that -K_1_y < 0 and -(Δ^1/2^) < 0, and so (-K_1_y-Δ^1/2^)/(2K_2_) < 0. The positive solution is x = (Δ^1/2^-K_1_y)/(2K_2_) > 0. This expression is introduced into K_2_ and gives the equation (Δ^1/2^-K_1_y)[-Δ^1/2^-(4K_2_-K_1_)y + 2K_2_b] = 2(4K_2_-K_1_)y^2^ + 4K_2_wy + 2yΔ^1/2^, which, after rewriting and simplification, becomes Ay^2^ + By = (Dy + E) (Fy^2^ + Gy)^1/2^, where A = K_1_(4K_2_-K_1_ + 1)-4K_2_ = (4K_2_-K_1_) (K_1_-1), B = -K_2_ [K_1_ (2a + b) + 2w], D = 2K_2_-K_1_ + 1, E = -K_2_b, F = K_1_(K_1_-4K_2_) and G = 4K_1_K_2_a. After elimination of radicals, simplification and rewriting, one obtains the cubic (third degree) equation αy^3^ + βy^2^ + γy + δ = 0, that can be solved analytically (Fettis, 1942; Zucker, 2008; vide infra) and where α = A^2^-D^2^F, β = 2AB-2DEF-D^2^G, γ = B^2^-E^2^F-2DEG, δ = -E^2^G. Example of verification key (for 1 real root): for a = 5, b = 8, w = 2, K_1_ = 0.6, K_2_ = 2.25, one obtains x = 1, y = 1.5. See SM, files g-S1-Num-expl, g-S1-St1-comp-after-add-B, g-S1-St3-comp-after-add-exc-B, v-key-ABW, v-verif-ABW.

Sections 5.1.2, 5.1.3 are, here, treated independently of each other.

#### 5.1.3 Calculation of the supplementary amount (excess) of amine B

Let [A]_in_ = a, [B]_in_ = b, [AB]_in_ = 0, [AB_2_]_in_ = 0, [H_2_O]_in_ = w be the initial concentrations of reagents for which the equilibrium is reached at the ratios m = [AB]_eq_/a and n = [AB_2_]_eq_/a. In this case, K_1_ and K_2_ can be calculated as done before. We now wish to know which is the supplementary amount (the excess) of B, which is to be added to the mixture at equilibrium, so that the yield of AB_2_ increases from s to p, which means [AB_2_]_eq_/a = p (where p > n). Let this excess of B be, in terms of concentration, g_B_ = [B]_ex_. With [AB_2_]_eq_ = pa, [AB]_eq_ = x, [A]_eq_ = a-x-pa, [B]_eq_ = b-x-2pa + g_B_, and [H_2_O]_eq_ = w + x + 2pa, we have K_1_ = x (w + x + 2pa)/[(a-x-pa) (b-x-2pa + g_B_)] and K_2_ = pa (w + x + 2pa)/[x (b-x-2pa + g_B_)]. We obtain K_1_/K_2_ = x^2^/{pa [(1-p)a-x]}, which leads to the quadratic equation x^2^ + pa (K_1_/K_2_)x - p (1-p) (K_1_/K_2_)a^2^ = 0, with the discriminant Δ = p^2^a^2^(K_1_/K_2_)^2^{1 + 4 [(1-p)/p](K_2_/K_1_)} = p^2^a^2^(K_1_/K_2_)^2^ [1 + 4 (1/p-1) (K_2_/K_1_)] and x_1,2_ = a [(p/2) (K_1_/K_2_)]{±[1 + 4 (1/p-1) (K_2_/K_1_)]^1/2^–1}. The positive value of x being to be chosen, we have x = a [(p/2) (K_1_/K_2_)]{[1 + 4 (1/p-1) (K_2_/K_1_)]^1/2^–1} = a [(p/2) (K_1_/K_2_)]{[1 + 4 (1-p)K_2_/(K_1_p)]^1/2^–1}.

Then, from K_2_ we have g_B_ = [pa (w + x + 2pa)/(K_2_x)]-(b-x-2pa) or from K_1_, g_B_ = {x (w + x + 2pa)/[K_1_ (a-pa-x)]}-(b-x-2pa), which gives g_B_/a equivalents of B with respect to A or g_B_/b equivalents of B with respect to B. See SM, files g-S1-Num-expl, g-S1-St2-calc-exc-B.

The expression of the excess of B is not included in the calculations hereafter.

#### 5.1.4 Influence of the amount of water

Depending on the values of imine formation constants, the amount of water may, more or less considerably, affect the composition at equilibrium (vide infra, Section 5.2.4).

### 5.2 Exchange with monoamine. General approach

On addition of primary (aliphatic) monoamine C, that should be - if good transimination yields are wished - more nucleophilic than B, the following exchanges are expected to occur, the target compound being the bis-imine AC_2_: AB_2_ + C ⇌ ABC + B and ABC + C ⇌ AC_2_ + B. If one does not consider the equations of formation of aldehyde hydrates, aminals or hemiaminals, the system can be described through the following equations:

$$A+B⇌\text{AB}+H_{2}O\;K_{1}=x\left(w+x+2y+z+2t+2u\right)/\left[\left(a-x-y-z-t-u\right)\left(b-x-2y-u\right)\right]$$

$$\text{AB}+B⇌{\text{AB}}_{2}+H_{2}O\;K_{2}=y\left(w+x+2y+z+2t+2u\right)/\left[x\left(b-x-2y-u\right)\right]$$

$$A+C⇌\text{AC}+H_{2}O\;K_{3}=z\left(w+x+2y+z+2t+2u\right)/\left[\left(a-x-y-z-t-u\right)\left(c-z-2t-u\right)\right]$$

$$\text{AC}+C⇌{\text{AC}}_{2}+H_{2}O\;K_{4}=t\left(w+x+2y+z+2t+2u\right)/\left[z\left(c-z-2t-u\right)\right]$$

$$\text{AB}+C⇌\text{ABC}+H_{2}O\;K_{5}=u\left(w+x+2y+z+2t+2u\right)/\left[x\left(c-z-2t-u\right)\right]$$

where the following was added or changed in the concentrations from the introductory part of Section 5.1: [C]_in_ = c, [AC]_in_ = [AC_2_]_in_ = [ABC]_in_ = 0, [C]_eq_ = c-z-2t-u, [AC]_eq_ = z, [AC_2_]_eq_ = t, [ABC]_eq_ = u, [A]_eq_ = a-x-y-z-t-u and [B]_eq_ = b-x-2y-u.

The initial concentrations of compounds A, B, C and H_2_O are their total molar concentrations prior to the formation of any imine. In this framework, the initial concentrations of compounds AB, AB_2_, AC, AC_2_ and ABC are 0. The relation between initial concentrations and concentrations before reaction can be exemplified as follows. If a solution containing A, AB_2_, AB, ABC and B is mixed with a solution containing AC_2_, AC and C, so that the molar concentrations in the reaction mixture before reaction are [A]_bfr_, [AB]_bfr_, [AB_2_]_bfr_, [B]_bfr_, [AC]_bfr_, [ABC]_bfr_, [AC_2_]_bfr_, [C]_bfr_ and [H_2_O]_bfr_, then the initial, total concentrations prior to the formation of any imine, are [A]_in_ = [A]_bfr_ + [AB]_bfr_ + [AB_2_]_bfr_ + [AC]_bfr_ + [ABC]_bfr_ + [AC_2_]_bfr_, [B]_in_ = [B]_bfr_ + [AB]_bfr_ + 2 [AB_2_]_bfr_ + [ABC]_bfr_, [C]_in_ = [C]_bfr_ + [AC]_bfr_ + 2 [AC_2_]_bfr_ + [ABC]_bfr_ and [H_2_O]_in_ = [H_2_O]_bfr_ - ([AB]_bfr_ + [AC]_bfr_) - 2 ([AB_2_]_bfr_ + [ABC]_bfr_ + [AC_2_]_bfr_). In a shortened numerical example, we consider a mixture consisting of 1 equiv. of imine AB_2_ of molar concentration n, 0.005 equiv. of AB, 0.6 equiv. of B and 0.4 equiv. of water, that reacts with 1.4 equiv. of amine C. In this case, the concentrations (in mole/L) before reaction are [AB_2_]_bfr_ = n, [AB]_bfr_ = 0.005n, [B]_bfr_ = 0.6n, [H_2_O]_bfr_ = 0.4n and [C]_bfr_ = 1.4n, and the initial concentrations (before formation of any imine) are [A]_in_ = 1.005n, [B]_in_ = 2n + 0.005n + 0.6n = 2.605n, [C]_in_ = 1.4n and [H_2_O]_in_ = 0.4n-2n-0.005n = −1.595n.

[H_2_O]_in_ is generally positive or zero. [H_2_O]_in_ < 0 is a mathematical way to indicate that a part of the amount of water that forms in the reaction, starting from the initial concentrations, leaves the system during the equilibration. [H_2_O]_eq_ should however be positive. See SM, files g-S1-A1B2_75C4_9-W-1, g-S1-A1B2_75C4_9-W-1_9. Typically, [H_2_O]_in_ may be negative when one starts from compound AB_2_ (beforehand synthesized and isolated), that is treated with compound C in a solution where the concentration of water before reaction is less than twice the concentration of AB_2_ before reaction [H_2_O]_bfr_ < 2 [AB_2_]_bfr_.

One does not need to consider the equilibrium AC + B ⇌ ABC + H_2_O in addition to the above listed equations, because it and its constant (K_1_K_5_/K_3_) can be deduced from the equilibriums AB + C ⇌ ABC + H_2_O (K_5_), AC + H_2_O ⇌ A + C (K_3_) and A + B ⇌ AB + H_2_O (K_1_).

These calculations can be applied to any couple of two amines which react with a dialdehyde, as well as to any exchange between a bis-imine and an amine, regardless of the aliphatic or aromatic nature of the two amines.

#### 5.2.1 Simulation of constants K_3_, K_4_ and K_5_

In this section, the constants K_1_ and K_2_ are considered to be known. One may wish to estimate the values of constants K_3_, K_4_ and K_5_ that produce desired or known yields at equilibrium for the initial concentrations a, b, c and w. One assumes that the yields (and the concentrations) of three of the products AB, AB_2_, AC, AC_2_ and ABC are known (or desired) at equilibrium and have a numerical value. There are three main situations which depend on the groups {x, y} or {z, t, u} to which may belong the two remaining unknown concentrations.

- The unknown concentrations are x and y (1 possibility). Let the known (or desired) yields be n = t/a, m = z/a and o = u/a. We have K_1_/K_2_ = x^2^/{y [a (1-m-n-o)-x-y]} which gives x_1,2_ = (±Δ^1/2^-K_1_y)/2K_2_, with Δ = Dy^2^ + Ey, D = K_1_(K_1_-4K_2_) and E = 4K_1_K_2_(a-z-t-u) = 4aK_1_K_2_(1-m-n-o). The positive value of x = (Δ^1/2^-K_1_y)/2K_2_ is introduced into the expression of K_2_. After rewriting and simplification, one gets the equation Ay^2^ + By = (Fy + G) (Dy^2^ + Ey)^1/2^, with A = 4K_2_ + K_1_(K_1_-4K_2_–1), B = K_2_{K_1_ [2 (a-z-t-u) + b-u] + 2 (w + z + 2t + 2u)} = K_2_{K_1_ [2a (1-m-n-o) + b-ao] + 2 [w + a (m + 2n + 2o)]}, F = K_1_-2K_2_–1, G = K_2_ (b-u) = K_2_ (b-ao), a-z-t-u = a (1-m-n-o) and z + 2t + 2u = a (m + 2n + 2o). Elimination of radicals and simplification lead to the cubic equation αy^3^ + βy^2^ + γy + δ = 0, where α = A^2^-DF^2^, β = 2AB-2DFG-EF^2^, γ = B^2^-DG^2^-2EFG and δ = -EG^2^;
- One unknown concentration from {x, y} and one from {z, t, u} (6 possibilities). Let the known (desired) yields be, for example, r = y/a, n = t/a and o = u/a. The following notations are introduced J = w + 2 (y + t + u), K = a-(y + t + u) and L = b-(2y + u). From K_2_, we obtain z = -(K_2_/y)x^2^ + (K_2_L/y-1)x-J. z is introduced into the expression of K_1_ and leads to the cubic equation αx^3^ + βx^2^ + γx + δ = 0, where α = [K_2_ (1-K_1_)]/y, β = [K_2_L (2K_1_-1)]/y, γ = -K_1_ (J + K + K_2_L^2^/y) and δ = K_1_L (J + K);
- Both unknown concentrations from {z, t, u} (3 possibilities). Let the known (desired) yields be, for example, q = x/a, r = y/a and m = z/a, along with the notations M = w + x + 2y + z, N = a-(x + y + z) and O = b-(x + 2y). From K_1_/K_2_ we obtain t + u = N-K_2_x^2^/(K_1_y) = P. From K_2_, we have u = O-y (M + 2P)/(K_2_x), then we obtain t = P-u.

With the known concentrations and the appropriate roots determined as shown in paragraphs i)-iii), one can calculate the expected values of K_3_, K_4_ and K_5_. See SM, file l-simul-K3-K4-K5.

#### 5.2.2 Composition at equilibrium

For constants K_1_ to K_5_ supposed to be known and for initial concentrations in the reaction mixture before formation of imines, a, b, c and w, one wishes to know the concentrations of the species in the equilibrated reaction mixture.

1. We could not find the analytical solution of the system consisting of the equations from K_1_ to K_5_ with the unknowns x, y, z, t and u, and we solved it numerically for t = ar, where 0 < r < 1. We proceed as above and from K_4_/K_5_, we have x = Gzu, where G = K_4_/(K_5_t). We also have K_1_K_4_/(K_2_K_3_) = (t/y) (x/z)^2^ and K_4_/(K_5_t) = (1/u) (x/z), which leads to y = Fu^2^, where F = K_2_K_3_K_4_/(tK_1_K_5_^2^). From K_1_/K_3_ = {Gu [(c-2t)-(z + u)]}/{z [b-u (Gz-1-2Fu)]}, we obtain the equation Hu^2^ + Iu + J = Kuz, where H = K_3_G-2K_1_F, I = -[K_1_ + GK_3_ (c-2t)], J = K_1_b and K = G (K_1_-K_3_), which gives z = (Hu^2^ + Iu + J)/(Ku) and, further, x = Gzu = G (Hu^2^ + Iu + J)/K. We can calculate the following expressions in t and u: a-x-y-z-t-u = a-Gzu-Fu^2^-z-t-u = {-(FK + GH)u^3^-(GI + H + K)u^2^ + [K (a-t)-GJ-I]u-J}/(Ku) and z^2^ = [H^2^u^4^ + 2HIu^3^ + (I^2^ + 2HJ)u^2^ + 2IJu + J^2^]/(K^2^u^2^), which can be introduced into K_3_/K_4_ = z^2^/[t (a-x-y-z-t-u)] and lead, after rewriting, to the quartic (fourth degree) equation αu^4^ + βu^3^ + γu^2^ + δu + ε = 0, where α = -K_4_H^2^-tK_3_K(FK + GH), β = -tK_3_K(GI + H + K)-2K_4_HI, γ = tK_3_K [K (a-t)-GJ-I]-K_4_(I^2^ + 2HJ), δ = -J (2K_4_I + tK_3_K) and ε = -K_4_J^2^. Quartic equations can be solved analytically (Yacoub and Fraidenraich, 2012). Each of the roots of this quartic equation is introduced into the expressions of x, y and z (in Excel). The expressions of x, y, z and u (which depend on t = ar) are now introduced into the equation obtained from K_1_, namely K_1_ [(a-x-y-z-t-u) (b-x-2y-u)]-x (w + x + 2y + z + 2t + 2u) = 0. This equation can be solved numerically (in Excel), for r between 0 and 1. A shortened example of verification key (for 2 real roots): for a = 8, b = 18, c = 32, w = 4, K_1_ = 0.2, K_2_ = 2, K_3_ = 1.2, K_4_ = 1.875, K_5_ = 3, r = t/a = 0.5, one obtains, amongst 2 real solutions, the following one: x = 0.2, y = 0.4, z = 1.6, t = 4, u = 0.8. See SM, files g-S1-Num-expl, g-S1-St4-comp-after-add-C, g-S1-St6-comp-after-add-exc-C, v-key-ABCW, v-verif-ABCW.
2. Alternatively, the system consisting of equations obtained from K_1_ to K_5_ can be solved numerically for z = ma, with m between 0 and 1; m is the yield of AC with respect to A. From the ratio K_4_/K_5_, we have u = txF, where F = K_5_/(K_4_z), and from K_1_K_4_/(K_2_K_3_) = (x/z)^2^ (t/y), we have y = x^2^tE, where E = K_2_K_3_/(K_1_K_4_z^2^). Now, the ratio K_1_/K_3_ = (x/z)[(c-z-2t-u)/(b-x-2y-u)] becomes K_1_/K_3_ = (x/z)[c-z-t (Fx + 2)]/[b-x-t (2Ex + F)x] and the solution of this equation in t is t = (Ax-B)/[x (Cx + D)], where A = K_3_c + ma (K_1_-K_3_), B = K_1_mab, C = K_3_(K_5_-2K_2_)/(K_4_ma) and D = (2K_3_K_4_-K_1_K_5_)/K_4_. From this expression of t, we obtain y = tx^2^E = xE (Ax-B)/(Cx + D) and u = txF = F (Ax-B)/(Cx + D). The expressions (in x) of y, t and u are introduced into the ratio K_3_/K_4_ = z^2^/[t (a-x-y-z-t-u)]. This leads to the following equation in x: (Ax-B)[(a-z) (Cx^2^ + Dx)-x^2^(Cx + D)-(Ex^2^ + Fx + 1) (Ax-B)] = Gx^2^(Cx + D)^2^, where G = K_4_z^2^/K_3_. This quartic equation becomes, after replacement of z by ma and rewriting, αx^4^ + βx^3^ + γx^2^ + δx + ε = 0, where α = -A (AE + C)-C^2^G, β = A [aC (1-m)-AF + 2BE-D] + C(B-2DG), γ = A [aD (1-m)-A + 2BF] + B [D-aC (1-m)-BE]-D^2^G, δ = B [2A-BF-aD (1-m)] and ε = -B^2^. Each of the roots of this quartic equation is introduced (in Excel) into the expressions of y, u and t, which become, like x, functions of z and, consequently, of m. The expressions of x, y, t and u are introduced (in Excel) into the equation obtained from K_5_, namely K_5_ [x (c-z-2t-u)] - u (w + x + 2y + z + 2t + 2u) = 0. Now, in this last equation, all unknowns are functions of m. It can be solved numerically (in Excel) for m∈ (0,1). See SM, files g-S1-Num-expl, g-S1-St4-comp-after-add-C-altern, g-S1-St6-comp-after-add-exc-C-altern.
Sections 5.2.1, 5.2.2 and 5.2.3 are, here, treated independently of each other.

#### 5.2.3 Supplementary amount (excess) of amine C

We now suppose that the reaction mixture with the constants K_1_ to K_5_ and the initial concentrations a, b, c and w, is equilibrated and that this equilibrium state is associated to the yield n = t/a. One wishes to calculate the supplementary amount of amine C that is to be added to the mixture, so that the yield at equilibrium be p = t/a. To this purpose, i) one may calculate the composition of the equilibrated reaction mixture for initial concentrations a, b, c and w, and, starting from that composition, calculate the supplementary amount of C that leads to the yield p, or ii) thanks to the dynamic character of the equilibriums, one may consider the initial reaction mixture, before the formation of any imine, and calculate the amount of compound C, so that t/a = p.

1. We use here the second method. The initial concentrations in the reaction mixture are a, b, c + g_C_ and w, where g_C_ is the supplementary amount of amine C, expressed as a concentration. We have t = ap and K_1_K_4_/(K_2_K_3_) = (x/z)^2^ t/y = (x/z)^2^ap/y. From the ratio K_4_/K_5_ we obtain x = uzK_4_/(apK_5_), which is introduced into the expression of K_1_K_4_/(K_2_K_3_) and leads to y = u^2^K_2_K_3_K_4_/(K_1_K_5_^2^ap). x and y are introduced into the expression of K_3_/K_4_, which leads, after rewriting, to the quadratic equation in z: z^2^ + {[1 + uK_4_/(K_5_ap)]K_3_ap/K_4_}z + K_3_ap{u [1 + uK_2_K_3_K_4_/(K_1_K_5_^2^ap)]-a (1-p)}/K_4_ = 0 or z^2^ + Pz + Q = 0, where O = uK_4_/(K_5_t), P = (O + 1)K_3_t/K_4_ and Q = K_3_ap{u [1 + uK_2_K_3_K_4_/(K_1_K_5_^2^ap)]-a (1-p)}/K_4_ = -K_3_ (a-t-u-y)t/K_4_, with the roots z_1,2_ = [-P±(P^2^-4Q)^1/2^]/2. Now x = Oz, y = K_2_K_3_O^2^t/(K_1_K_4_) and z are all functions of u = ar and they can be introduced into the expression of K_2_. The resulting equation K_2_ [x (b-x-2y-u)] - y (w + x + 2y + z + 2t + 2u) = 0 can be solved numerically for u = ar, where 0 < r < 1. The supplementary amount of C, g_C_ can be calculated from K_4_, g_C_ = t (w + x + 2y + z + 2t + 2u)/(K_4_z) + z + 2t + u-c, or from K_1_/K_3_, g_C_ = (K_1_/K_3_) (z/x) (b-x-2y-u) + z + 2t + u-c. See SM, files g-S1-Num-expl, g-S1-St5-calc-suppl-amt-C.
2. Alternatively, in view of a numerical solution, one can express z as ma, where m is the yield [AC]_eq_/a = z/a and lies between 0 and 1. We still have t = ap. We also have K_4_/K_5_ = xt/zu or x = uA, where A = (K_4_/K_5_) (z/t). From K_3_/K_4_, we obtain, after rewriting, x + y + z = B, where B = a-z-t-(K_4_/K_3_) (z^2^/t). Both A and B are functions of z and, consequently, of m = z/a. Further, x = uA is introduced into the expression of B, that becomes B = y + uC, where C = A + 1; y = B-uC. The expressions x = Au and y = B-uC are introduced into that of K_2_ and lead, after rewriting, to the following quadratic equation in u, u^2^D + uE + F = 0, where D = K_2_A (2C-A-1) + C (A-2C + 2) = (K_2_-1)A (A + 1), E = K_2_A (b-2B)-B (A-2C + 2) + C (w + 2B + z + 2t) = A [K_2_ (b-2B) + B] + C (w + 2B + z + 2t) and F = -B (w + 2B + z + 2t). The discriminant is Δ = E^2^-4DF and the roots are u_1,2_ = [-E±(E^2^-4DF)^1/2^]/2D, which are all functions of z. All expressions of unknowns x, y and t as functions of z are now introduced in that of the equation obtained from K_1_/K_2_, i.e. K_1_y(a-x-y-z-t-u)-K_2_x^2^ = 0. This equation can be solved numerically (in Excel) for m between 0 and 1. Further, the supplementary amount of amine C, g_C_ (expressed as a concentration), is calculated as described above. See SM, file g-S1-St5-calc-suppl-amt-C-altern.

#### 5.2.4 Influence of water. A numerical example

**FIGURE 19:**
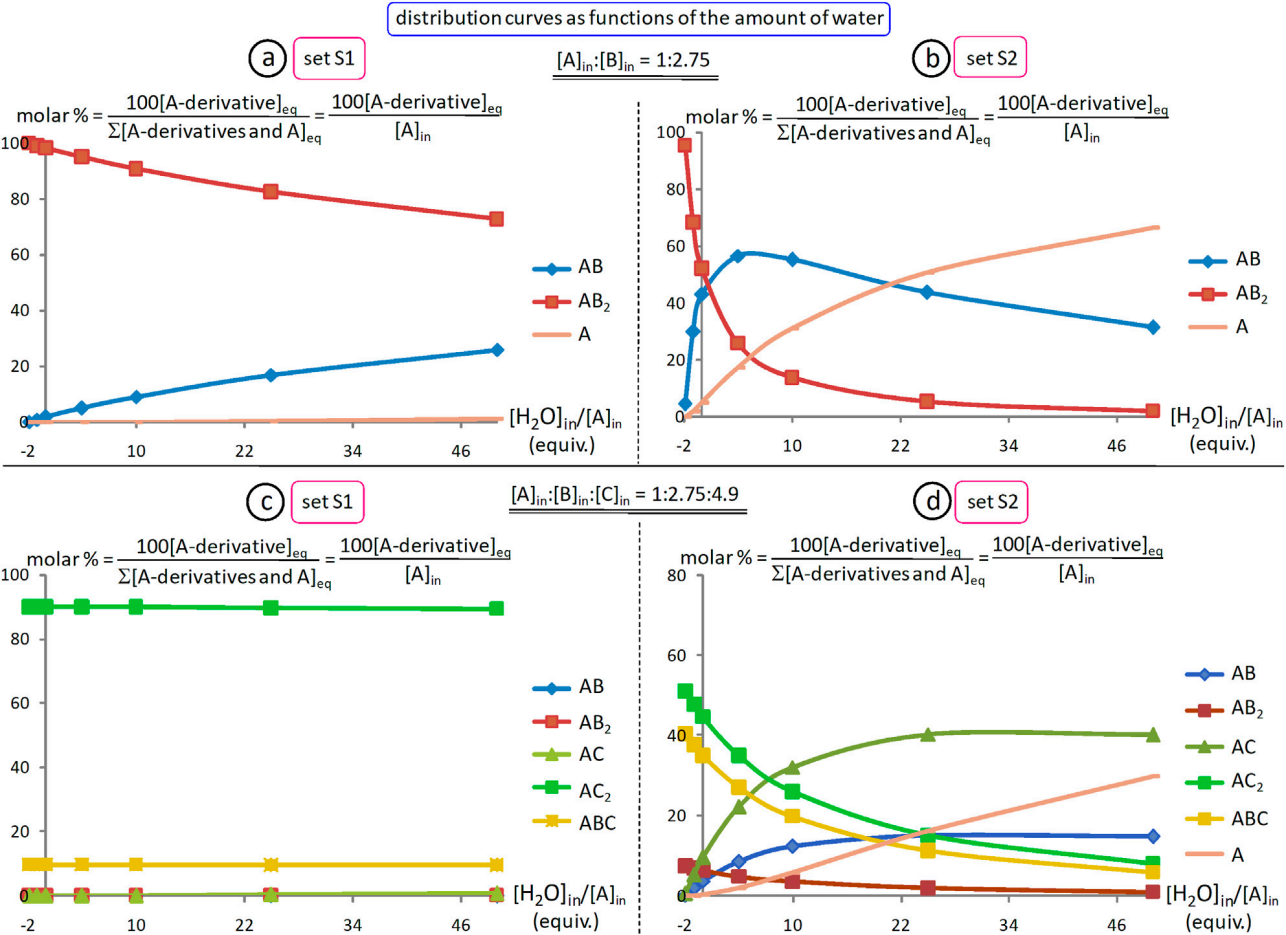
Theoretically calculated distribution curves at equilibrium (related to the Section 5.2.4) representing molar percentages of dialdehyde A and its derivatives (with respect to the initial amount of A) as functions of the initial number of equivalents of water (with respect to A), and corresponding to reaction mixtures (general approach) obtained from: **(a,b)** dialdehyde A and amine B (molar ratio 1:2.75; initial concentrations before formation of any imines [A]_in_ = 6.20 × 10^−3^ M, [B]_in_ = 17.05 × 10^−3^ M; (a) → set S1 of constants, K_1_ = 10^3^, K_2_ = 140; **(b)** → set S2 of constants, K_1_ = 10, K_2_ = 1.4); **(c,d)** dialdehyde A and amines B and C (molar ratio 1:2.75:4.9; initial concentrations before formation of any imines [A]_in_ = 6.20 × 10^−3^ M, [B]_in_ = 17.05 × 10^−3^ M, [C]_in_ = 30.38 × 10^−3^ M; (c) → set S1 of constants, K_1_ = 10^3^, K_2_ = 140, K_3_ = 16.5 × 10^3^, K_4_ = 2.35 × 10^3^, K_5_ = 4.75 × 10^3^; **(d)** → set S2 of constants, K_1_ = 10, K_2_ = 1.4, K_3_ = 16, K_4_ = 2.4, K_5_ = 4.8). Yields, expressed as molar percentages, are all calculated with respect to the total (initial) amount of A. See SM, files: g-S1-A1B2_75-f-water-crv **(a)**, g-S2-A1B2_75-f-water-crv **(b)**, g-S1-A1B2_75C4_9-W-crv **(c)** and g-S2-A1B2_75C4_9-W-crv **(d)**. SM = the supplementary material.

- Framework. In a numerical example (see SM, file g-S1-Num-expl) we theoretically modeled, through calculations, several of the steps we follow in practice, when implementing a pH-controlled covalent switch. The imine formation constants (set S1: K_1_ = 10^3^, K_2_ = 140, K_3_ = 16.5 × 10^3^, K_4_ = 2.35 × 10^3^, K_5_ = 4.75 × 10^3^) which we considered, are in or close to the order of magnitude of those roughly determined for reactions of dialdehyde **dialpy** with amines **L4** and **R4** (K_1_ = 9.0 × 10^2^, K_2_ = 1.6 × 10^2^, K_3_ = 15.0 × 10^3^, K_4_ = 1.8 × 10^3^, K_5_ = 3.2 × 10^3^; vide supra, Section 2.3), and the concentrations of compounds A, B and C are in the range of our working concentrations. For a mixture of dialdehyde A and amine, B with [B]_in_/[A]_in_ = 2 and [A]_in_ = 6.20 mM, we calculated the composition at equilibrium (step 1), the excess of B required to reach a desired yield of AB_2_ (which led to [B]_in_/[A]_in_ = 2.75; step 2) and the new composition at equilibrium after addition of the excess of B (step 3). We further calculated the composition at equilibrium after addition of 2.75 equiv. of amine C (step 4), the supplementary amount of C required to reach a desired yield of AC_2_ (which led to [C]_in_/[A]_in_ = 4.9; step 5) and the new composition at equilibrium after the addition of the excess of C (step 6). See SM, file g-S1-Num-expl. The seventh step deals with the influence of the pH (vide infra).
- Method. In order to look at the influence of the amount of water on the concentration of bis-imines in equilibrated reaction mixtures, we considered the calculated initial concentrations (before formation of imines) resulted from the sixth step of this numerical example ([A]_in_ = 6.20 mM, [B]_in_ = 17.05 mM, [C]_in_ = 30.38mM; [A]_in_: [B]_in_: [C]_in_ = 1:2.75:4.9). For these concentrations, we calculated the composition at equilibrium for reaction mixtures of A and B (for the formation of AB_2_) and of A, B and C (for the formation of AC_2_ upon addition of C to the equilibrated mixture of A and B), where [H_2_O]_in_/[A]_in_ = −1.9, −1, 0, 4, 10, 25 and 50. Comparatively, to the same concentrations [A]_in_, [B]_in_ and [C]_in_ and ratio [H_2_O]_in_/[A]_in_, we associated a second set S2 of lower imine formation constants: K_1_ = 10, K_2_ = 1.4, K_3_ = 16, K_4_ = 2.4, K_5_ = 4.8. The set S2 is a hypothetical one obtained by simply lowering (through division by 10^2^ or 10^3^ and rounding) the constants from the set S1.
- Results.
  1. Mixture of A and B. For the set S1, in the equilibrated reaction mixture of A and B (Figure 19a), when the ratio [H_2_O]_in_/[A]_in_ increases from −1.9 to 50, the percentage 100[AB_2_]_eq_/[A]_in_ decreases from practically 100% to about 73%. In parallel, the percentage 100[AB]_eq_/[A]_in_ increases from, roughly, 1%–26%, while 100[A]_eq_/[A]_in_ remains lower than 1.3%. For the set S2 (Figure 19b), the yield of bis-imine AB_2_ dramatically diminishes from about 95% to about 2%, that of AB increases from about 5% to a maximum of about 57%, then decreases to about 32%, while 100[A]_eq_/[A]_in_ increases from about 0% to about 66%.
  See SM, files g-S1-A1B2_75-f-water-crv, g-S2-A1B2_75-f-water-crv
  2. Mixture of A, B and C. In the equilibrated reaction mixture of A, B and C, in the case of set S1, when the ratio [H_2_O]_in_/[A]_in_ increases from −1.9 to 50, the percentages 100[bis-imine]_eq_/[A]_in_ for the three bis-imines AB_2_, ABC and AC_2_ remain, practically, unchanged (percent change 100|conc_-1.9_-conc_50_|/conc_-1.9_ is less than 1.1%) (Figure 19c). For the set S2, the percentage 100[bis-imine]_eq_/[A]_in_ considerably diminishes (Figure 19d), namely, roughly, AB_2_ from 7% to 1%, AC_2_ from 51% to 8%, and ABC from 41% to 6%, with an average percent change 100|conc_-1.9_-conc_50_|/conc_-1.9_ of 85%. In parallel, 100[compound]_eq_/[A]_in_ increases from less than 1% to, roughly, 15% (AB), 40% (AC) and 30% (A).
  See SM, files g-S1-A1B2_75C4_9-W … (8 files), g-S2-A1B2_75C4_9-W … (8 files).

### 5.3 Influence of the pH. General approach. Equilibrium-constants-based modeling of the pH-adaptive behavior of a small dynamic library of imines

Reaction of a mixture of A, AB, AB_2_ and B with amine C, leads to a mixture containing, in principle, the 8 following organic species: 1 dialdehyde (A), 2 aldehyde-imines (AB, AC), 3 bis-imines (AB_2_, AC_2_, ABC) and 2 amines (B, C). The same holds for the reaction of dialdehyde A with a mixture of amines B and C. The ways to modulate such dynamic covalent libraries of imines include supramolecular interactions (Gambaro et al., 2020), distillation (Osowska and Miljanić, 2011), crystallization (Chow et al., 2007), phase separation (Hafezi and Lehn, 2012; Pérez-Fernández et al., 2008), and the pH (Giuseppone and Lehn, 2006).

If one wishes to represent, in a rather quantitative manner, the composition of the equilibrated reaction mixture as a function of the pH (concentration = ƒ(pH)), then, one may consider that the system can be described, thanks to its dynamic character, through the following equations:

$$A+B⇌\text{AB}+H_{2}O\;K_{1}=x\left(w+x+2y+z+2t+2u\right)/\left[\left(a-x-y-z-t-u\right)\left(b-x-2y-u-v\right)\right]$$

$$\text{AB}+B⇌{\text{AB}}_{2}+H_{2}O\;K_{2}=y\left(w+x+2y+z+2t+2u\right)/\left[x\left(b-x-2y-u-v\right)\right]$$

$$A+C⇌\text{AC}+H_{2}O\;K_{3}=z\left(w+x+2y+z+2t+2u\right)/\left[\left(a-x-y-z-t-u\right)\left(c-z-2t-u-s\right)\right]$$

$$\text{AC}+C⇌{\text{AC}}_{2}+H_{2}O\;K_{4}=t\left(w+x+2y+z+2t+2u\right)/\left[z\left(c-z-2t-u-s\right)\right]$$

$$\text{AB}+C⇌\text{ABC}+H_{2}O\;K_{5}=u\left(w+x+2y+z+2t+2u\right)/\left[x\left(c-z-2t-u-s\right)\right]$$

$$B+H^{+}⇌{\text{BH}}^{+}\;1/K_{6}=v/\left[h\left(b-x-2y-u-v\right)\right]$$

$$C+H^{+}⇌{\text{CH}}^{+}\;1/K_{7}=s/\left[h\left(c-z-2t-u-s\right)\right]$$

where the following was added or changed in the above notations for concentrations: [B]_eq_ = b-x-2y-u-v, [C]_eq_ = c-z-2t-u-s, [BH^+^]_eq_ = v, [CH^+^]_eq_ = s and [H^+^]_eq_ = h. This approach does not include equations which describe the protonation of pyridines and imines, the dissociation of water or the formation of aminals, hemiaminals and hydrates from aldehydes.

From K_6_, we obtain v = (b-x-2y-u)h/(K_6_ + h) and from K_7_, we have s = (c-z-2t-u)h/(K_7_ + h), which lead to b-x-2y-u-v = (b-x-2y-u)K_6_/(K_6_ + h) and c-z-2t-u-s = (c-z-2t-u)K_7_/(K_7_ + h).

Now, the expressions of K_1_ to K_5_ become

$$K_{1}K_{6}/\left(K_{6}+h\right)=K_{1h}=x\left(w+x+2y+z+2t+2u\right)/\left[\left(a-x-y-z-t-u\right)\left(b-x-2y-u\right)\right]$$

$$K_{2}K_{6}/\left(K_{6}+h\right)=K_{2h}=y\left(w+x+2y+z+2t+2u\right)/\left[x\left(b-x-2y-u\right)\right]$$

$$K_{3}K_{7}/\left(K_{7}+h\right)=K_{3h}=z\left(w+x+2y+z+2t+2u\right)/\left[\left(a-x-y-z-t-u\right)\left(c-z-2t-u\right)\right]$$

$$K_{4}K_{7}/\left(K_{7}+h\right)=K_{4h}=t\left(w+x+2y+z+2t+2u\right)/\left[z\left(c-z-2t-u\right)\right]$$

$$K_{5}K_{7}/\left(K_{7}+h\right)=K_{5h}=u\left(w+x+2y+z+2t+2u\right)/\left[x\left(c-z-2t-u\right)\right]$$

The roots of this system are all functions of h = [H^+^]_eq_ = 10^−pH^ and could be used to express the concentrations of the components of the reaction mixture at equilibrium as functions of the pH (concentration-pH distribution curves). To this end, one may choose a range of pH values (for example, those values from 0 to 14 which are natural numbers) and, for each of these pH values, one may numerically solve the system based on K_1h_ to K_5h_ (like in the case of the system based on K_1_ to K_5_). Vide supra, Section 5.2.2.1. It may happen, like in our examples, that a root of the quartic equation is appropriate only for one or two pH intervals (e.g. for 0 ≤ pH ≤ 1 and 10 ≤ pH ≤ 14), while another root is appropriate for the remaining pH interval (e.g. for 2 ≤ pH ≤ 9). See SM, file g-S1-St7-pH-crv.

**FIGURE 20:**
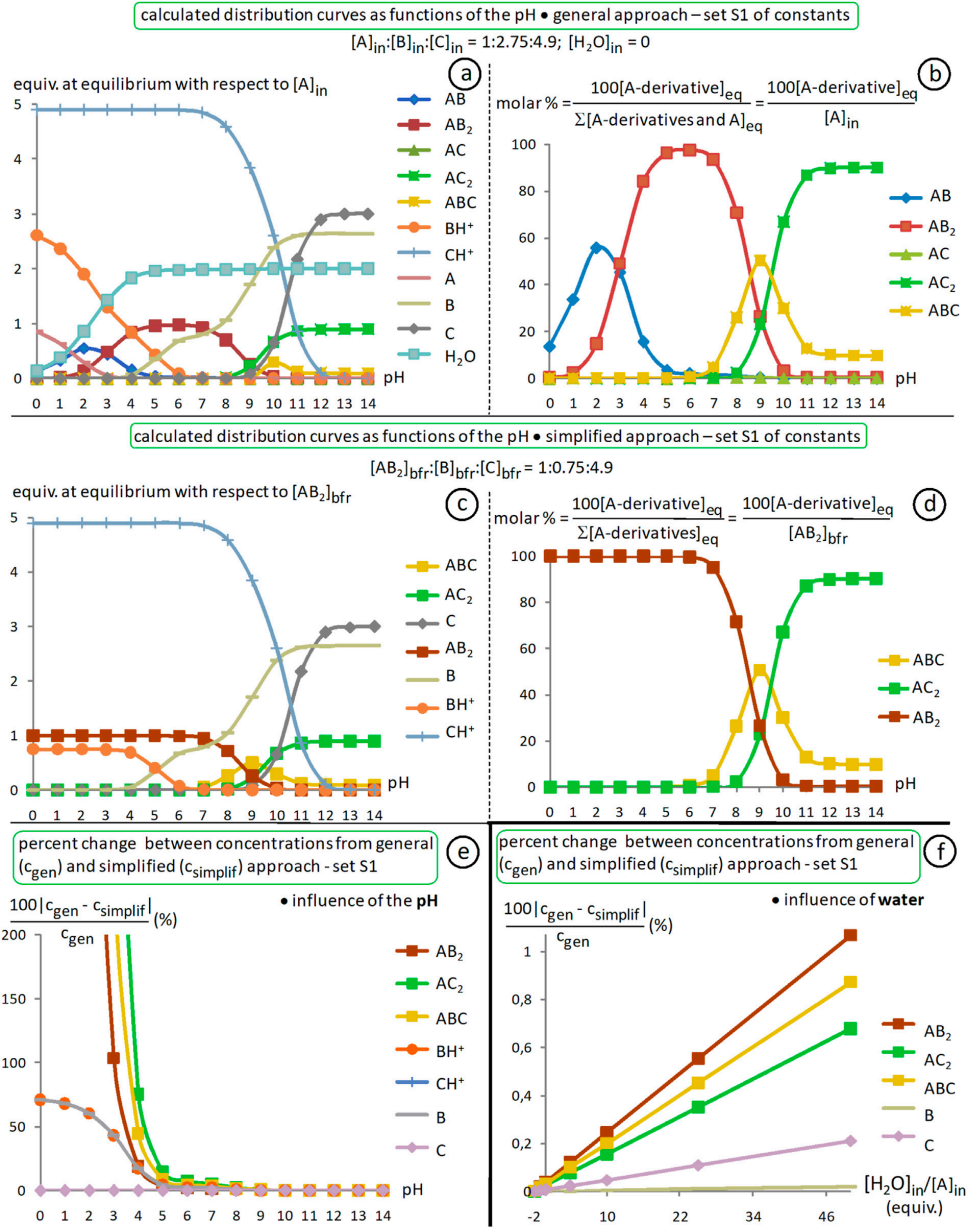
For the set S1 of constants (K_1_ = 10^3^, K_2_ = 140, K_3_ = 16.5 × 10^3^, K_4_ = 2.35 × 10^3^, K_5_ = 4.75 × 10^3^, K_6_ = 10^−5.07^, K_7_ = 10^−10.6^, K_I_ = K_5_/K_2_, K_II_ = K_3_K_4_/(K_2_K_5_)): **(a–d)** theoretically calculated distribution curves (see section 5.3) representing equivalents at equilibrium and molar percentages of dialdehyde A and its derivatives (with respect to the initial amount of A), as functions of the pH, and corresponding to equilibrated reaction mixtures obtained from **(a,b)** dialdehyde A and amines B and C (molar ratio 1:2.75:4.9; initial concentrations before formation of any imine [A]_in_ = 6.20 × 10^−3^ M, [B]_in_ = 17.05 × 10^−3^ M, [C]_in_ = 30.38 × 10^−3^ M, the initial concentrations of other species before formation of any imine being 0) (general approach), and **(c,d)** bis-imine AB_2_ and amines B and C (molar ratio 1:0.75:4.9; concentrations before reaction [AB_2_]_bfr_ = 6.20 × 10^−3^ M, [B]_bfr_ = 4.65 × 10^−3^ M, [C]_bfr_ = 30.38 × 10^−3^ M) (simplified approach, where the amounts of compounds possessing free, unreacted CHO groups, are seen as practically negligible). Numbers of equivalents **(a,c)** and yields **(b,d)** are all calculated with respect to the total (initial) amount of A; **(e,f)** differences between concentrations from the general (c_gen_) approach and those from the simplified (c_simplif_) approach expressed as a percentage from c_gen_, namely 100|c_gen_-c_simplif_|/c_gen_, and plotted as functions of the pH (e; [H_2_O]_in_ = 0) and as functions of the initial number of equivalents of water with respect to A, before formation of imines and in the absence of acid (f; [H_2_O]_in_/[A]_in_ (general approach) = −1.9, −1, 0, 4, 10, 25, 50). See SM, files g-S1-St7-pH-crv **(a,b)**, s-S1-pH-crv **(c,d)**, c-S1-perc-chg-f-pH **(e)** and c-S1-perc-chg-f-water-graph **(f)**.

**FIGURE 21:**
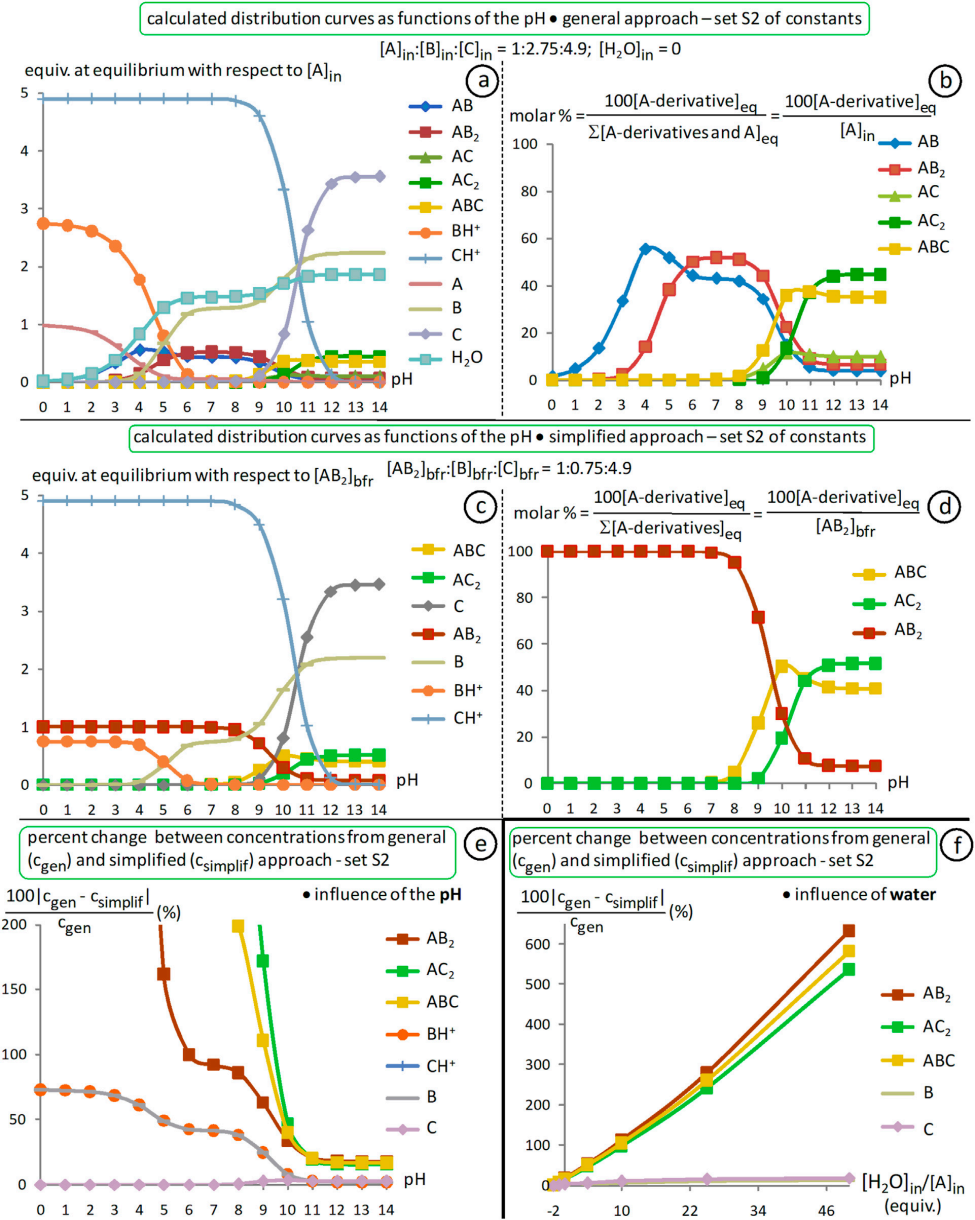
For the set S2 of constants (K_1_ = 10, K_2_ = 1.4, K_3_ = 16, K_4_ = 2.4, K_5_ = 4.8, K_6_ = 10^−5.07^, K_7_ = 10^−10.6^, K_I_ = K_5_/K_2_ and K_II_ = K_3_K_4_/(K_2_K_5_)): **(a–d)** theoretically calculated distribution curves (see section 5.3) representing equivalents at equilibrium and molar percentages of dialdehyde A and its derivatives (with respect to the initial amount of A), as functions of the pH, and corresponding to equilibrated reaction mixtures obtained from **(a,b)** dialdehyde A and amines B and C (molar ratio 1:2.75:4.9; initial concentrations before formation of any imines [A]_in_ = 6.20 × 10^−3^ M, [B]_in_ = 17.05 × 10^−3^ M, [C]_in_ = 30.38 × 10^−3^ M, the initial concentrations of other species before formation of any imine being 0) (general approach), and **(c,d)** bis-imine AB_2_ and amines B and C (molar ratio 1:0.75:4.9; concentrations before reaction [AB_2_]_bfr_ = 6.20 × 10^−3^ M, [B]_bfr_ = 4.65 × 10^−3^ M, [C]_bfr_ = 30.38 × 10^−3^ M) (simplified approach, where the amounts of compounds possessing free, unreacted CHO groups, are seen as practically negligible). Numbers of equivalents **(a,c)** and yields **(b,d)** are all calculated with respect to the total (initial) amount of A; **(e,f)** differences between concentrations from the general (c_gen_) approach and those from the simplified (c_simplif_) approach expressed as a percentage from c_gen_, namely 100|c_gen_-c_simplif_|/c_gen_, and plotted as functions of the pH (e; [H_2_O]_in_ = 0) and as functions of the initial number of equivalents of water with respect to A, before formation of imines and in the absence of acid (f; [H_2_O]_in_/[A]_in_ (general approach) = −1.9, −1, 0, 4, 10, 25, 50). See SM, files g-S2-St7-pH-crv **(a,b)**, s-S2-pH-crv **(c,d)**, c-S2-perc-chg-f-pH **(e)** and c-S2-perc-chg-f-water-gaph” **(f)**.

In order to establish pH-dependent distribution curves, we applied this general approach to the above mentioned (vide supra, Section 5.2.4) set of initial molar concentrations (before formation of any imine) [A]_in_ = 6.2 mM, [B]_in_ = 17.05 mM (2.75 equiv.), [C]_in_ = 30.38 mM (4.9 equiv.) and [H_2_O]_in_ = 0, which can be seen as a typical example. To them are associated the two sets S1 and S2 of constants K_1_ - K_5_ and the constants K_6_ = 10^−5.07^ and K_7_ = 10^−10.6^ (average acidity constants of protonated amines in water; acidity constants are expected to have different values between water and chloroform; however, those for water were used here for the sake of approximation, those for chloroform being unavailable). The results obtained for natural pH values from 0 to 14, were used to plot distribution curves (Figures 20a,b, 21a,b). For the set S1 of constants, the highest amount of aromatic bis-imine AB_2_ forms at a pH between 5 and 7 (96% with respect to the total amount of A), while the highest amount of aliphatic bis-imine AC_2_ (89%) appears for pH values greater than 10. Correlatively, for the set S2, a maximum of AB_2_ (about 51%) is noticed for 6 ≤ pH ≤ 8, and a maximum of AC_2_ (about 44%), for 12 ≤ pH ≤ 14 (the percentages are average values). See SM, files g-S1-Num-expl, g-S1-St7-pH0 to g-S1-St7-pH14, g-S1-St7-pH-crv, g-S2-St7-pH0 to g-S2-St7-pH14, g-S2-St7-pH-crv.

Such pH-dependent distribution curves make possible the theoretical exploration of pH domains of interest.

### 5.4 Simplification of the treatment of exchanges

If the equilibriums in which compounds (here, A, AB and AC) possessing aldehyde groups are involved, could be seen, under particular conditions, as playing only a minor role, then the equilibrated reaction mixture can be described in a simplified way, where these equilibriums do not longer appear. This may be done when the reaction mixture contains practically no compounds possessing carbonyl groups capable of reacting with amines (all CHO groups appear, in this framework, as imines) and the formation of such compounds through hydrolysis could be seen as negligible (presence of water, but enough high formation constants of imines or a significant excess of amines, or “absence” of water).

Hereafter we present an example of such calculations. The mathematical treatment (based on 2 polynomial equations corresponding to K_I_ and K_II_; for the composition at equilibrium before addition of acid, the equation of highest degree to be solved is a cubic one, which can be solved analytically) of the mixture at equilibrium is simplified by comparison with the general approach (5 polynomial equations corresponding to K_1_-K_5_). This can be, for example, the case when one starts from anhydrous bis-imine AB_2_ (which is further treated with amine C), under the assumption that hydrolysis of imines can be seen as negligible.

#### 5.4.1 Composition at equilibrium. Simplified approach

Under such conditions, for the reaction of a dialdehyde-based bis-imine with a primary amine (see Section 5.2), instead of equilibriums A (dialdehyde) + B (amine) ⇌ AB+ H_2_O (K_1_), AB + B ⇌ A_2_B+ H_2_O (K_2_), A+ C (amine) ⇌ AC + H_2_O (K_3_), AC + C ⇌ AC_2_ + H_2_O (K_4_) and AB+ C ⇌ ABC + H_2_O (K_5_), we have

$${\text{AB}}_{2}+C⇌\text{ABC}+B\;K_{I}=K_{5}/K_{2}=\left(j+e\right)\left(l+j+2k\right)/\left[\left(d-j-k\right)\left(g-j-2k\right)\right]$$

$$\text{ABC}+C⇌{\text{AC}}_{2}+B\;K_{\text{II}}=K_{3}K_{4}/\left(K_{1}K_{5}\right)=\left(k+f\right)\left(l+j+2k\right)/\left[\left(j+e\right)\left(g-j-2k\right)\right]$$

with the concentrations before reaction [AB_2_]_bfr_ = d, [C]_bfr_ = g, [ABC]_bfr_ = e, [AC_2_]_bfr_ = f, [B]_bfr_ = l and the concentrations at equilibrium [AB_2_]_eq_ = d-j-k, [C]_eq_ = g-j-2k, [ABC]_eq_ = j + e, [AC_2_]_eq_ = k + f, [B]_eq_ = l + j + 2k. j and k are unknowns: j = [ABC]_formed_, k = [AC_2_]_formed_.

In order to calculate the composition at equilibrium, one introduces the notations χ = j + e = [ABC]_eq_ and ζ = k + f = [AC_2_]_eq_ and one obtains K_I_/K_II_ = χ^2^/{ζ[(d + e + f)- χ-ζ]}. This leads to the equation (in χ) K_II_χ^2^ + K_I_ζχ + K_I_ζ(ζ-d-e-f) = 0 with the discriminant Δ = Eζ^2^ + Fζ, where E = K_I_(K_I_-4K_II_) and F = 4K_I_K_II_(d + e + f). χ_1,2_ = (-K_I_ζ±Δ^1/2^)/(2K_II_). The positive χ should be χ = (Δ^1/2^-K_I_ζ)/(2K_II_). Introduced into the expression of K_II_, it leads to the equation (in ζ) Gζ^2^ + Hζ=(Iζ + J) (Eζ^2^ + Fζ)^1/2^, where G = K_I_(4K_II_-K_I_ + 1)-4K_II_, H = -K_II_ [K_I_(g + 2d + 3e + 4f) + 2 (l-e−2f)], I = -K_I_ + 2K_II_ + 1 and J = -K_II_(g + e + 2f). After elimination of radicals, simplification and rewriting, one obtains the cubic equation (in ζ) αζ^3^ + βζ^2^ + γζ + δ = 0, where α = G^2^-EI^2^, β = 2GH-2EIJ-FI^2^, γ = H^2^-EJ^2^-2FIJ, δ = -FJ^2^. This equation can be solved analytically. From the solution ζ = k + f = [AC_2_]_eq_, we obtain k = ζ-f = [AC_2_]_formed_, as well as χ = j + e = (Δ^1/2^-K_I_ζ)/(2K_II_) = [ABC]_eq_ and j = χ-e = [ABC]_formed_. See SM, files s-comp-equil, s-S1-S2-Num-expl, s-S1-comp-equil, s-S2-comp-equil. The imine exchange constants from the set S1 are K_I_ = K_5_/K_2_ ≈ 33.9 and K_II_ = K_3_K_4_/(K_1_K_5_) ≈ 8.2 and those from the set S2 are K_I_ = K_5_/K_2_ ≈ 3.4 and K_II_ = K_3_K_4_/(K_1_K_5_) = 0.8 (for K_1_-K_5_ from each set, vide supra, section 5.2.4).

If [A]_in_ = a, [B]_in_ = b, [C]_in_ = c and [H_2_O]_in_ = w are the total, initial (→ in) concentrations before the formation of any imine, and [AB_2_]_bfr_ = d, [AC_2_]_bfr_ = f, [ABC]_bfr_ = e, [B]_bfr_ = l, [C]_bfr_ = g and [H_2_O]_bfr_ = w_bfr_ are the starting concentrations before reaction (→ bfr), then the following relationships of equivalence held: a = d + e + f, b = 2d + e + l, c = g + e + 2f and w = w_bfr_ - 2 (d + e + f). One notices that [H_2_O]_bfr_ does not appear in the simplified approach.

#### 5.4.2 Influence of the pH

In addition to the two transiminations, one considers the protonation of amines B and C, as follows

$${\text{AB}}_{2}+C⇌\text{ABC}+B\;K_{I}=\left(j+e\right)\left(l+j+2k-v\right)/\left[\left(d-j-k\right)\left(g-j-2k-s\right)\right]$$

$$\text{ABC}+C⇌{\text{AC}}_{2}+B\;K_{\text{II}}=\left(k+f\right)\left(l+j+2k-v\right)/\left[j\left(g-j-2k-s\right)\right]$$

$$B+H^{+}⇌{\text{BH}}^{+}\;1/K_{6}=v/\left[h\left(l+j+2k-v\right)\right]$$

$$C+H^{+}⇌{\text{CH}}^{+}\;1/K_{7}=s/\left[h\left(g-j-2k-s\right)\right]$$

with the following changes in the concentrations at equilibrium [BH^+^]_eq_ = v, [B]_eq_ = l + j+2k-v, [CH^+^]_eq_ = s, [C]_eq_ = g-j-2k-s and [H^+^]_eq_ = h.

From K_6_ and K_7_, we have v = h (l + j + 2k)/(K_6_ + h) and s = h (g-j-2k)/(K_7_ + h), which, introduced into the expressions of K_I_ and K_II_ lead to (j + e) (l + j + 2k)/[(d-j-k) (g-j-2k)] = K_I_K_7_(K_6_ + h)/[K_6_(K_7_ + h)] = K_Ih_ and (k + f) (l + j + 2k)/[j (g-j-2k)] = K_II_K_7_(K_6_ + h)/[K_6_(K_7_ + h)] = K_IIh_. In order to establish distribution curves (concentrations as functions of the pH; Figures 20c,d, 21c,d), one may consider (natural) pH values between 0 and 14, and solve the system of equations obtained from K_Ih_ and K_IIh_, for these values of the pH. See SM, files s-S1-S2-Num-expl, s-S1-pH, s-S1-pH-crv, s-S2-pH, s-S2-pH-crv.

#### 5.4.3 Addition of an amount of acid

A known amount of acid HX (here, usually, CF_3_COOH; K_a_ = K_8_) is added (in a small volume of solvent, that makes possible the approximation that the volume of the reaction mixture remains constant) to the equilibrated reaction mixture obtained after addition of amine C. The molar concentration of HX in the reaction mixture before reaction is m and one wishes to calculate the concentrations of species at equilibrium. The equation to be added to those from section 5.4.2, is

$$\text{HX}⇌H^{+}+X^{-}\;K_{8}=h\left(h+v+s\right)/\left[m-\left(h+v+s\right)\right]$$

with [HX]_bfr_ = m, [X^−^]_eq_ = h + v + s and [HX]_eq_ = m-(h + v + s).

The equation to be solved is that obtained from K_8_, namely (h + v + s) (K_8_ + h)-mK_8_ = 0. This was done numerically, in Excel, for r = h/m with 0 < r < 1 (r is, here, the degree of dissociation of HX). h is a function of r, but v and s are functions of h and, consequently, of r, and so, the whole expression (h + v + s) (K_8_+h)-mK_8_ is a function of r∈ (0,1). See SM, files s-S1-S2-Num-expl, s-S1-aj-acid.

#### 5.4.4 Addition of an amount of base

A known amount of base (usually triethylamine TEA) is added to the equilibrated reaction mixture obtained after addition of acid, the volume being seen as constant. The molar concentration of base before reaction is p. The supplementary equilibrium to be considered, in addition to those from sections 5.4.2 and 5.4.3, is

$$\text{Base}+H^{+}⇌{\text{BaseH}}^{+}\;1/K_{9}=i/\left[h\left(p-i\right)\right]$$

with [Base]_bfr_ = p, [HX]_bfr_ = m, [Base]_eq_ = i, [X^−^]_eq_ = h + v + s + i and [HX]_eq_ = m-(h + v + s + i). From K_9_, we have h = K_9_i/(p-i). We also have K_8_ = h (h + v + s + i)/[m-(h + v + s + i)] and the equation to be solved is (h + v + s + i) (K_8_+h)-mK_8_ = 0. i can be written as i = qp, where q ∈ (0,1) is the degree of protonation of the base. In this way, unknowns h, v, s and i are functions of q and the above equation from K_8_ can be solved numerically (in Excel) for q between 0 and 1. See SM, files s-S1-S2-Num-expl, s-S1-aj-base.

#### 5.4.5 Comparison between general and simplified approaches

- Set S1 of constants. Thanks to excesses of amines and to enough strong constants from set S1, the distribution of bis-imines AB_2_, ABC and AC_2_ is, for 7 ≤ pH ≤ 14 (e), quite similar between the general approach, with [H_2_O]_in_ = 0, and the simplified one. For a quantitative comparison, we used the percentage change 100|c_gen_-c_simplif_|/c_gen_ which is, for that pH range, lower than 5.6%. At pH = 6, this percentage change for bis-imines is between 2% and 7.5%, at pH = 5, it lies between 3.9% and 14.5%, and for 0 ≤ pH ≤ 4, it is superior to 18.8% (Figure 20e; see SM, file c-S1-perc-chg-f-pH). The influence of water from the general approach - water does not appear in the calculations from the simplified approach -, in the absence of acid, is very weak (Figure 20f). Indeed, for [H_2_O]_in_/[A]_in_ from −1.9–50 equiv., the difference 100|c_gen_-c_simplif_|/c_gen_ lies between 3.8 × 10^−5^% and 1.1%. See SM, files s-S1-S2-Num-expl, c-S1-perc-chg-f-water, c-S1-perc-chg-f-water-graph.
- Set S2 of constants. In the case of set S2, the formation constants of imines are quite low, and even with an excess of 0.75 equiv. of amine B and an excess of 2.9 equiv. of amine C, the difference 100|c_gen_-c_simplif_|/c_gen_ for AB_2_, ABC and AC_2_ is, in the pH range from 0 to 14 and for [H_2_O]_in_ = 0, greater than 15% (Figure 21e). See SM, file c-S2-perc-chg-f-pH. In such cases, it is better to consider the general approach. The influence of water (in systems without acid) on the difference between the concentrations of imines calculated in the general approach and those calculated in the simplified approach (Figure 20f), can be seen as weak only for [H_2_O]_in_/[A]_in_ = −1.9 (the negative sign means that 1.9 equiv. of water leave, in the general approach, the system during the equilibration), with the percentage change 100|c_gen_-c_simplif_|/c_gen_ for AB_2_, ABC and AC_2_ between 0.76% and 0.88%. However, 100|c_gen_-c_simplif_|/c_gen_ lies between 7.7% and 8.8% for [H_2_O]_in_/[A]_in_ = −1 and it is bigger than 15% for [H_2_O]_in_ > 0. See SM, files s-S1-S2-Num-expl, c-S2-perc-chg-f-water, c-S2-perc-chg-f-water-graph.

### 5.5 Solving of equations and systems

#### 5.5.1 Solutions of systems

The systems of polynomial equations were solved by substitution. In order to avoid very long or too complicated mathematical expressions in the solving of equations, we introduced notations (usually, capital or Greek letters) corresponding to intermediate parameters or coefficients. In the solving of equations and systems, it is assumed that coefficients, parameters, their combinations and other expressions are nonzero, when they appear as divisors.

#### 5.5.2 Stop-criteria

In the cases where we had to solve equations and/or systems of equations in a numerical way, the variable or unknown (e.g. the concentration of a bis-imine or of an aldehyde-amine) convenient for the solving was expressed as a function of its yield with respect to the difunctional compound (dialdehyde). This yield (let it be, here, r) is necessarily between 0 and 1. One gives to this yield values between 0 and 1, and looks at the sign and general trend of the polynomial function of the equation eq(r), which is to be solved. We did this manually in Excel. If a sign change is found for two values r_1_ and r_2_ of the yield, this means that a root of the equation is between r_1_ and r_2_. One should find, between r_1_ and r_2_, values of r for which eq(r) is as closest to zero as possible. This process is stopped thank to criteria defined by the chemist. One may use, as a stop-criterion related to numerical values of the yield, the number of digits after the decimal separator. To this criterion one should add a second one, which can be the closest to 0 value of eq(r), namely the smallest absolute value of eq(r).

A supplementary means to verify the solutions obtained through numerical methods is the value of the equilibrium constants recalculated on the basis of the roots of the equation. One may consider the sum of the percentages of differences between the starting constants (K) and the recalculated ones (K_recalculated_), namely Σ100ΔK_i_/K_i_, where ΔK = |K_recalculated_-K|. If several numerical solutions which may have a real chemical meaning, are found, one should retain that for which Σ100ΔK_i_/K_i_ is the smallest.

One can, of course, use other numerical methods. See, for example, (Crerar, 1975).

For the sake of mathematical accuracy, when numerically solving the equations in Excel, we sought to obtain a value of the left-hand side, as close as possible to 0, which may lead to mathematical solutions with numerous (sometimes more than 8) digits after the decimal separator. However, in practice such solutions should be rounded (to ones with only 2-3 digits after the decimal separator), considering also the uncertainty associated to the inputs (concentrations, constants).

#### 5.5.3 Verification keys

Sets of arbitrary chosen numerical values of the initial concentrations and of those of newly formed products at equilibrium were used to calculate constants without particular chemical meaning. In this way, one is sure that at least one set of mathematically right roots of the system is known. We use such sets as verification keys, to check if the formulae we wrote in Excel are mathematically right. See SM, files v-key-ABCW, v-key-ABW, v-verif-ABCW, v-verif-ABW.

#### 5.5.4 Analytical solving of cubic equations

For the equation αx^3^ + βx^2^ + γx + δ = 0, one defines its discriminant discr3 (or discrIII) = 18αβγδ-4β^3^δ-27α^2^δ^2^-4αγ^3^ + β^2^γ^2^, μ = (3αγ-β^2^)/(3α^2^) and ν = (27α^2^δ-9αβγ + 2β^3^)/(27α^3^). If discr3 < 0, the cubic equation has one real root and two non-real roots. The real root is the real algebraic one x_r_ = [-ν/2 + (μ^3^/27 + ν^2^/4)^1/2^]^1/3^ + [-ν/2-(μ^3^/27 + ν^2^/4)^1/2^]^1/3^-β/(3α). If discr3 > 0, the cubic equation has three distinct real roots. Its trigonometric solution is based on φ = arccos [(-ν/2)/(-μ^3^/27)^1/2^]. In this case (said casus irreducibilis), one has x_1,2,3_ = -β/(3α) + 2 (-μ/3)^1/2^cos [(φ + 2kπ)/3], where k = 0, 1, 2. In our Excel files, the discriminant may appear, but it is not systematically calculated. We preferred to introduce the numerical data into the formulae for both algebraic and trigonometric solutions and to check directly which solution (algebraic or trigonometric) is the convenient one (Fettis, 1942; Zucker, 2008).

#### 5.5.5 Analytical solving of quartic equations

We used here the method established by Yacoub and Fraidenraich (2012). For the equation αx^4^ + βx^3^ + γx^2^ + δx + ε = 0, these authors defined (with other notations) the parameters τ_0_, τ_1_, τ_2_ and τ_3_: τ_0_ = -αδ^2^ + β^2^ε, τ_1_ = β^2^δ-4αγδ + 8αβε, τ_2_ = β^2^γ-4αγ^2^ + 2αβδ + 16α^2^ε and τ_3_ = β^3^-4αβγ + 8α^2^δ. If τ_2_ = τ_3_ = 0, then x_1_ = x_2_ = [-β + (3β^2^-8αγ)^1/2^]/(4α) and x_3_ = x_4_ = [-β-(3β^2^-8αγ)^1/2^]/(4α). If τ_2_ ≠ 0 and τ_3_ = 0, then x_1,2_ = -{β + [±2 (β^4^-8β^2^αγ + 16α^2^γ^2^-64α^3^ε)^1/2^ + 3β^2^-8αγ]^1/2^}/(4α) and x_3,4_ = -{β-[±2 (β^4^-8β^2^αγ + 16α^2^γ^2^-64α^3^ε)^1/2^ + 3β^2^-8αγ]^1/2^}/(4α). If τ_2_ ≠ 0 and τ_3_ ≠ 0, then the following cubic equation is to be solved: τ_3_λ^3^ + τ_2_λ^2^ + τ_1_λ + τ_0_ = 0. Yacoub and Fraidenraich consider any of the three possible roots of this cubic equation. We focused here only on its real roots. Are also defined discr3 (or discrIII) = 18τ_3_τ_2_τ_1_τ_0_-4τ_2_^3^τ_0_-27τ_3_^2^τ_0_^2^-4τ_3_τ_1_^3^ + τ_2_^2^τ_1_^2^, μ = (3τ_3_τ_1_-τ_2_^2^)/3τ_3_^2^ and ν = (27τ_3_^2^τ_0_-9τ_3_τ_2_τ_1_ + 2τ_2_^3^)/(27τ_3_^3^). If discr3 < 0, the cubic equation has one real root and two non-real roots. The real root is λ_r_ = [-ν/2 + (μ^3^/27 + ν^2^/4)^1/2^]^1/3^ + [-ν/2-(μ^3^/27 + ν^2^/4)^1/2^]^1/3^-τ_2_/(3τ_3_). If discr3 > 0, the cubic equation has three real roots. Its trigonometric solution is based on φ = arccos [(-ν/2)/(-μ^3^/27)^1/2^]. In this case, λ_trig1,2,3_ = -τ_2_/(3τ_3_) + 2 (-ν/3)^1/2^cos [(φ + 2kπ)/3], where k = 0, 1, 2. For the solving of the quartic equation, Yacoub and Fraidenraich also defined η = [τ_3_/(β + 4αλ_s_)]^1/2^ and ρ = (β^3^-4α^2^δ-2αβγ + 6αβ^2^λ_s_-16α^2^γλ_s_)/(β + 4αλ_s_), where λ_s_ is λ_r_ (if discr3 < 0) or any of the roots λ_trig1,2,3_ (if discr3 > 0). The solutions of the quartic equation are: x_1,2_ = {-β-η±2^1/2^ [ρ + η(β + 4αλ_s_)]^1/2^}/(4α) and x_3,4_ = {-β + η±2^1/2^ [ρ-η(β + 4αλ_s_)]^1/2^}/(4α). These formulae can be computed (in Excel) in a quite accessible way.

## 6 Conclusion

The present study sustains the concept of (non mechanical) covalent constitutional switches based on imine/amine exchanges (transiminations), where, in the presence of a (di)aldehyde, the use of an aromatic amine as first amine (to produce the first (bis-)imine) and of an aliphatic amine as second amine (to produce the second (bis-)imine), makes possible the modulation through alternate additions of acid and base, in the same “pot”. One can also start with an aliphatic amine in the presence of a (di)aldehyde (or with the corresponding (bis-)imine) and go further with the aromatic imine and acid, then with base.

The carbonyl compound that generates the imines is, for the (bis-)imine/amine exchanges in solution, a pyridine-derived (di)aldehyde, which has a good reactivity towards imines. The main aromatic amines used here are aniline-based ones, while the aliphatic ones are primary alkylamines. Trifluoroacetic acid (TFA) and triethylamine (TEA) were used as pH-triggers, the equilibration after addition of acid being particularly fast. The calculated amounts of reagents should sometimes be slightly increased, in order to reach optimal yields. No metal ions are needed to perform the exchanges.

For most dialdehydes investigated here, a general trend seems to be that the ratio between the constant of formation of aldehyde-imine from dialdehyde and amine, and the constant of the subsequent formation of the bis-imine from aldehyde-imine and amine, lies, when the amine is an aliphatic one, between 8 and 13, with an average of 10.5.

For the same kind of substituents, similarly disposed, pyridine-derived aldehydes reacted, with identical amines, generally faster than the corresponding aldehydes derived from benzene.

(Bis-)imines can undergo exchanges with (di)aldehydes as well, and several examples of this kind are reported herein.

In a green chemistry approach, both (bis-)imine/amine and (bis-, tris-)imine/(di)aldehyde exchanges can be implemented under solvent-free conditions. IR spectroscopy was used as an analytical tool for such reactions without solvents.

Successive transiminations can not only be performed in a switch-like, back and forth manner between two (bis-)imines, and triggered by pH changes (Im1→Im2→Im1→Im2→Im1 …), but also from an imine to a second one, then to a third and, finally, to a fourth one, in just one direction (Im1→Im2→Im3→Im4), in a multistep process (the modulation through pH changes being not compulsory). In this last case, the transimination can be associated to a change of the number imine groups per imine-containing molecule, namely, in the example reported here (vide supra, Section 4.3), from 1 to 3, then to 2 and, finally, again to 1. This sequence of three transiminations is based on imine/aldehyde exchanges and was done without solvent.

A solvent-free sequence based on imine/amine exchanges is also reported here: a second imine is obtained from a first one through transimination, and a third one, from the second imine. Upon addition of acid is regenerated the second imine, while subsequent addition of base produces the third imine.

Ordered sets of amines (based on a qualitative estimation of their relative rate of reaction with aldehydes) and aldehydes (based on the formation constants of their imines) were established.

Mathematical models applied to calculate the composition at equilibrium on systems based in a dialdehyde and two competing amines, the excess of amines or the distribution of species as a function of the pH or of the amount of water, lead to systems of polynomial equations, for which analytical or numerical solutions were proposed. Under particular conditions (e.g. excess of amines, strong formation constants), where dialdehyde and aldehyde-imines can practically be seen as consumed, the mathematical treatment can be simplified.

In perspective, such switches could be introduced in more complex structures. The preliminary work on (di)aldehyde/(bis-)imine exchanges, as well as that on mathematical models will be continued. Amongst the perspectives of interest are also a comparison, at equilibrium, between the solvent-free exchanges and those in various solvents, a deep kinetic study of imine/aldehyde exchanges, and the identification of catalysts for such exchanges.

## Data Availability

The original contributions presented in the study are included in the article/Supplementary Material, further inquiries can be directed to the corresponding author.

## References

[B1] Alam M. M. (2011). Template synthesis of new type of macrocyclic molecule derived from Pyridine-2, 6-decarboxaldehyde and 1,2-bis(2-Aminoethoxy) ethane. J. Banglad. Acad. Sci. 35, 61–65. 10.3329/jbas.v35i1.7971

[B2] Alvarez-Santamaría L. Juaristi E. Arroyo-Colín A. B. Palma-Flores J. Cabrera-Rivera F. A. Escalante J. (2019). Efficient solvent-free preparation of imines, and their subsequent oxidation with m-CPBA to afford oxaziridines. Green Sust. Chem. 9, 143–154. 10.4236/gsc.2019.94011

[B3] Ayme J.-F. Beves J. E. Campbell C. J. Leigh D. A. (2019). Probing the dynamics of the imine-based pentafoil knot and pentameric circular helicate assembly. J. Am. Chem. Soc. 141, 3605–3612. 10.1021/jacs.8b12800 30707020 PMC6429429

[B4] Bartulin J. Ramos M. L. Rivas B. L. (1986). Polycondensation of glyoxal with aromatic diamines. Polym. Bull. 15, 405–409. 10.1007/BF00265721

[B5] Belowich M. E. Stoddart J. F. (2012). Dynamic imine chemistry. Chem. Soc. Rev. 41, 2003–2024. 10.1039/C2CS15305J 22310886

[B6] Brotzel F. Chu Y. C. Mayr H. (2007). Nucleophilicities of primary and secondary amines in water. J. Org. Chem. 72, 3679–3688. 10.1021/jo062586z 17411095

[B7] Brown H. C. McDaniel D. H. Häfliger O. (1955). Determination of organic structures by physical methods, 1. New York Academic Press, 567–662.

[B8] Carlson R. Prochazka M. P. Lundstedt T. Westlid K. Lönnberg H. Berg J. E. (1988). Principal properties for synthetic screening: amines. Acta Chem. Scand. B42, 157–165. 10.3891/acta.chem.scand.42b-0157

[B9] Chakma P. Konkolewicz D. (2019). Dynamic covalent bonds in polymeric materials. Angew. Chem. Int. Ed. 58, 9682–9695. 10.1002/anie.201813525 30624845

[B10] Chow C. Fujii S. Lehn J.-M. (2007). Crystallization-driven constitutional changes of dynamic polymers in response to neat/solution conditions. Chem. Commun., 4363–4365. 10.1039/b713413d 17957287

[B11] Ciaccia M. Di Stefano S. (2015). Mechanisms of imine exchange reactions in organic solvents. Org. Biomol. Chem. 13, 646–654. 10.1039/c4ob02110j 25415257

[B12] Ciaccia M. Cacciapaglia R. Mencarelli P. Mandolini L. Di Stefano S. (2013). Fast transimination in organic solvents in the absence of proton and metal catalysts. A keytoimine metathesis catalyzed by primary amines under mild conditions. Chem. Sci. 4, 2253–2261. 10.1039/C3SC50277E

[B13] Ciaccia M. Pilati S. Cacciapaglia R. Mandolini L. Di Stefano S. (2014). Effective catalysis of imine metathesis by means of fast transiminations between aromatic-aromatic or aromatic-aliphatic amines. Org. Biomol. Chem. 12, 3282–3287. 10.1039/C4OB00107A 24733042

[B14] Corbett P. T. Leclaire J. Vial L. West K. R. Wietor J.-L. Sanders J. K. M. (2006). Dynamic combinatorial chemistry. Chem. Rev. 106, 3652–3711. 10.1021/cr020452p 16967917

[B15] Crawford D. Casaban J. Haydon R. Giri N. McNally T. James S. L. (2015). Synthesis by extrusion: continuous, large-scale preparation of MOFs using little or no solvent. Chem. Sci. 6, 1645–1649. 10.1039/C4SC03217A 29308131 PMC5639793

[B16] Crawford D. E. Miskimmin C. K. Cahir J. James S. L. (2017). Continuous multi-step synthesis by extrusion - telescoping solvent-free reactions for greater efficiency. Chem. Commun. 53, 13067–13070. 10.1039/C7CC06010F 29165442

[B200] Crerar D. A. (1975). A method for computing multicomponent chemical equilibria based on equilibrium constants. Geochim. Cosmochim. Acta 39, 1375–1384. 10.1016/0016-7037(75)90116-7

[B84] Dattler D. Fuks G. Heiser J. Moulin E. Perrot A. Yao X. (2020). Design of collective motions from synthetic molecular switches, rotors, and motors. Chem. Rev. 120 (1), 310–433. 10.1021/acs.chemrev.9b00288 31869214

[B17] Deng Y. Zhang Q. Qu D.-H. Tian H. Feringa B. L. (2022). A chemically recyclable crosslinked polymer network enabled by orthogonal dynamic covalent chemistry. Angew. Chem. Int. Ed. 61, e202209100. 10.1002/anie.202209100 35922379 PMC9804754

[B18] Dess D. B. Martin J. C. (1983). Readily accessible 12-I-5oxidant for the conversion of primary and secondary alcohols to aldehydes and ketones. J. Org. Chem. 48, 4155–4156. 10.1021/jo00170a070

[B19] Diez-Castellnou M. Suo R. Marro N. Matthew S. A. L. Kay E. R. (2021). Rapidly adaptive all-covalent nanoparticle surface engineering. Chem. Eur. J. 27, 9948–9953. 10.1002/chem.202101042 33871124 PMC8362155

[B20] Dizdarevic A. Efiana N. A. Phan T. N. Q. Matuszczak B. Bernkop-Schnürch A. (2019). Imine bond formation: a novel concept to incorporate peptide drugs in self-emulsifying drug delivery systems (SEDDS). Eur. J. Pharm. Biopharm. 142, 92–100. 10.1016/j.ejpb.2019.06.002 31176724

[B21] Ferguson M. Giri N. Huang X. Apperley D. James S. (2014). One-pot two-step mechanochemical synthesis: ligand and complex preparation without isolating intermediates. Green Chem. 16, 1374–1382. 10.1039/C3GC42141D

[B22] Fettis H. E. (1942). On various methods of solving cubic equations. Nat. Math. Mag. 17 (3), 117–130. 10.2307/3028120

[B23] French T. C. Bruice T. C. (1964). Rates and equilibrium constants of imine formation with pyridine-4-aldehyde and various aminoacids. Biochem. Biophys. Res. Commun. 15, 403–408. 10.1016/0006-291X(64)90475-9 5827785

[B24] Gambaro S. Talotta C. Della Sala P. Soriente A. De Rosa M. Gaeta C. (2020). Kinetic and thermodynamic modulation of dynamic imine libraries driven by the hexameric Resorcinarene capsule. J. Am. Chem. Soc. 142, 14914–14923. 10.1021/jacs.0c04705 32786766 PMC8010792

[B25] Gibson V. C. Redshaw C. Solan G. A. (2007). Bis(imino)pyridines: surprisingly reactive ligands and a gateway to new families of catalysts. Chem. Rev. 107, 1745–1776. 10.1021/cr068437y 17488059

[B26] Giri A. Shreeraj G. Dutta T. K. Patra A. (2023). Transformation of an imine cage to a covalent organic framework film at the liquid–liquid interface. Angew.Chem. Int. Ed. 62, e202219083. 10.1002/anie.202219083 36912437

[B27] Giuseppone N. Lehn J.-M. (2006). Protonic and temperature modulation of constituent expression by component selection in a dynamic combinatorial library of imines. Chem. Eur. J. 12, 1715–1722. 10.1002/chem.200501038 16402400

[B28] Green R. W. Rogerson M. J. (1968). Schiff base equilibria. VI. n- and t-Butylimines of pyridine-2-aldehyde. Aust. J. Chem. 21, 2427–2431. 10.1071/ch9682427

[B29] Hafezi N. Lehn J.-M. (2012). Adaptation of dynamic covalent systems of imine constituents to medium change by component redistribution under reversible phase separation. J. Am. Chem. Soc. 134, 12861–12868. 10.1021/ja305379c 22783895

[B30] Hall Jr., H. K. (1957). Correlation of the base strengths of amines. J. Am. Chem. Soc. 79, 5441–5444. 10.1021/ja01577a030

[B31] Horwath A. L. Getzen F. W. Maczynska Z. (1995). IUPAC solubility Data series, halogenated methanes with water, vol. 60, 94.

[B32] John R. A. (1995). Pyridoxal phosphate-dependent enzymes. Biochim. Biophys. Acta 1248, 81–96. 10.1016/0167-4838(95)00025-P 7748903

[B33] Kanzian T. Nigst T. A. Maier A. Pichl S. Mayr H. (2009). Nucleophilic reactivities of primary and secondary amines in acetonitrile. Eur. J. Org. Chem. 2009, 6379–6385. 10.1002/ejoc.200900925

[B34] Kolodziejski M. Stefankiewicz A. R. Lehn J.-M. (2019). Dynamic polyimine macrobicyclic cryptands – self-sorting with component selection. Chem. Sci. 10, 1836–1843. 10.1039/C8SC04598D 30842852 PMC6369437

[B35] Krishnamoorthy S. Grubbs R. H. (2020). Aldehyde-functionalized magnetic particles to capture off-target chemotherapeutic agents. ACS Omega 5, 29121–29126. 10.1021/acsomega.0c03840 33225143 PMC7675571

[B36] Lehn J.-M. (2007). From supramolecular chemistry towards constitutional dynamic chemistry and adaptive chemistry. Chem. Soc. Rev. 36, 151–160. 10.1039/B616752G 17264919

[B37] León F. Li C. Reynes J. F. Singh V. K. Lian X. Ong H. C. (2023). Mechanosynthesis and photophysics of colour-tunable photoluminescent group 13 metal complexes with sterically demanding salen and salophen ligands. Faraday Discuss. 241, 63–78. 10.1039/D2FD00117A 36218327

[B38] Leoni L. Carletta A. Fusaro L. Dubois J. Tumanov N. A. Aprile C. (2019). A simple and efficient mechanochemical route for the synthesis of salophen ligands and of the corresponding Zn, Ni, and Pd complexes. Molecules 24, 2314. 10.3390/molecules24122314 31234486 PMC6631197

[B39] Liao Y. Aspin A. Yang Z. (2022). Anaerobic oxidation of aldehydes to carboxylic acids under hydrothermal conditions. RSC Adv. 12, 1738–1741. 10.1039/D1RA08444E 35425195 PMC8979077

[B40] Liu Z. Wang L. Zhou F. (1994). Quantitative structure-free energy relationship for the dehalogenation of halogenated aromatic compounds. Chemosph 29, 1683–1689. 10.1016/0045-6535(94)90315-8

[B41] Lobo M. J. Moratti S. C. Hanton L. R. (2019). A design strategy for single-stranded helicates using pyridine-hydrazone ligands and Pb^II^ . Chem. Asian J. 14 (8), 1184–1193. 10.1002/asia.201801784 30575299

[B42] Madec P.-J. Pérès R. Borgès-Lopès E. Jeanne-Rose V. Lafontaine E. Maréchal E. (1997). Transimination: an efficacious reaction for the synthesis of macrocycles. Macromol. Symp. 122, 137–142. 10.1002/masy.19971220122

[B85] Morgan F. L. C. Beeren I. A. O. Bauer J. Moroni L. Baker M. B. (2024). Structure–reactivity relationships in a small library of imine-type dynamic covalent materials: determination of rate and qquilibrium constants enables model prediction and validation of a unique mechanical softening in dynamic hydrogels. J. Am. Chem. Soc. 146 (40), 27499–27516. 10.1021/jacs.4c08099 39350717 PMC11467966

[B43] Nakada K. Kondo S. Matsumoto Y. Yamanaka M. (2017). Synthesis of a C3-symmetric tris-imine *via* dynamic covalent bond formation between a trialdehyde and a triamine. Tetrahedron Lett. 58, 4612–4616. 10.1016/j.tetlet.2017.10.061

[B44] Nitschke J. R. Schultz D. Bernardinelli G. Gérard D. (2004). Selection rules for helicate ligand component self-assembly: Steric, pH, charge, and solvent effects. J. Am. Chem. Soc. 126, 16538–16543. 10.1021/ja046001z 15600358

[B45] Osowska K. Miljanić O. Š. (2011). Self‐sorting of dynamic imine libraries during distillation. Angew. Chem. Int. Ed. 50, 8345–8349. 10.1002/anie.201102813 21766404

[B86] Palafox M. A. Rastogi V. K. Vats J. K. (2006). 4-Aminobenzonitrile: ab initio calculations, FTIR and Raman spectra. J. Raman Spectrosc. 37 85–99. 10.1002/jrs.1477

[B46] Patel D. C. Higuchi W. I. (1980). Mechanism of cholesterol gallstone dissolution. II. Correlation between the effect of the alkyl amines as cholesterol gallstone dissolution rate accelerators and the degree of binding of the alkyl amines to the bile acid micelles. J. Colloid. Interf. Sci. 74, 211–219. 10.1016/0021-9797(80)90185-x

[B47] Pattillo C. C. Moore J. S. (2019). A tetrahedral molecular cage with a responsive vertex. Chem. Sci. 10, 7043–7048. 10.1039/C9SC02047K 31588271 PMC6676470

[B48] Pérez-Fernández R. Pittelkow M. Belenguer A. M. Sanders J. K. M. (2008). Phase-transfer dynamic combinatorial chemistry. Chem. Commun., 1738–1740. 10.1039/b718075f 18379677

[B49] Rajakumar P. Swaroop M. G. Jayavelu S. Murugesan K. (2006). Synthesis, complexation studies and biological applications of some novel stilbenophanes, indolophanes and bisindolostilbenophanes *via* McMurry coupling. Tetrahedron 62, 12041–12050. 10.1016/j.tet.2006.09.078

[B50] Rowan S. J. Cantrill S. J. Cousins G. R. L. Sanders J. K. M. Stoddart J. F. (2002). Dynamic covalent chemistry. Angew. Chem. Int. Ed. 41, 898–952. 10.1002/1521-3773(20020315)41:6<898::aid-anie898>3.0.co;2-e 12491278

[B51] Schick T. H. G. Rominger F. Mastalerz M. (2020). Examination of the dynamic covalent chemistry of [2 + 3]-Imine cages. J. Org. Chem. 85 (21), 13757–13771. 10.1021/acs.joc.0c01887 32933246 PMC7659045

[B52] Schoustra S. K. Groeneveld T. Smulders M. M. J. (2021). The effect of polarity on the molecular exchange dynamics in imine-based covalent adaptable networks. Polym. Chem. 12, 1635–1642. 10.1039/D0PY01555E

[B53] Schultz D. Nitschke J. R. (2005). Dynamic covalent and supramolecular direction of the synthesis and reassembly of copper(I) complexes. Proc. .Natl. Acad. Sci. U.S.A. 102, 11191–11195. 10.1073/pnas.0502830102 16061815 PMC1183559

[B54] Schultz D. Nitschke J. R. (2006). Designing multistep transformations using the hammett equation: imine exchange on acopper(I) template. J. Am. Chem. Soc. 128, 9887–9892. 10.1021/ja061841u 16866547

[B55] Sharma V. S. Jadeja U. H. Patel R. B. (2017). Study of the molecular structure on liquid crystal properties with reference to thermotropic azoester derivatives. Mol. Cryst. Liq. Cryst. 643, 28–39. 10.1080/15421406.2016.1262681

[B56] Shaw T. E. Shultz L. R. Garayeva L. R. Blair R. G. Noll B. C. Jurca T. (2018). Mechanochemical routes for the synthesis of acetyl- and bis-(imino)pyridine ligands and organometallics. Dalton Trans. 47, 16876–16884. 10.1039/C8DT03608J 30351333

[B57] Shaw T. E. Mathivathanan L. Jurca T. (2019). One-pot, one-step precatalysts through mechanochemistry. Organometallics 38, 4066–4070. 10.1021/acs.organomet.9b00575

[B58] Shaw T. E. Arami J. Ayme J.-F. Lehn J.-M. Jurca T. (2024). Dynamic mechanochemistry: accelerated self-sorting of two imine-based metal complexes under solvent-free mechanochemical conditions. RSC Mechanochem 1, 33–37. 10.1039/D3MR00021D

[B59] Si M. Zhu W. Zhang Y. Barboiu M. Chen J. (2020). Fluorodynamers displaying tunable fluorescence on constitutional exchanges in solution and at solid film–solution interface. Chem. Eur. J. 26, 10191–10194. 10.1002/chem.202000981 32220132

[B60] Singh V. K. Chamberlain-Clay A. Ong H. C. León F. Hum G. Par M. Y. (2021). Multigram mechanochemical synthesis of a salophen complex: a comparative analysis. ACS Sustain. Chem. Eng. 9, 1152–1160. 10.1021/acssuschemeng.0c06374

[B61] Skene W. G. Lehn J.-M. P. (2004). Dynamers: polyacylhydrazone reversible covalent polymers, component exchange, and constitutional diversity. Proc. Natl. Acad. Sci. U. S. A. 22, 8270–8275. 10.1073/pnas.0401885101 15150411 PMC420383

[B62] Soniya K. Awasthi S. Nair N. N. Chandra A. (2019). Transimination reaction at the active site of aspartate aminotransferase: a proton hopping mechanism through pyridoxal 5′-Phosphate. ACS Catal. 9 (7), 6276–6283. 10.1021/acscatal.9b00834

[B63] Stadler A.-M. (2013). Modulation of the selectivity of schiff base formation in mixtures of two NH2 compounds and one aldehyde or of two aldehydes and one amine. Isr. J. Chem. 53, 113–121. 10.1002/ijch.201300003

[B64] Stadler A.-M. Lehn J.-M. P. (2014). Coupled nanomechanical motions: metal ion-effected, pH-modulated, simultaneous extension/contraction motions of double domain helical/linear molecular strands. J. Am. Chem. Soc. 136 (9), 3400–3409. 10.1021/ja408752m 24547897

[B65] Suzuki S. Sakaki S. Ishizuka S. Nishino T. Ito H. Nonaka R. (2018). Efficient Solvent- and catalyst-free syntheses of imine derivatives applying the pressure reduction technique: remarkable change of the reaction rate with the phase transition. Green Sust. Chem. 8, 167–179. 10.4236/gsc.2018.82012

[B66] Suzuki S. Ito H. Ishizuka S. Nonaka R. Noike M. Kodama T. (2019). Perfect Solvent- and catalyst-free syntheses of imine derivatives using the pressure reduction technique. Green Sust. Chem. 9, 105–118. 10.4236/gsc.2019.94008

[B67] Tao Y. Liu T. Yang X. Murphy J. G. (2021). Kinetics and products of the aqueous phase oxidation of triethylamine by OH. ACS Earth Space Chem. 5 (8), 1889–1895. 10.1021/acsearthspacechem.1c00162

[B68] Teipel J. Gottstein V. Hölzle E. Kaltenbach K. Lachenmeier D. W. Kuballa T. (2022). An easy and reliable method for the mitigation of deuterated chloroform decomposition to stabilise susceptible NMR samples. Chemistry 4, 776–785. 10.3390/chemistry4030055

[B69] Ulrich S. Buhler E. Lehn J.-M. (2009). Reversible constitutional switching between macrocycles and polymers induced by shape change in a dynamic covalent system. New J. Chem. 33, 271–292. 10.1039/b817261g

[B70] Vitaku E. Dichtel W. R. (2017). Synthesis of 2D imine-linked covalent organic frameworks through formal transimination reactions. J. Am. Chem. Soc. 139, 12911–12914. 10.1021/jacs.7b06913 28853570

[B71] Wanasinghe S. V. Dodo O. J. Konkolewicz D. (2022). Dynamic bonds: adaptable timescales for responsive materials. Angew. Chem. Int. Ed. 61, e202206938. 10.1002/anie.202206938 36167937 PMC10092857

[B72] Wang J. Ganguly R. Yongxin L. Díaz J. Soo H. S. Garcí F. (2016). A multi-step solvent-free mechanochemical route to indium(iii) complexes. Dalton Trans. 45, 7941–7946. 10.1039/C6DT00978F 27112317

[B73] Wang L.-M. Yue J.-Y. Cao X. Wang D. (2019). Insight into the transimination process in the fabrication of surface Schiff-based covalent organic frameworks. Langmuir 35, 6333–6339. 10.1021/acs.langmuir.9b00565 31002521

[B74] Wang X.-L. Zhang L.-T. He S. Chen X.-X. Huang X.-C. Zhou H.-L. (2023). Dynamic imine exchange reactions for facile synthesis of imine-linked covalent organic frameworks. Chem. Mater. 35, 10070–10077. 10.1021/acs.chemmater.3c02092

[B75] Yacoub M. D. Fraidenraich G. (2012). 96.33 A solution to the quartic equation. Math. Gaz. 96 (536), 271–275. 10.1017/S002555720000454X

[B76] Yang Z. Lehn J.-M. (2020). Dynamic covalent self-sorting and kinetic switching processes in two cyclic orders: macrocycles and macrobicyclic cages. J. Am. Chem. Soc. 142, 15137–15145. 10.1021/jacs.0c07131 32809804

[B77] Ye W. Seneviratne U. I. Chao M.-W. Ravindra K. C. Wogan G. N. Tannenbaum S. R. (2012). Transimination of quinone imines: a mechanism for embedding exogenous redox activity into the nucleosome. Chem. Res. Toxicol. 25, 2627–2629. 10.1021/tx3004517 23194336 PMC3525013

[B78] You L. (2023). Dual reactivity based dynamic covalent chemistry: mechanisms and applications. Chem. Commun. 59, 12943–12958. 10.1039/D3CC04022D 37772969

[B79] Zentner C. A. Anson F. Thayumanavan S. Swager T. M. (2019). Dynamic imine chemistry at complex double emulsion interfaces. J. Am. Chem. Soc. 141, 18048–18055. 10.1021/jacs.9b06852 31674769

[B80] Zhou X. Tan Y. H. Finch J. A. (2018). Effect of pH and time on hydrodynamic properties of dodecylamine. Physicochem. Probl. Min. Process. 54, 1237–1244. 10.5277/ppmp18165

[B81] Zou H. Hai Y. Ye H. You L. (2019). Dynamic covalent switches and communicating networks for tunable multicolor luminescent systems and vapor-responsive materials. J. Am. Chem. Soc. 141, 16344–16353. 10.1021/jacs.9b07175 31547653

[B82] Zucker I. J. (2008). 92.34 the cubic equation - a new look at the irreducible case. Math. Gaz. 92 (524), 264–268. 10.1017/S0025557200183135

[B83] Zuo S. Zheng S. Liu J. Zuo A. (2022). Mechanochemical synthesis of unsymmetrical salens for the preparation of Co–salen complexes and their evaluation as catalysts for the synthesis of α-aryloxy alcohols *via* asymmetric phenolic kinetic resolution of terminal epoxides. Beilstein J. Org. Chem. 18, 1416–1423. 10.3762/bjoc.18.147 36300012 PMC9577384

