# Supplementary material for: Transfer of structural units through imine exchanges, in solution or without solvent: successive transiminations, stimuli (pH)-modulated covalent switches, and mathematical models
Source: Front Chem. 2026 Apr 13;13:1241625. doi: 10.3389/fchem.2025.1241625 (PMC13112204; doi:10.3389/fchem.2025.1241625)
Supplement: Supplementary file 1 [file Supplementaryfile1.zip › Supplementary Material/g-S1-Num-expl.pdf]

A numerical example – general approach

- starting from equilibrium constants and initial concentrations -

In this example, one considers the dialdehyde A and the amine B, the equilibrium constants K_1 (formation of AB from A and B) and K_2 (formation of AB_2 from AB and B), as well the initial concentrations of A and B. One wishes to calculate the composition at equilibrium starting from initial concentrations of A and B (step 1)and the excess (or supplementary amount) of B needed to reach a desired yield of AB_2 (step 2), as well as the composition at equilibrium after addition of the excess of B (step 3). To the last reaction mixture one adds a known amount of amine C and one wishes to calculate the composition at equilibrium (step 4), the excess of C needed to reach a desired yield of AC_2 (with respect to A; step 5) and the composition at equilibrium after addition of the excess of C (step 6). Equilibrium constants K_3 (formation of AC from A and C), K_4 (formation of AC_2 from AC and C) and K_5 (formation of ABC from AB and C) are supposed to be known. Finally, if the acidity constants of protonated amines BH^+ (K_6) and CH^+ (K_7) are known, then one can calculate and draw distribution curves where concentrations are functions of the pH (step 7).

It is assumed that the reagents are added in small volumes of solution, the volume of the reaction mixture is seen as constant and that water comes only from the initial amount (if any) and from reactions.

To simplify and approximate, we considered here a case where the formation constants of imines $K_1 = 10^3$, $K_2 = 0.14x10^3$, $K_3 = 16.5x10^3$, $K_4 = 2.35x10^3$, $K_5 = 4.75x10^3$ are in or close to the order of magnitude of those associated to reactions of 2,6-pyridinedicarboxaldehyde with 4-(hexyloxy)aniline and decylamine, respectively in chloroform, and $K_6 = 10^{-5.07}$ and $K_7 = 10^{-10.6}$ are average acidity constants in water (because we could not find those for chloroform). The values of the concentrations presented hereafter and obtained from calculations were simply and conveniently rounded.

Step 1 (paper -> section 5.1.2). Composition at equilibrium of a mixture "A+2B". Equilibrated reaction mixture 1. One considers a mixture of dialdehyde A and aromatic amine B, with the initial concentrations (before formation of any imine) in the reaction mixture $[A]_{in1} = a_1 = 6.20$ mM, $[B]_{in1} = b_1 = 12.40$ mM (2 equiv. of B with respect to A) and $[H_2O]_{in1} = w_1 = 0$. On this basis, one can calculate the concentrations at equilibrium: $[AB_2]_{eq1} = 5.52$mM (89%), $[AB]_{eq1} = 0.67$mM, $[A]_{eq1} = 0.01$mM, $[B]_{eq1} = 0.69$mM and $[H_2O]_{eq1} = 11.71$mM. See SM (= the Supplementary Material), the Excel file "g-S1-St1-comp-after-add-B".

Step 2 (paper -> section 5.1.3). Calculation of the excess of amine B. In order to increase the yield of AB_2, one should now calculate the required excess of B and one may express it as a concentration, g_B. Let the desired yield be of to 98.2%. We obtain $g_B/a_1 = 0.756$ and we retain, to simplify, the down rounded value 0.75, which gives $g_B = 0.75a_1 = 4.65$mM. See SM, the Excel file "g-S1-St2-calc-exc-B".

Step 3 (paper -> section 5.1.2). Composition at equilibrium after addition of the excess of B. Equilibrated reaction mixture 2. With the excess g_B, the initial concentrations before formation of imines are now $[A]_{in2} = a_1$, $[B]_{in2} = b_2 = b_1+g_B = 17.05$mM (2.75 equiv.) and $[H_2O]_{in2} = w_1$, and they lead to the concentrations at equilibrium: $[AB_2]_{eq2} = 6.09$mM, $[AB]_{eq2} = 0.11$mM, $[A]_{eq2} = 2.9\cdot10^{-4}$mM, $[B]_{eq2}$

= 4.76mM and $[H_2O]_{eq2}$ = 12.29mM. We have $[AB_2]_{eq2}/a$ = 0.982. See SM, the Excel file "g-S1-St3-comp-after-add-exc-B".

Step 4 (paper -> section 5.2.2.1). Composition at equilibrium after addition of C to the previous mixture. Equilibrated reaction mixture 3. To this equilibrated mixture, one adds aliphatic amine C, firstly, as much as the total amount (expressed as a concentration) of B (free amine and imines). The new initial concentrations before formation of imines are now $[A]_{in3} = a_1$, $[B]_{in3} = b_2$, $[H_2O]_{in3} = w_1$ and $[C]_{in3} = c_1 = b_2$, and they lead to the following calculated concentrations at equilibrium: $[AC_2]_{eq3}$ = 4.68mM (about 75-76%), $[AC]_{eq3} = 3.9 \cdot 10^{-3}$mM, $[ABC]_{eq3}$ = 1.41mM, $[AB_2]_{eq3}$ = 0.10mM, $[AB]_{eq3} = 5.9 \cdot 10^{-4}$mM, $[A]_{eq3} = 4.7 \cdot 10^{-7}$mM, $[B]_{eq3}$ = 15.43mM, $[C]_{eq3}$ = 6.27mM and $[H_2O]_{eq3}$ = 12.40mM. See SM, the Excel files "g-S1-St4-comp-after-add-C" and "g-S1-St4-comp-after-add-C-altern".

Step 5 (paper -> section 5.2.3.1). Calculation of the excess of amine C. One can now calculate the supplementary amount (excess) g_C (expressed as a concentration) of amine C, that can lead to a desired yield of bis-imine AC_2, bigger than the yield from step 4. Let this yield be of 90%. We have g_C/a_1 = 2.15 or g_C = 13.33mM. See SM, the Excel files: "g-S1-St5-calc-suppl-amt-C" and "g-S1-St5-calc-suppl-amt-C-altern".

Step 6 (paper -> section 5.2.2.1). Composition at equilibrium after addition of the excess of C. Equilibrated reaction mixture 4. With this excess g_C of amine C, the new initial concentrations before formation of imines are $[A]_{in4} = a_1$, $[B]_{in4} = b_2$, $[H_2O]_{in4} = w_1$ and $[C]_{in4} = c_2 = c_1+g_C$ = 30.38mM (4.9 equiv. with respect to A). The calculated concentrations at equilibrium are $[AC_2]_{eq4}$ =5.58mM (90%), $[AC]_{eq4} = 1.6 \cdot 10^{-3}$mM, $[ABC]_{eq4}$ = 0.60mM, $[AB_2]_{eq4} = 1.6 \cdot 10^{-2}$mM, $[AB]_{eq4} = 8.5 \cdot 10^{-5}$mM, $[A]_{eq4} = 6.4 \cdot 10^{-8}$mM (practically 0), $[B]_{eq4}$ = 16.42mM, $[C]_{eq4}$ = 18.62mM and $[H_2O]_{eq4}$ = 12.40mM. $[AC_2]_{eq4}/a$ = 0.9. See SM, the Excel files: "g-S1-St6-comp-after-add-exc-C" and "g-S1-St6-comp-after-add-exc-C-altern".

Step 7 (paper -> section 5.3). Influence of the pH. Concentration versus pH distribution curves have been calculated and plotted on the basis of composition at equilibrium calculated for each natural value of the pH from 0 to 14. See SM, the Excel files: "g-S1-St7-pH0" to "g-S1-St7-pH14" and "g-S1-St7-pH-crv".

In our Excel files, for the solving of cubic equations with three distinct roots (discriminant $\neq$ 0), we systematically write the formula of the sole real root associate to a discriminant less than 0, together with the formulae of the three real roots associated to a discriminant greater than 0.

For the solving of quartic equations we used the method established by Yacoub and Fraidenraich (Yacoub, M. D., and Fraidenraich, G. (2012). A solution to the quartic equation. *Math. Gaz*. 96 (536), 271-275. doi:10.1017/S002555720000454X). For the equation $\alpha x^4 + \beta x^3 + \gamma x^2 + \delta x + \varepsilon = 0$, one has to define the parameters τ_0, τ_1, τ_2 and τ_3: $\tau_0 = -\alpha\delta^2+\beta^2\varepsilon$, $\tau_1 = \beta^2\delta-4\alpha\gamma\delta+8\alpha\beta\varepsilon$, $\tau_2 = \beta^2\gamma-4\alpha\gamma^2+2\alpha\beta\delta+16\alpha^2\varepsilon$ and $\tau_3 = \beta^3-4\alpha\beta\gamma+8\alpha^2\delta$. If $\tau_2 \neq 0$ and $\tau_3 \neq 0$, then the following cubic equation is to be solved: $\tau_3\lambda^3+\tau_2\lambda^2+\tau_1\lambda+\tau_0 = 0$. In our excel files, for the solving of this cubic equation with three distinct roots (discriminant $\neq$ 0), we systematically write the formula of the sole real root associate to a discriminant less than 0, together with the formula of one of the three real roots associated to a discriminant greater than 0.

When numerically solving the equations in Excel, we sought to obtain a value as close as possible to 0 of the left-hand side of the equation, which may lead to solutions with numerous (sometimes more than 8) digits after the decimal separator. However, in practice such solutions should be rounded (to ones with only 2-3 digits after the decimal separator), considering also the uncertainty associated to the unknowns (concentrations).

In our Excel files, the decimal separator is a comma.
