# Supplementary material for: Transfer of structural units through imine exchanges, in solution or without solvent: successive transiminations, stimuli (pH)-modulated covalent switches, and mathematical models
Source: Front Chem. 2026 Apr 13;13:1241625. doi: 10.3389/fchem.2025.1241625 (PMC13112204; doi:10.3389/fchem.2025.1241625)
Supplement: Supplementary file 1 [file Supplementaryfile1.zip › Supplementary Material/s-S1-S2-Num-expl.pdf]

A numerical example - simplified approach

- starting from equilibrium constants and concentrations before reaction -

In this example, one starts from a mixture of 1 equiv. of bis-imine AB_2 ($[AB_2]_{bfr} = 6.20x10^{-3}M$) with an excess of 0.75 equiv. of amine B ($[B]_{bfr} = 4.65x10^{-3}M$). The mixture is treated with 4.9 equiv. of amine C ($[C]_{bfr} = 30.38x10^{-3}M$). The molar concentrations $[AB_2]_{bfr}$, $[B]_{bfr}$, $[C]_{bfr}$, $[ABC]_{bfr}$ and $[AC_2]_{bfr}$ are those in the reaction mixture before the reaction of AB_2 with C takes place; one notices that, here, $[ABC]_{bfr} = [AC_2]_{bfr} = 0$. These concentrations before reaction correspond to the following initial concentrations (before formation of any imine): $[A]_{in} = 6.20x10^{-3}M$, $[B]_{in} = 17.05x10^{-3}M$ and $[C]_{in} = 30.38x10^{-3}M$. In this example, B is an aliphatic amine and C, an aromatic one. All these initial concentrations correspond to those calculated in the steps 5 and 6 of the numerical example from the general approach (see SM, files "g-S1-Num-expl", "g-S1-St5-calc-suppl-amt-C", "g-S1-St6-comp-after-add-exc-C").

It is assumed that the amounts of dialdehyde A and of intermediates of type aldehyde-amine AB and AC can be seen as negligible (no water in the reaction mixture or, if water, the constants and/or excess of amines are enough high to minimize the formation of A, AB and AC through hydrolysis). Now, instead of the equilibriums $A + B \rightleftharpoons AB + H_2O$ (K_1), $AB + B \rightleftharpoons A_2B + H_2O$ (K_2), $A + C \rightleftharpoons AC + H_2O$ (K_3), $AC + C \rightleftharpoons AC_2 + H_2O$ (K_4) and $AB + C \rightleftharpoons ABC + H_2O$ (K_5), we consider only the equilibriums $AB_2 + C \rightleftharpoons ABC + B$ ($K_I = K_5/K_2$) and $ABC + C \rightleftharpoons AC_2 + B$ ($K_{II} = K_3K_4/(K_1K_5)$).

Starting from concentrations before reaction of AB_2, B and C, and from the corresponding equilibrium constants, one wishes to calculate, in this simplified approach, the composition at equilibrium after exchange (section 1), and to compare the composition at equilibrium obtained in the simplified approach with that obtained in the general approach (section 2). For the simplified approach, one also wishes to establish pH-dependent distribution curves (section 3) and to calculate the composition at equilibrium after addition of acid (section 4), then of base (section 5).

<u>1. Composition at equilibrium after exchange (paper -> section 5.4.1).</u> We have $K_1 = 10^3$, $K_2 = 0.14x10^3$, $K_3 = 16.5x10^3$, $K_4 = 2.35x10^3$, $K_5 = 4.75x10^3$ (which are in or close to the order of magnitude of those of reactions of 2,6-pyridinedicarboxaldehyde with 4-(hexyloxy)aniline and decylamine in chloroform) and $K_I = K_5/K_2$ and $K_{II} = K_3K_4/(K_1K_5)$ (the values of K_I and K_{II} in Excel were, in this case, for the sake of convenience, not rounded). To a mixture of AB_2 and B was added C. We consider the above concentrations before reaction. The composition at equilibrium is, according to the simplified approach (see SM, the Excel file "s-S1-comp-equil"): $[AB_2]_{eq} = 1.57x10^{-5}M$, $[ABC]_{eq} = 6.03x10^{-4}M$, $[AC_2]_{eq} = 5.58x10^{-3}M$, $[B]_{eq} = 1.64x10^{-2}M$, $[C]_{eq} = 1.86x10^{-2}M$.

<u>2. Comparison between general and simplified approach.</u> For the purposes of comparison between the general approach and the simplified one, we consider, for the same set of concentrations before reaction ($[AB_2]_{bfr} = 6.20x10^{-3}M$, $[B]_{bfr} = 4.65x10^{-3}M$, $[C]_{bfr} = 30.38x10^{-3}M$, to which correspond the initial concentrations $[A]_{in} = 6.20x10^{-3}M$, $[B]_{in} = 17.05x10^{-3}M$ and $[C]_{in} = 30.38x10^{-3}M$), two sets of equilibrium constants, namely the set S1 ($K_1 = 10^3$, $K_2 = 0.14x10^3$, $K_3 = 16.5x10^3$, $K_4 = 2.35x10^3$, $K_5 = 4.75x10^3$) and the S2 ($K_1 = 10$, $K_2 = 1.4$, $K_3 = 16$, $K_4 = 2.4$, $K_5 = 4.8$), to which we applied both general and simplified approaches. For each of the two sets of constants, we

compare the general approach with the simplified one. For the general approach, the values of the ratio $[H_2O]_{in}/[A]_{in}$ are -1.9, -1, 0, 4, 10, 25 and 50.

(a) Set S1. Starting from the above concentrations before reaction and formation constants, in the case of compounds AB_2, AC_2, ABC, B and C, at equilibrium, the percent change (with respect to c_{gen}) between their concentrations calculated through the general approach (c_{gen}) and those calculated through the simplified one ($c_{simplif}$), namely $100|c_{gen}-c_{simplif}|/c_{gen}$, lies between $3.8x10^{-5}$ % and 1.1% (see SM, files "c-S1-perc-chg-f-water", "c-S1-perc-chg-f-water-graph"). The concentrations at equilibrium of compounds containing CHO groups are, in the general approach, $[A]_{eq} < 4.3x10^{-8}$M, $[AB]_{eq} < 2.2x10^{-6}$M and $[AC]_{eq} < 4.1x10^{-5}$M (see SM, file "c-S1-perc-chg-f-water"). These species do not appear in the simplified approach.

(b) Set S2. For the same concentrations before reaction, but where K_1-K_5 are considerably lower, i.e. $K_1 = 10$, $K_2 = 1.4$, $K_3 = 16$, $K_4 = 2.4$, $K_5 = 4.8$, the percent change between the concentrations of species AB_2, AC_2, ABC, B and C at equilibrium calculated through the general approach (c_{gen}) and those calculated through the simplified one ($c_{simplif}$), namely $100|c_{gen}-c_{simplif}|/c_{gen}$, lies between 0.1 % and 0.9 % for $[H_2O]_{in}/[A]_{in} = -1.9$ (see SM, file "c-S2-perc-chg-f-water"). For the same constants and concentrations before reaction, the percent change lies however between 1% and 9% for $[H_2O]_{in}/[A]_{in} = -1$ and between about 2% and 630% for $[H_2O]_{in}/[A]_{in}$ = 0, 4, 10 and 50 (see SM, file "c-S2-perc-chg-f-water").

The constants from the set S2 were obtained from those from the set S1 through division by 10^2 or 10^3.

(c) These comparative results (see SM, files "c-S1-perc-chg-f-water", "c-S2-perc-chg-f-water") show that the two approaches – the general and the simplified ones – lead to equivalent results only under specific conditions (enough high formation constants and/or excesses of amines or relatively low amounts of water).

(d) In the general approach, $[H_2O]_{in} < 0$ means that a part of the amount of water that forms in the reaction, leaves the system during the equilibration; $[H_2O]_{eq}$ will however be positive. $[H_2O]_{in}/[A]_{in}$ is close to -2 (limit value) when, for example, one starts from anhydrous AB_2 that is reacted with anhydrous C in an anhydrous solvent (rather ideal conditions).

In the general approach, if the concentrations at equilibrium of species A, AB and AC are, when they appear in the denominator, very close to 0, then the division through these concentrations could operate in a way similar to the division through 0. In such cases, the recalculation of constants K_1-K_5 (useful to check the numerical solutions) may not work properly.

3. pH-dependent distribution curves - simplified approach (paper -> section 5.4.2). For constants $K_I = K_5/K_2$ and $K_{II} = K_3K_4/(K_1K_5)$ obtained from the set S1 ($K_1 = 10^3$, $K_2 = 0.14x10^3$, $K_3 = 16.5x10^3$, $K_4 = 2.35x10^3$ and $K_5 = 4.75x10^3$), for acidity constants of BH^+ ($K_6 = 10^{-5.07}$) and CH^+ ($K_7 = 10^{-10.6}$) and for concentrations before reaction $[AB_2]_{bfr} = 6.20x10^{-3}$M, $[B]_{bfr} = 4.65x10^{-3}$M and $[C]_{bfr} = 30.38x10^{-3}$M, we calculated the distribution curves of species AB_2, ABC, AC_2, B and C as functions of the pH (see SM, files "s-S1-pH", "s-S1-pH-crv"). The same was done for the constants from the set S2 ($K_1 = 10$, $K_2 = 1.4$, $K_3 = 16$, $K_4 = 2.4$, $K_5 = 4.8$; see SM, files "s-S2-pH", "s-S2-pH-crv"). The calculations were done only for natural values from the pH interval [0, 14].

Given that pK_a values of acids and protonated amines are not available for all solvents and that the values we found are for water, we however used, in this example, for the sake of simplicity, the values for water, although the formation constants of imines are based on those we determined in $CDCl_3$. One also notices that chloroform usually contains traces of water.

4. Addition of acid - simplified approach (paper -> section 5.4.3). For the set S1 of formation constants and concentrations before reaction (namely $K_I = K_5/K_2$ and $K_{II} = K_3K_4/(K_1K_5)$, where $K_1 = 10^3$, $K_2 = 0.14x10^3$, $K_3 = 16.5x10^3$, $K_4 = 2.35x10^3$ and $K_5 = 4.75x10^3$), was calculated the composition at equilibrium (including the pH) after addition of 4.9 equiv. of HX (here, TFA) to the equilibrated reaction mixture from $[AB_2]_{bfr} = 6.20x10^{-3}M$, $[B]_{bfr} = 4.65x10^{-3}M$ and $[C]_{bfr} = 30.38x10^{-3}M$. $K_8 = K_a(TFA) = 10^{-pKa} = 10^{-0.52}$. See SM, file "s-S1-aj-acid". The yield of target bis-imine AB_2, namely $100[AB_2]_{eq}/[AB_2]_{bfr}$ is of about 98%.

5. Addition of base - simplified approach (paper -> section 5.4.4). Further, we calculated the composition at equilibrium after addition of 4.9 equiv. of base (here, TEA) to the previous equilibrated reaction mixture. $K_9 = K_a(BaseH^+) = 10^{-10.75}$. See SM, file "s-S1-aj-base". The yield of target bis-imine AC_2, namely $100[AC_2]_{eq}/[AB_2]_{bfr}$ is of about 81%.

For the solving of cubic equations with three distinct roots (discriminant $\neq 0$), in our Excel files we systematically write the formula of the sole real root associate to a discriminant less than 0, together with the formulae of the three real roots associated to a discriminant greater than 0.

For the solving of quartic equations we used the method established by Yacoub and Fraidenraich (Yacoub, M. D., and Fraidenraich, G. (2012). A solution to the quartic equation. *Math. Gaz*. 96 (536), 271-275. doi:10.1017/S002555720000454X). For the equation $\alpha x^4 + \beta x^3 + \gamma x^2 + \delta x + \varepsilon = 0$, one has to define the parameters τ_0, τ_1, τ_2 and τ_3: $\tau_0 = -\alpha\delta^2+\beta^2\varepsilon$, $\tau_1 = \beta^2\delta-4\alpha\gamma\delta+8\alpha\beta\varepsilon$, $\tau_2 = \beta^2\gamma-4\alpha\gamma^2+2\alpha\beta\delta+16\alpha^2\varepsilon$ and $\tau_3 = \beta^3-4\alpha\beta\gamma+8\alpha^2\delta$. If $\tau_2 \neq 0$ and $\tau_3 \neq 0$, then the following cubic equation is to be solved: $\tau_3\lambda^3+\tau_2\lambda^2+\tau_1\lambda+\tau_0 = 0$. In our excel files, for the solving of this cubic equation with three distinct roots (discriminant $\neq 0$), we systematically write the formula of the sole real root associate to a discriminant less than 0, together with the formula of one of the three real roots associated to a discriminant greater than 0.

When numerically solving the equations in Excel, we sought to obtain a value as close as possible to 0 of the left-hand side of the equation, which may lead to solutions with numerous (sometimes more than 8) digits after the decimal separator. However, in practice such solutions should be rounded (to ones with only 2-3 digits after the decimal separator), considering also the uncertainty associated to the unknowns (concentrations).

In our Excel files, the decimal separator is a comma.
