# Supplementary material for: Transfer of structural units through imine exchanges, in solution or without solvent: successive transiminations, stimuli (pH)-modulated covalent switches, and mathematical models
Source: Front Chem. 2026 Apr 13;13:1241625. doi: 10.3389/fchem.2025.1241625 (PMC13112204; doi:10.3389/fchem.2025.1241625)

Supplementary material

General considerations 2
Synthesis (**P2**, **P3**, **P4**, **M**, **A**, **AR3$_2$**, **AR4$_2$**, **AL3$_2$**, **triim**) 3
NMR spectra of compound **P2** 5
NMR spectra of compound **P3** 11
NMR spectra of compound **P4** 19
NMR spectra of compound **M** 26
NMR spectra of compound **A** 31
NMR spectra of compound **R1** 43
NMR spectra of compound **R2** 50
NMR spectra of compound **R4** 56
NMR spectra of compound **AR3$_2$** 62
NMR spectra of compound **AR4$_2$** 73
NMR spectra of compound **AL3$_2$** 88
NMR spectra of compound **triim** 99
ESI-MS spectra 101
^{1}H NMR DOSY spectra for reactions 109
IR spectra 128
Competition between two monoaldehydes 129
Transimination-switches 130

Commercially available chemicals were used as received, without further purification. $CDCl_3$ was usually filtered on basic alumina. The following compounds were prepared according to procedures described in the literature: 2-phenyl-4,6-pyrimidinedicarboxaldehyde (Schmitt, J.-L., Stadler, A.-M., Kyritsakas, N., Lehn, J.-M., *Helv. Chim. Acta*, 2003, *86*, 1598-1624), 3-hydroxy-2-pyridinecarboxaldehyde (Tretyakov, E. V., Eltsov, I. V., Fokin, S. V., Shvedenkov, Y. G., Romanenko, G. V., Ovcharenko, V. I., *Polyhedron*, 2003, *22*, 2499-2514), the trialdehyde **triald** (Rajakumar, P., Swaroop, M. G., Jayavelu S., Murugesan K., *Tetrahedron*, 2006, *62*, 12041-12050). NMR spectra were collected on 400 or 500 MHz Bruker spectrometers. IR spectra (by ATR) were recorded on a Nicolet IS50 FR-IR spectrometer.

Abbreviations for the multiplicity of NMR signals: s = singlet, d = doublet, t = triplet, dd = doublet of doublets, ddd = doublet of doublet of doublets, m = multiplet. The chemical shifts are given in parts per million (ppm). Other abbreviations: py = pyridine, r.t. = room temperature.

In ^{1}H NMR spectra, chemical shifts are shown with 3 digits after the decimal point and described with 2 digits after the decimal point. In ^{13}C NMR spectra, chemical shifts are shown with 2 digits after the decimal point and described with 1 digit after the decimal point. δ_{ref} for ^{13}C NMR spectra in $CDCl_3$ is 77.36 ppm on spectra and it is rounded to 77.4 ppm in the description of spectra.

Description of cross peaks from COSY, ROESY, HSQC and HMBC spectra

For ^{1}H-^{13}C HSQC and ^{1}H-^{13}C HMBC

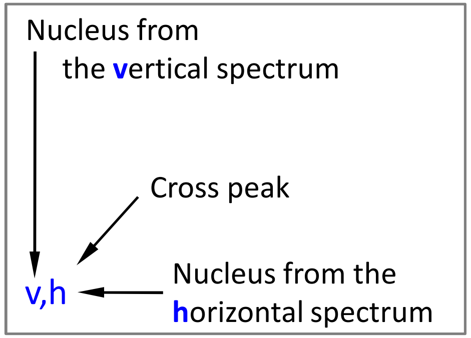

For ^{1}H-^{13}C HSQC

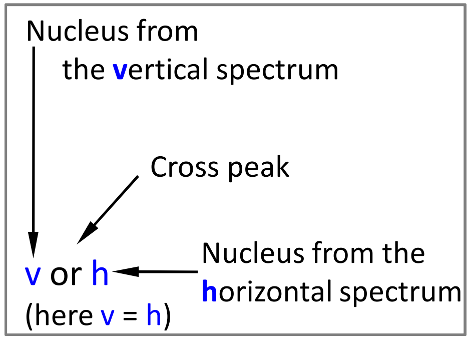

One or more protons of the same kind, and the carbon atoms bound to these protons have, in the case of the compounds from this work, the same number. For simplification, the corresponding HSQC cross peak has just one number, which is the same as that of H and C atoms. For example, one writes simply *a* instead of (*a*,*a*) or (C_a,H_a)).

For ^{1}H-^{1}H COSY and ^{1}H-^{1}H ROESY

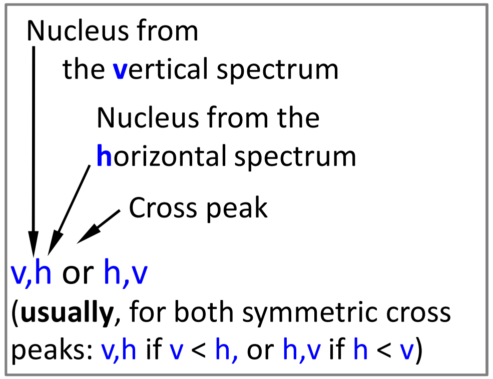

<u>Compound **P2** (6-hydrazineylpyridin-2-yl)methanol</u>

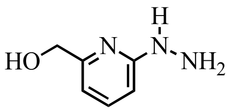

6-bromo-2-(hydroxymethyl)pyridine (866 mg) was refluxed for 48h in hydrazine hydrate (16 mL). The hydrazine hydrate was removed using a rotary evaporator. Dichloromethane (30 mL) was added to the solid residue, the mixture was stirred for 2 minutes at r.t., then CH_2Cl_2 was removed using a rotary evaporator (this operation was repeated three times). The solid residue was dissolved in dichloromethane (50 mL) and solid $NaHCO_3$ (1 g) was added to the mixture, which was stirred for 30 minutes. After filtration, the solvent was removed and yielded a grey solid (577 mg, 90 %). ^{1}H NMR (400 MHz, $CDCl_3$, δ_{ref} = 7.26 ppm): 7.49 (dd, 1H, py, J = 8.1 Hz, J = 7.4 Hz), 6.63-6.57 (m, 2H, py), 5.92 (s, broad, 1H, OH), 4.62 (s, 2H, CH_2), 3.80 (s, broad, 3H, $HNNH_2$) ppm. ^{13}C NMR (125 MHz $CDCl_3$, δ_{ref} = 77.4 ppm): 161.0, 157.5, 138.7, 111.1, 105.4, 64.2 ppm. ESI-TOF MS (m/z): $[C_6H_{10}ON_3+H]^+$ calculated 140.0818, found 140.0812.

<u>Compound **P3**</u>

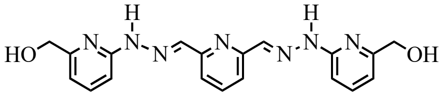

A solution of 2,6-pyridinedicarboxaldehyde (251 mg, 1.85 mmol) in chloroform (15 mL) was added to a solution of (6-hydrazineylpyridin-2-yl)methanol (574 mg, 4.12 mmol) in 100 mL methanol. The mixture was stirred at r.t. for 48 hours. The precipitate was separated by centrifugation, washed with chloroform and dried under vacuum to afford diol **P3** as a solid (610 mg, 87%). ^{1}H NMR (500 MHz, DMSO-d_6, δ_{ref} = 2.50 ppm): 11.21 (s, 2H, NH), 8.02 (s, 2H), 7.87-7.78 (m, 3H), 7.68 (t, J = 7.8 Hz, 2H), 7.17 (d, J = 8.2 Hz, 2H) 6.92 (d, J = 7.3 Hz, 2H), 5.33 (t, J = 5.8 Hz, 2H), 4.45 (d, J = 5.8 Hz, 4H) ppm. ^{13}C NMR (125 MHz DMSO-d_6, δ_{ref} = 39.5 ppm). 160.5, 155.9, 154.0, 138.8, 138.6, 136.9, 117.9, 111.7, 104.6, 64.1 ppm. ESI-TOF MS (m/z): $[C_{19}H_{19}O_2N_7+H]^+$ calculated 378.1673 found 378.1685; $[C_{19}H_{19}O_2N_7+2H]^{2+}$ calculated 189.5873 found 189.5880.

<u>Compound **P4**</u>

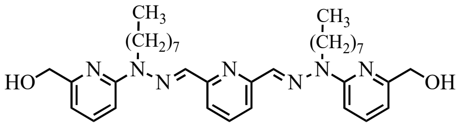

Diol **P3** (780 mg, 2.06 mmol) was dissolved in DMF (25 mL) and produced a yellow solution. To this solution was added a solution of KOH (247 mg, 4.4 mmol) in methanol (3 mL), which produced a red solution. To this red mixture was added 1-bromooctane (3.7 mg, 19 mmol). The mixture was stirred at 30°C for 16 h, then at 50°C for 4h. To the reaction mixture cooled at r.t., was added water (70 mL). The mixture was filtered. The solid was dried, than dissolved in chloroform (90 mL) and washed two times with water (10 mL), then with brine (50 mL). The organic phase was dried on magnesium sulfate. Chloroform was removed using a rotary evaporator. Flash chromatography on alumina, eluent $CHCl_3$. Yield 500 mg (41%). ^{1}H NMR (500 MHz, $CDCl_3$, δ_{ref} = 7.26 ppm): 7.94 (d, 2H, J = 7.8 Hz), 7.82 (s, 2H), 7.74 (t, 1H, J = 7.8 Hz), 7.62-7.61 (m, 4H), 6.74 (dd, 2H, J = 3.2 Hz, J = 4.8 Hz), 4.70 (d, 4H, C**H**$_2$OH, J = 4.8 Hz), 4.34 ("t", 4H, J = 5.0 Hz), 3.66 (t, 2H, OH, J = 4.8 Hz), 1.72 (qui, 4H, J = 7.7 Hz), 1.46-1.22 (m, 20H), 0.88 (t, 6H, J = 7.0 Hz) ppm. ^{13}C NMR (125 MHz, $CDCl_3$, δ_{ref} = 77.4 ppm): 156.8, 156.6, 155.3, 138.6, 136.9, 134.3, 118.6, 112.3, 108.7, 64.050, 42.5, 32.2, 29.8, 29.6, 27.6, 25.4, 23.0, 14.5 ppm. ESI-TOF MS (m/z): $[C_{35}H_{51}N_7O_2+H]^+$ calculated 602.4177, found 602.4169; $[C_{35}H_{51}N_7O_2+2H]^{2+}$ calculated 301.7122, found 301.7125.

Synthesis

Compounds **A** and **M**

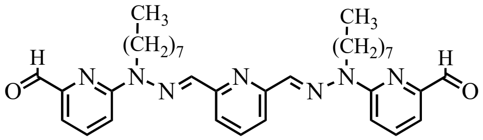

A

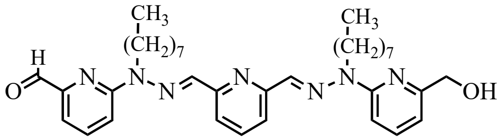

M

A solution of Dess-Martin periodinane (1.155 g, 2.72 mmol) in dichloromethane (50 mL) was added to a solution of diol **P4** (365 mg, 0.61 mmol) in dichloromethane (115 mL) and the mixture was stirred for 9 hours at r.t. To this mixture, was added saturated solution of $NaHCO_3$ (110 mL), then $Na_2S_2O_3$ (4.3 g, 27 mmol in 20 ml water) and dichloromethane (50 mL). The mixture was stirred for 30 minutes at r.t. The organic phase was separated and the solvent was removed with using a rotary evaporator. Flash chromatography on alumina, eluent CH_2Cl_2. Yield: 266 mg dialdehyde (73%) and 55 mg monoaldehyde (15%).

Dialdehyde **A**. ^{1}H NMR (400 MHz $CDCl_3$, δ_{ref} =7.26 ppm): 9.97 (d, 2H, *J* = 0.6 Hz), 7.95 (dd, 2H, *J* = 8.5 Hz, *J* = 0.7 Hz), 7.95 (d, 2H, *J* = 7.8 Hz), 7.87 (s, 2H), 7.77 (ddd, 2H, *J* = 8.5 Hz, *J* = 7.5 Hz, *J* = 0.6 Hz), 7.76 (t, 1H, *J* = 7.8 Hz), 7.51 (dd, 2H, *J* = 7.2 Hz, *J* = 0.8 Hz), 4.44 ('t', 4H, *J* = 7.6 Hz), 1.75 (quintuplet, 4H, *J* = 7.6 Hz), 1.50-1.35 (m, 8H), 1.35-1.20 (m, 12H), 0.88 (t, 6H, *J* = 7.0 Hz) ppm. ^{13}C NMR (125 MHz, $CDCl_3$, δ_{ref} = 77.4 ppm): 194.1, 157.8, 155.1, 150.9, 138.5, 136.9, 135.0, 118.8, 114.9, 114.4, 42.3, 32.2, 29.6, 29.6, 27.3, 25.1, 23.0, 14.4 ppm. ESI-TOF MS (m/z): $[C_{35}H_{47}N_7O_2+H]^+$ calculated 598.3864, found 598.3648; $[C_{35}H_{47}N_7O_2+Na]^+$ calculated 620.3668, found 620.3683.

Monoaldehyde **M**. ^{1}H NMR (400 MHz $CDCl_3$, δ_{ref} = 7.26 ppm): 9.97 (d, *J* = 0.6 Hz, 1H), 7.97-7.90 (o, 3H), 7.87 (s, 1H), 7.83 (s, 1H), 7.80-7.71 (m, 2H), 7.65 - 7.58 (m, 2H), 7.50 (dd, *J* = 7.2 Hz, *J* = 0.7 Hz, 1H), 6.77-6.72 (m, 1H), 4.70 (d, *J* = 4.6 Hz, 2H), 4.43 ('t', *J* = 7.5 Hz, 2H), 4.34 ('t', *J* = 7.6 Hz, 2H), 3.63 (t, *J* = 4.7 Hz, 1H), 1.79-1.67 (m, 4H), 1.49-1.20 (m, 20H), 0.88 (m, 6H) ppm. ^{13}C NMR (125 MHz $CDCl_3$, δ_{ref} = 77.4 ppm): 194.2, 157.8, 156.8, 156.7, 155.4, 155.0, 150.9, 138.6, 138.4, 136.9, 135.1, 134.2, 118.7, 118.6, 114.9, 114.4, 112.4,108.6, 64.0, 42.5, 42.3, 32.2, 32.1, 29.7, 29.6, 29.6, 29.3, 27.5, 27.3, 25.3, 25.2, 23.0, 23.0, 14.4, 14.4 ppm. ESI-TOF MS (m/z): $[C_{35}H_{49}N_7O_2+H]^+$ calculated 600.4010, found 600.4021; $[C_{35}H_{49}N_7O_2+Na]^+$ calculated 622.3827, found 622.3840.

Compounds **AR3**$_2$, **AR4**$_2$ and **AL3**$_2$

Synthesis of bis-imine **AR3**$_2$. A solution of dialdehyde **A** (20.83 mg, 34.8 mmol, 1 equiv.) and amine **R3** (*p*-toluidine, 12.2 mg, 111.9 mmol, 3.2 equiv.) in chloroform (3 mL; chloroform was stored on $NaCO_3$ and filtered through basic alumina) was stirred at 20°C for 16 hours. The solvent was removed to 0.30 mL, then acetonitrile (5 mL) was added, the mixture was centrifuged and the solid product was washed thrice with acetonitrile (centrifugation and elimination of supernatant). The solid product was dried under vacuum. Yield: 21 mg (77%).

Synthesis of bis-imine **AR4**$_2$. A solution of dialdehyde **A** (23.6 mg, 39.5 mmol, 1 equiv.) and amine **R4** (15.2 mg, 79 mmol, 2 equiv.) in chloroform (3.5 mL; chloroform was stored on $NaCO_3$ and filtered through basic alumina) was stirred at 20°C for 16 hours. The solvent was removed until to 1 mL, then acetonitrile (10 mL) was added, the mixture was centrifuged and the solid product was washed thrice with acetonitrile (centrifugation and elimination of supernatant). The solid product was dried under vacuum. Yield: 30.5 mg (81%).

Synthesis of bis-imine **AL3**$_2$. In a 5-mm-diameter NMR tube were mixed dialdehyde **A** (3 mg, 5 mmol, 1 equiv.) and amine **L3** (hepthylamine, 1.1 mg, 11 mmol, 2 equiv.) with 0.47 mL of $CDCl_3$ (filtered through basic alumina). After 3 hours at 22°C, the reaction was almost quantitative (yield > 97% based on the ^{1}H NMR spectrum).

Compound **triim**

Trialdehyde **triald** (6.37mg, 1 equiv.) was mixed with *n*-octadecylamine (10.9mg, 3.05 equiv.) and the mixture was heated, in a small vial with a good cap, at 85°C for 18h, then at 95°C for 4h, to produce the compound trim (yield > 95%). The compound was used without further purification.

NMR spectra of compound

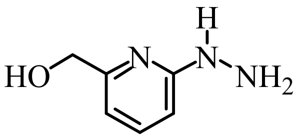

P2

^{1}H NMR spectrum (400 MHz, $CDCl_3$, δ_{ref} = 7.26 ppm) of compound **P2**

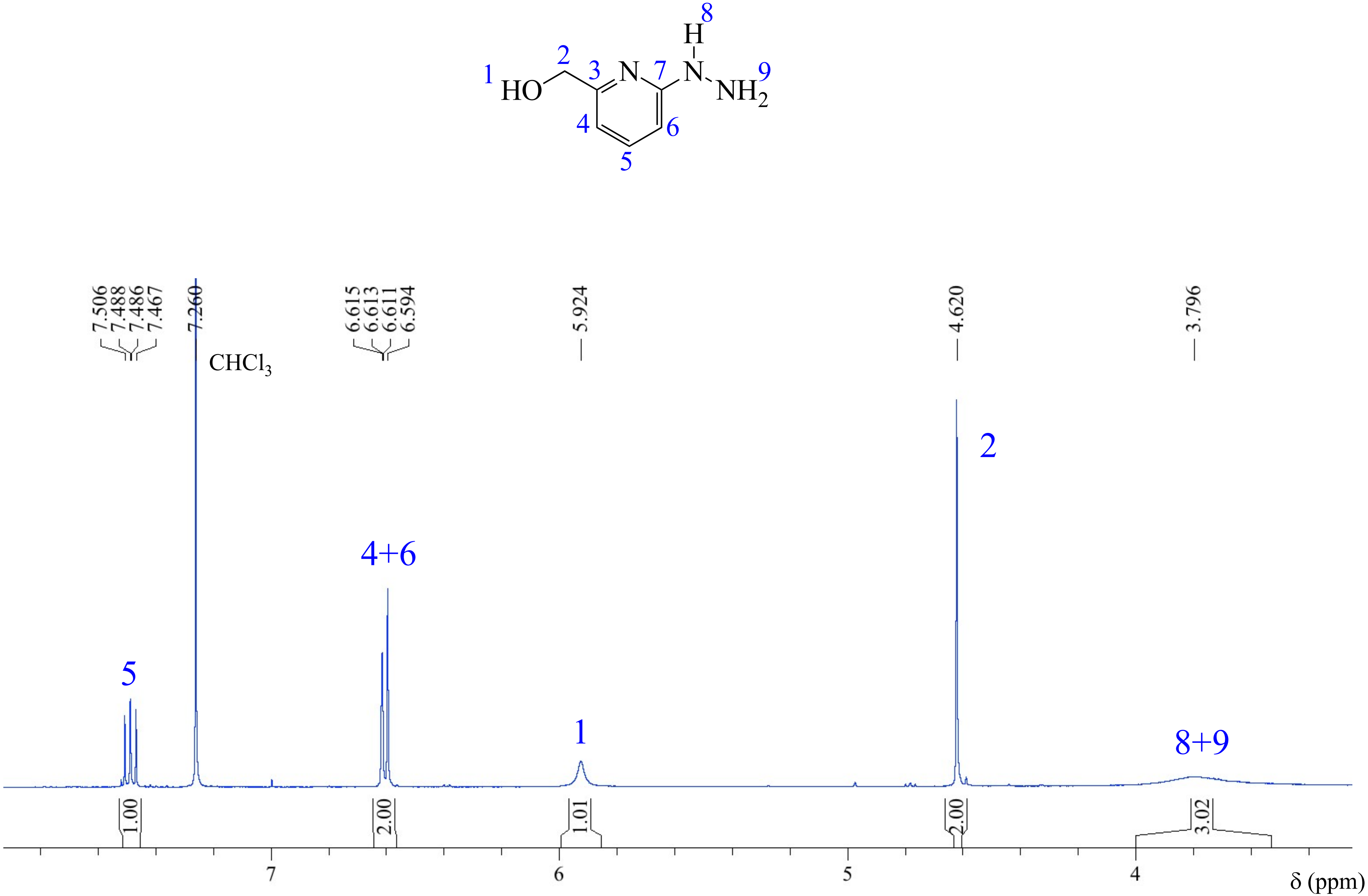

^{13}C NMR spectrum (125 MHz, $CDCl_3$, δ_{ref} = 77.4 ppm) of compound **P2**

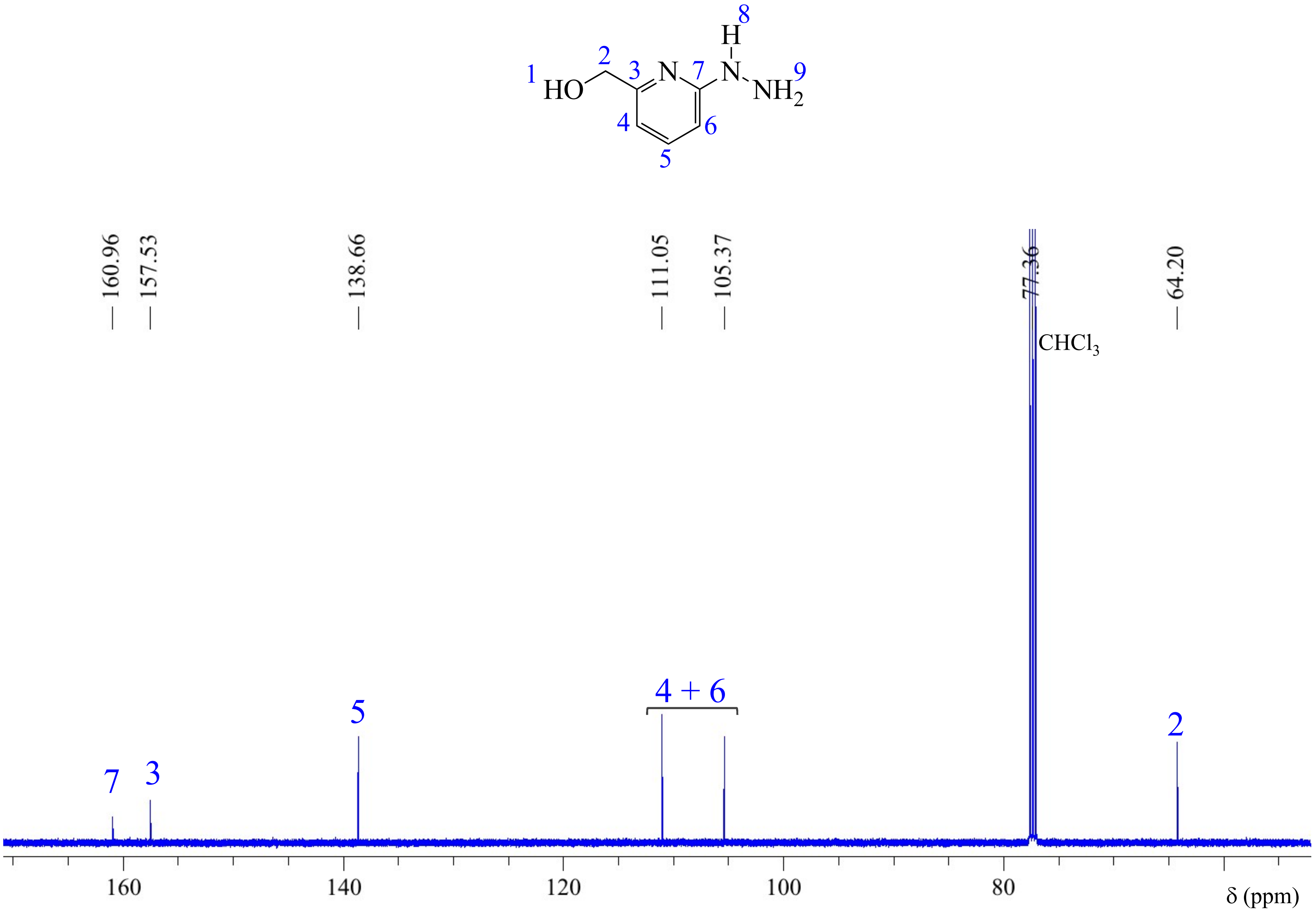

^{1}H-^{1}H COSY NMR spectrum (500 MHz, $CDCl_3$) of compound **P2**

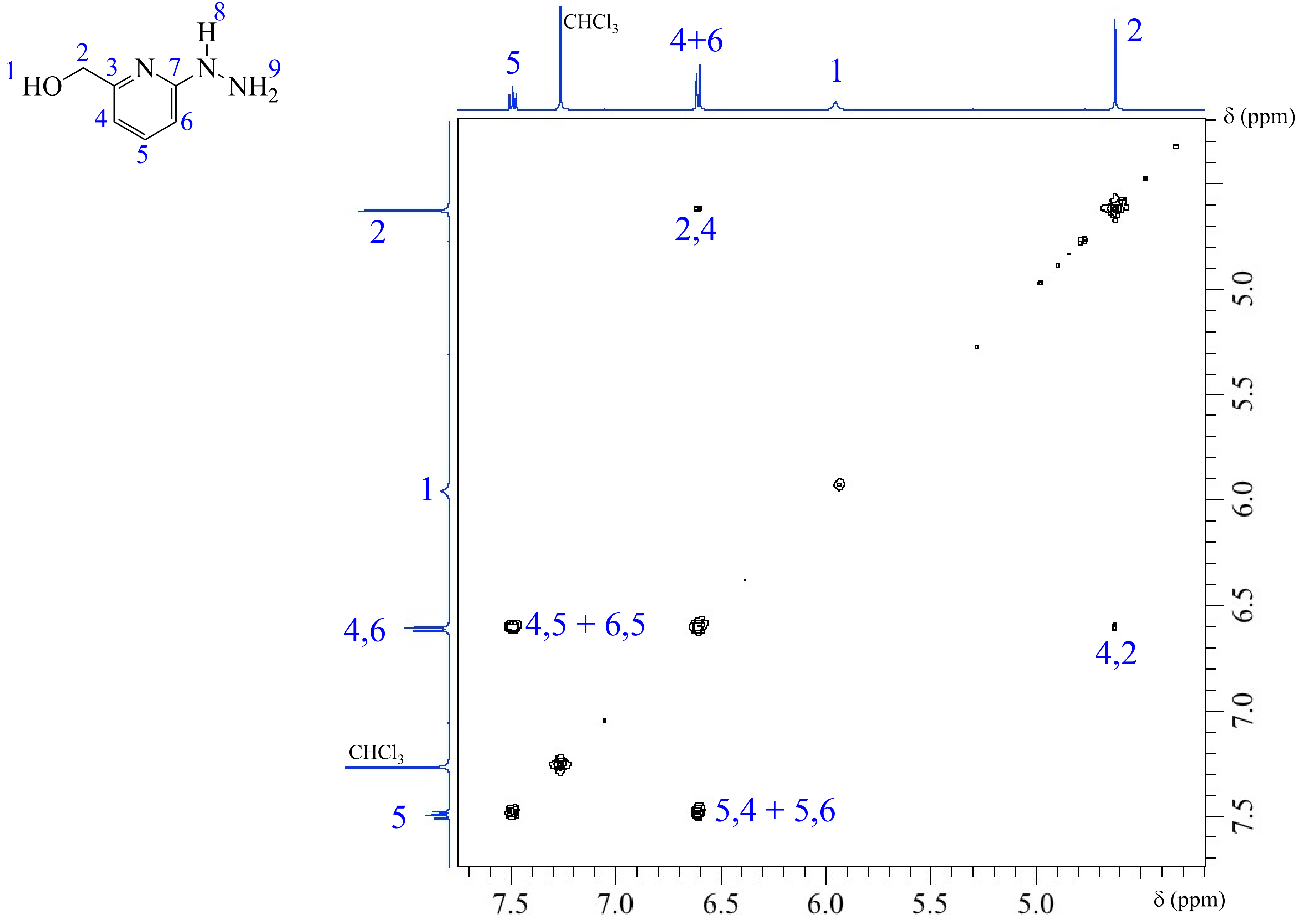

^{1}H-^{13}C HSQC NMR spectrum (500 MHz, $CDCl_3$) of compound **P2**

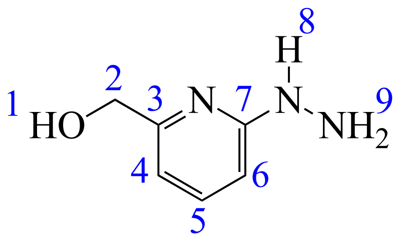

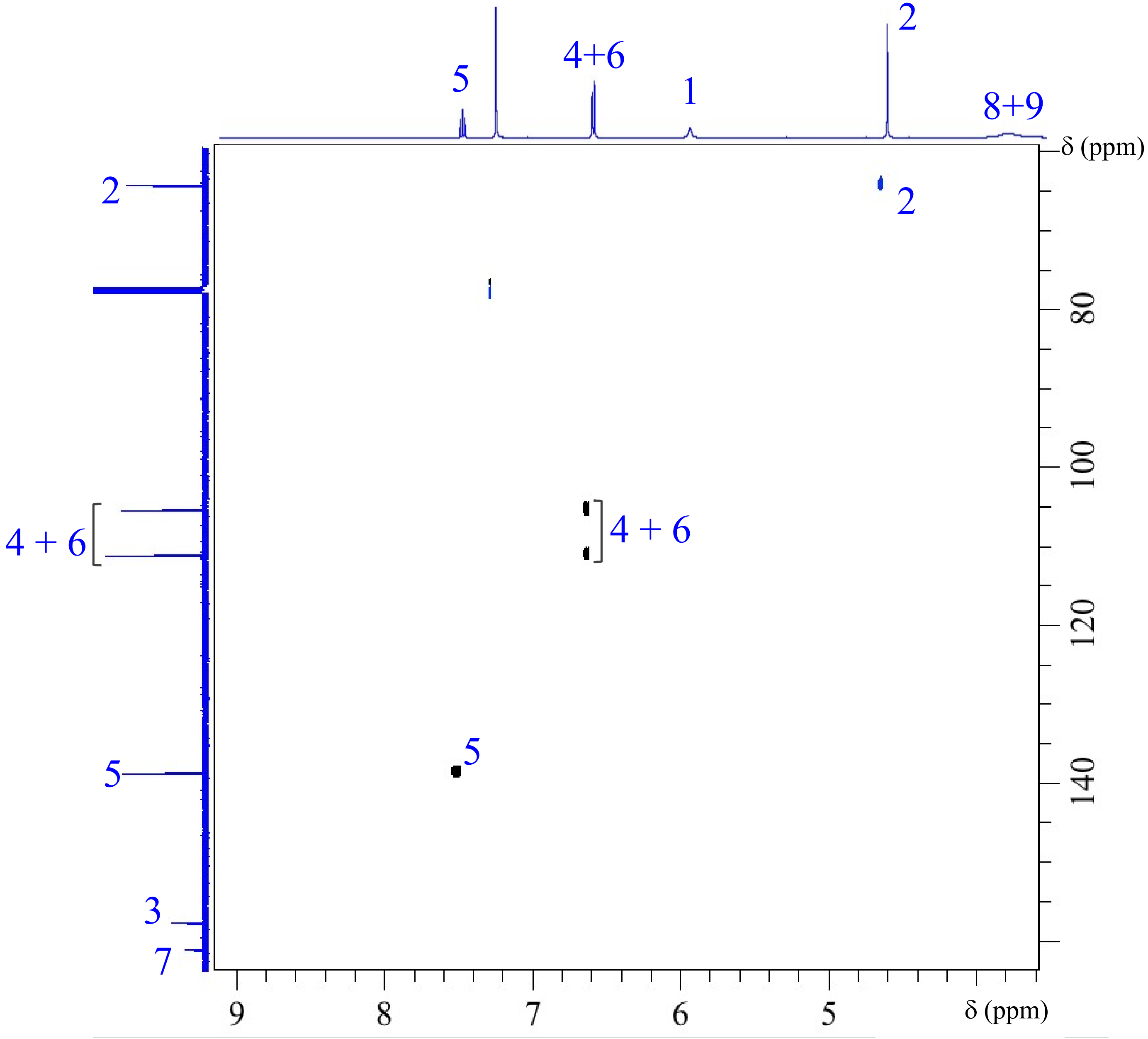

^{1}H-^{13}C HMBC NMR spectrum (500 MHz, $CDCl_3$) of compound **P2**

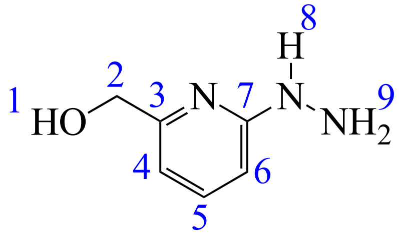

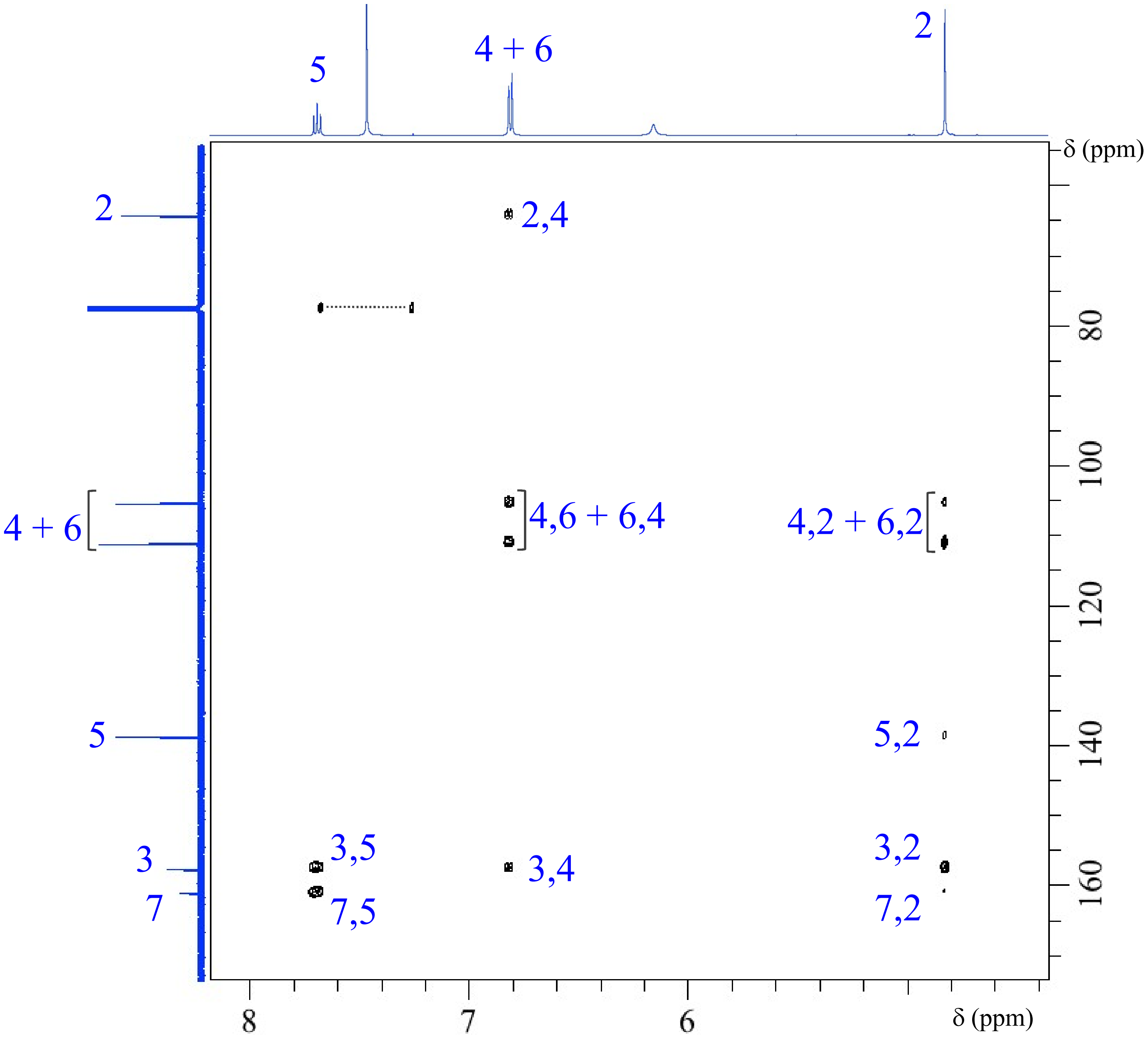

NMR spectra of compound

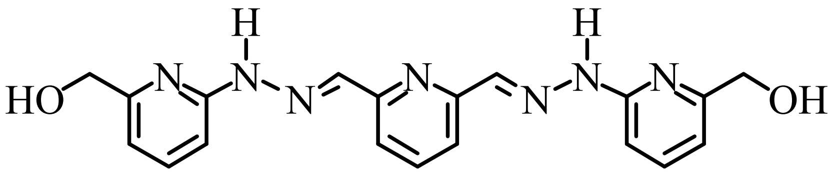

P3

^{1}H NMR spectrum (500 MHz, DMSO-d$_6$, δ_{ref} = 2.50 ppm) of compound **P3**

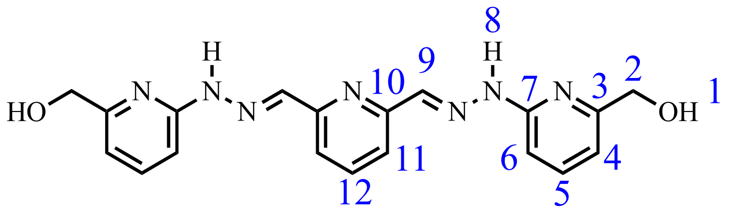

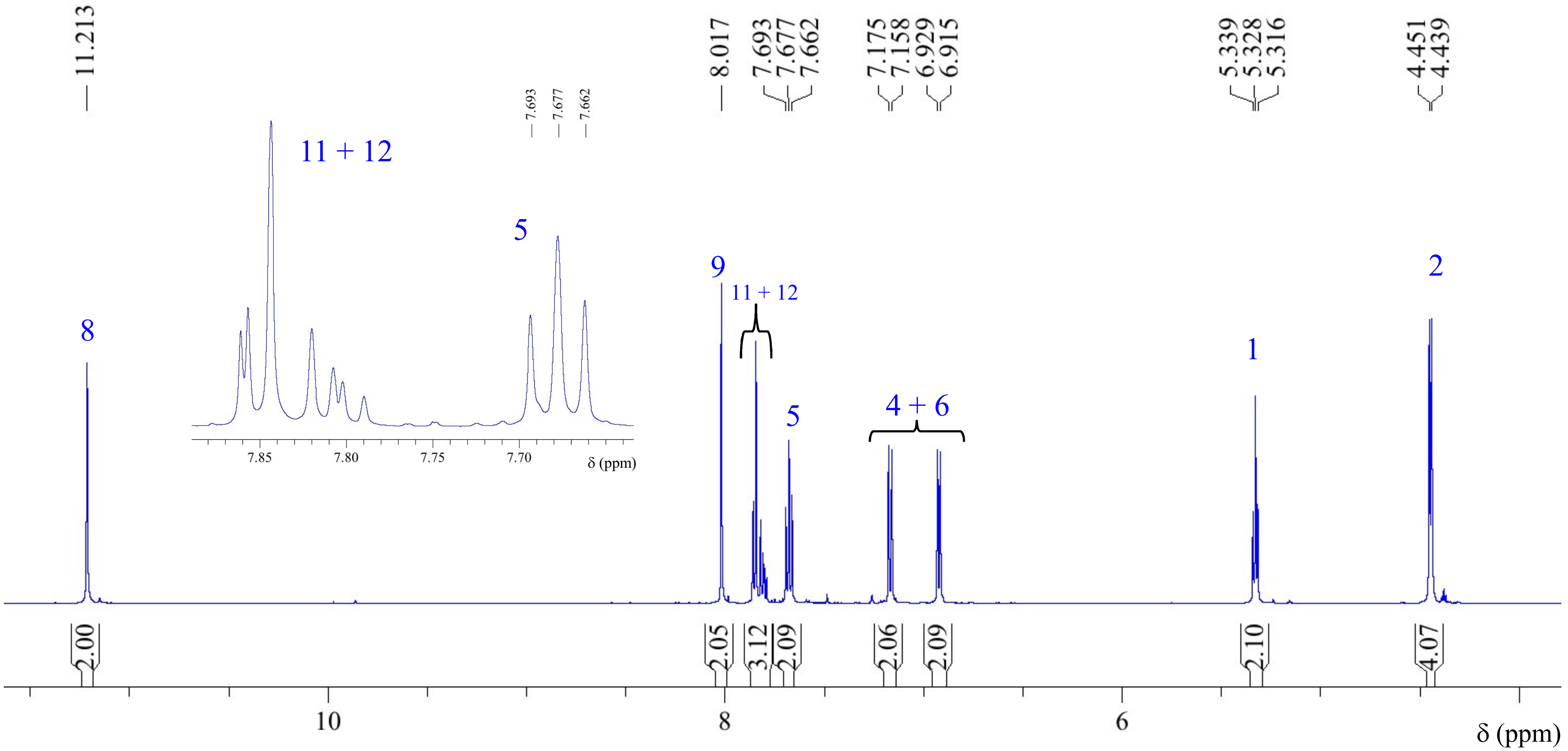

^{13}C NMR spectrum (125 MHz, DMSO-d_6, δ_{ref} = 39.5 ppm) of compound **P3**

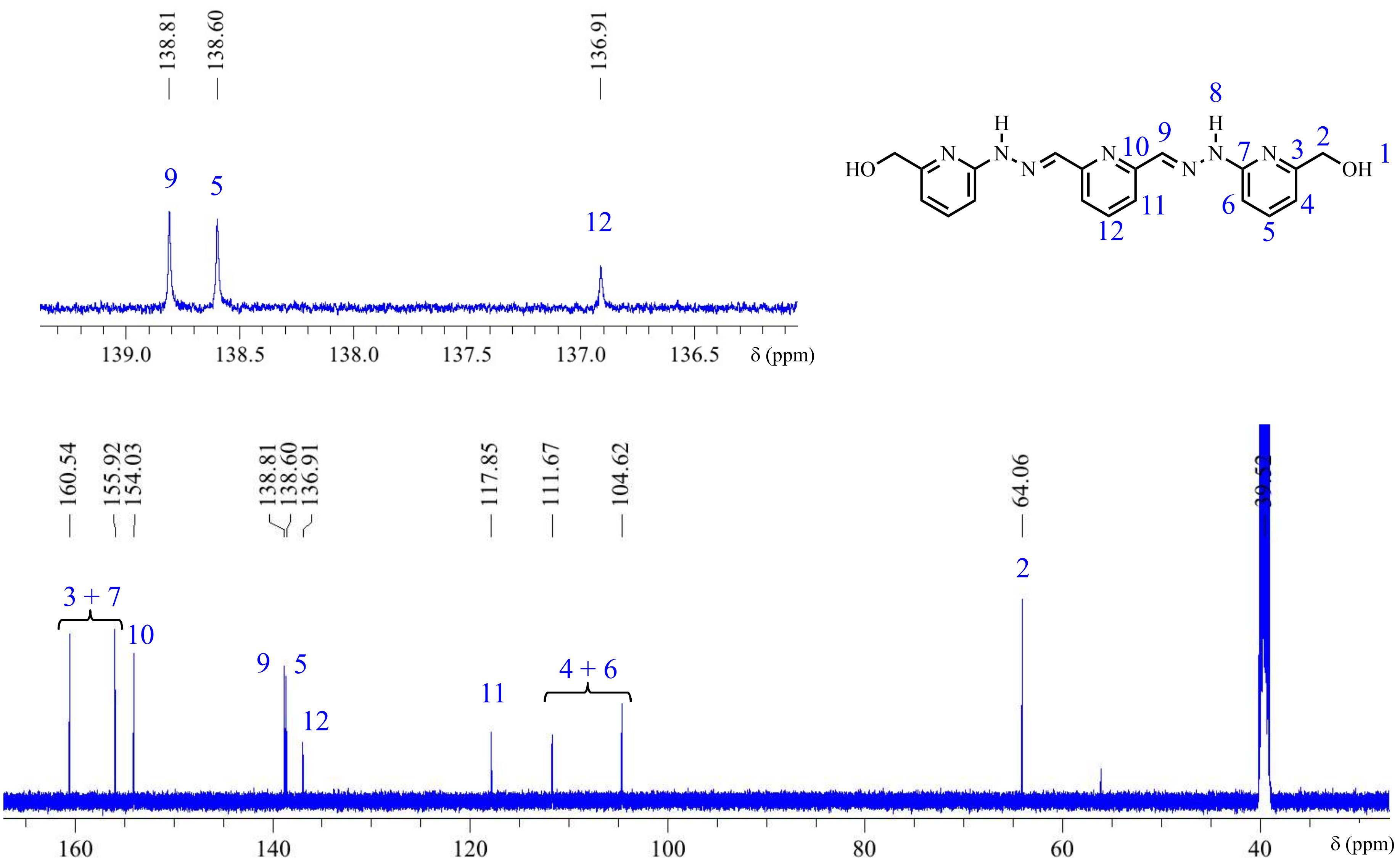

^{1}H-^{1}H COSY NMR spectrum (500 MHz, DMSO-d_6) of compound **P3**

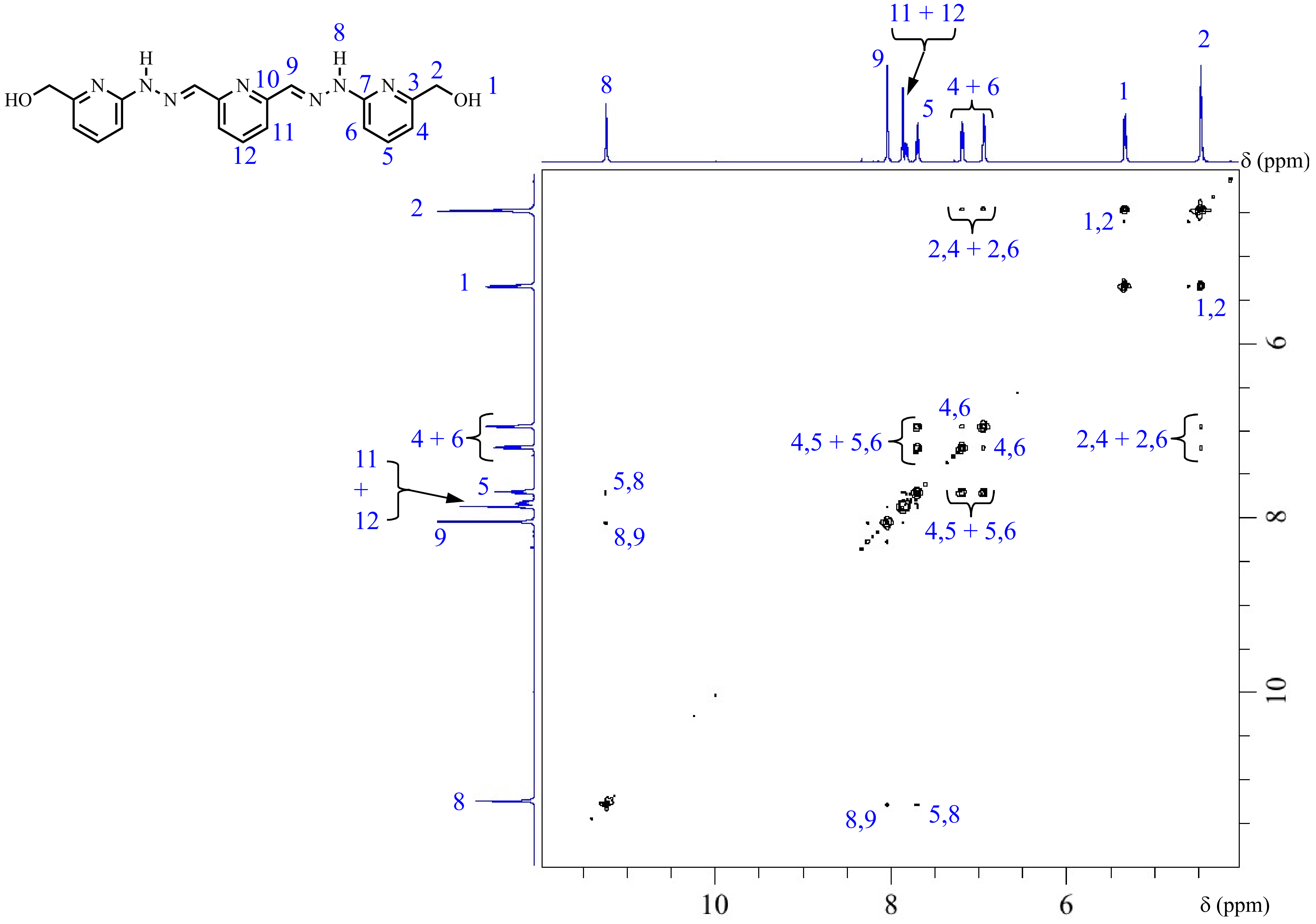

Part of the ^{1}H-^{1}H COSY NMR spectrum (500 MHz, DMSO-d_6) of compound **P3**

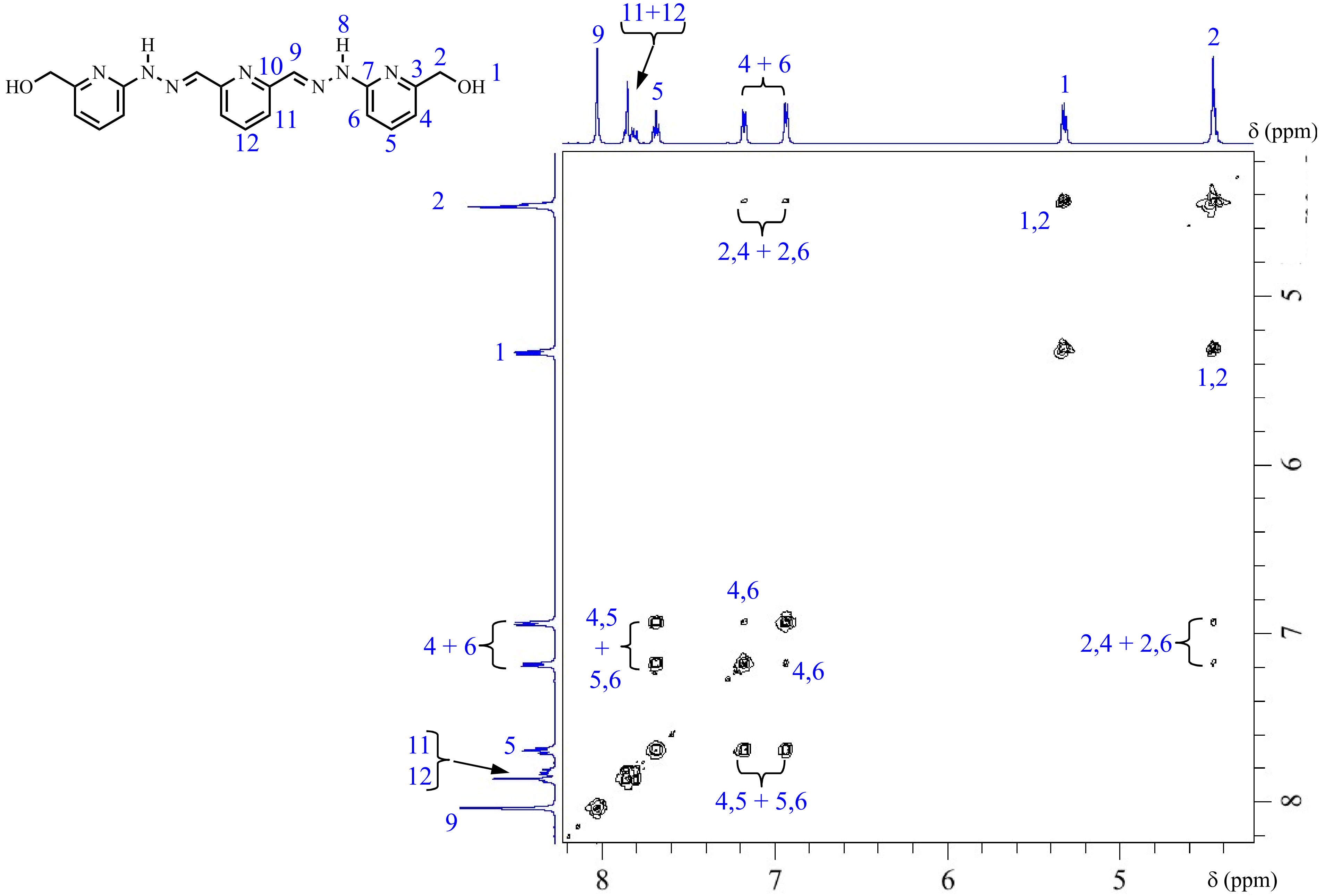

^{1}H-^{13}C HSQC NMR spectrum (500 MHz, DMSO-d_6) of compound **P3**

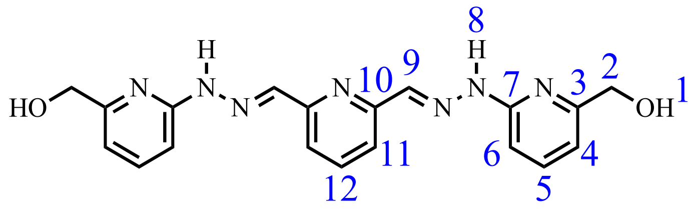

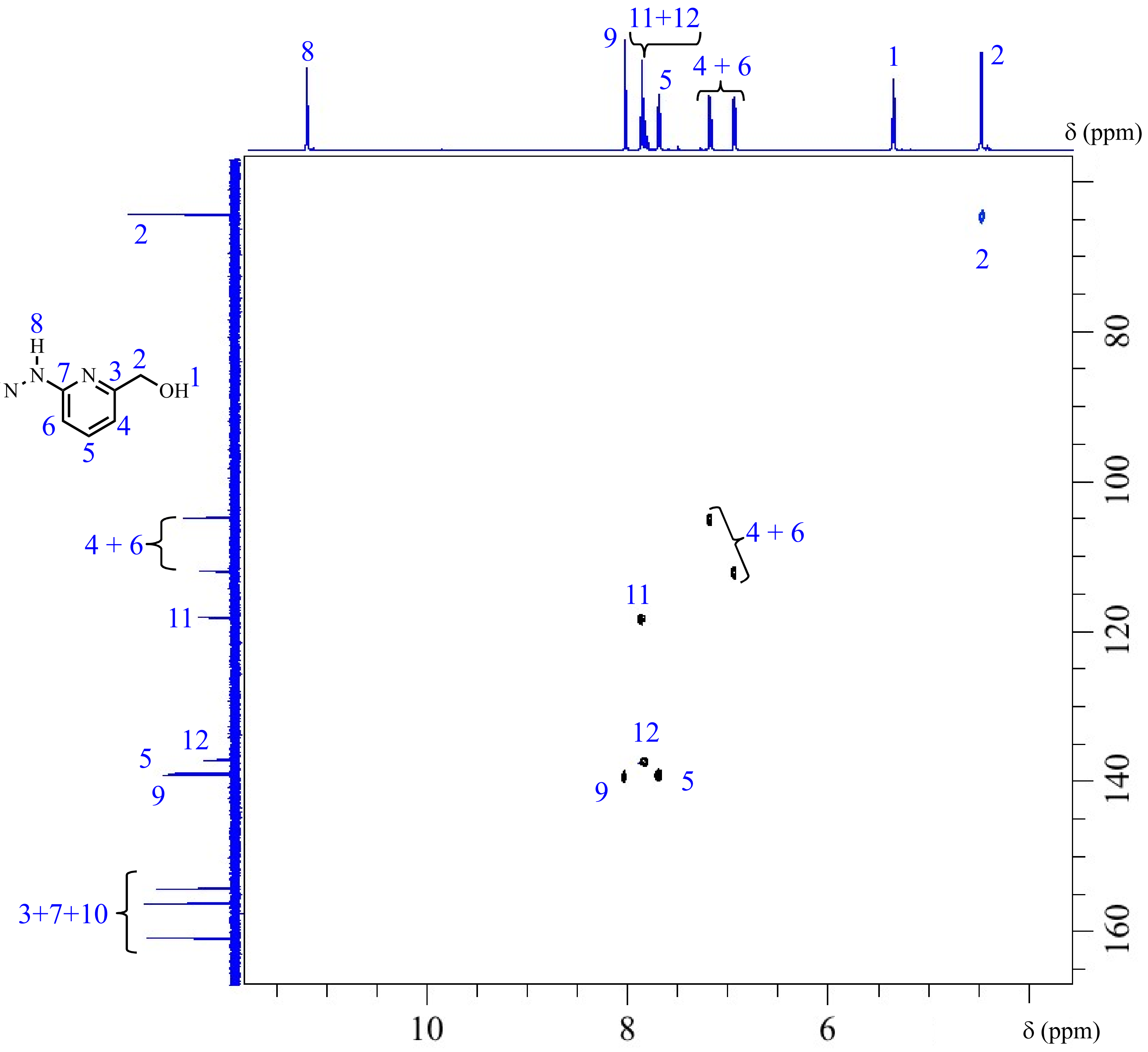

Part of the ^{1}H-^{13}C HSQC NMR spectrum (500 MHz, DMSO-d_6) of compound **P3**

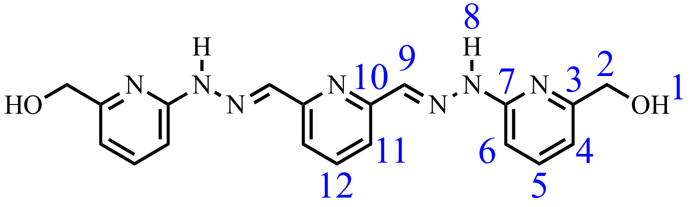

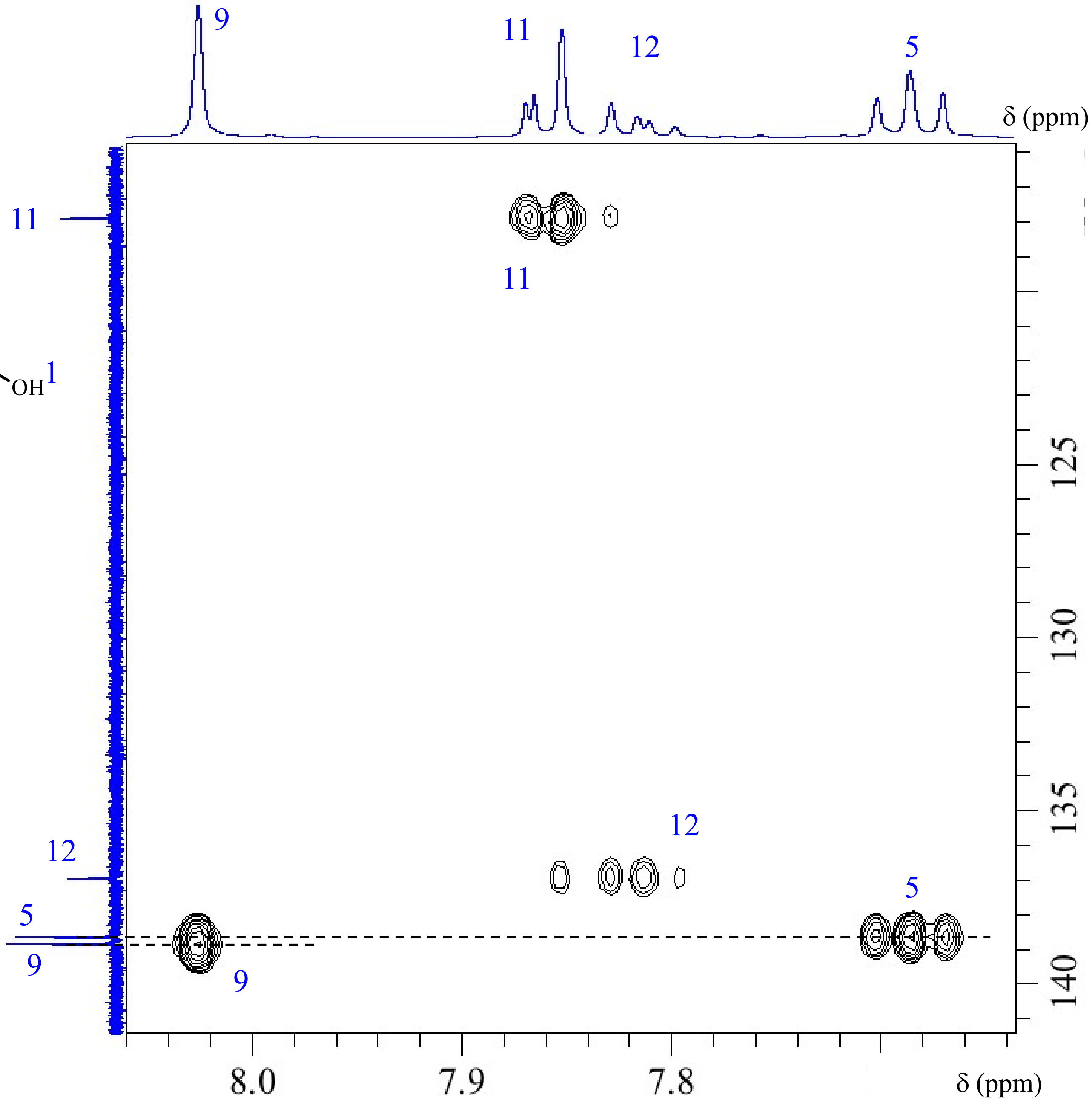

^{1}H-^{13}C HMBC NMR spectrum (500 MHz, DMSO-d_6) of compound **P3**

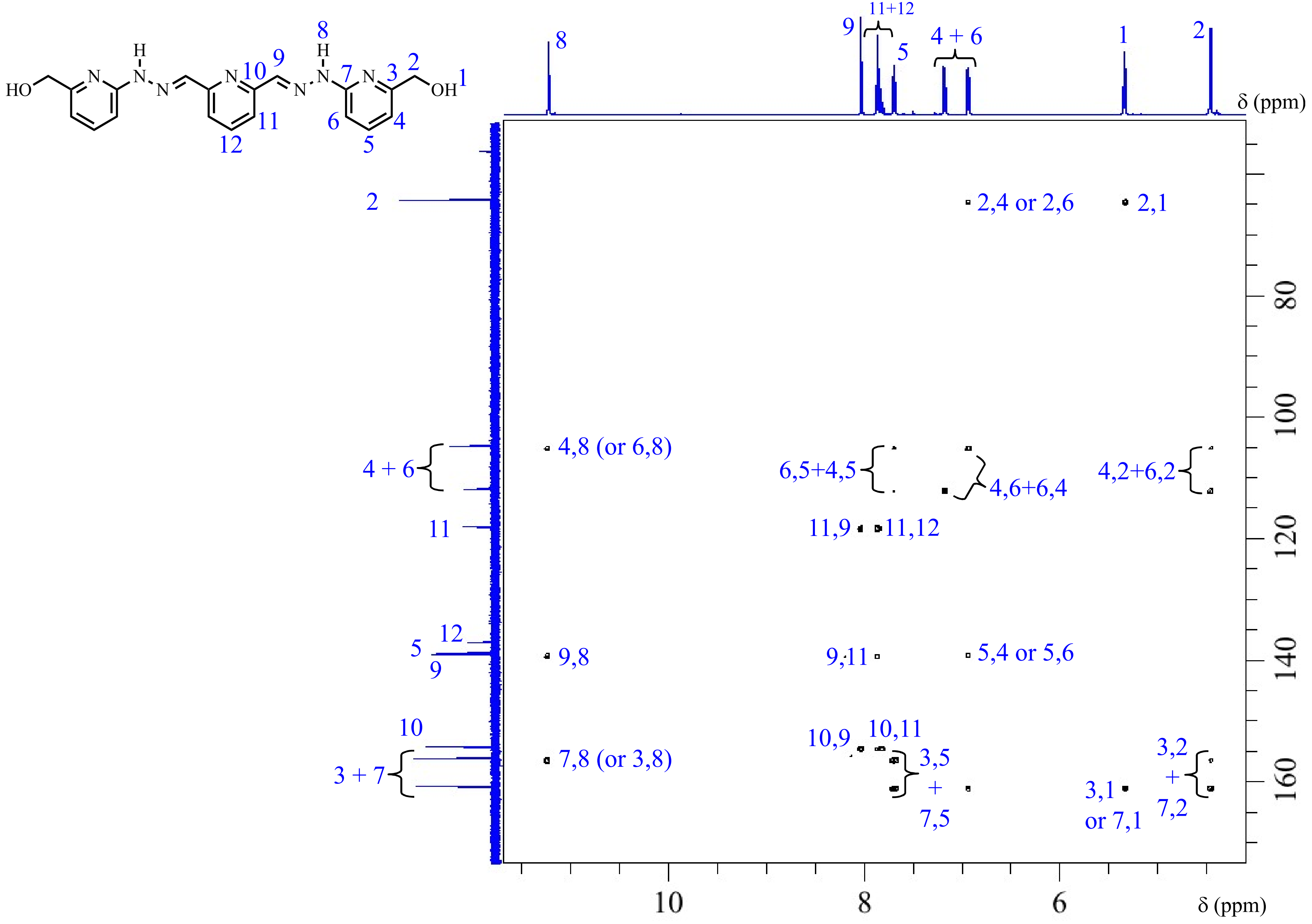

NMR spectra of compound

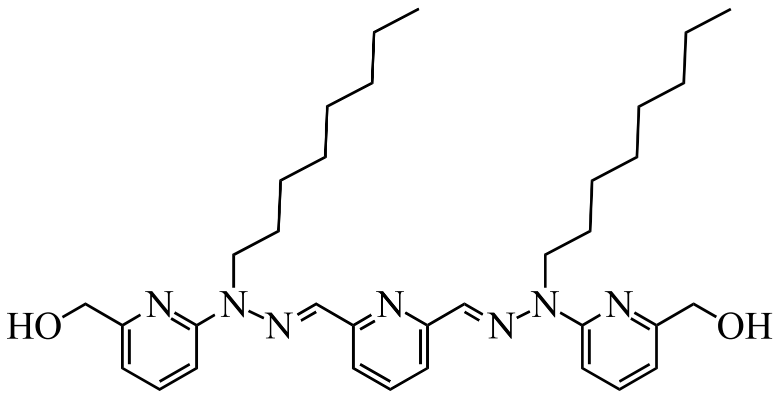

P4

^{1}H NMR spectrum (500 MHz, $CDCl_3$, δ_{ref} = 7.26 ppm) of compound **P4**

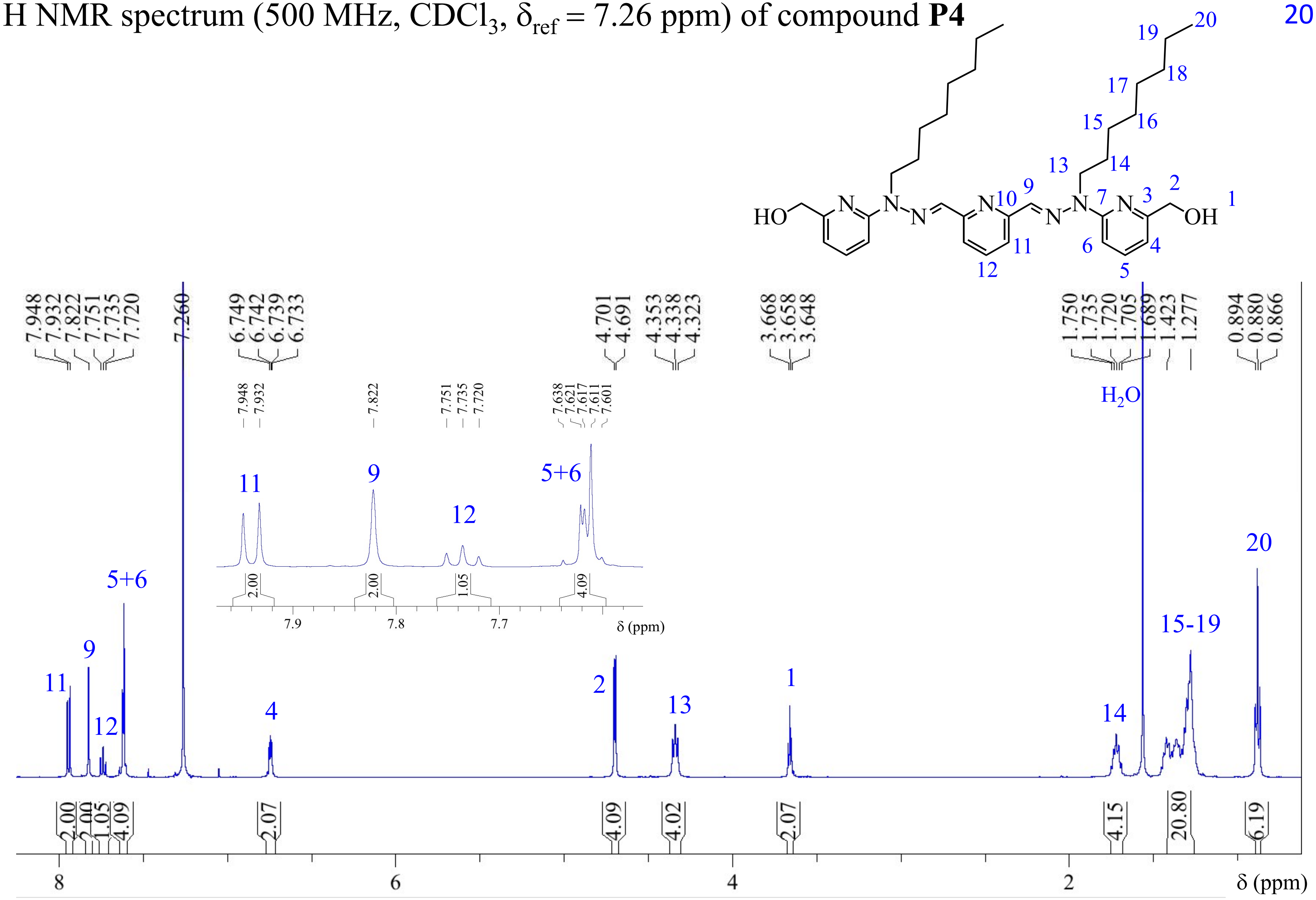

^{13}C NMR spectrum (125 MHz, $CDCl_3$, δ_{ref} = 77.4 ppm) of compound **P4**

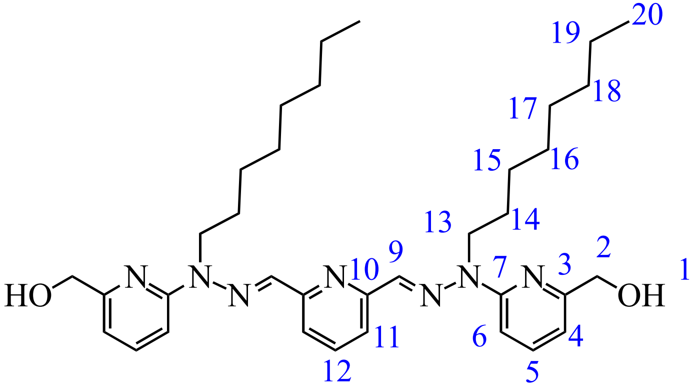

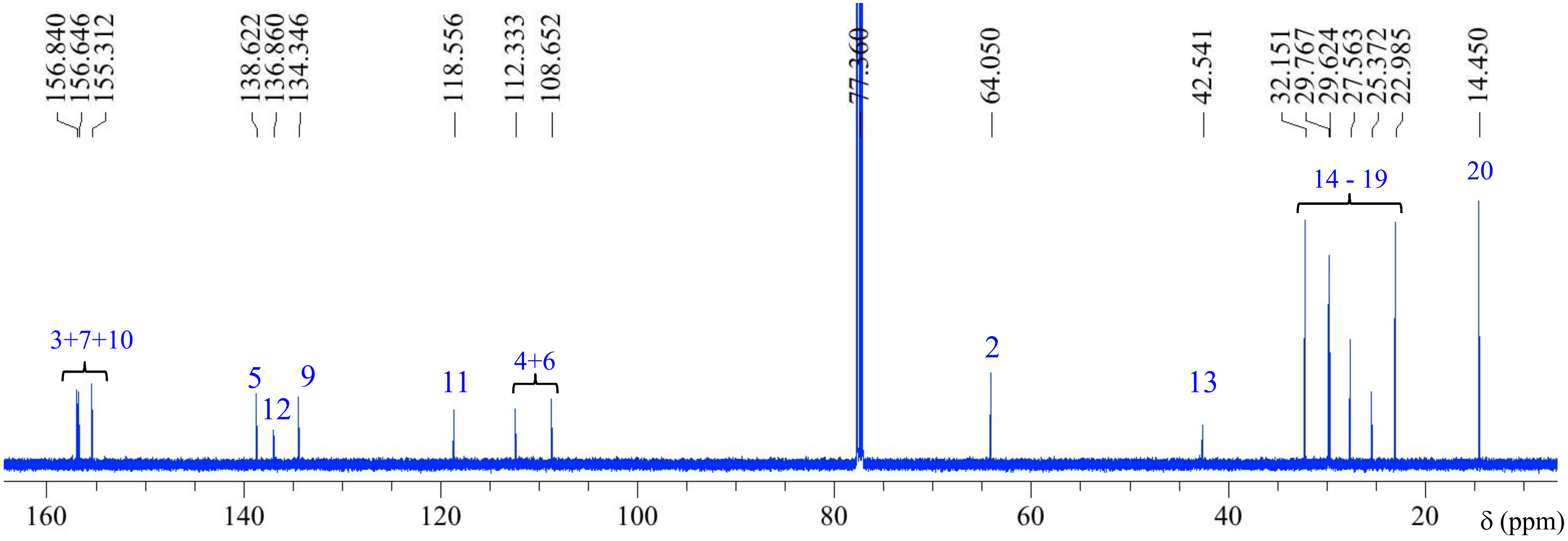

^{1}H-^{1}H COSY NMR spectrum (500 MHz, $CDCl_3$) of compound **P4**

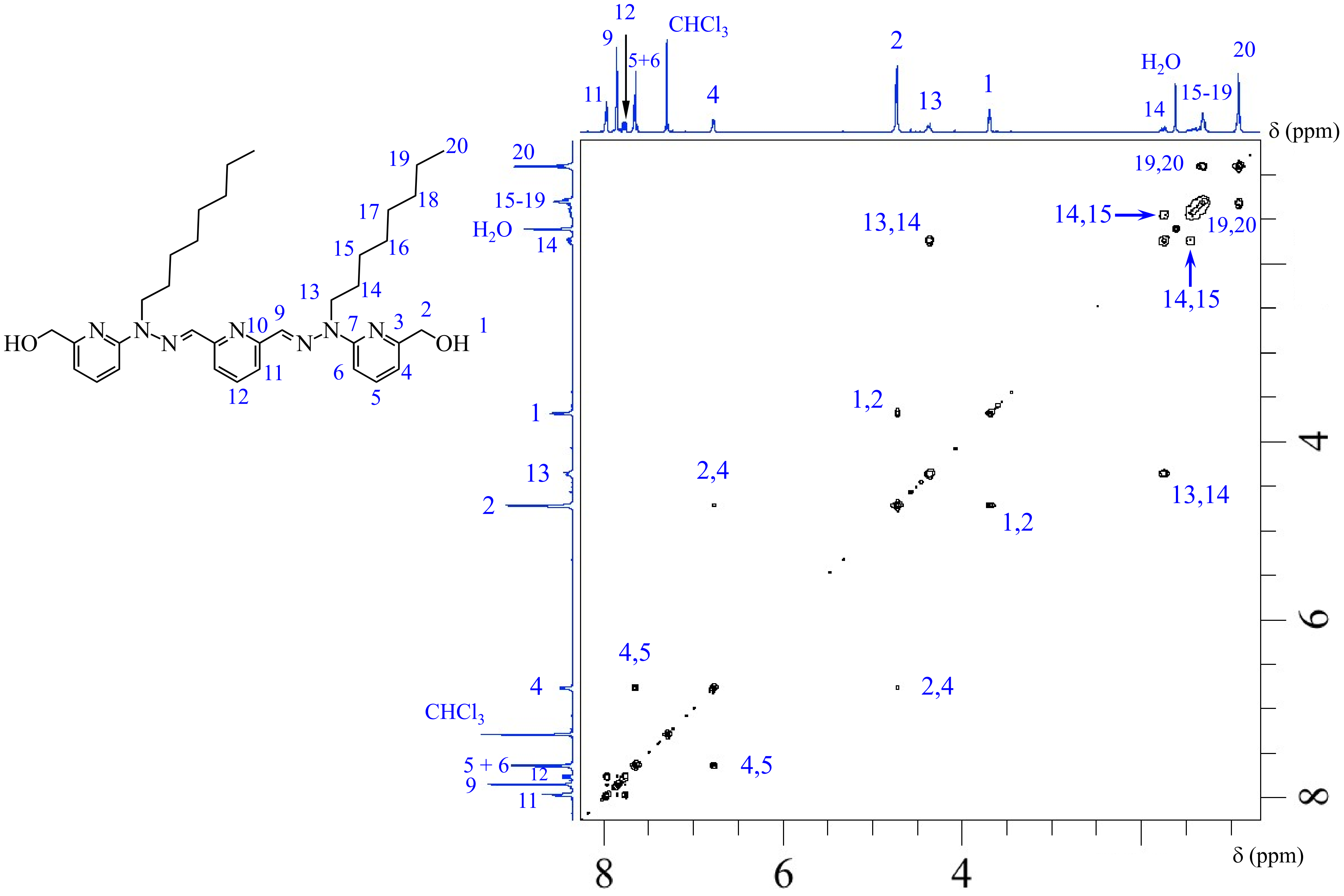

Part of the ^{1}H-^{1}H COSY NMR spectrum (500 MHz, $CDCl_3$) of compound **P4**

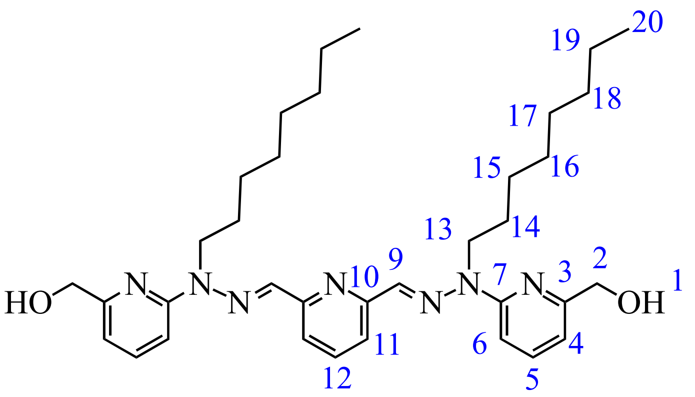

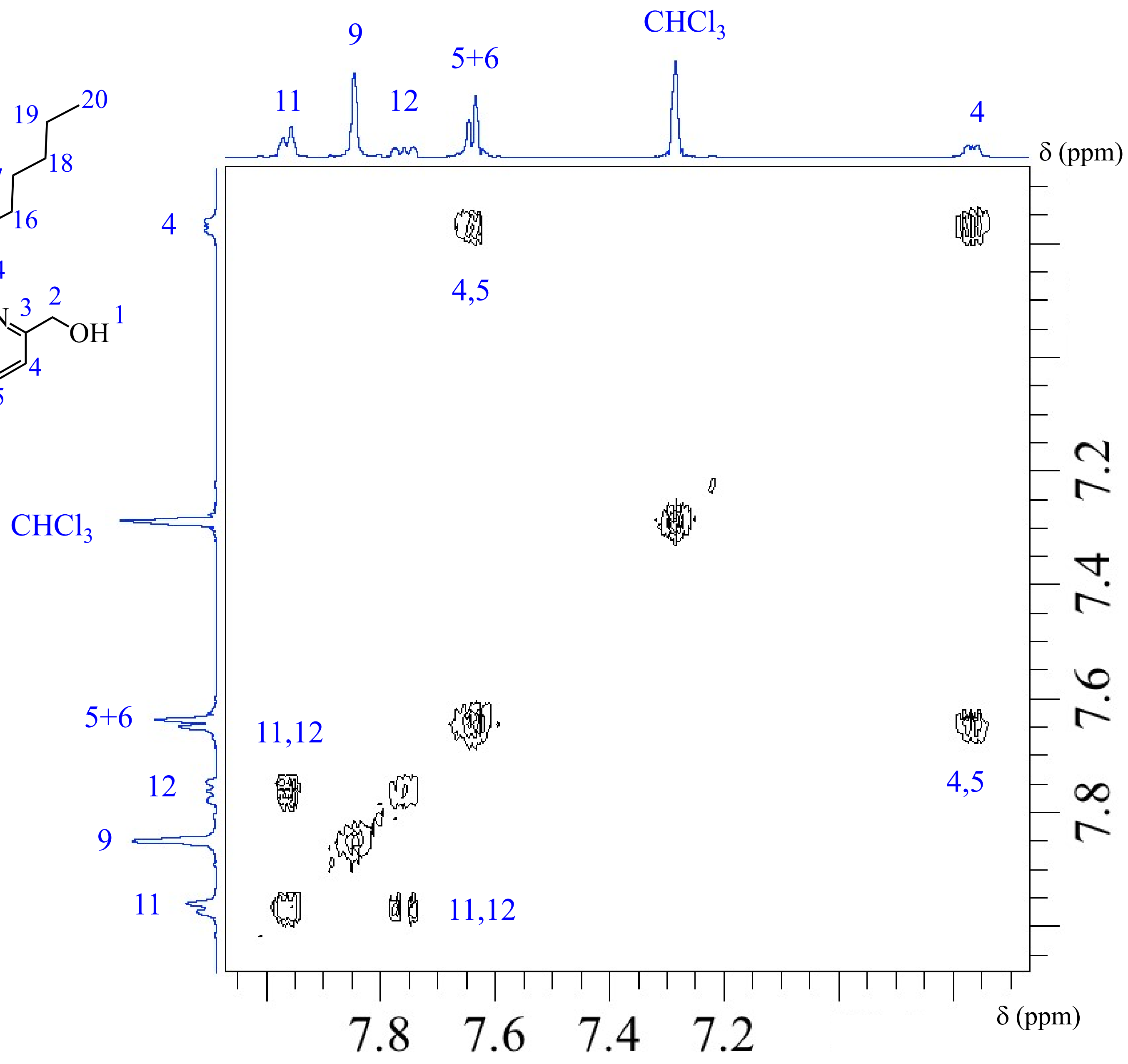

^{1}H-^{13}C HSQC NMR spectrum (500 MHz, $CDCl_3$) of compound **P4**

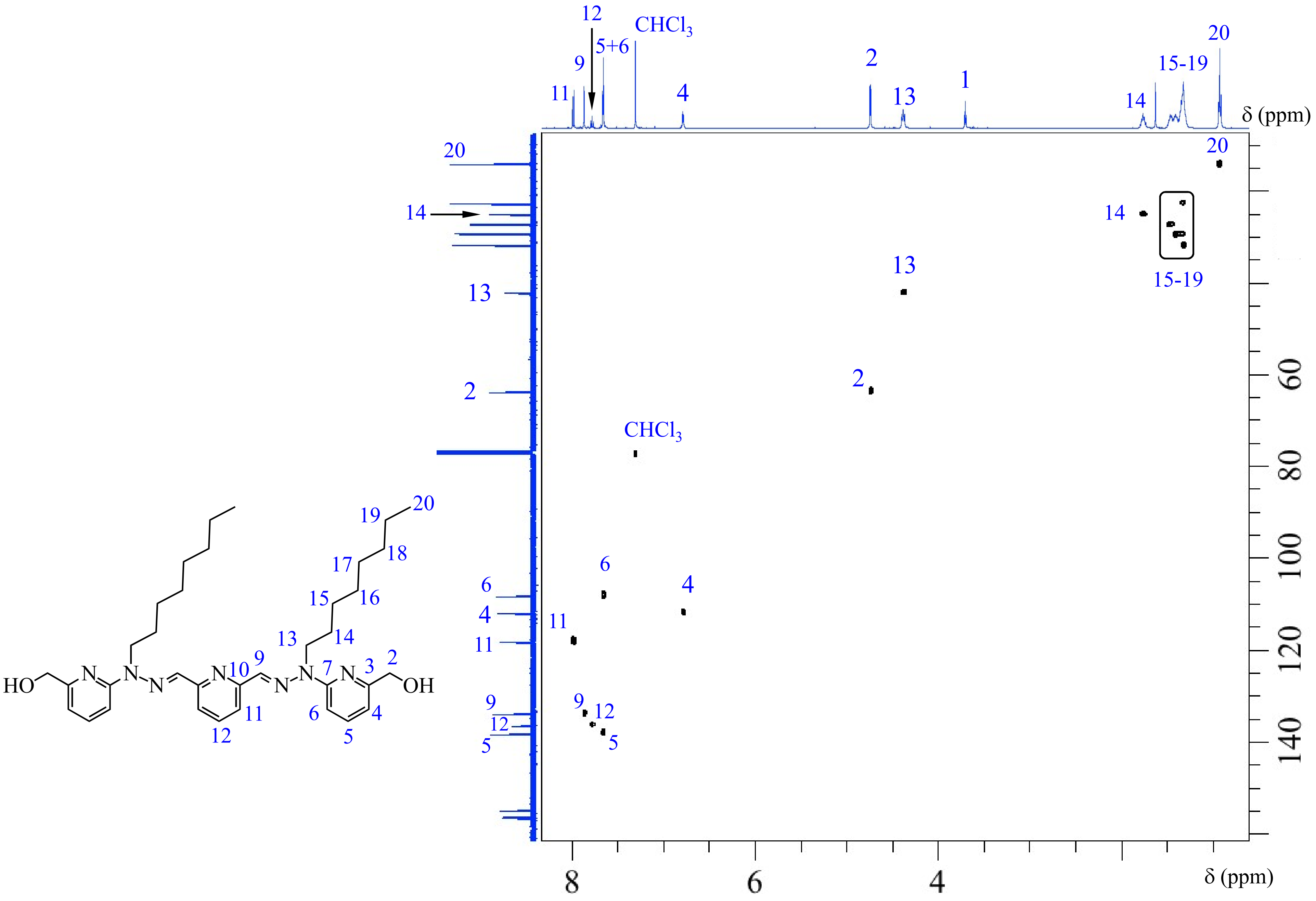

Part of the ^{1}H-^{13}C HSQC NMR spectrum (500 MHz, $CDCl_3$) of compound **P4**

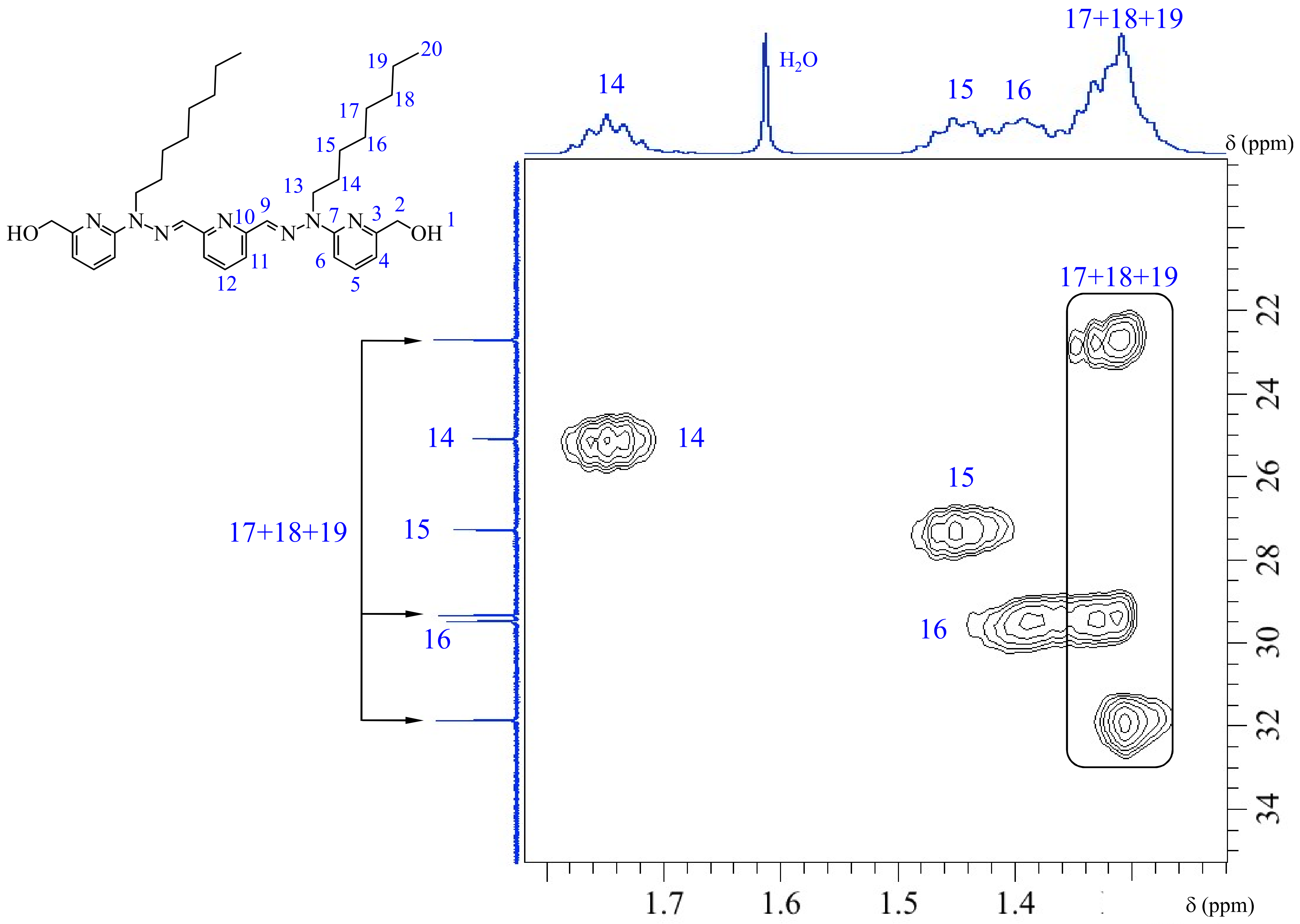

NMR spectra of compound

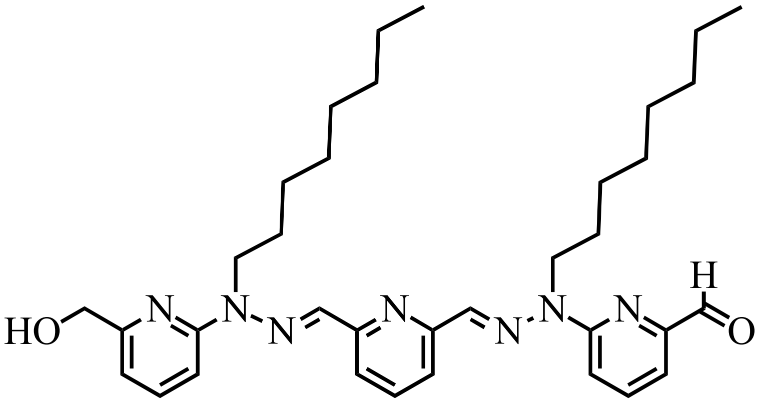

M

^{1}H NMR spectrum (400 MHz, $CDCl_3$, δ_{ref} = 7.26 ppm) of compound **M**

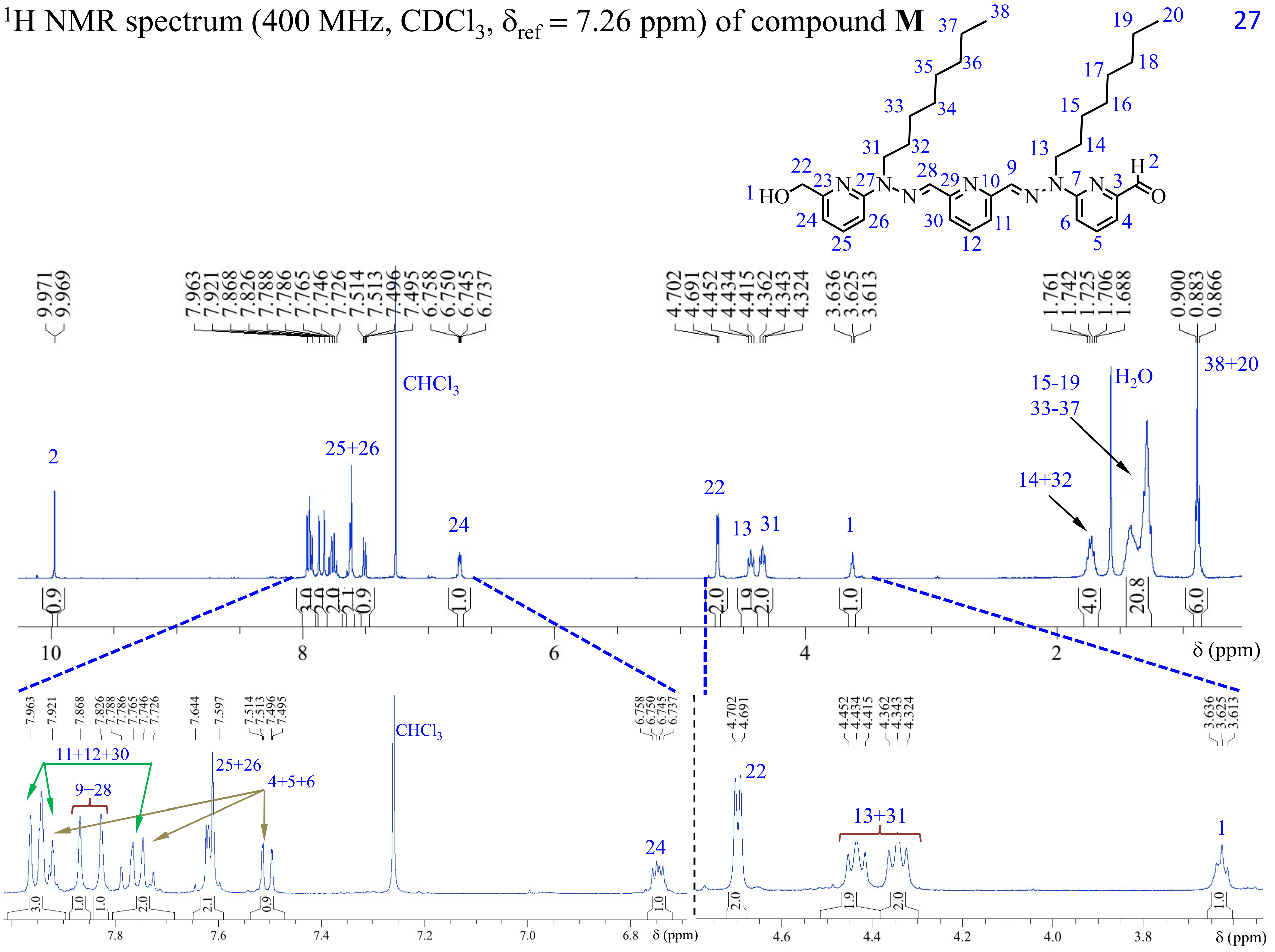

^{13}C NMR spectrum (125 MHz, $CDCl_3$, δ_{ref} = 77.4 ppm) of compound **M**

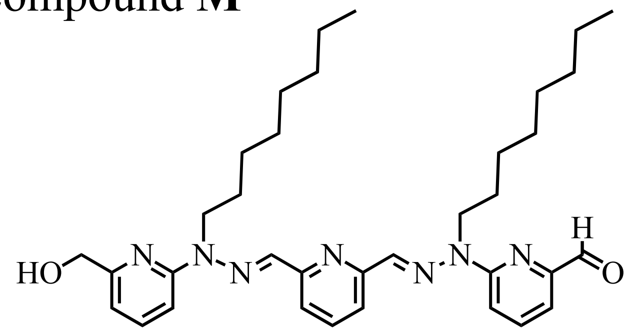

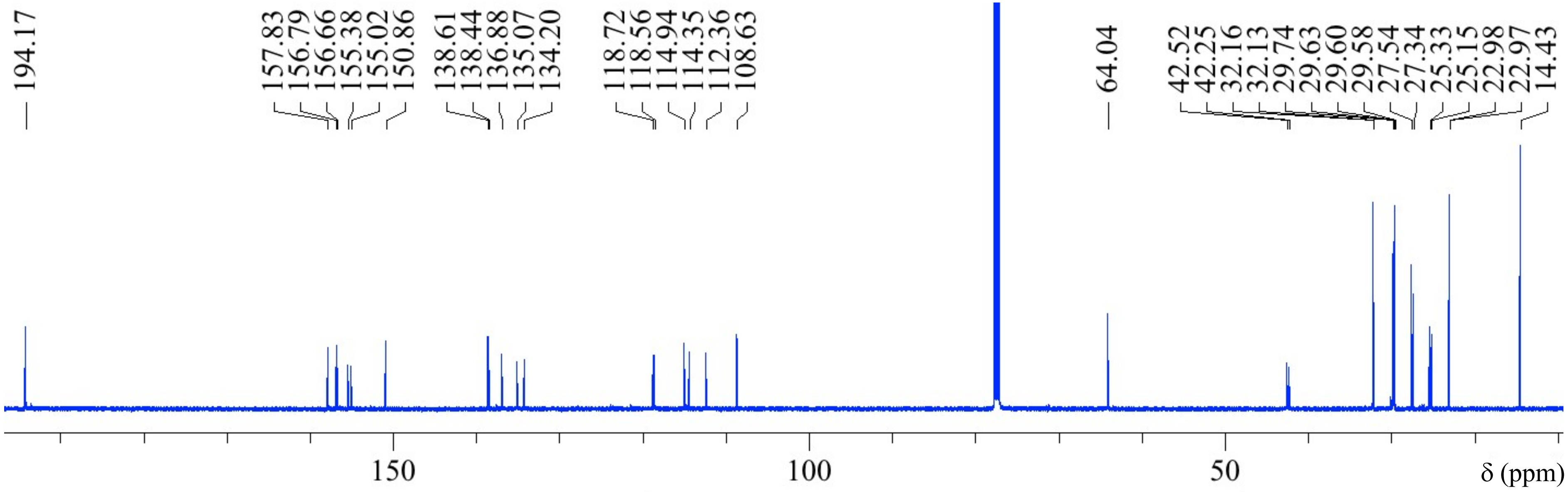

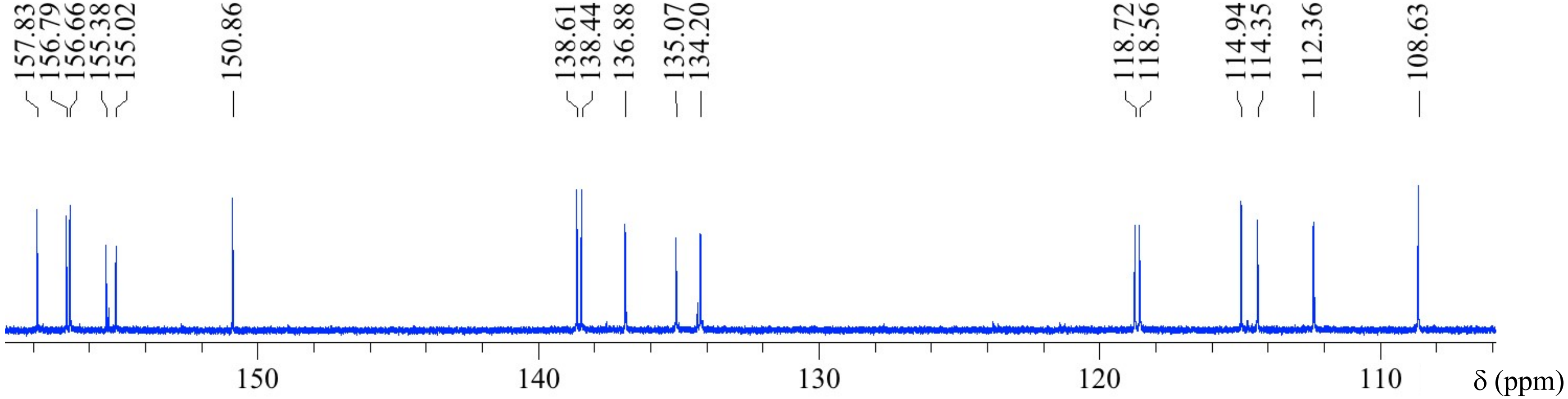

^{1}H-^{1}H COSY NMR spectrum (500 MHz, $CDCl_3$) of compound **M**

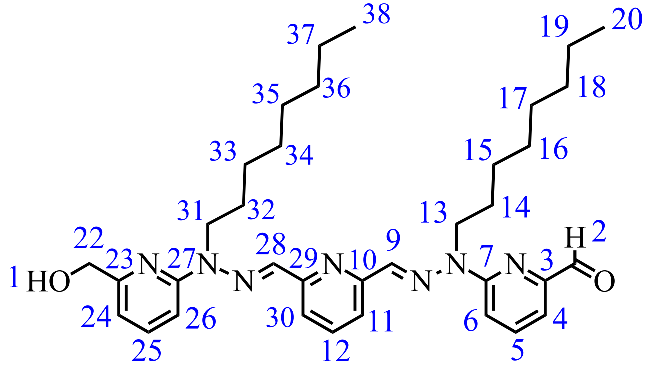

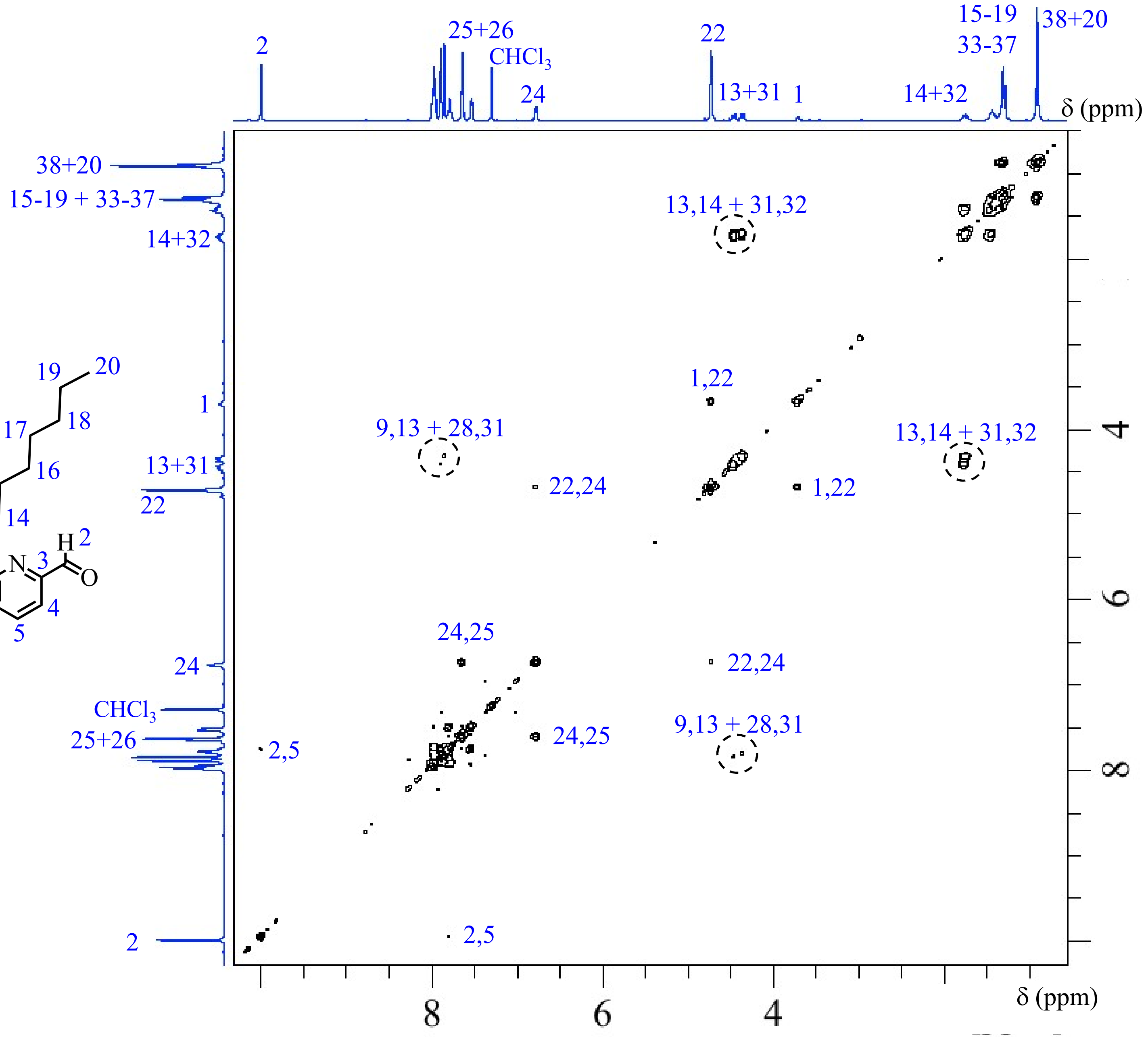

Part of the ^{1}H-^{1}H COSY NMR spectrum (500 MHz, $CDCl_3$) of compound **M**

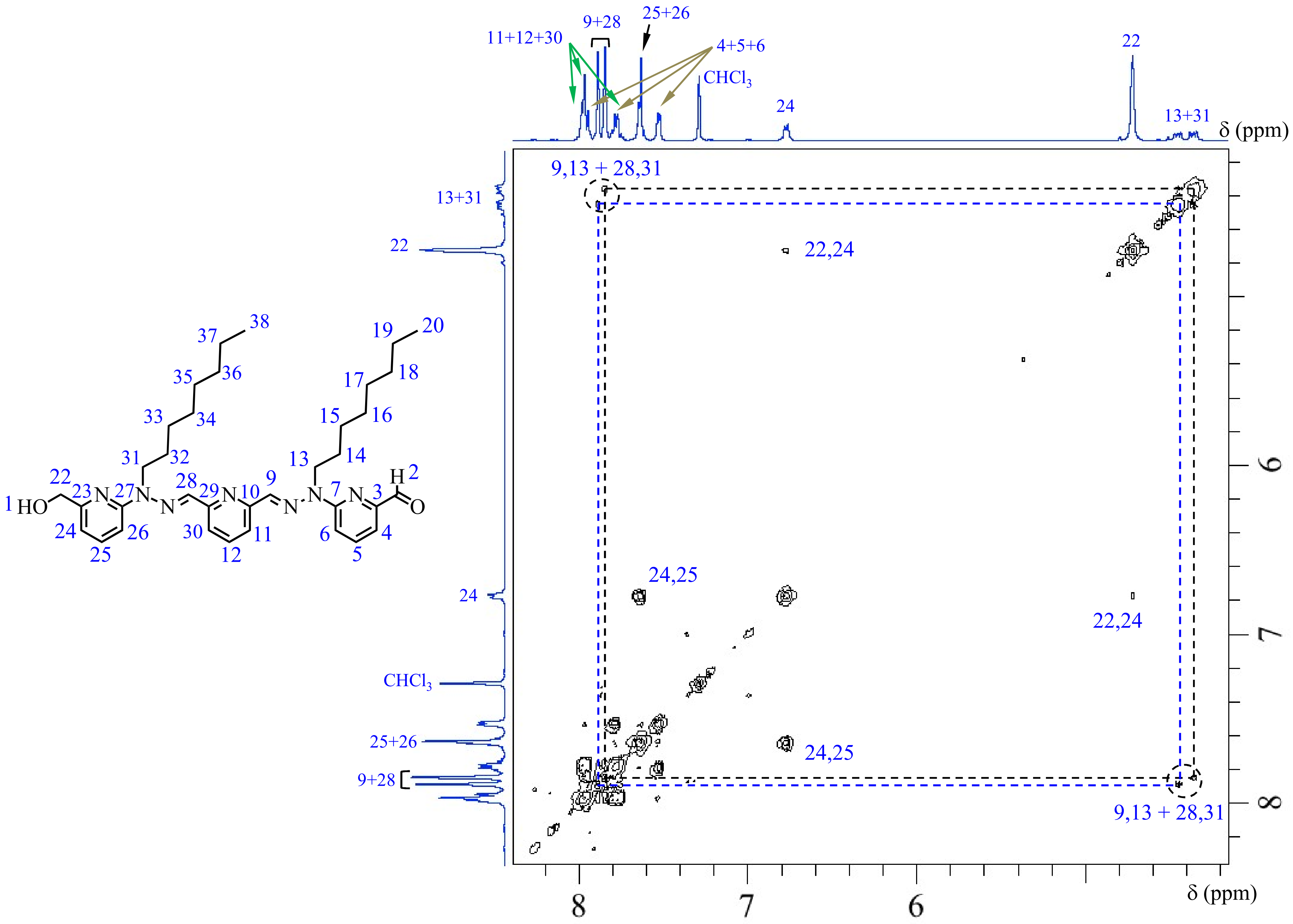

NMR spectra of compound

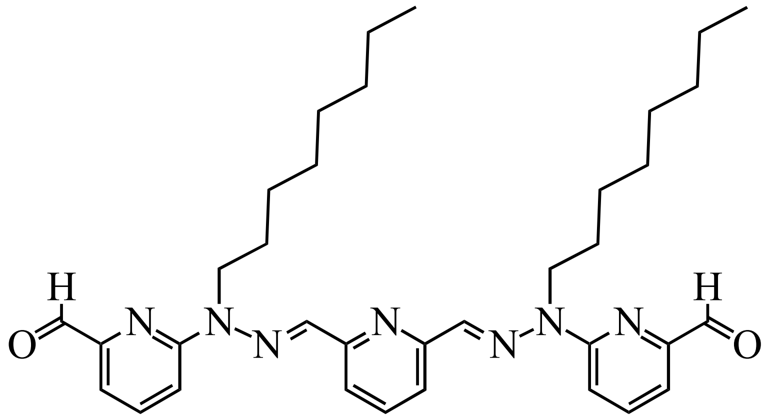

A

^{1}H NMR spectrum (400 MHz, $CDCl_3$, δ_{ref} = 7.26 ppm) of compound **A**

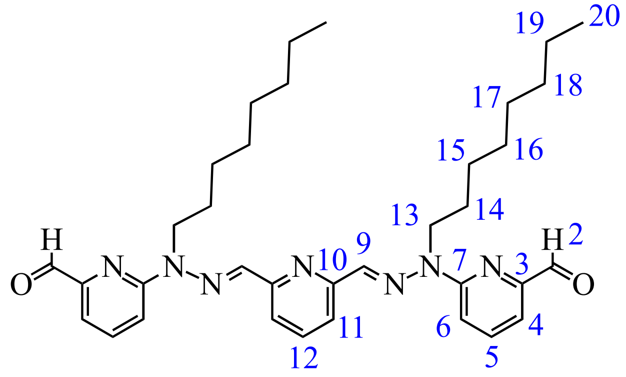

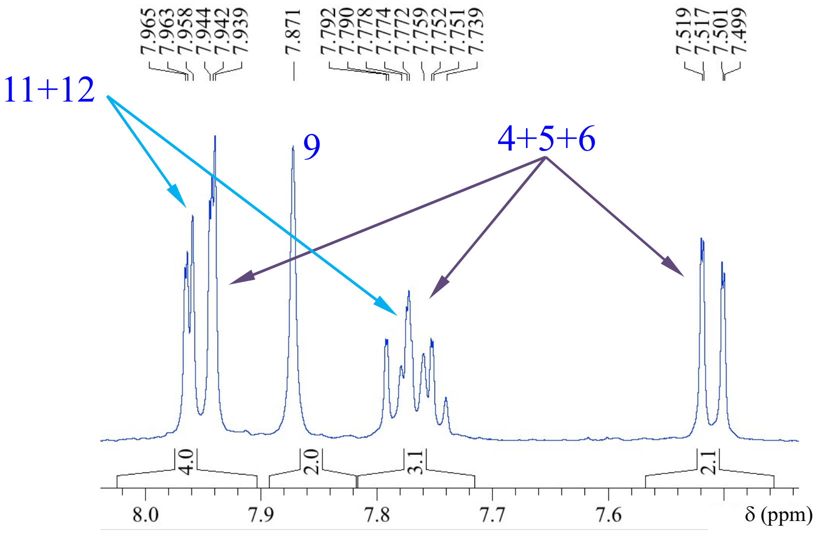

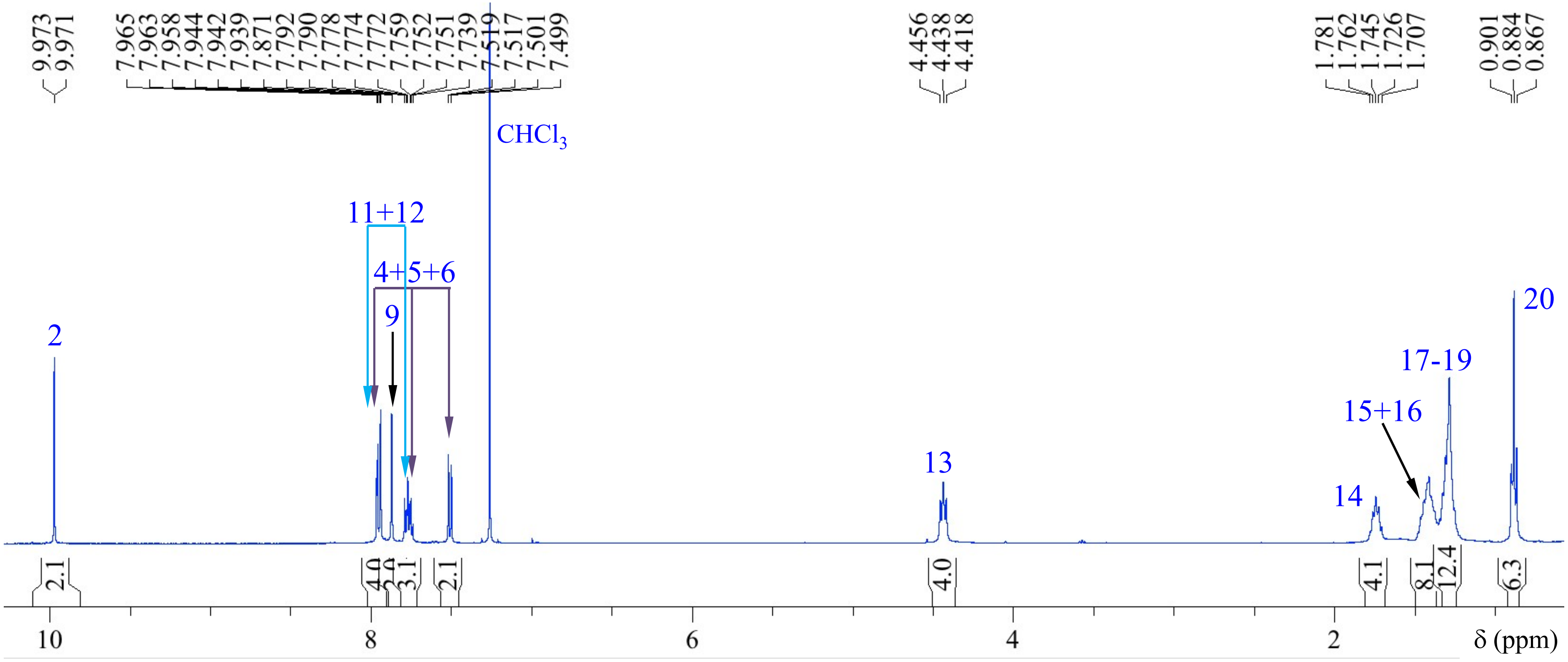

^{13}C NMR spectrum (125 MHz, $CDCl_3$, δ_{ref} = 77.4 ppm) of compound **A**

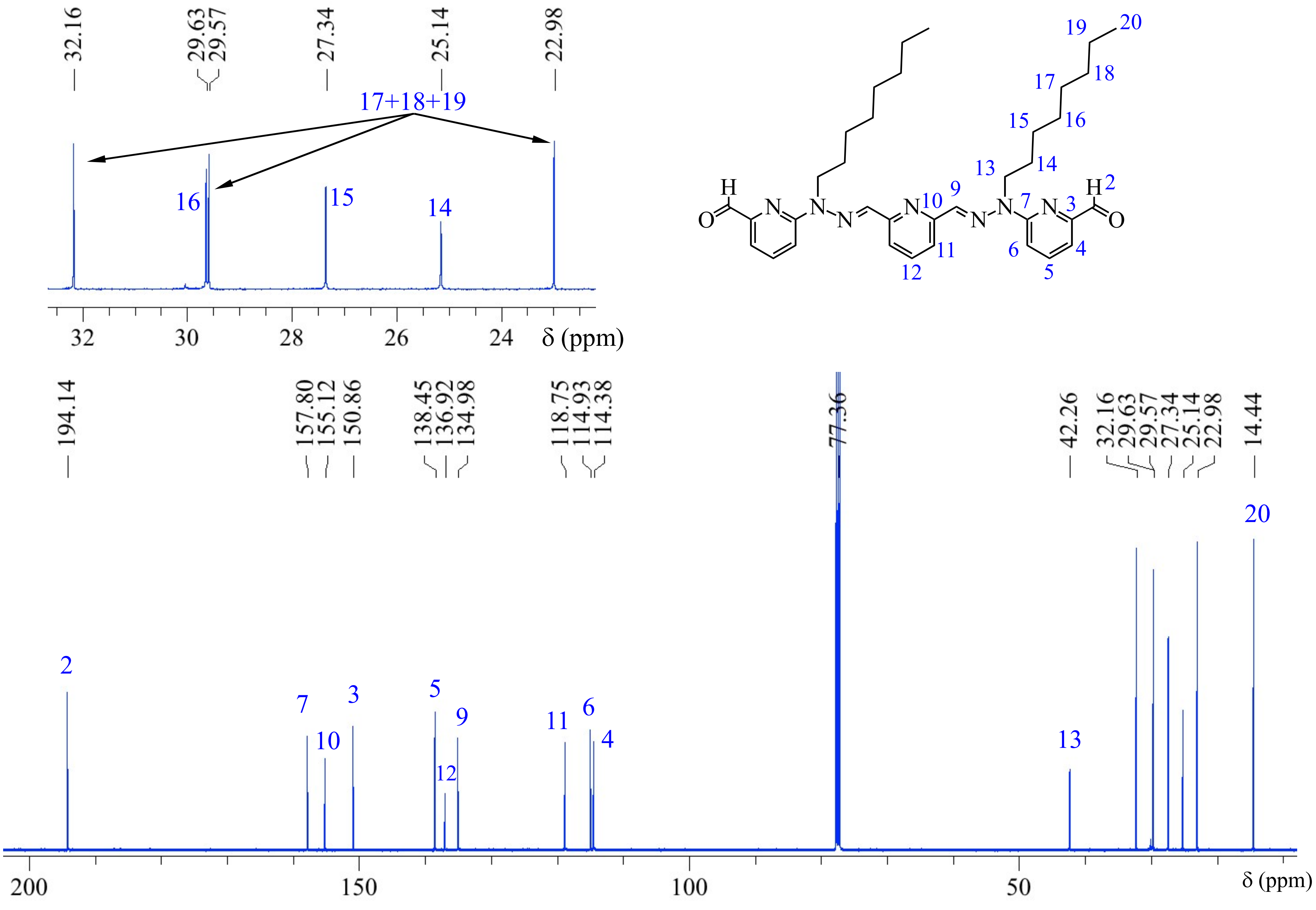

^{1}H-^{1}H COSY NMR spectrum (500 MHz, $CDCl_3$) of compound **A**

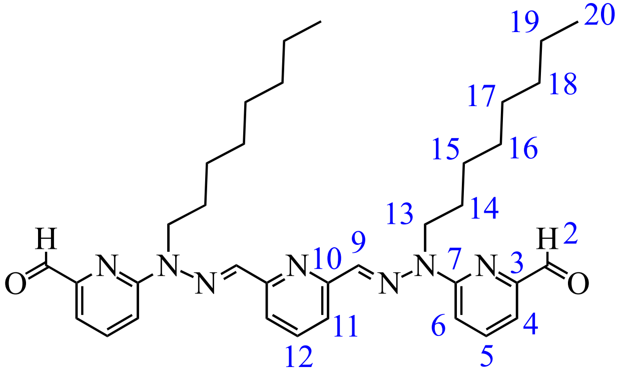

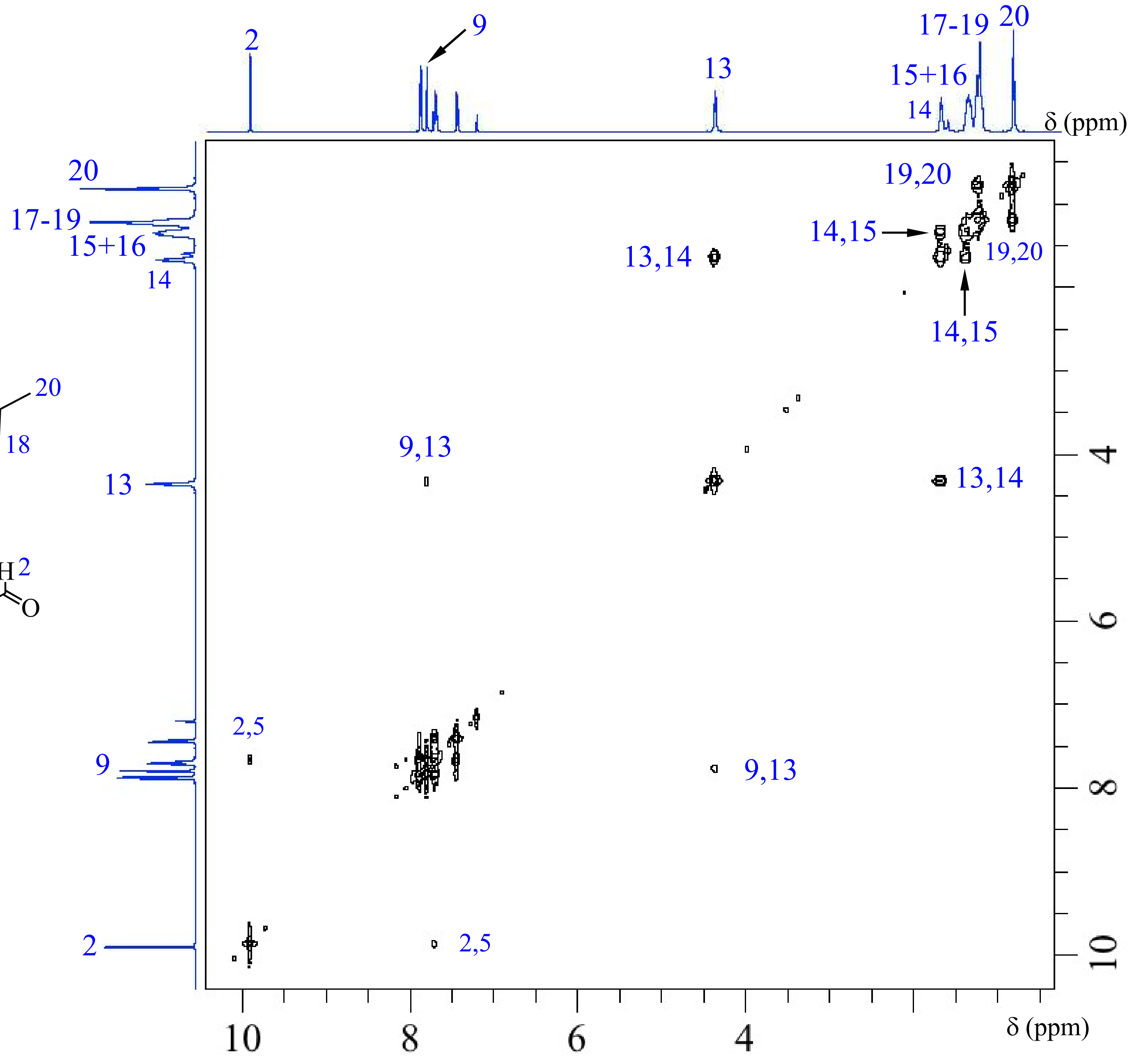

Part of ^1H-^1H COSY NMR spectrum (500 MHz, $CDCl_3$) of compound **A**

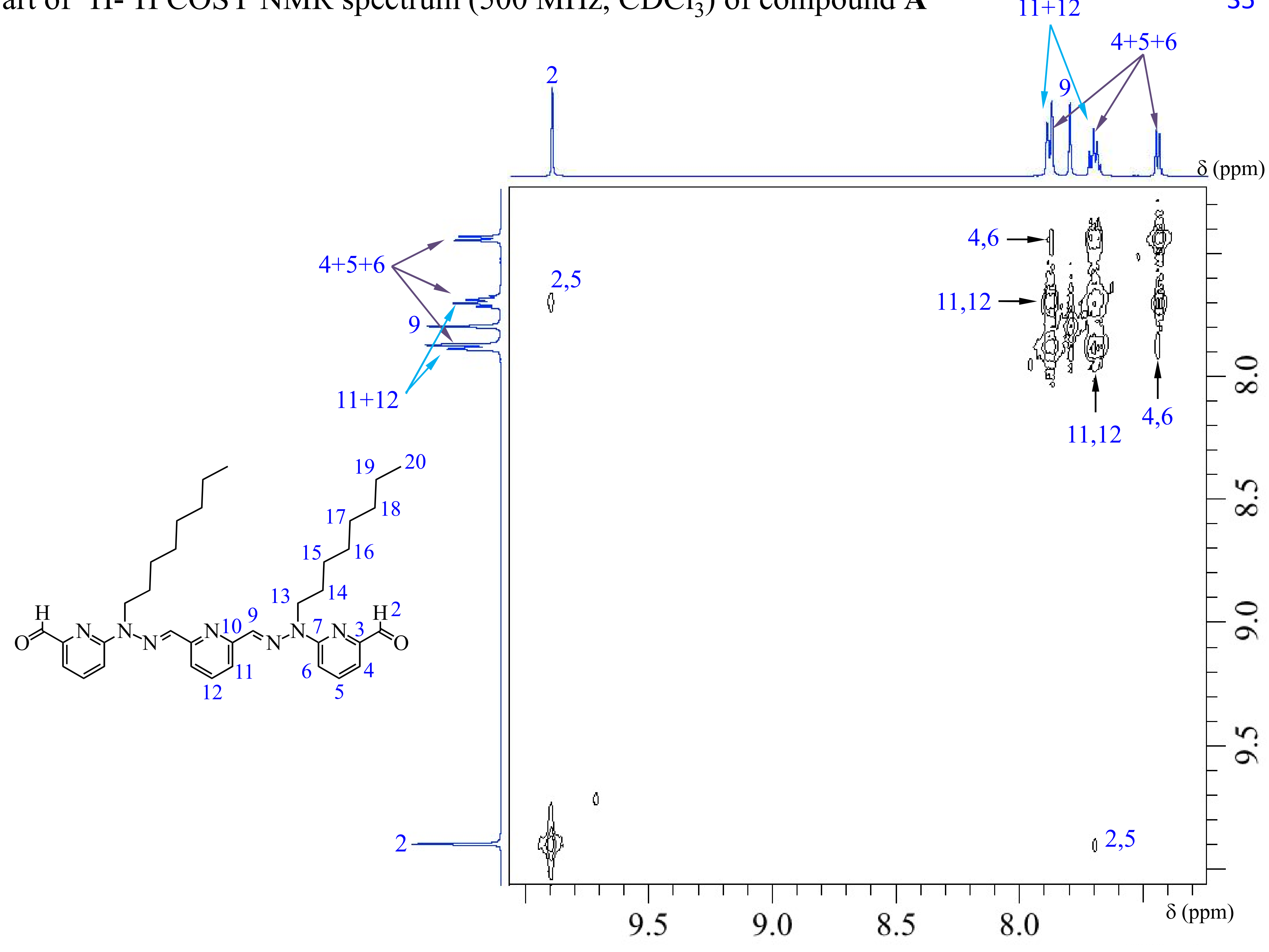

Part of ^1H-^1H COSY NMR spectrum (500 MHz, $CDCl_3$) of compound **A**

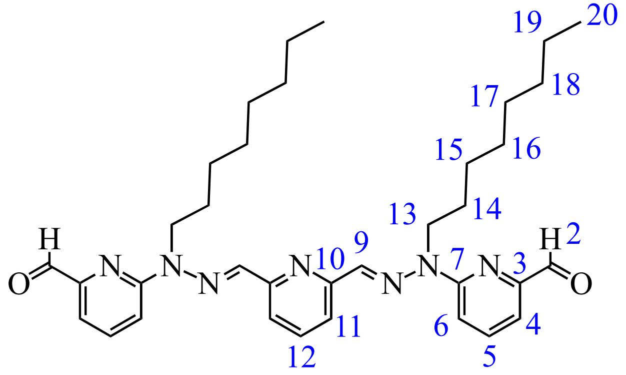

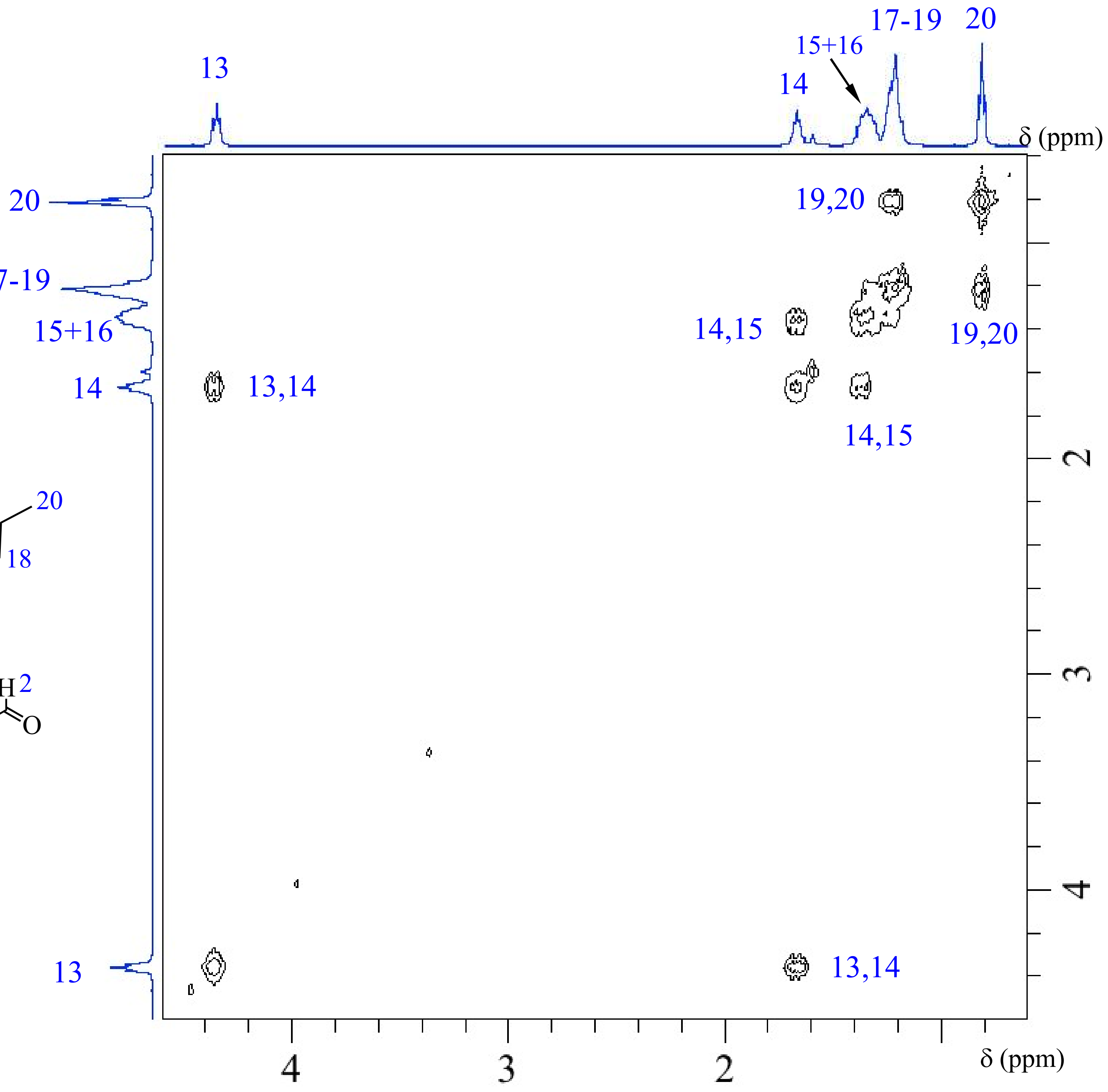

^{1}H-^{13}C HSQC NMR spectrum (500 MHz, $CDCl_3$) of compound **A**

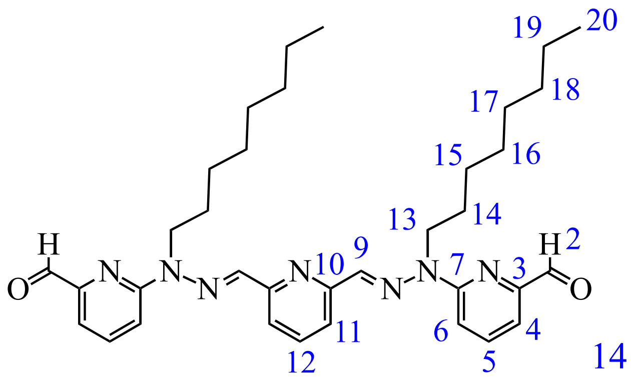

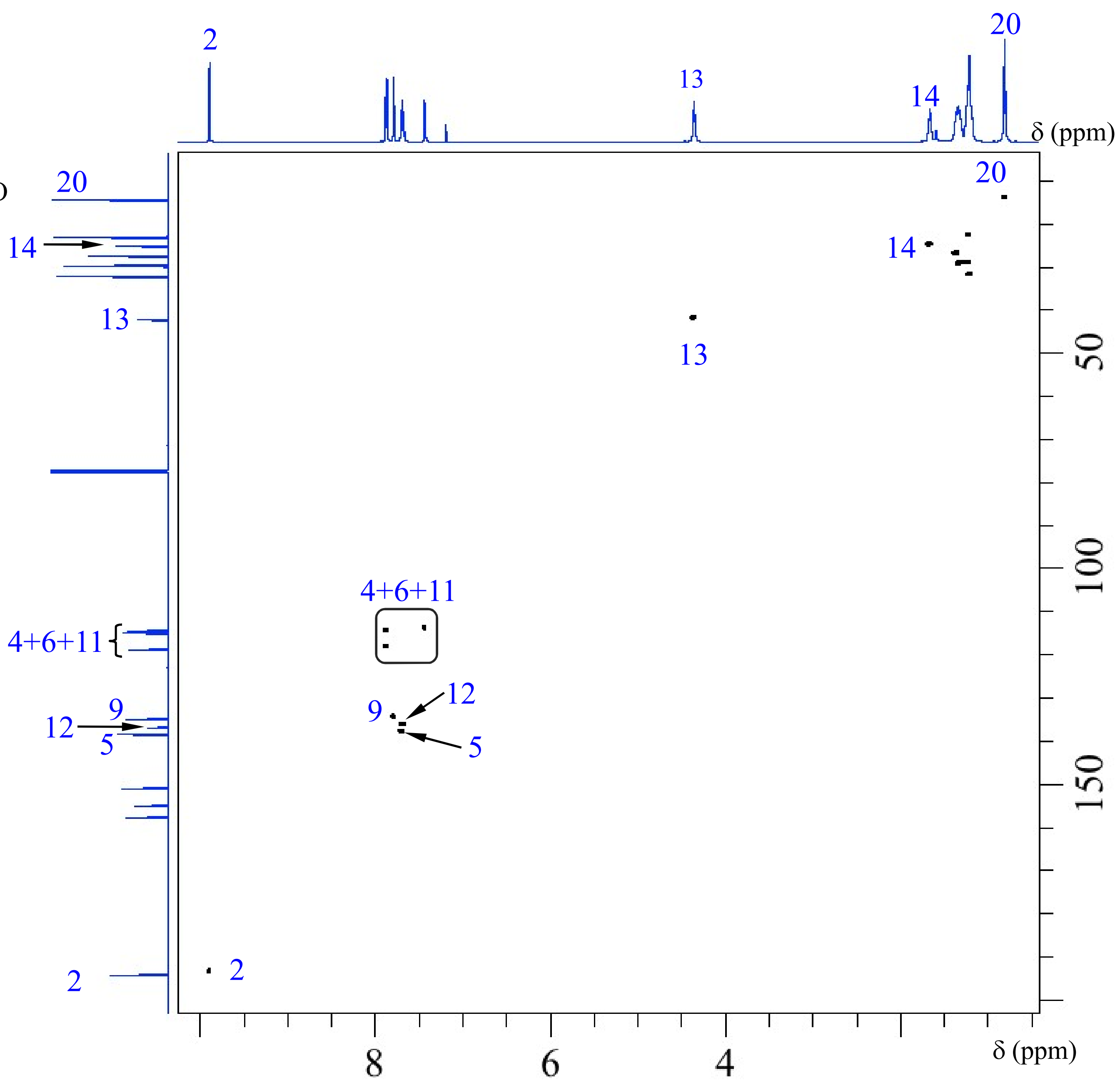

Part of the ^{1}H-^{13}C HSQC NMR spectrum (500 MHz, $CDCl_3$) of compound **A**

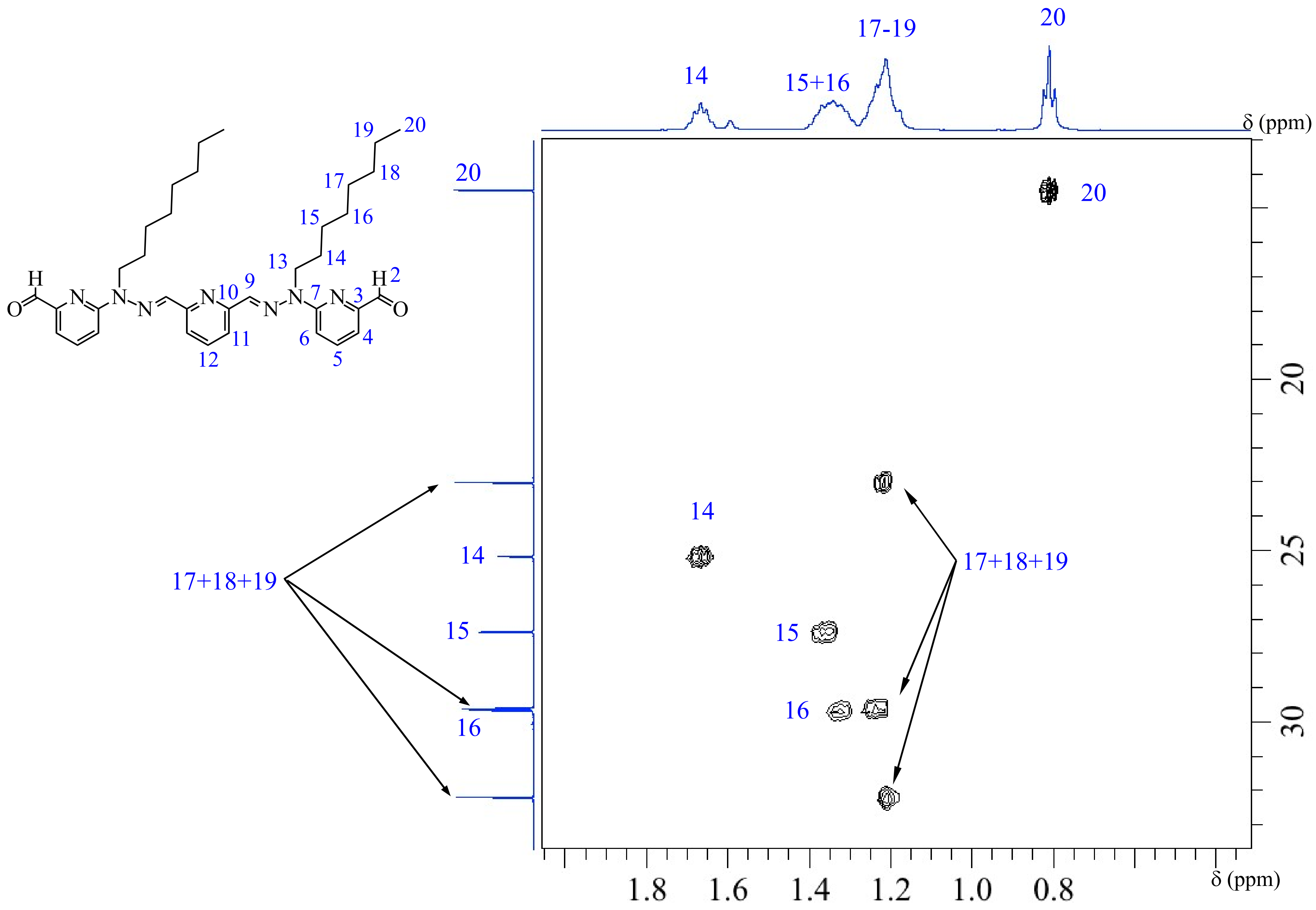

Part of the ^{1}H-^{13}C HSQC NMR spectrum (500 MHz, $CDCl_3$) of compound **A**

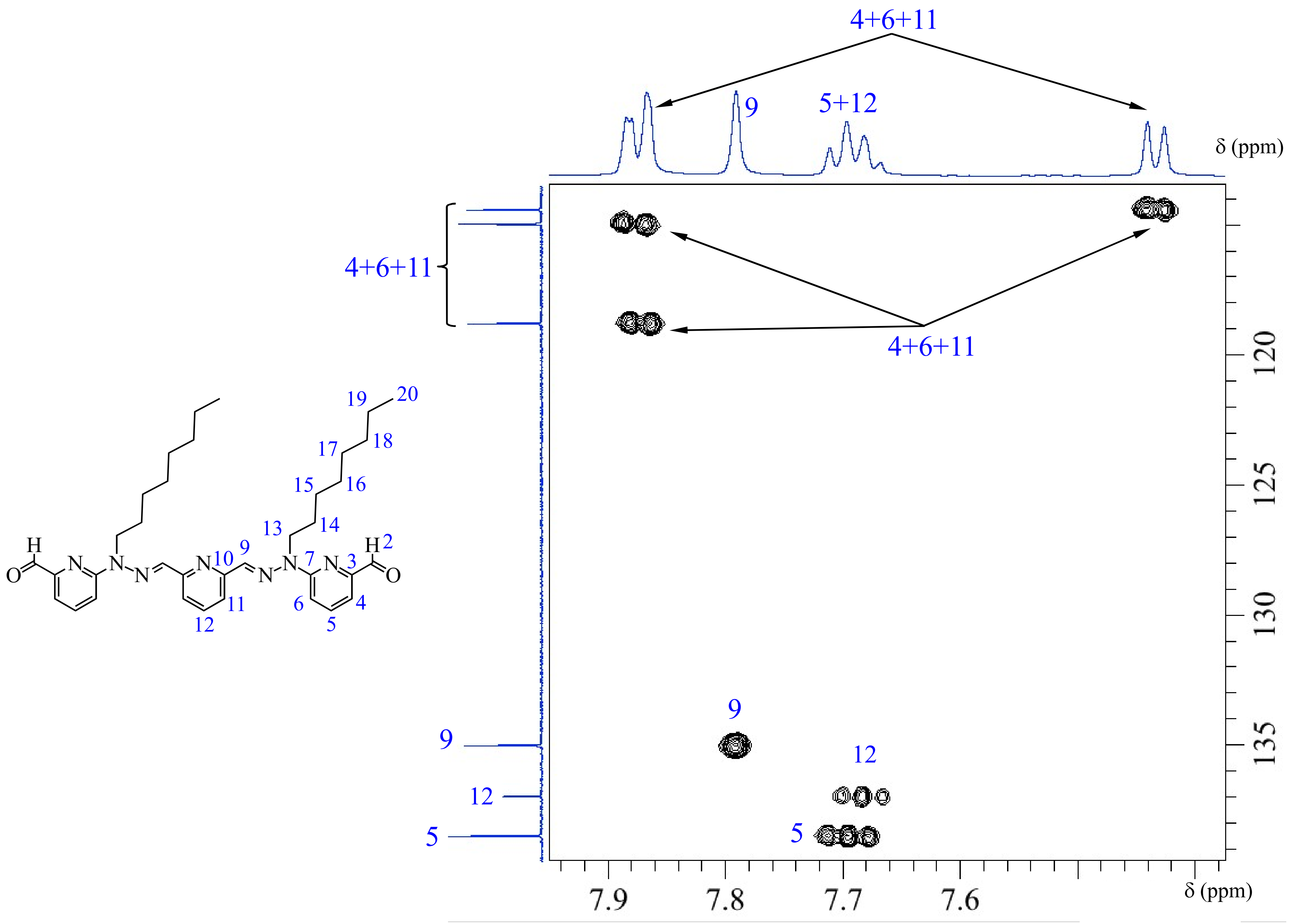

^{1}H-^{13}C HMBC NMR spectrum (500 MHz, $CDCl_3$) of compound **A**

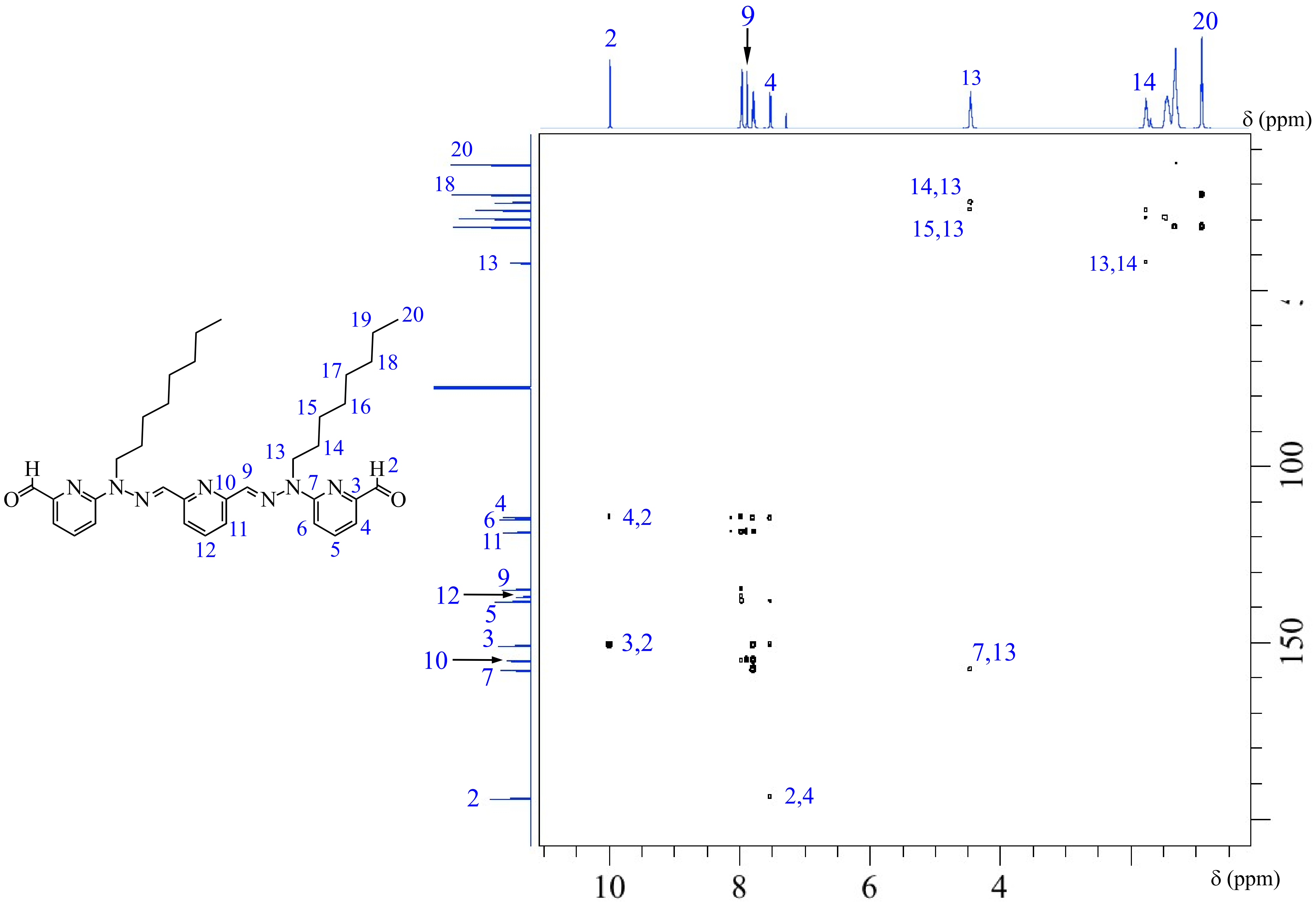

Part of the ^{1}H-^{13}C HMBC NMR spectrum (500 MHz, $CDCl_3$) of compound **A**

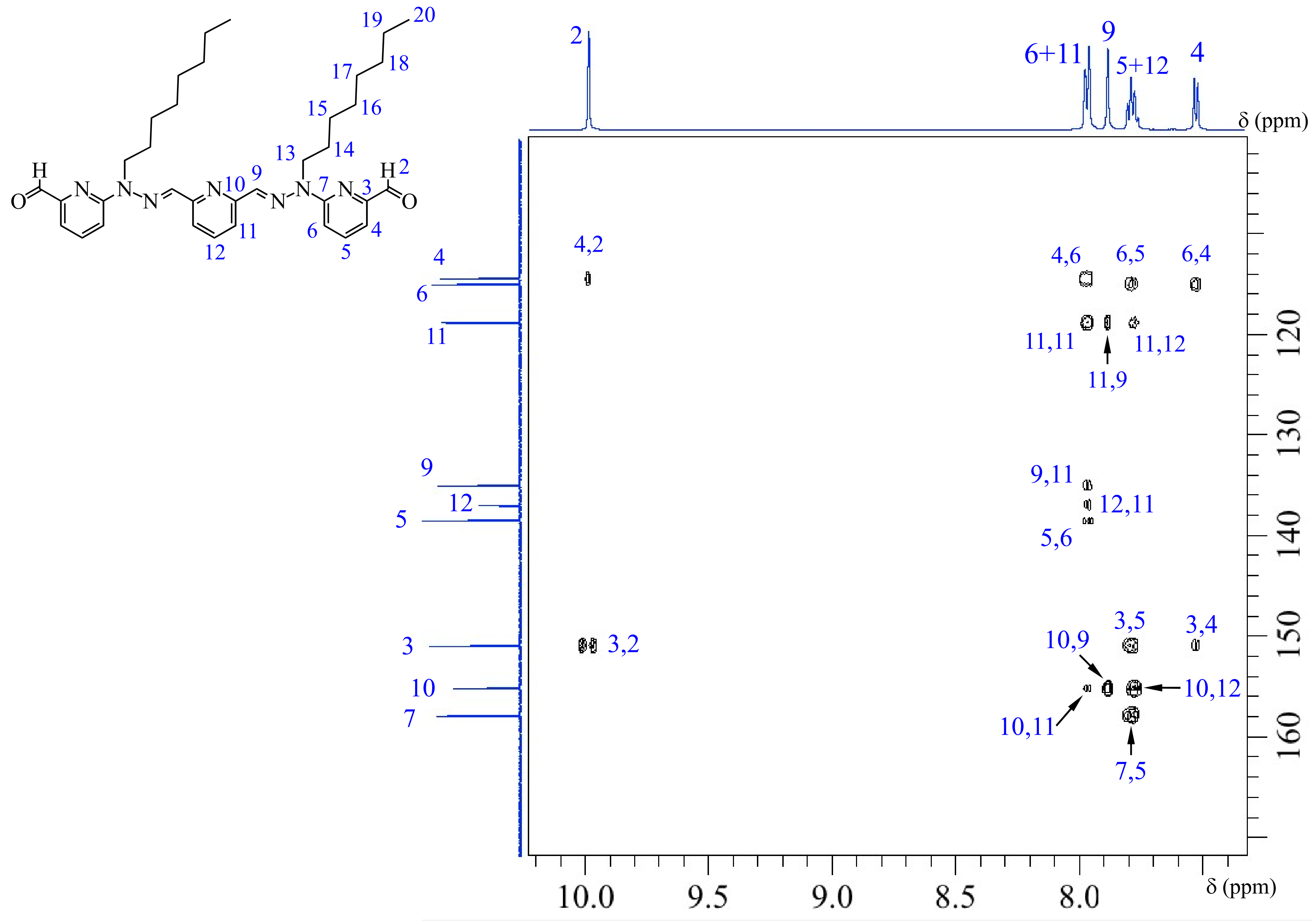

Part of the ^{1}H-^{13}C HMBC NMR spectrum (500 MHz, $CDCl_3$) of compound **A**

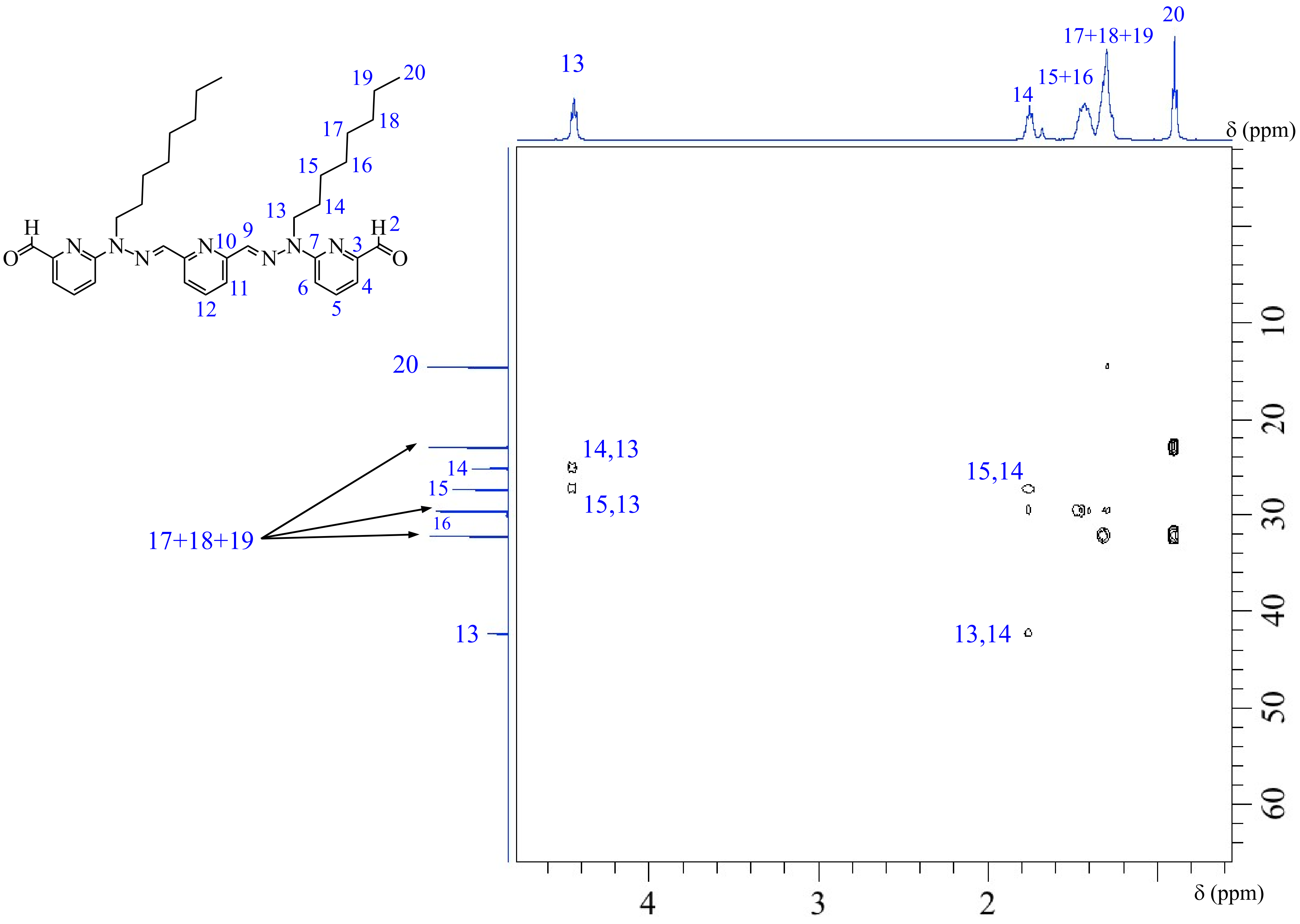

NMR spectra of compound **R1**

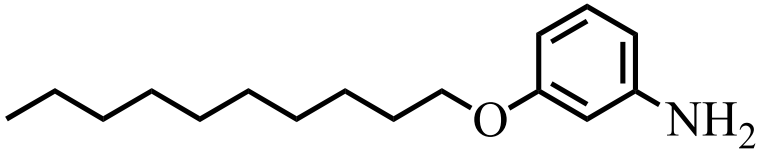

^{1}H NMR spectrum (500 MHz, $CDCl_3$, δ_{ref} = 7.26 ppm) of compound **R1**

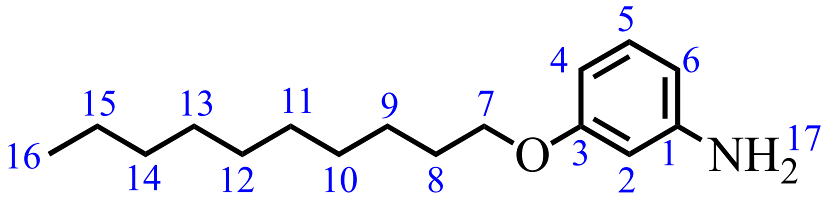

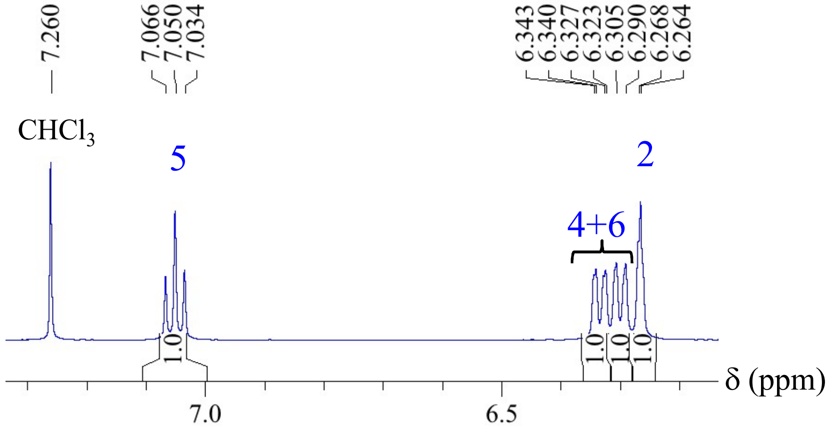

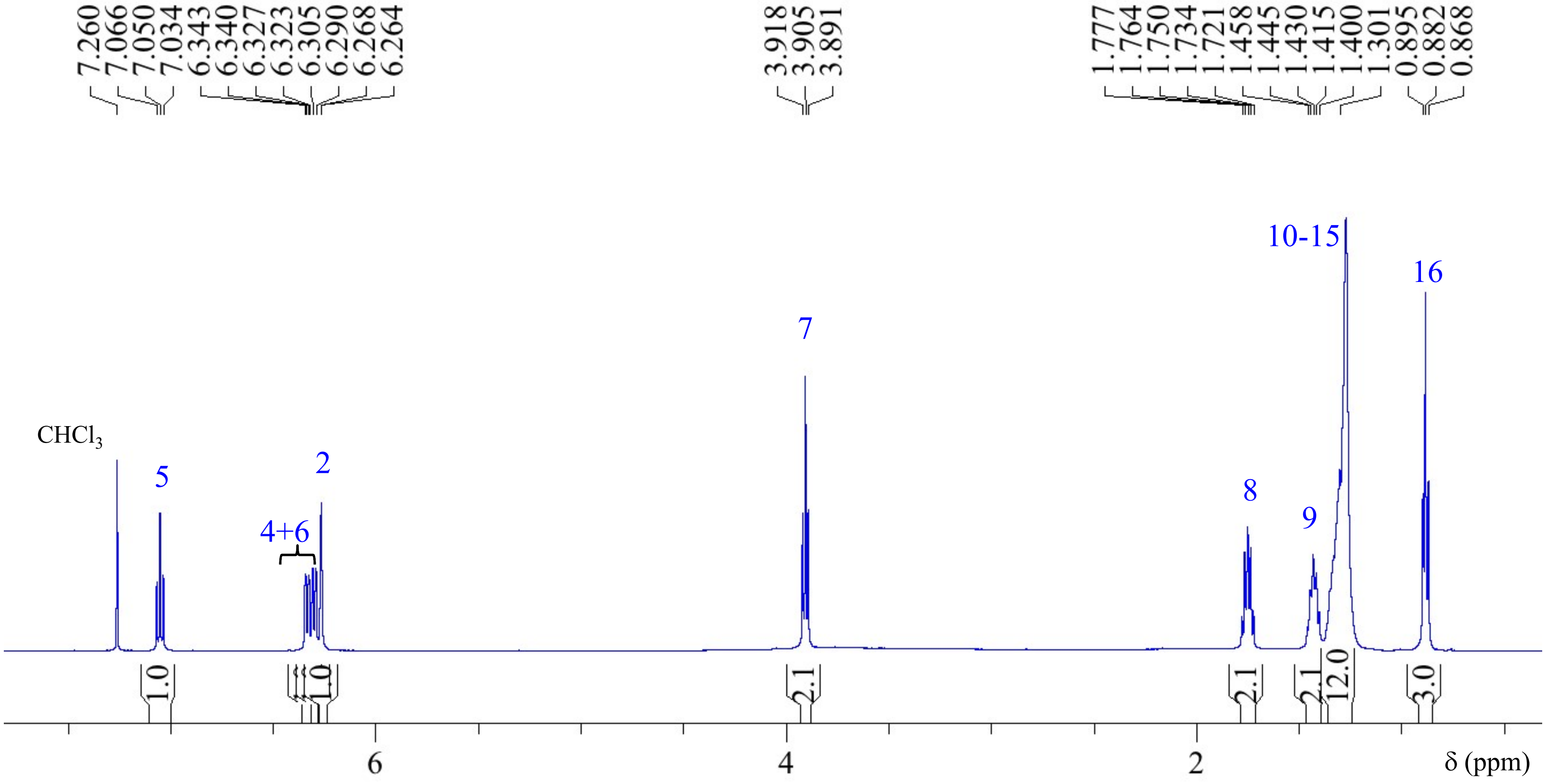

^{13}C NMR spectrum (125 MHz, $CDCl_3$, δ_{ref} = 77.4 ppm) of compound **R1**

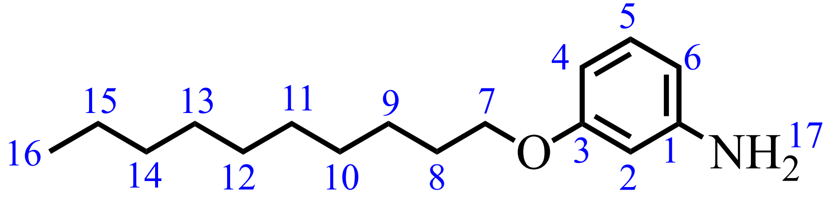

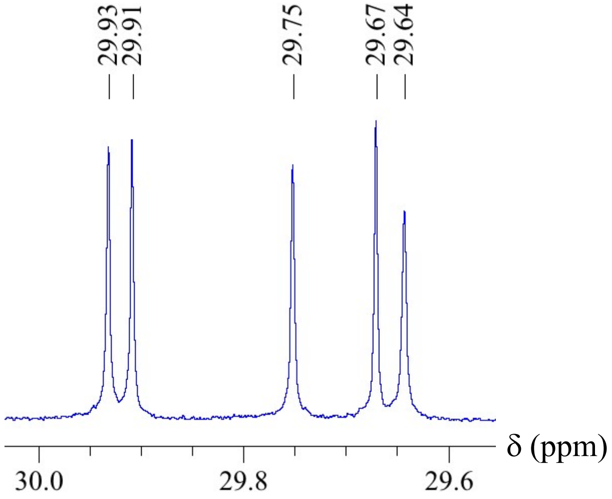

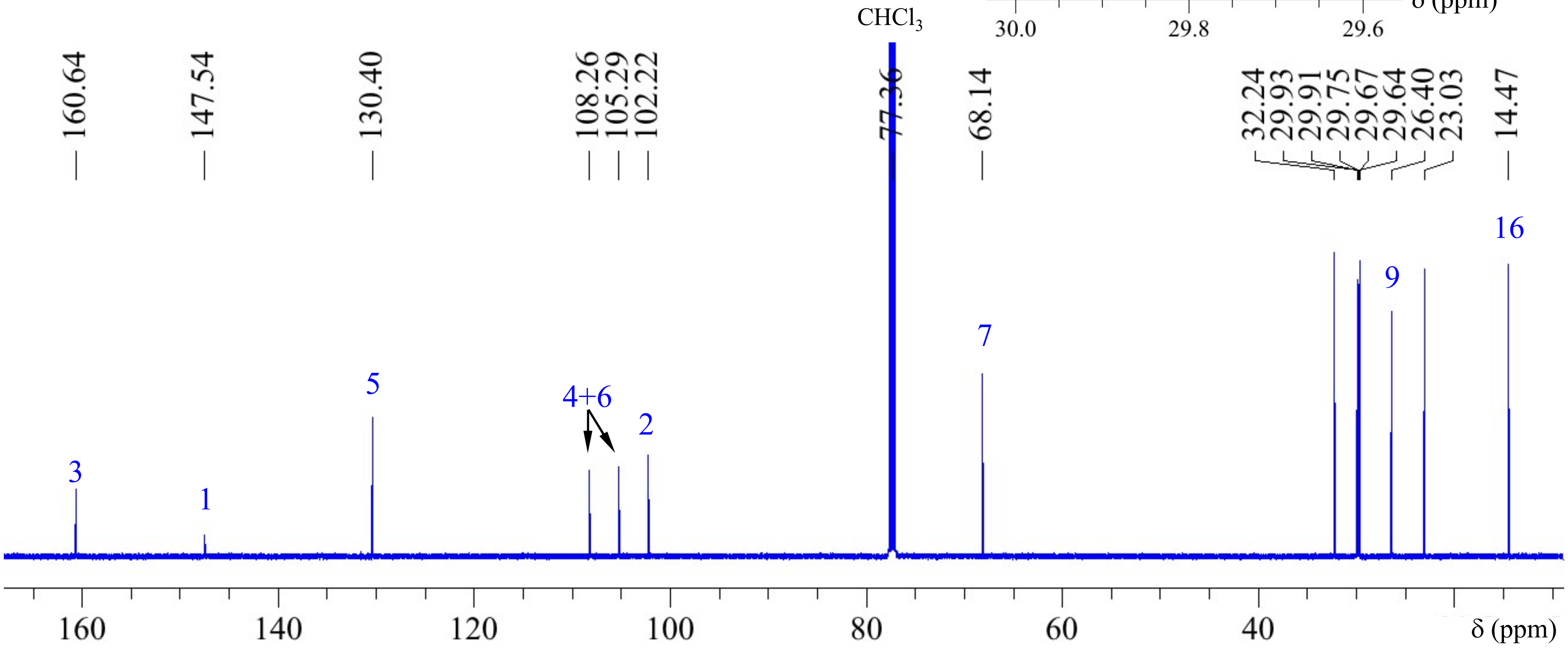

^{1}H-^{1}H COSY NMR spectrum (500 MHz, $CDCl_3$) of compound **R1**

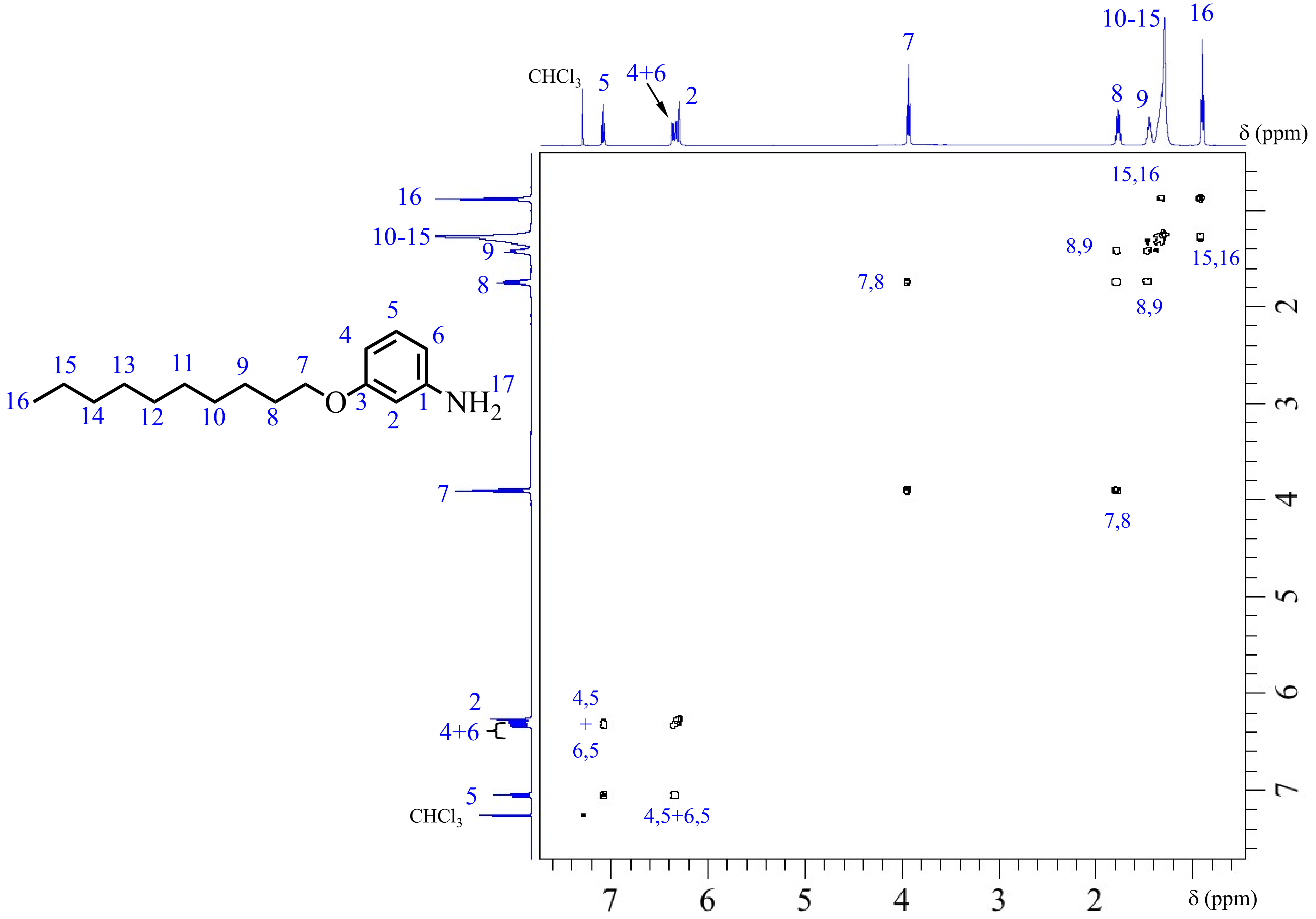

^{1}H-^{13}C HSQC NMR spectrum (500 MHz, $CDCl_3$) of compound **R1**

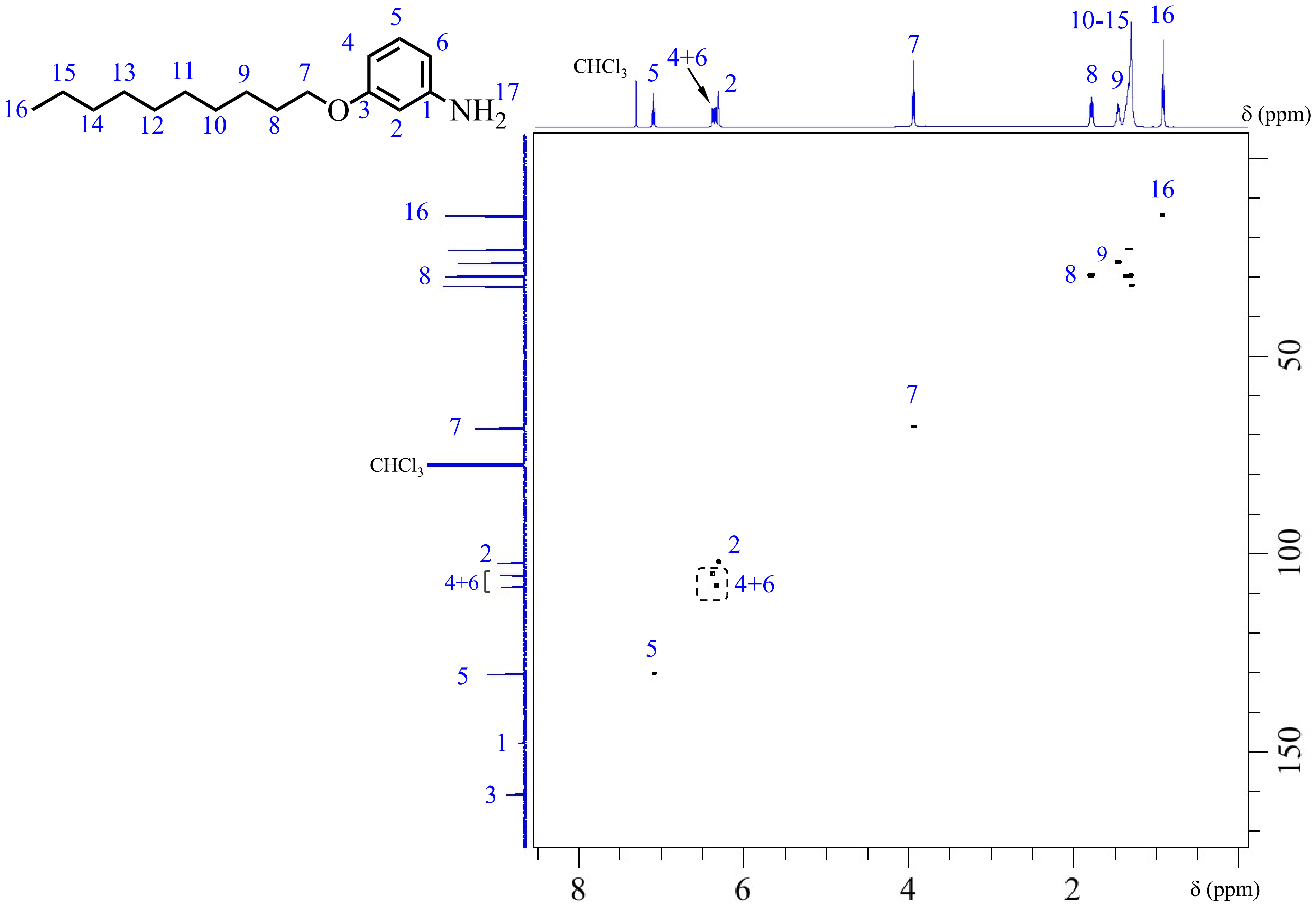

Part of the ^{1}H-^{13}C HSQC NMR spectrum (500 MHz, $CDCl_3$) of compound **R1**

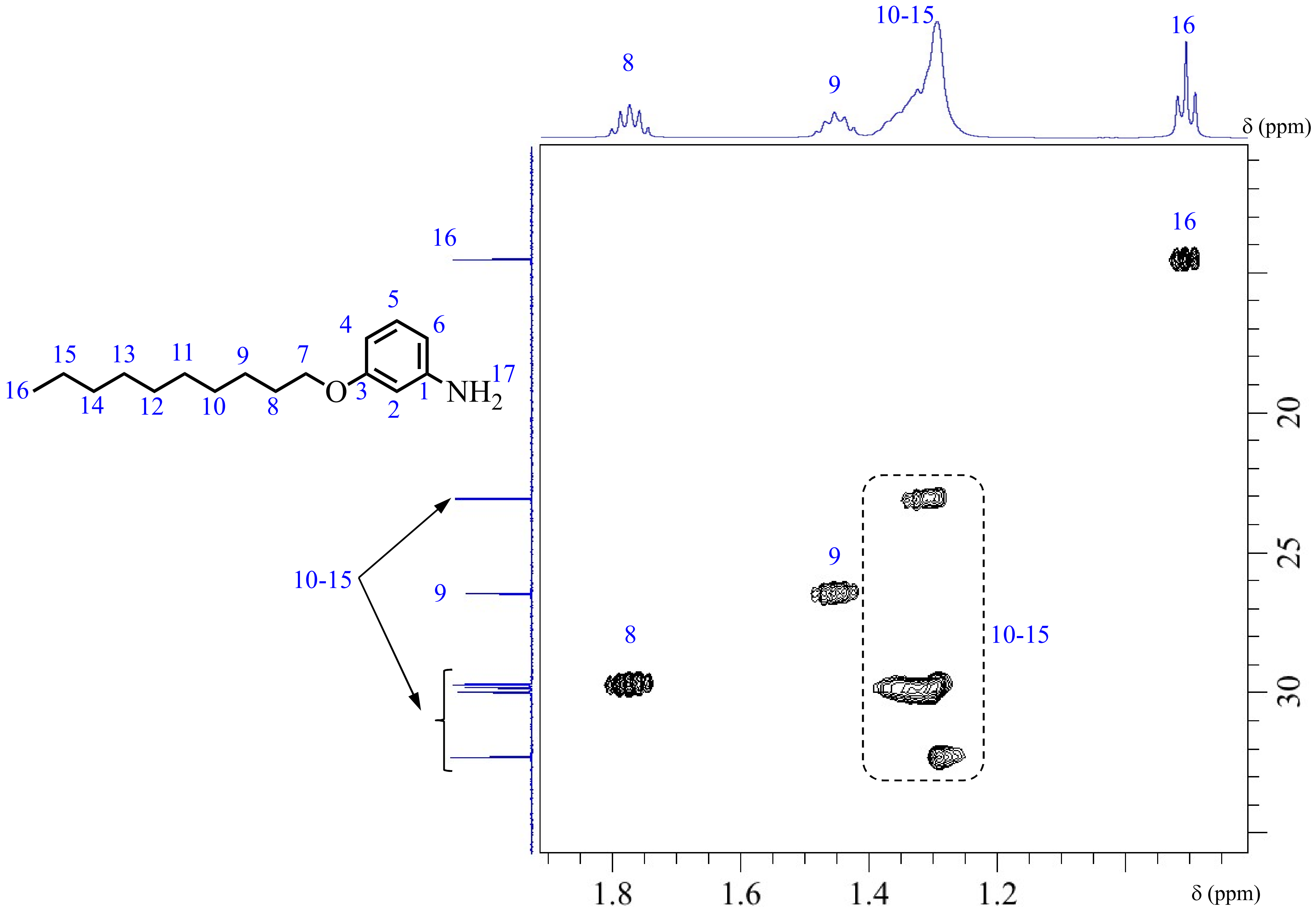

^{1}H-^{13}C HMBC NMR spectrum (500 MHz, $CDCl_3$) of compound **R1**

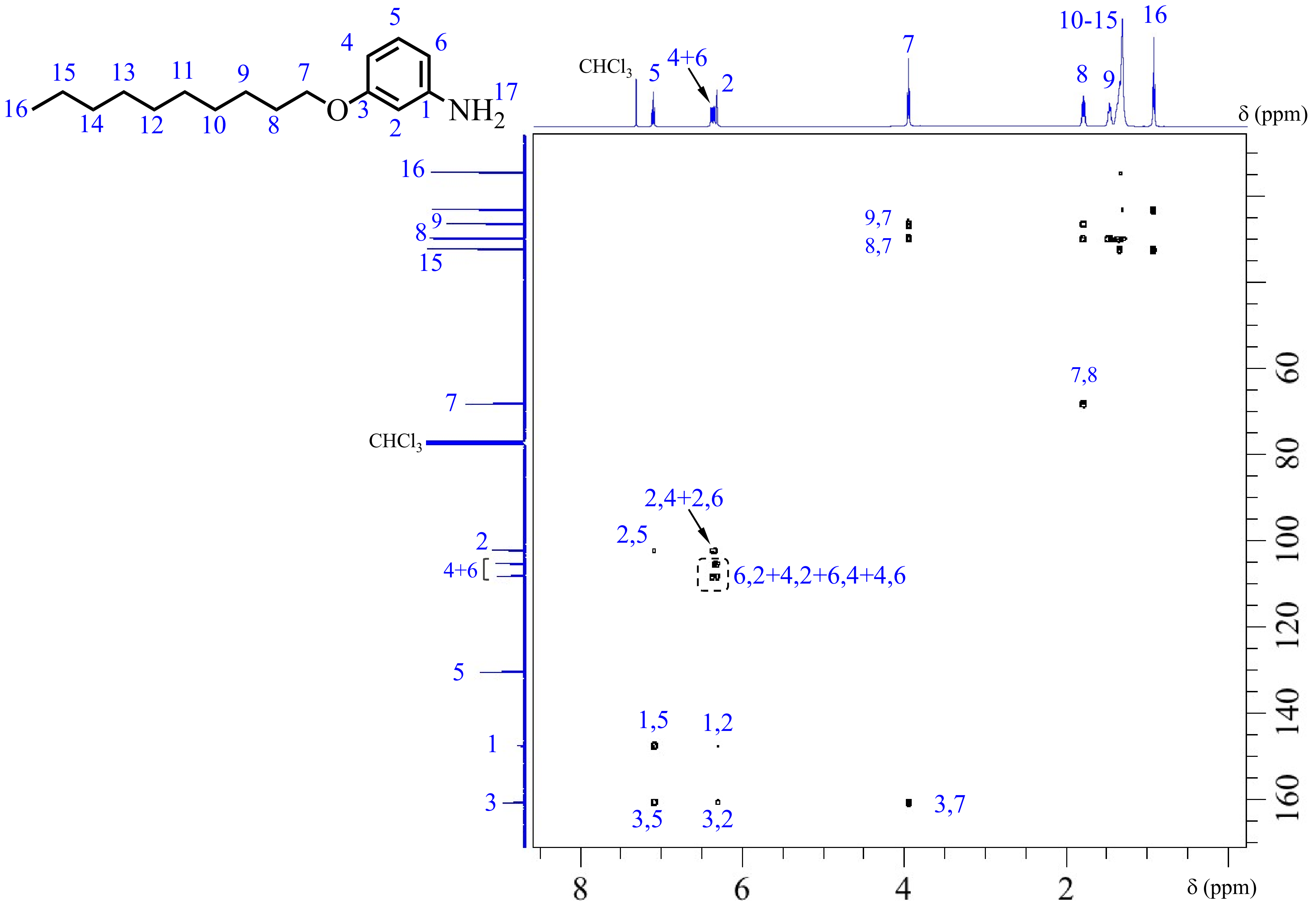

NMR spectra of compound **R2**

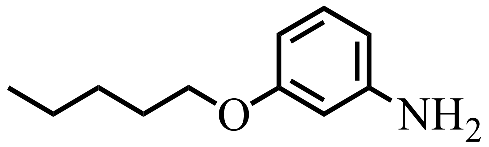

^{1}H NMR spectrum (500 MHz, $CDCl_3$, δ_{ref} = 7.26 ppm) of compound **R2**

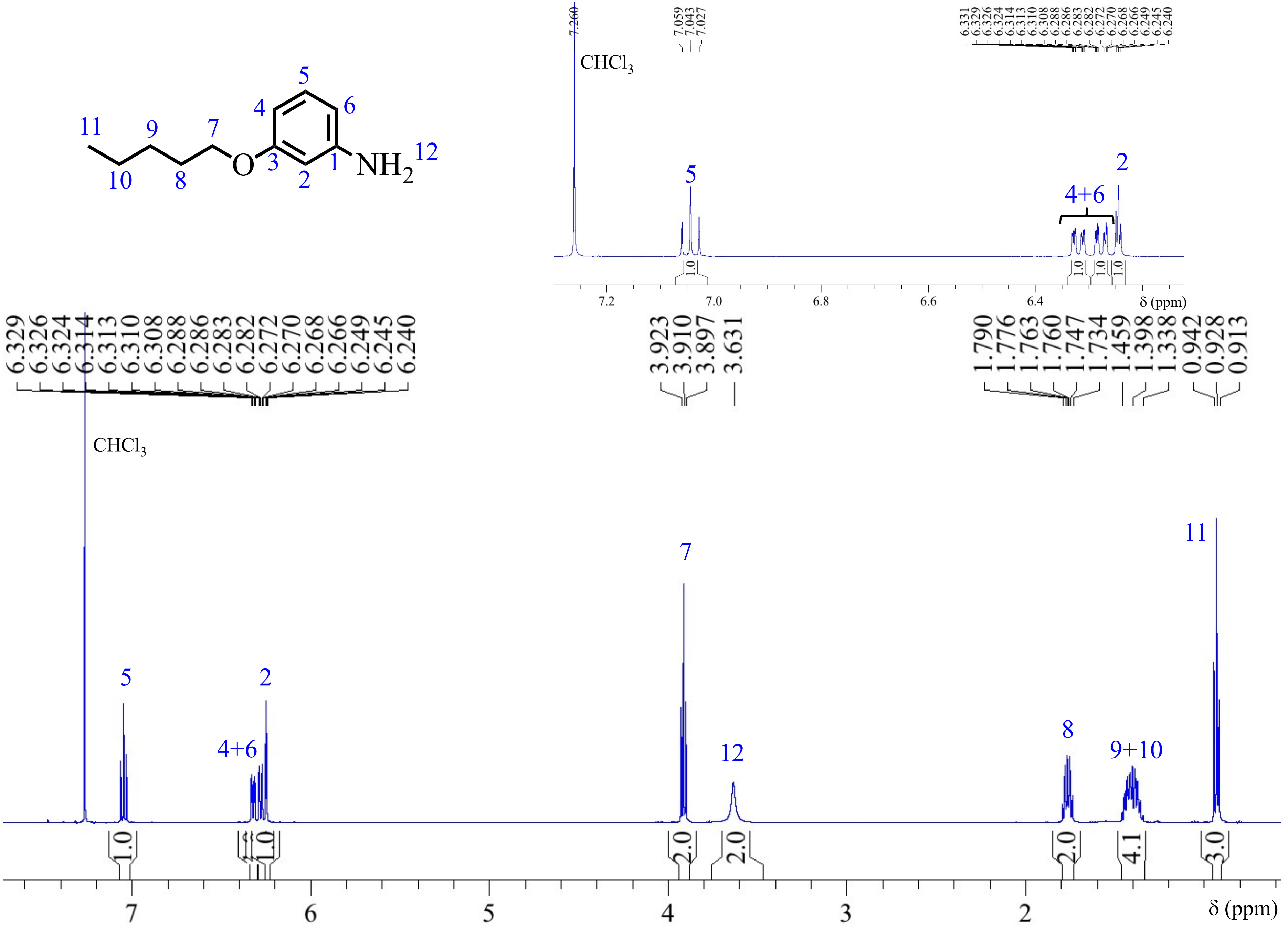

^{13}C NMR spectrum (125 MHz, $CDCl_3$, δ_{ref} = 77.4 ppm) of compound **R2**

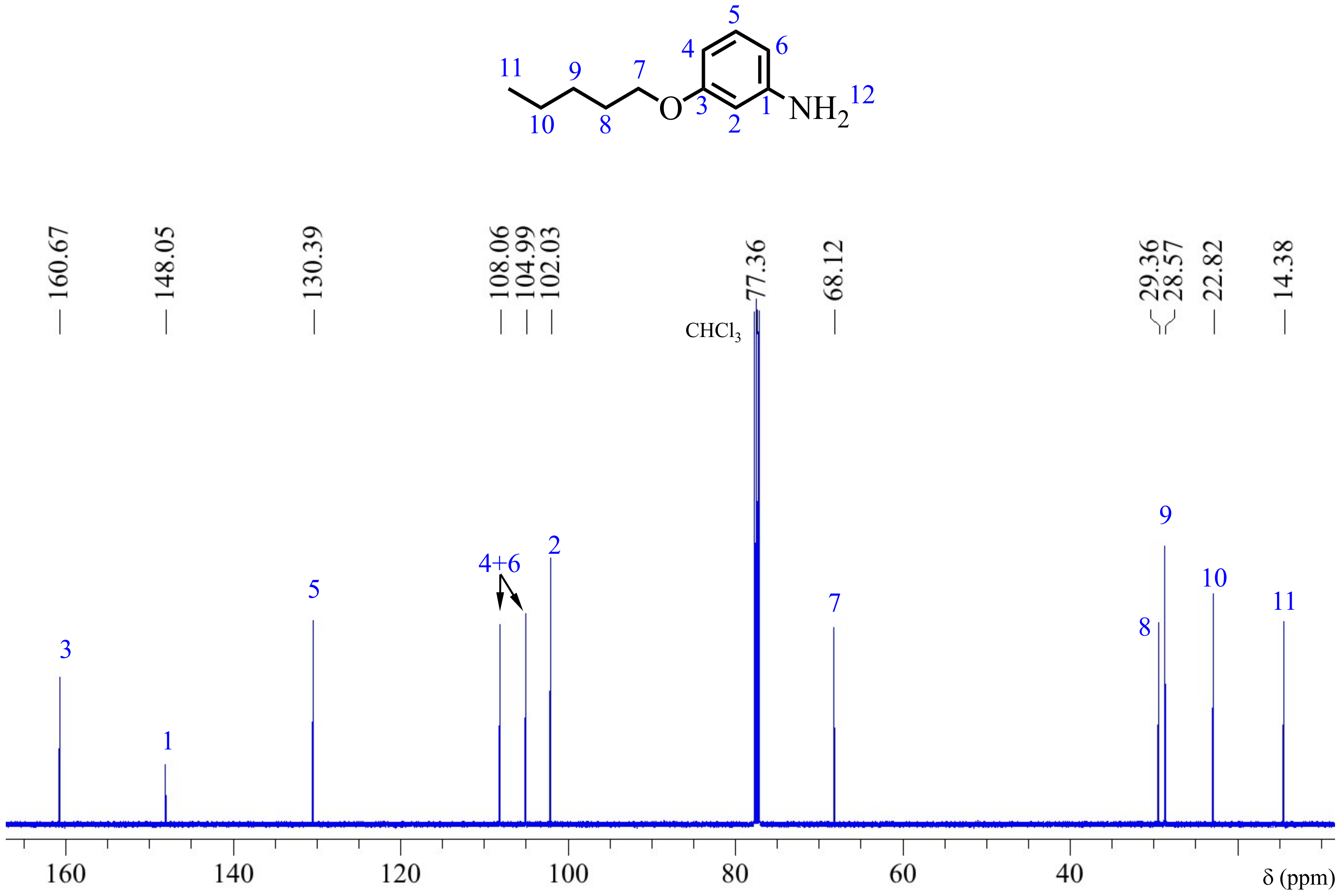

^{1}H-^{1}H COSY NMR spectrum (500 MHz, $CDCl_3$) of compound **R2**

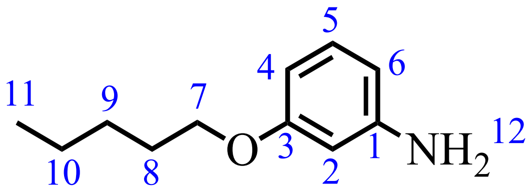

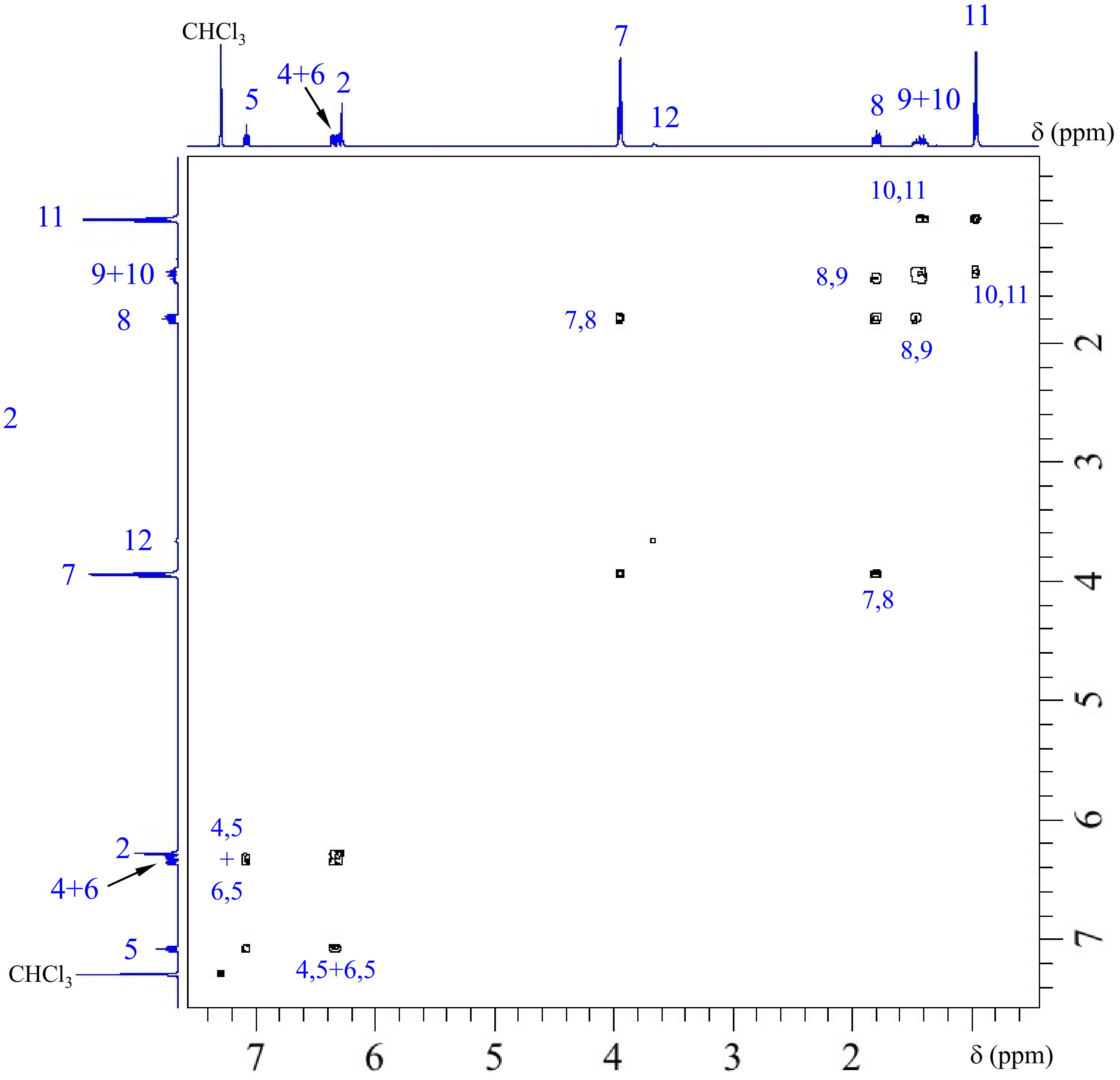

^{1}H-^{13}C HSQC NMR spectrum (500 MHz, $CDCl_3$) of compound **R2**

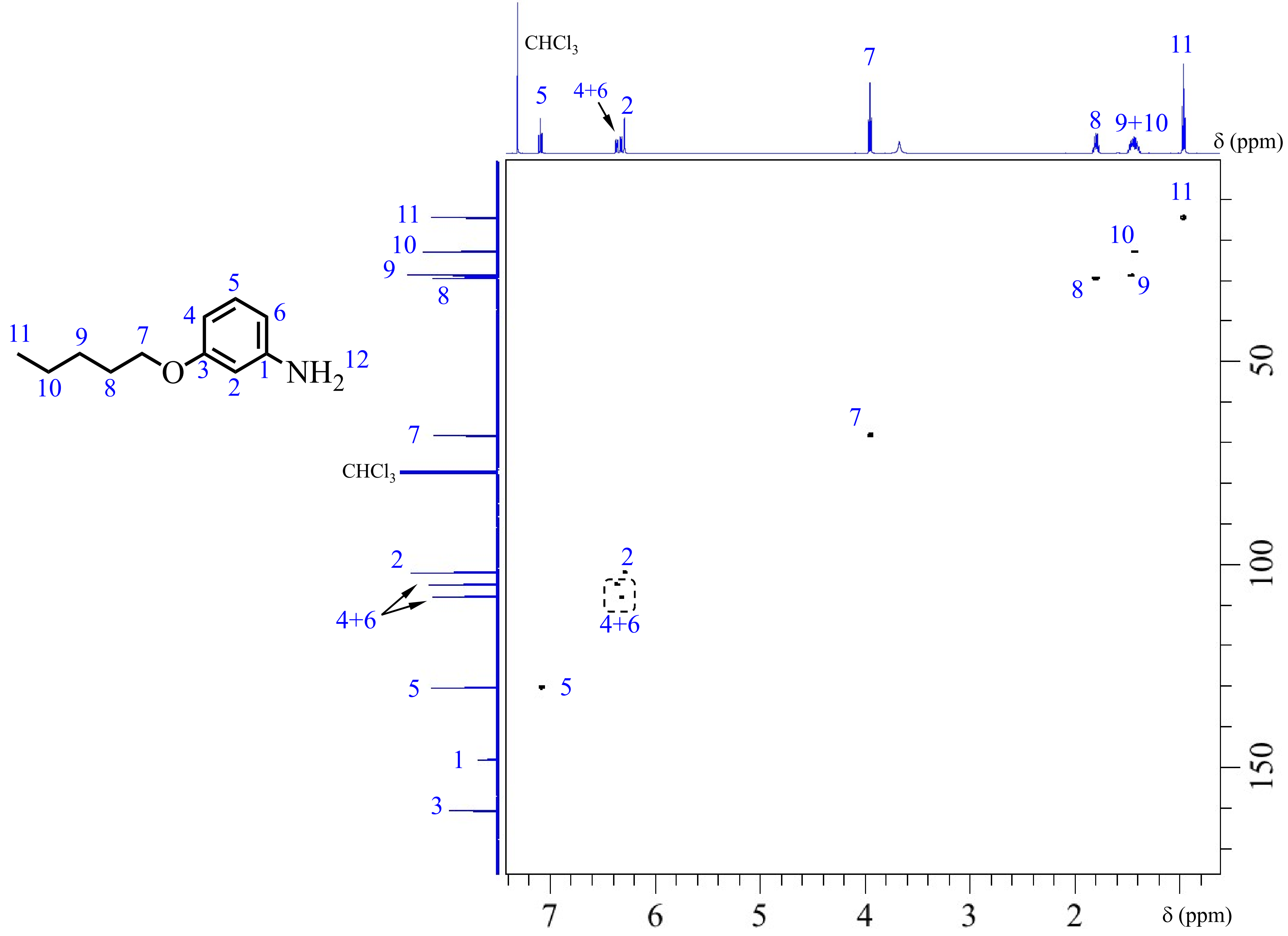

^{1}H-^{13}C HMBC NMR spectrum (500 MHz, $CDCl_3$) of compound **R2**

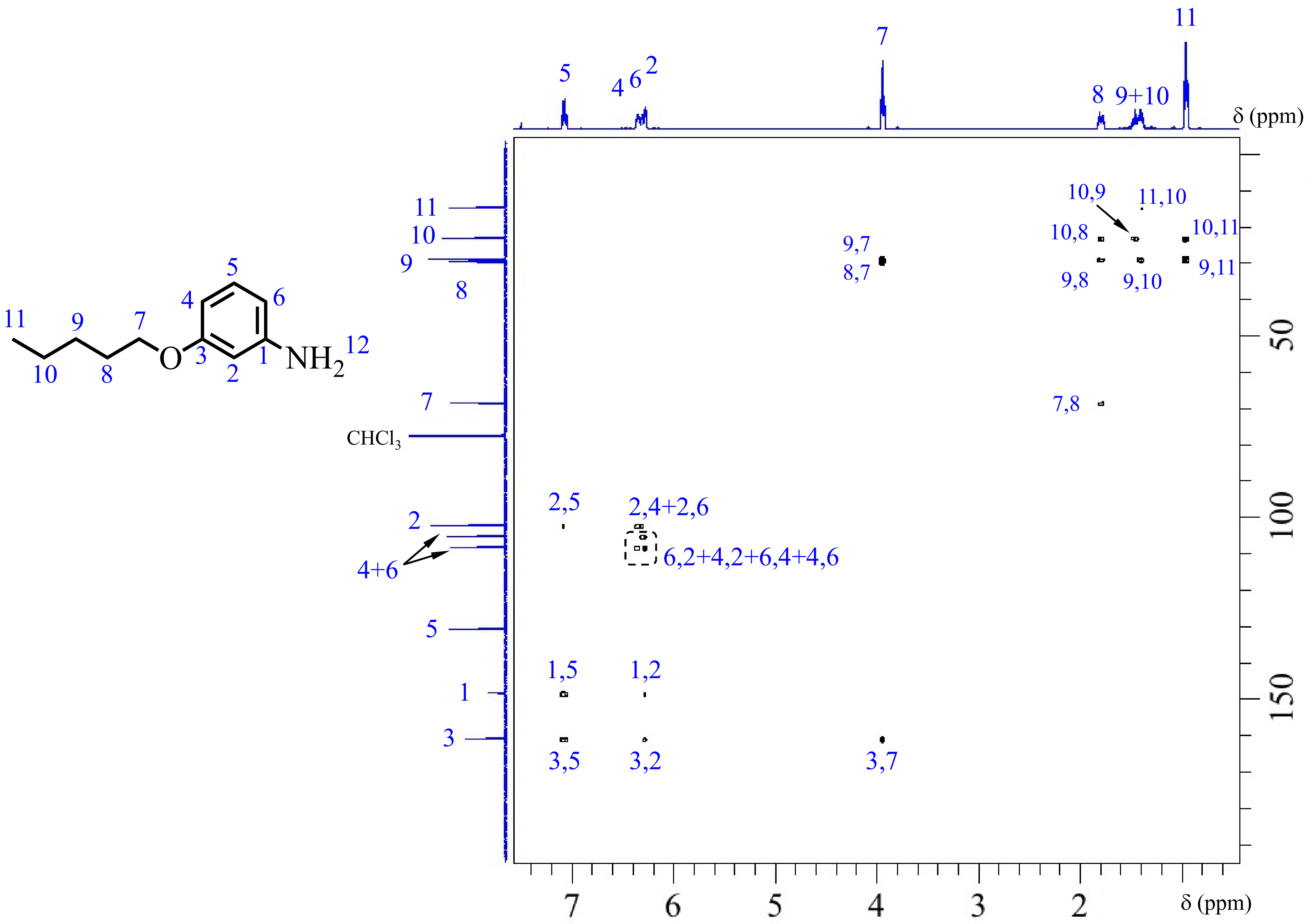

NMR spectra of compound **R4**

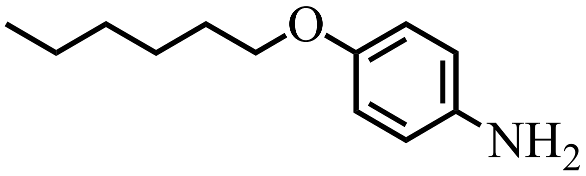

^{1}H NMR spectrum (500 MHz, $CDCl_3$, δ_{ref} = 7.26 ppm) of compound **R4**

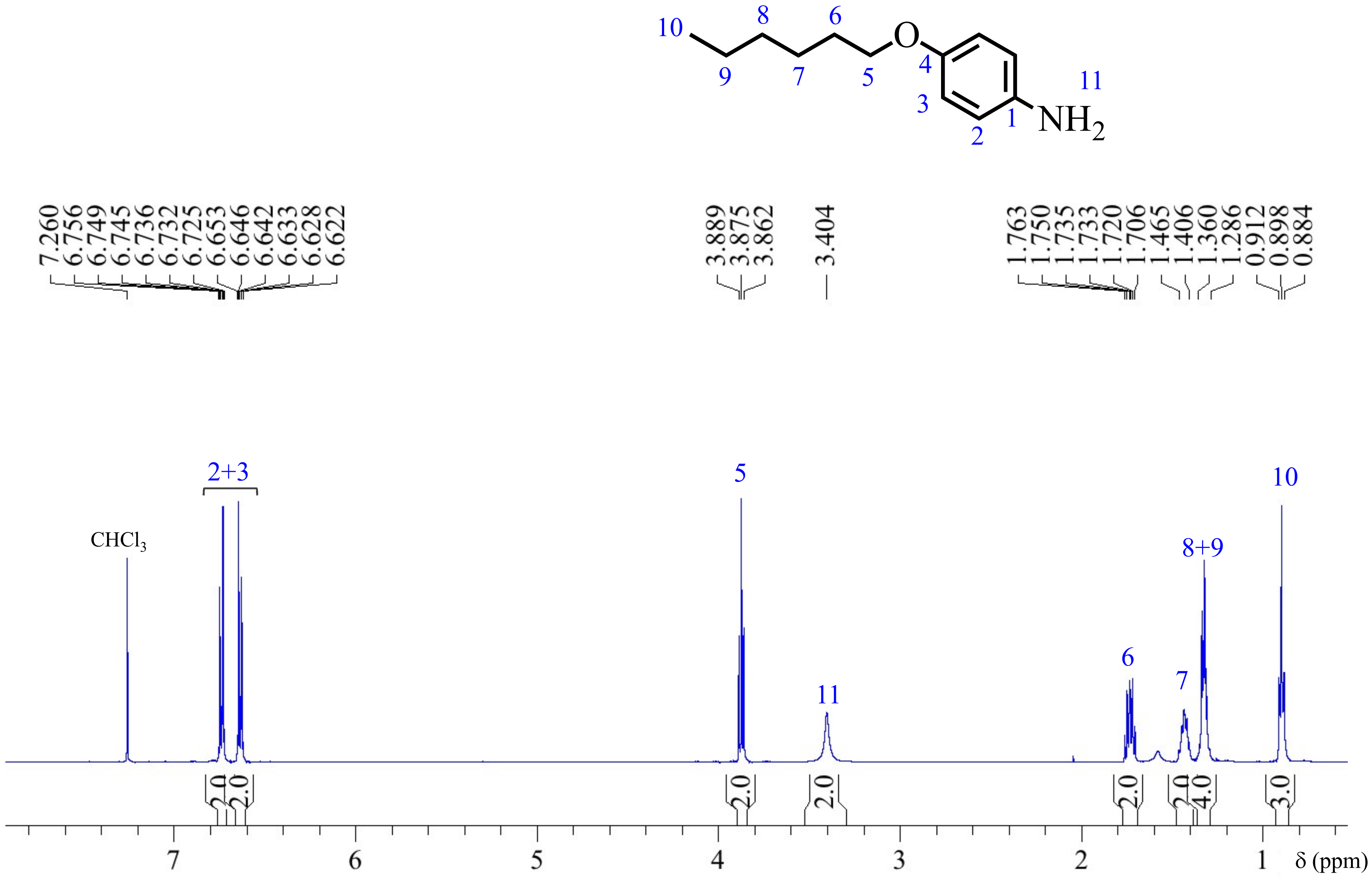

^{13}C NMR spectrum (125 MHz, $CDCl_3$, δ_{ref} = 77.4 ppm) of compound **R4**

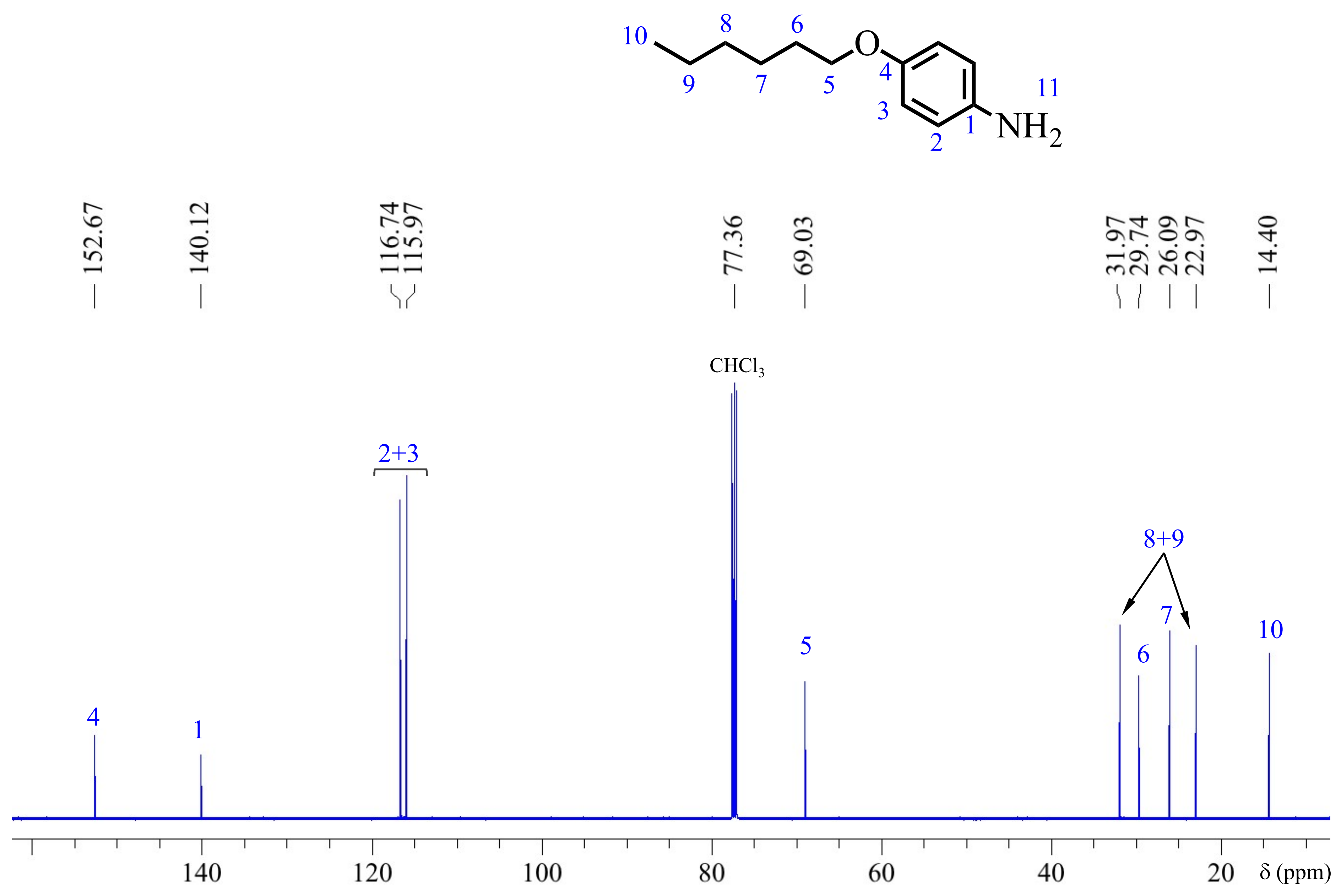

^{1}H-^{1}H COSY NMR spectrum (500 MHz, $CDCl_3$) of compound **R4**

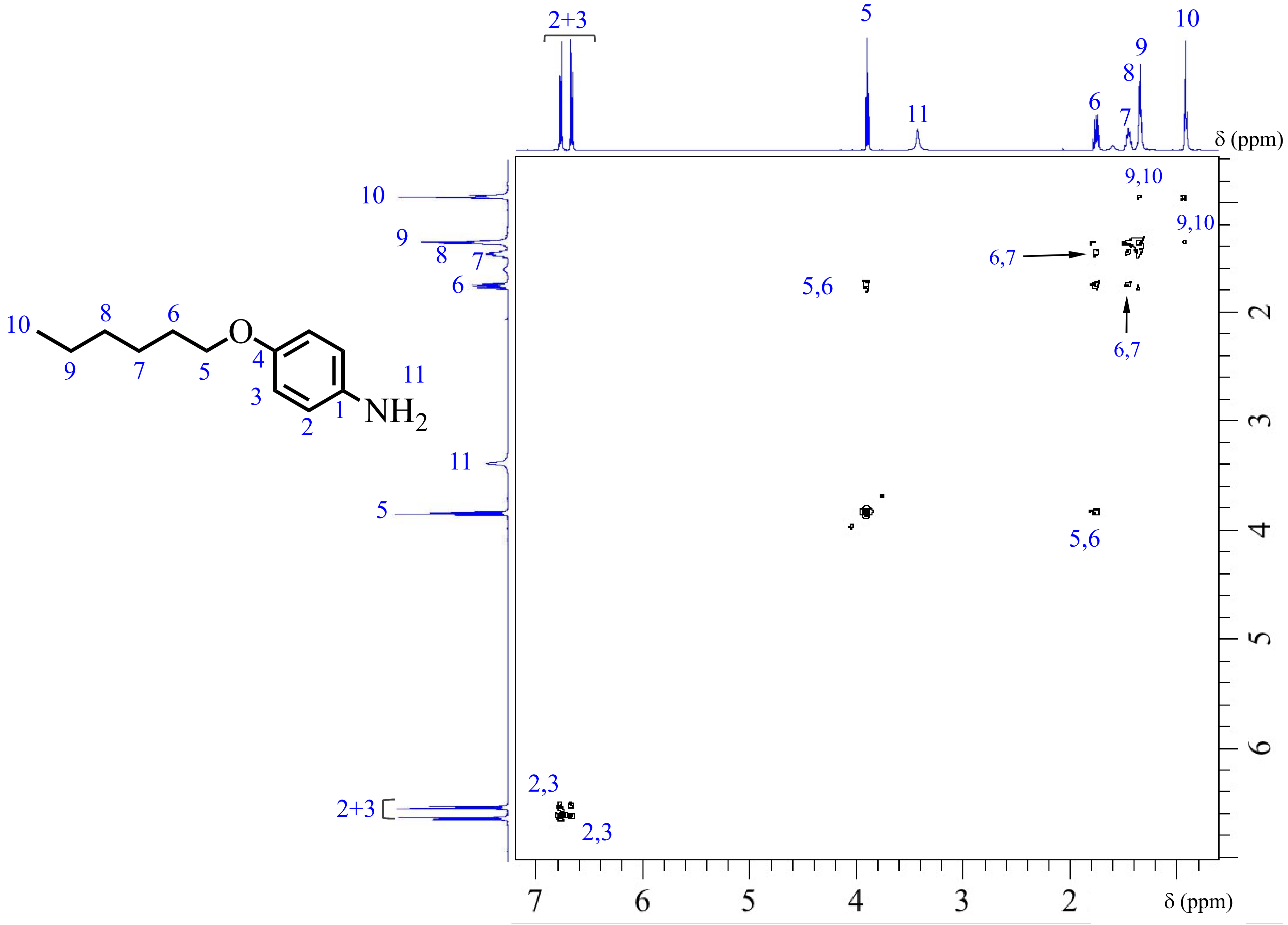

^{1}H-^{1}H HSQC NMR spectrum (500 MHz, $CDCl_3$) of compound **R4**

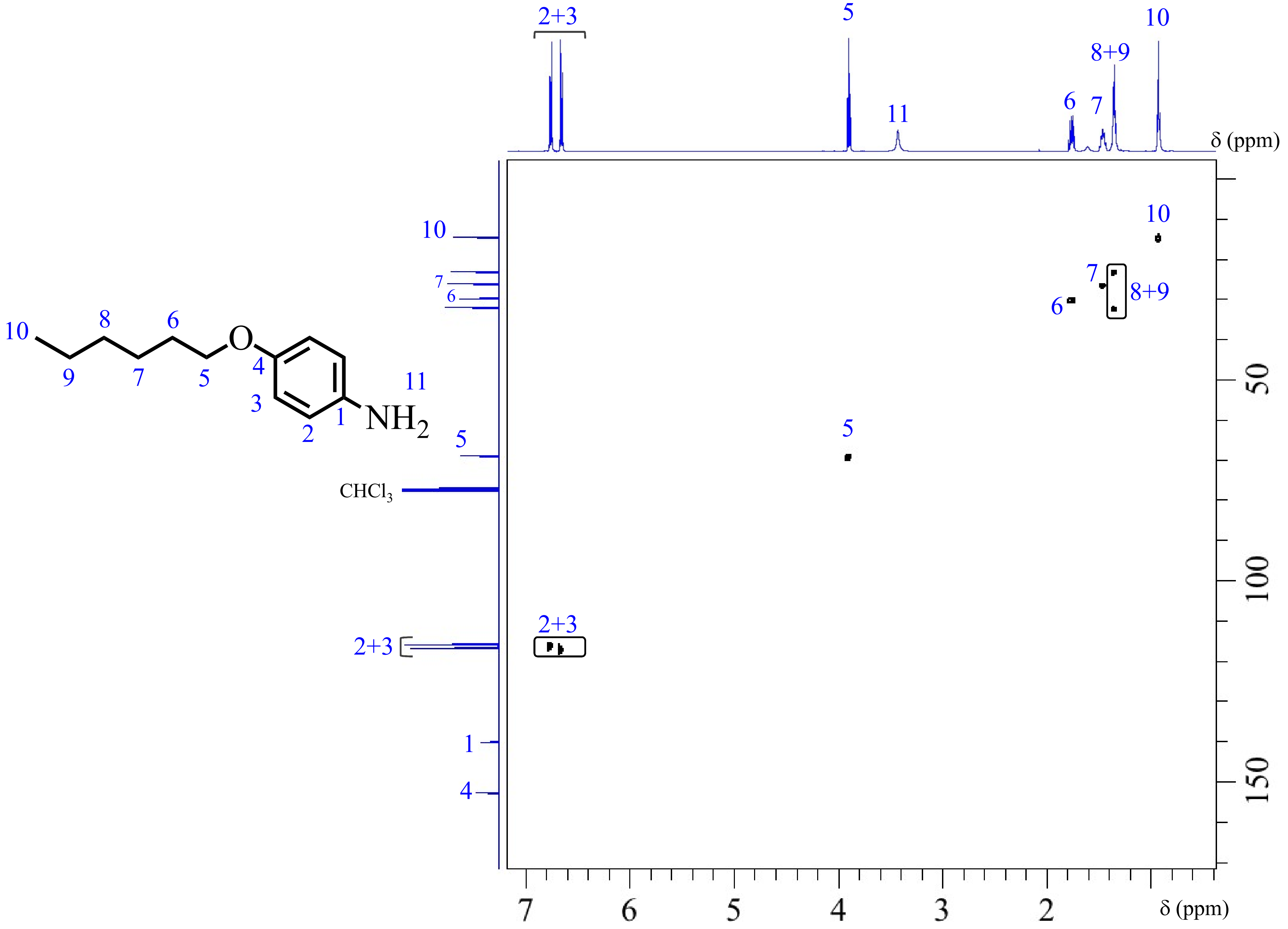

^{1}H-^{1}H HMBC NMR spectrum (500 MHz, $CDCl_3$) of compound **R4**

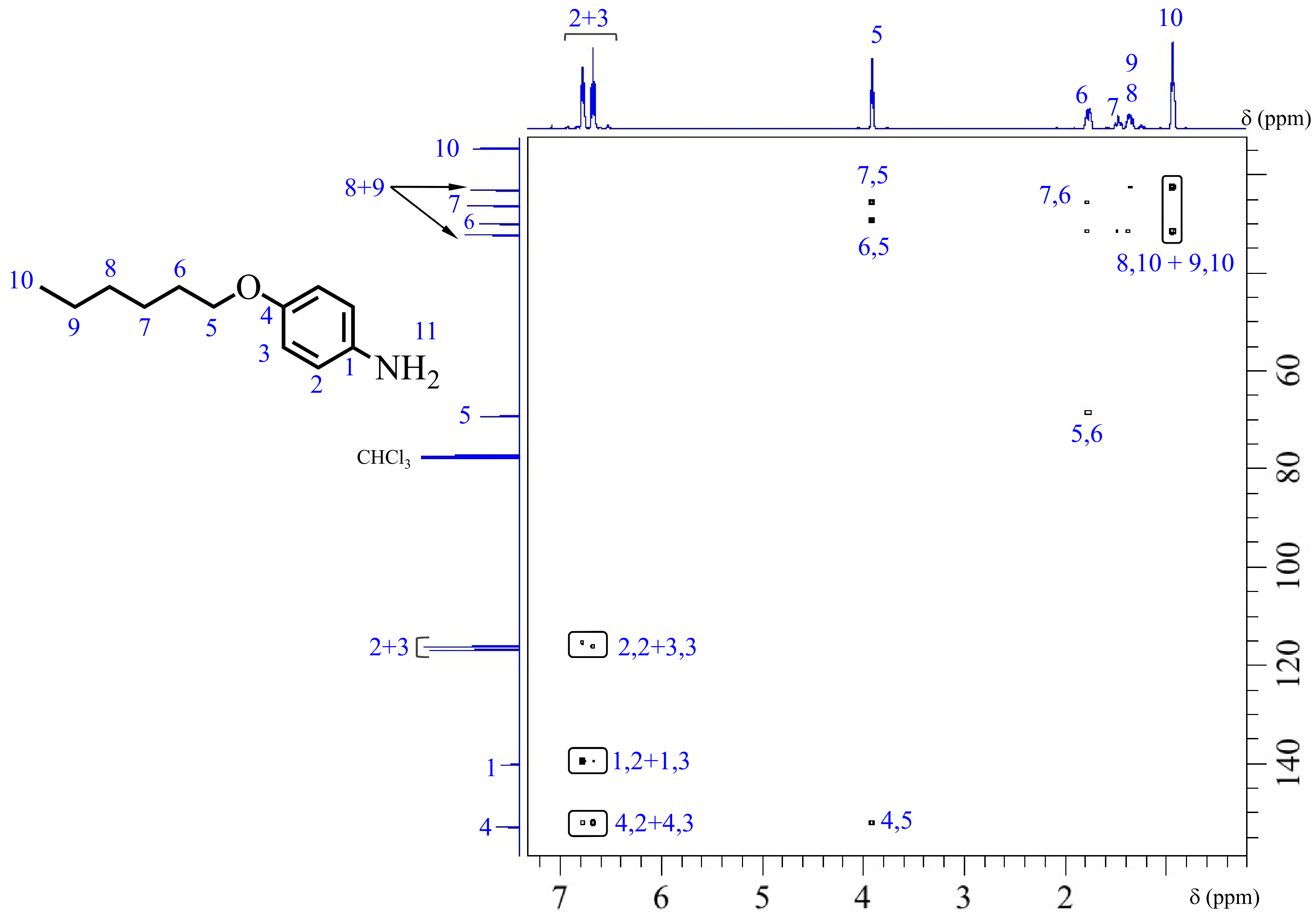

NMR spectra of compound **AR3$_2$**

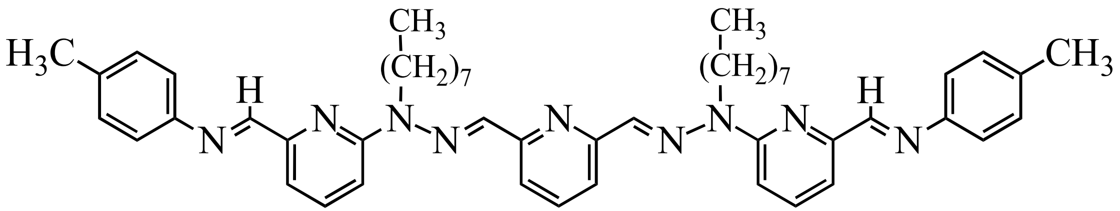

^{1}H NMR spectrum (400 MHz, $CDCl_3$, δ_{ref} = 7.26 ppm) of compound **$AR3_2$**

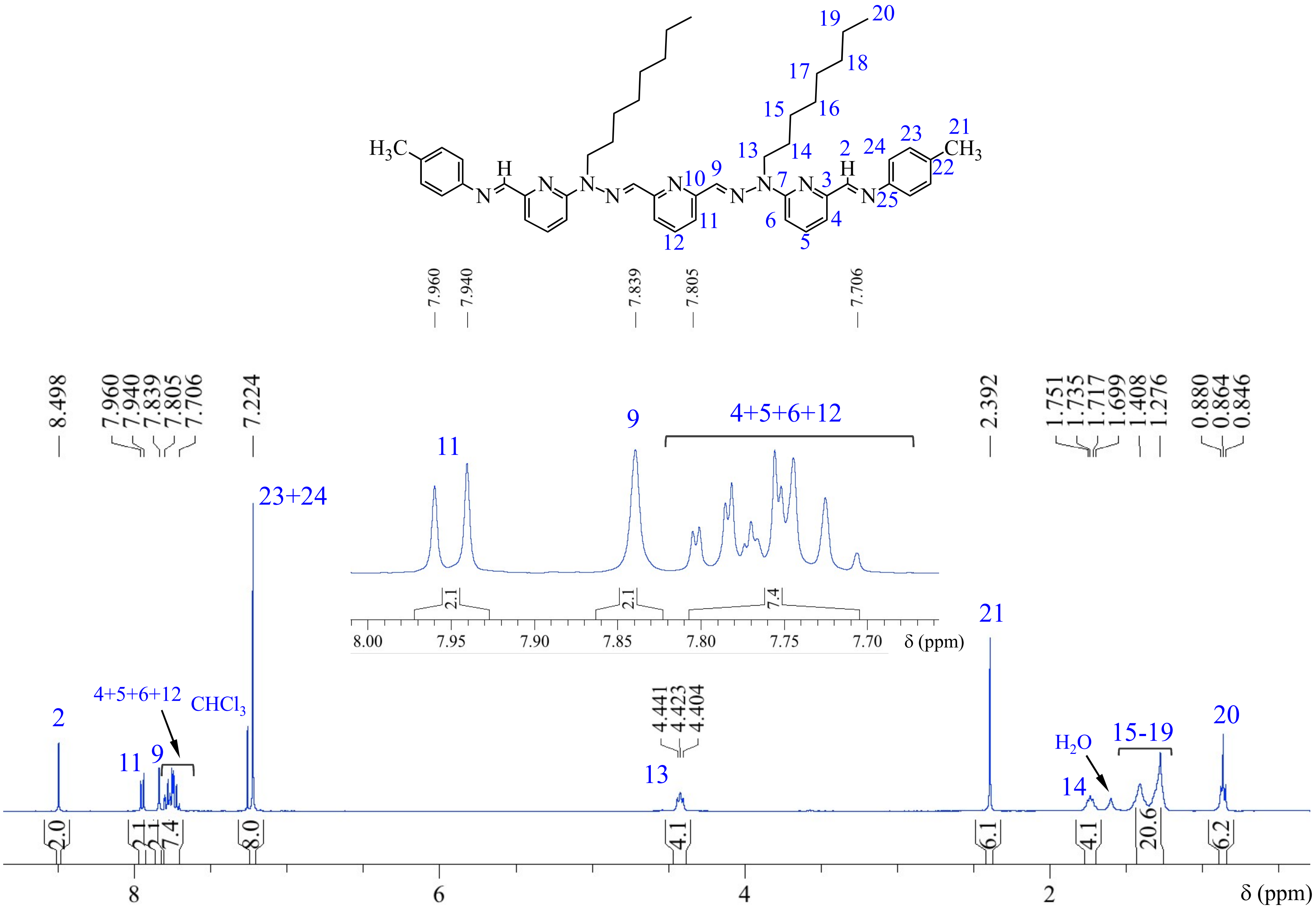

^{13}C NMR spectrum (125 MHz, $CDCl_3$, δ_{ref} = 77.4 ppm) of compound **AR3$_2$**

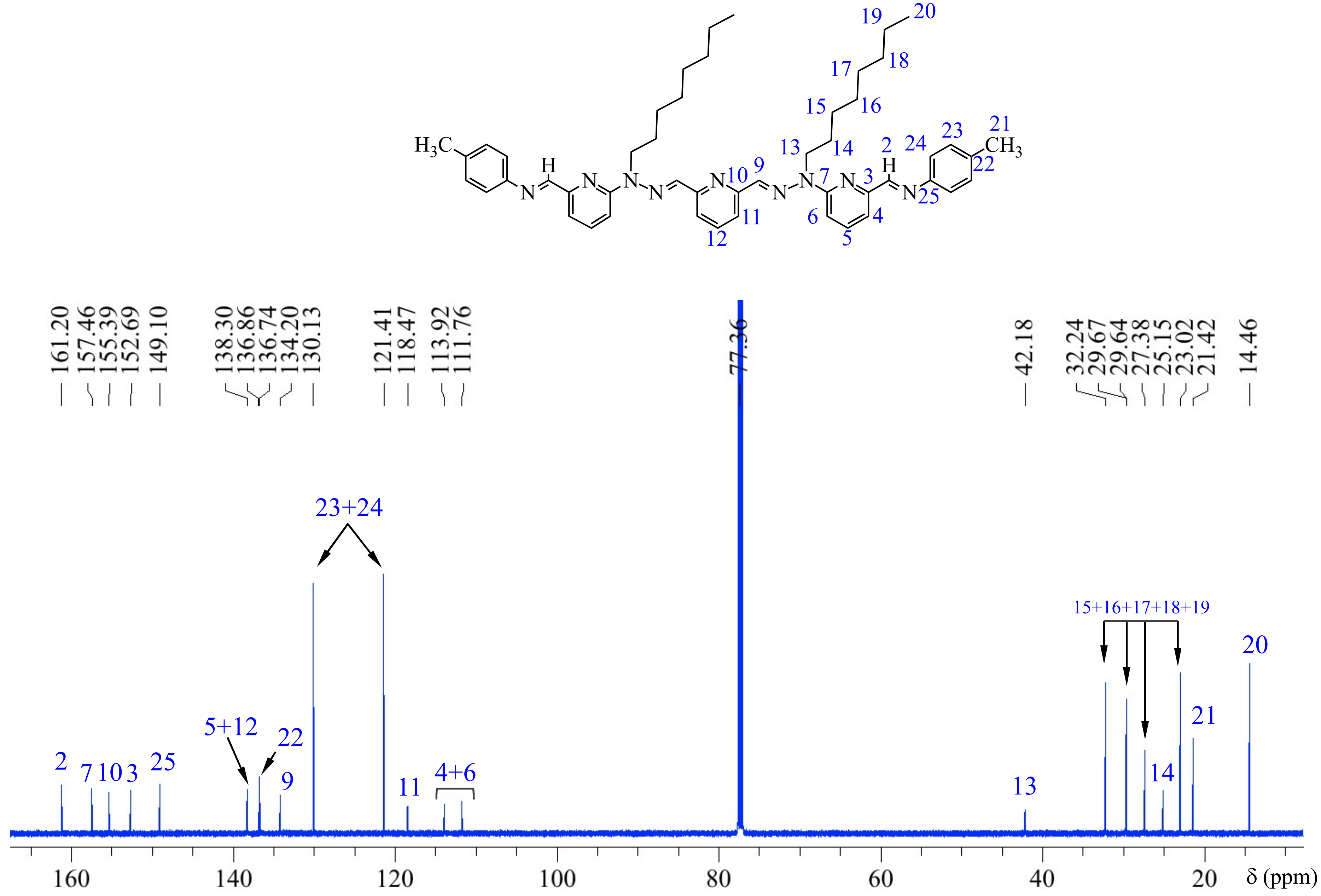

^1H-^1H COSY NMR spectrum (500 MHz, $CDCl_3$) of compound **$AR3_2$**

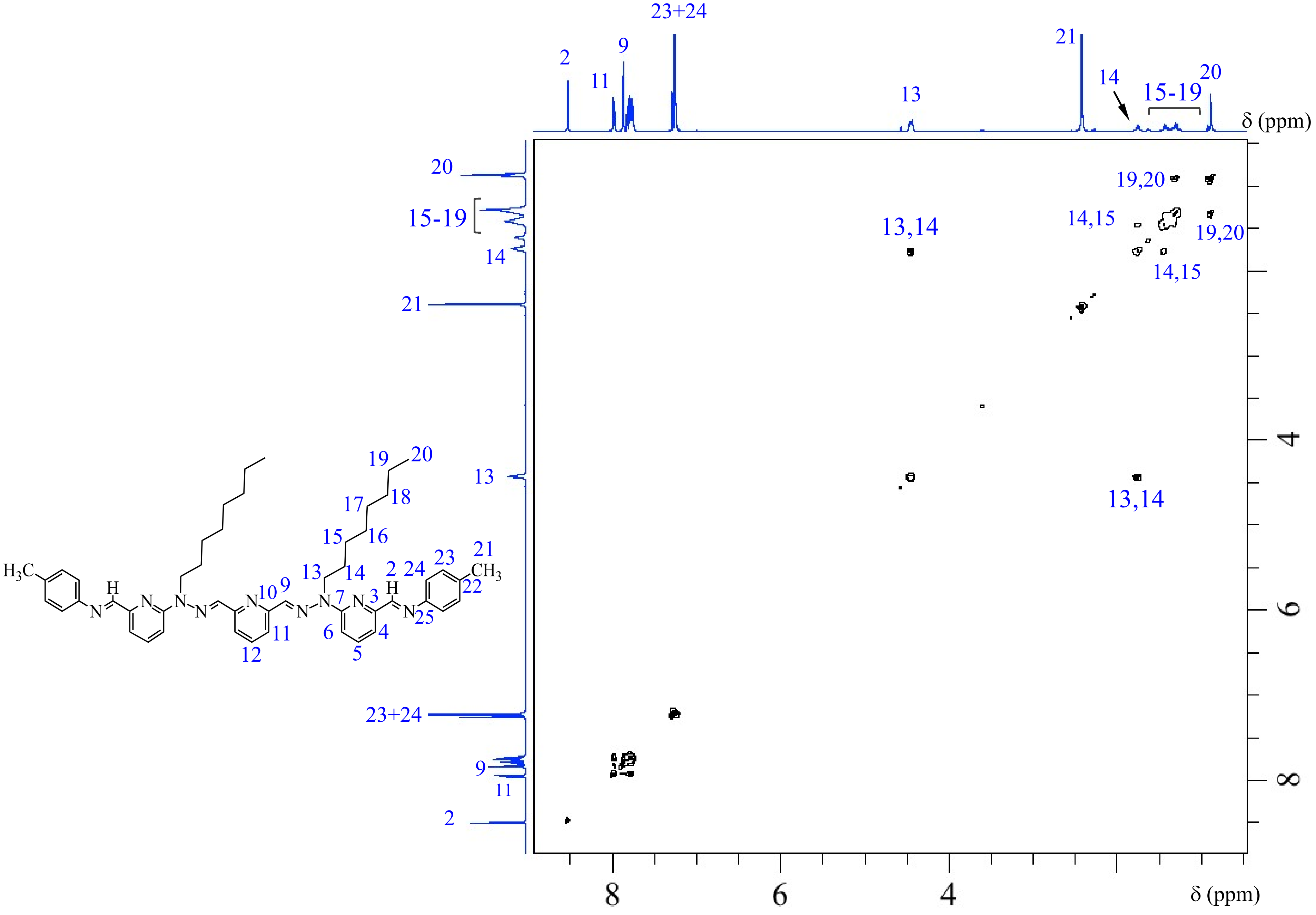

Part of the ^1H-^1H COSY NMR spectrum (500 MHz, $CDCl_3$) of compound **AR3$_2$**

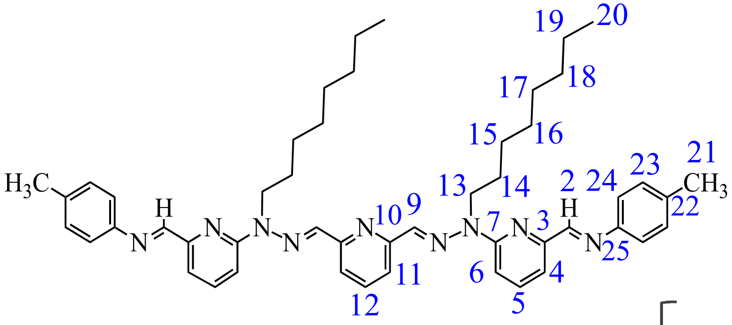

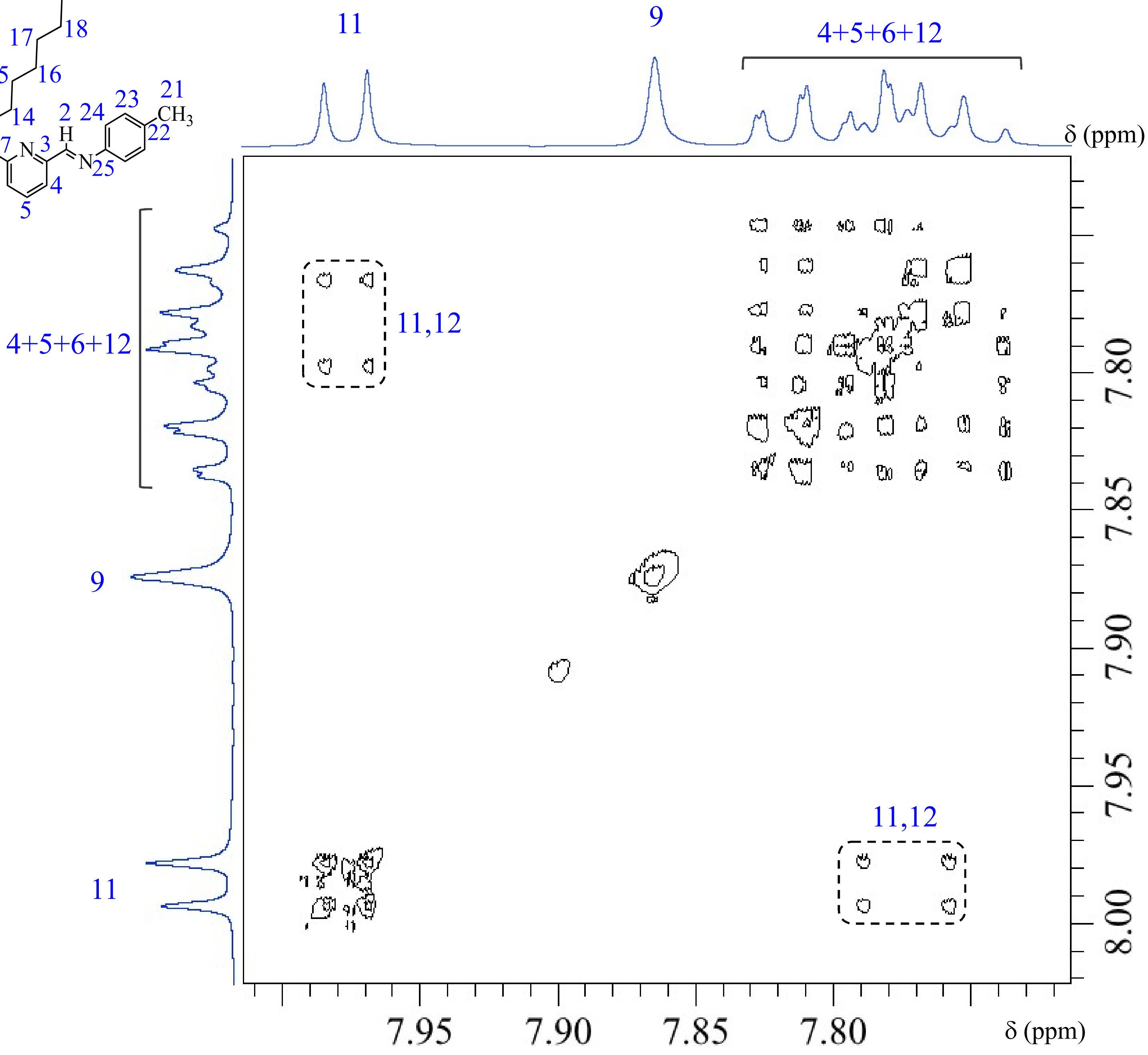

^{1}H-^{1}H ROESY NMR spectrum (500 MHz, $CDCl_3$) of compound **AR3$_2$**

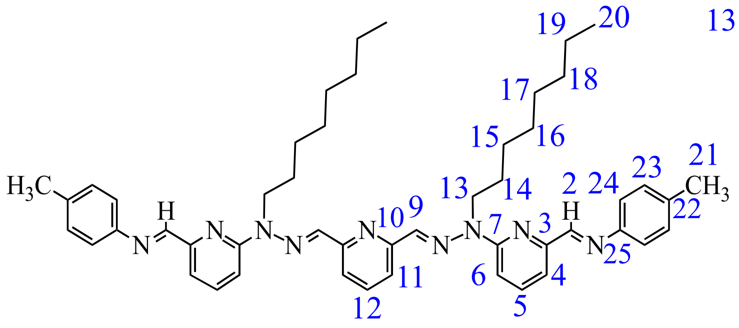

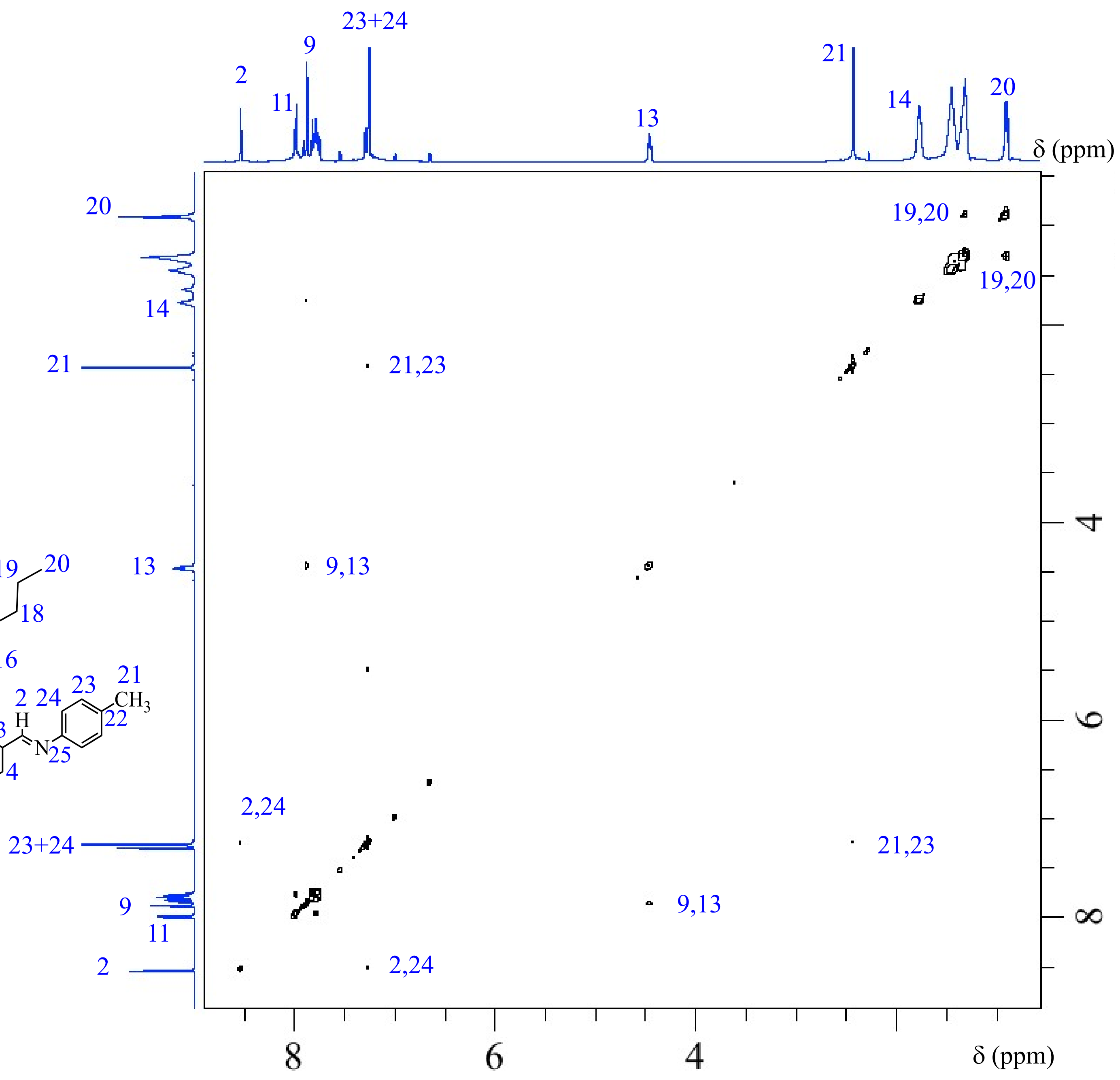

Part of the ^{1}H-^{1}H ROESY NMR spectrum (500 MHz, $CDCl_3$) of compound **AR3$_2$**

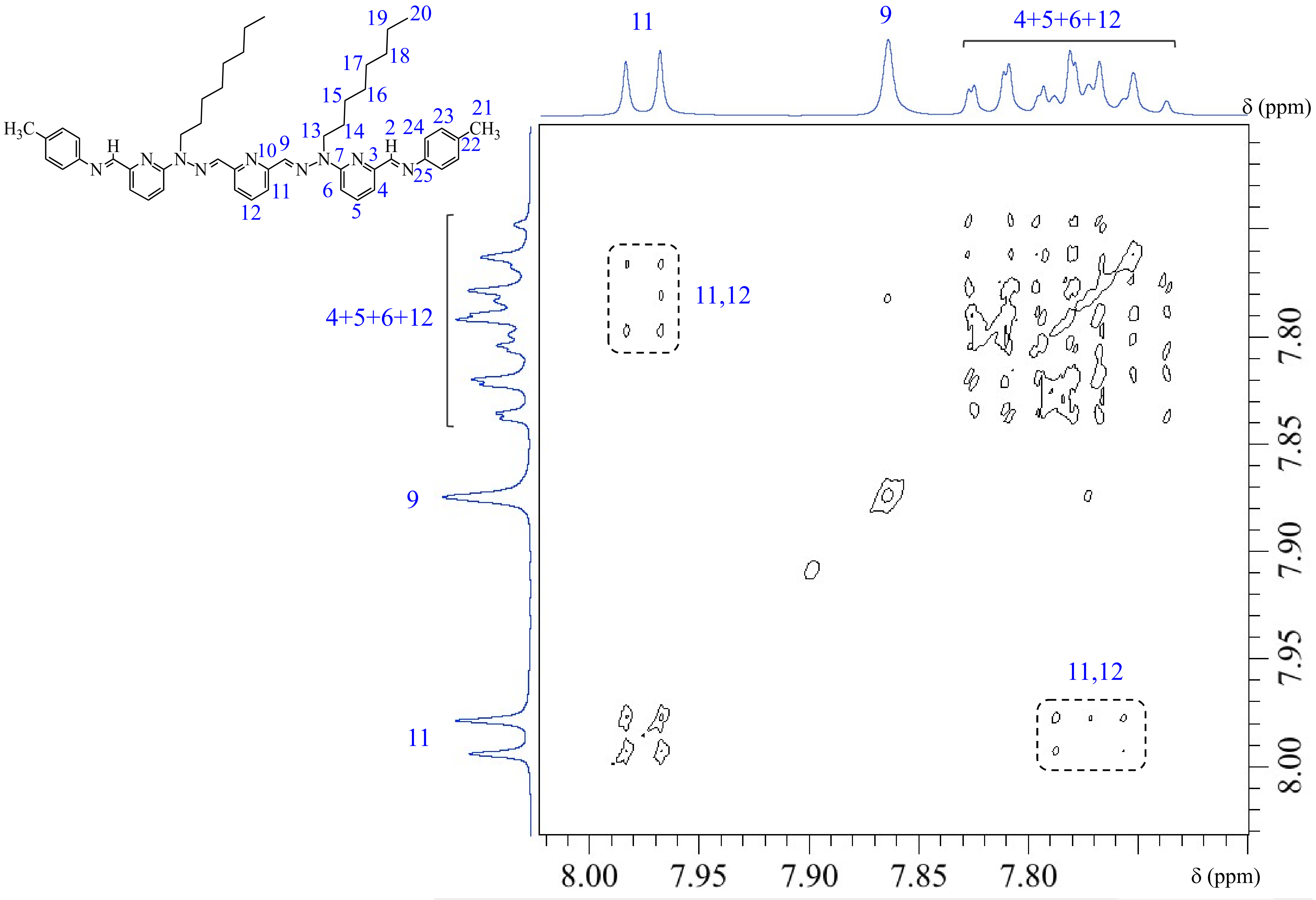

^{1}H-^{13}C HSQC NMR spectrum (500 MHz, $CDCl_3$) of compound **AR3$_2$**

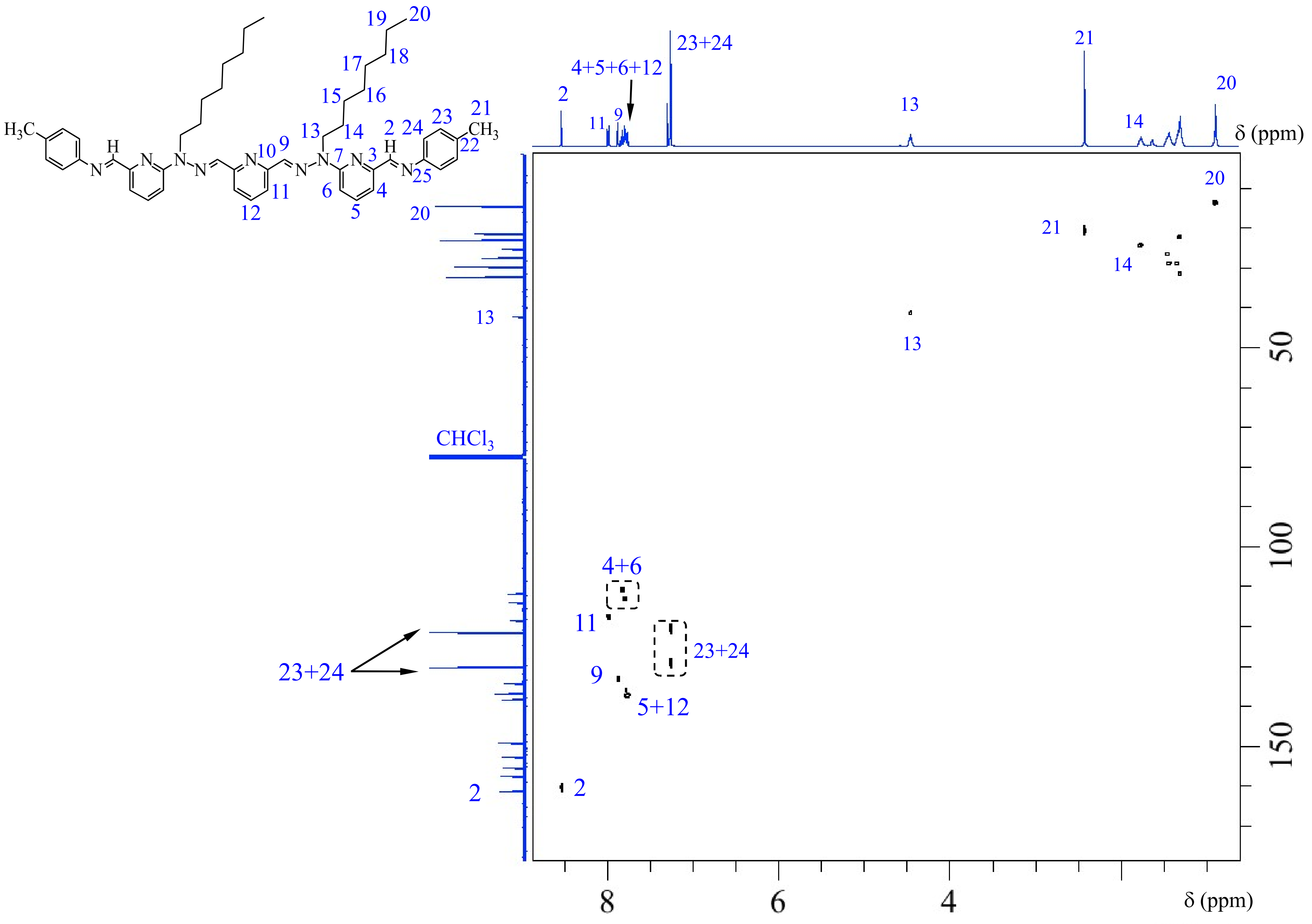

Part of the ^{1}H-^{13}C HSQC NMR spectrum (500 MHz, $CDCl_3$) of compound **AR3$_2$**

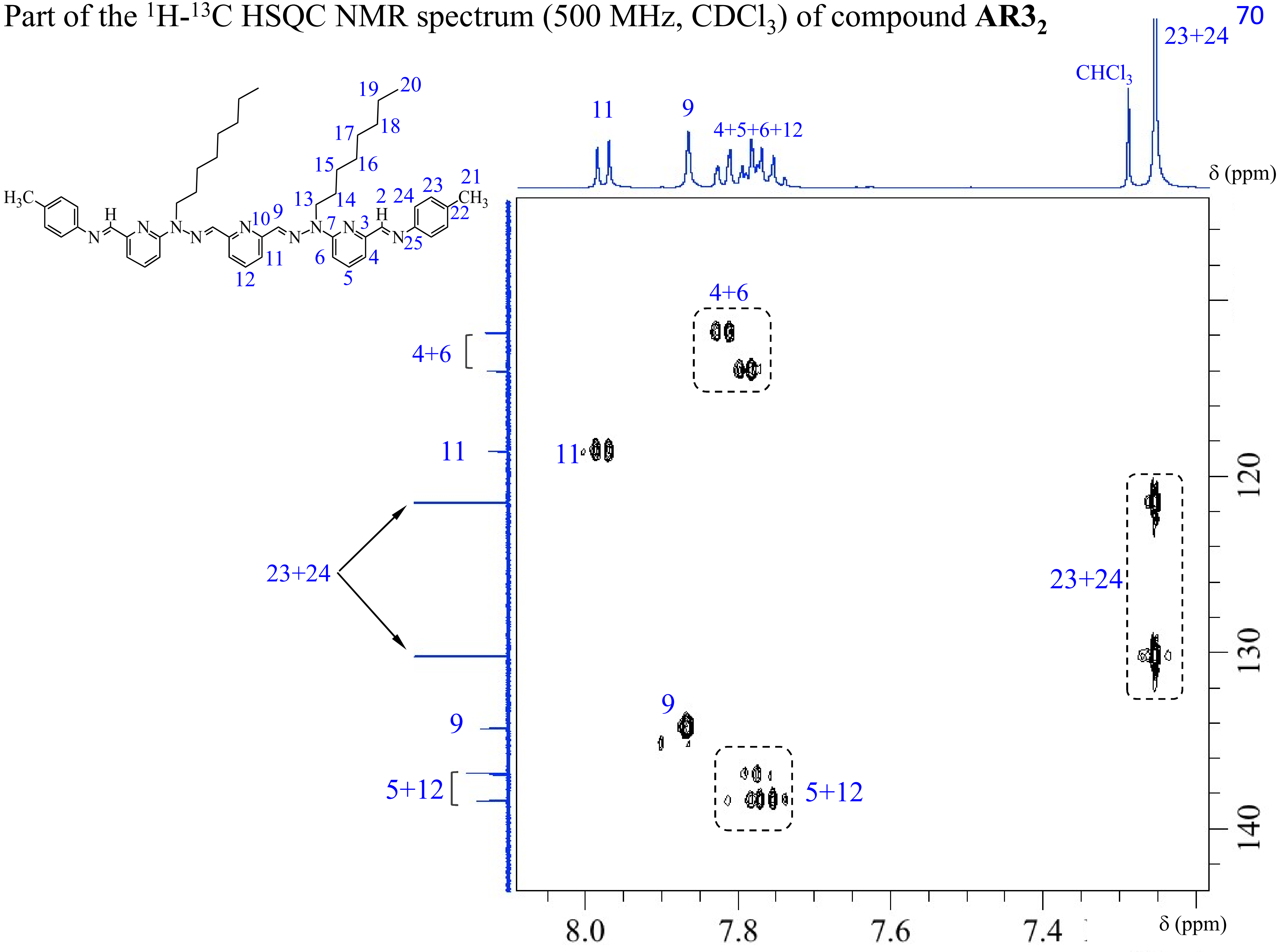

Part of the ^{1}H-^{13}C HSQC NMR spectrum (500 MHz, $CDCl_3$) of compound **AR3$_2$**

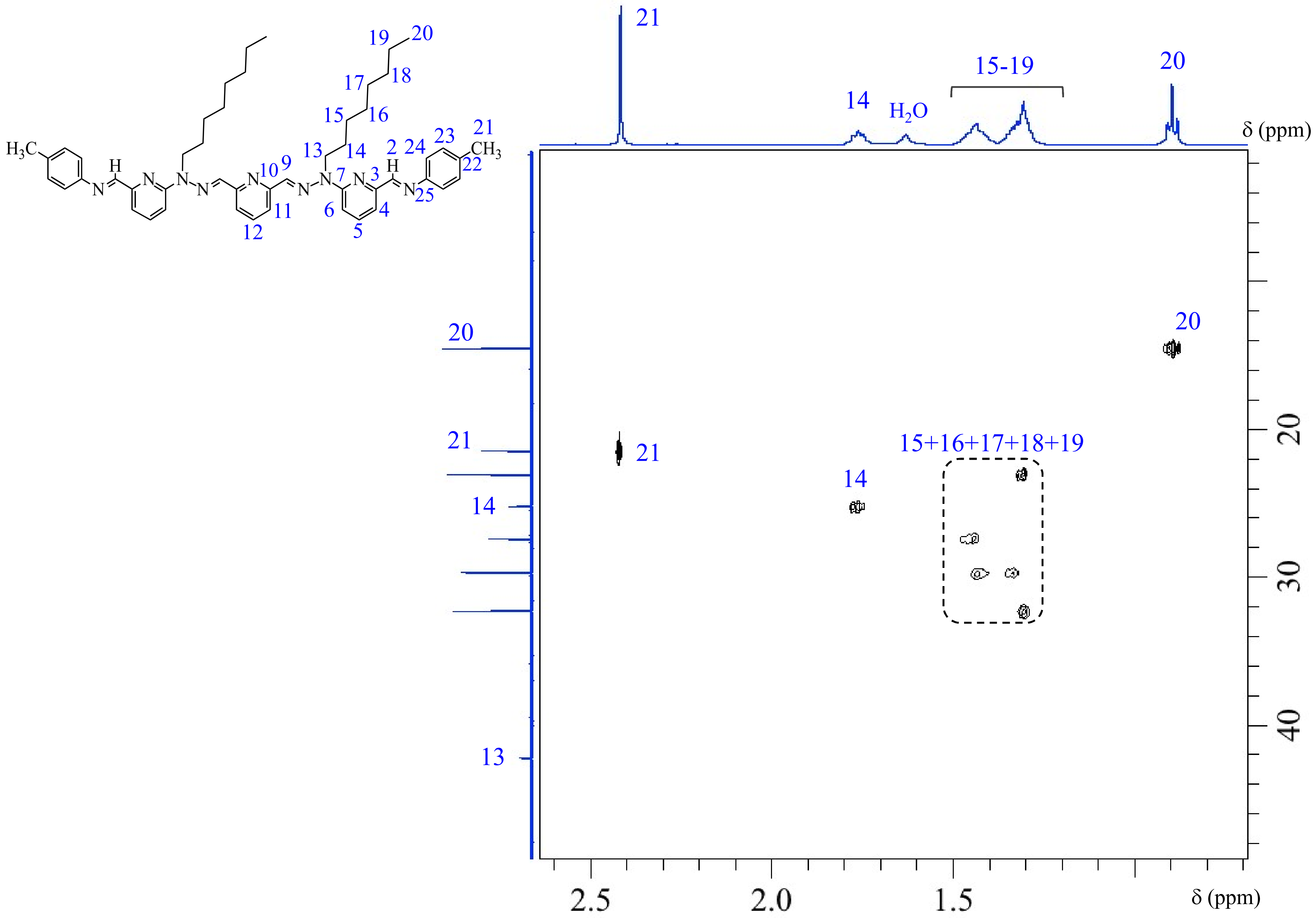

Part of the ^{1}H-^{13}C HMBC NMR spectrum (500 MHz, $CDCl_3$) of compound **AR3$_2$**

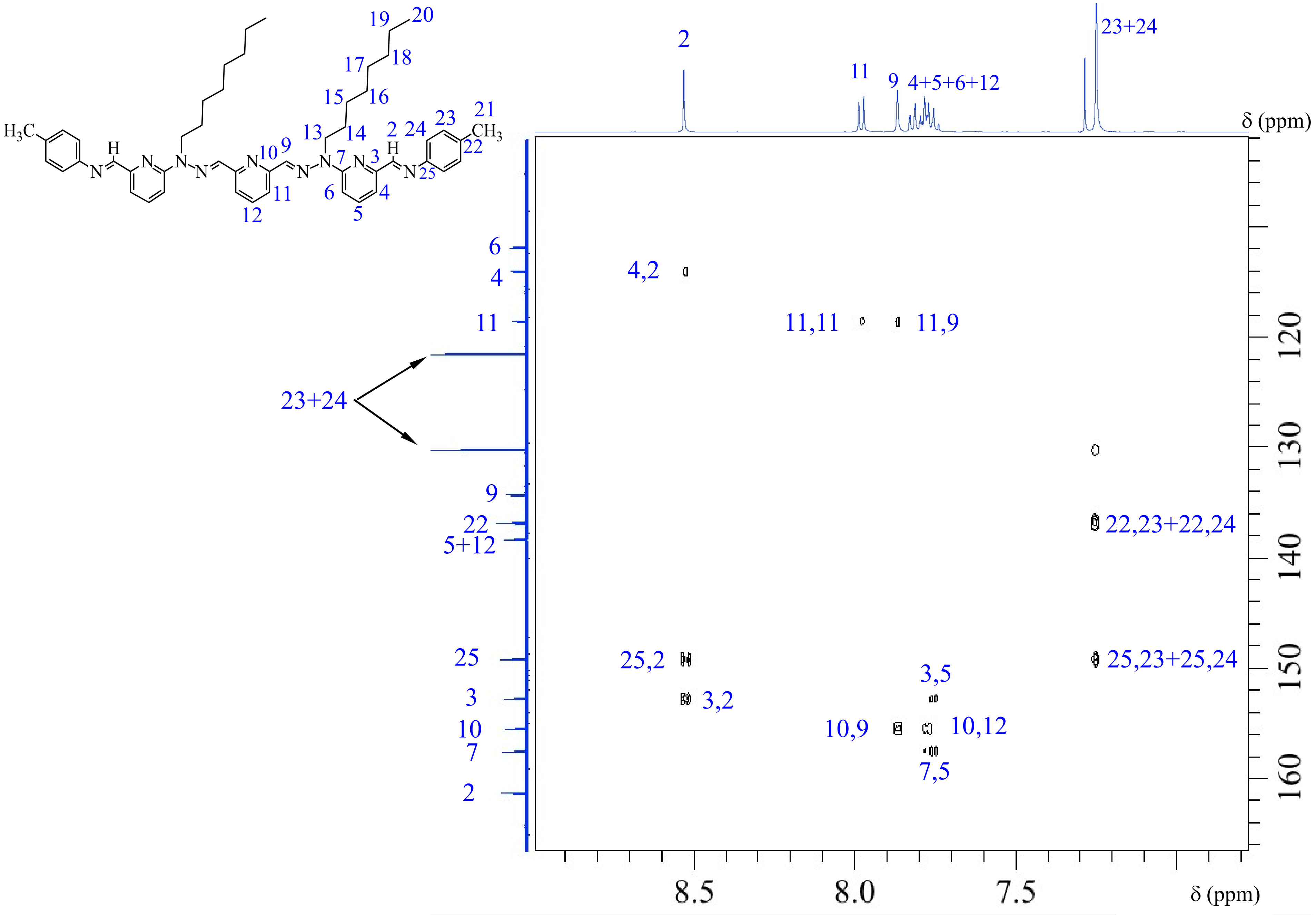

NMR spectra of compound **AR4$_2$**

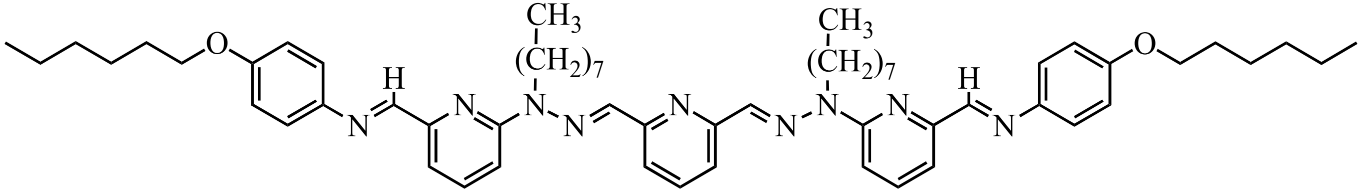

^{1}H NMR spectrum (400 MHz, $CDCl_3$, δ_{ref} = 7.26 ppm) of compound **AR4$_2$**

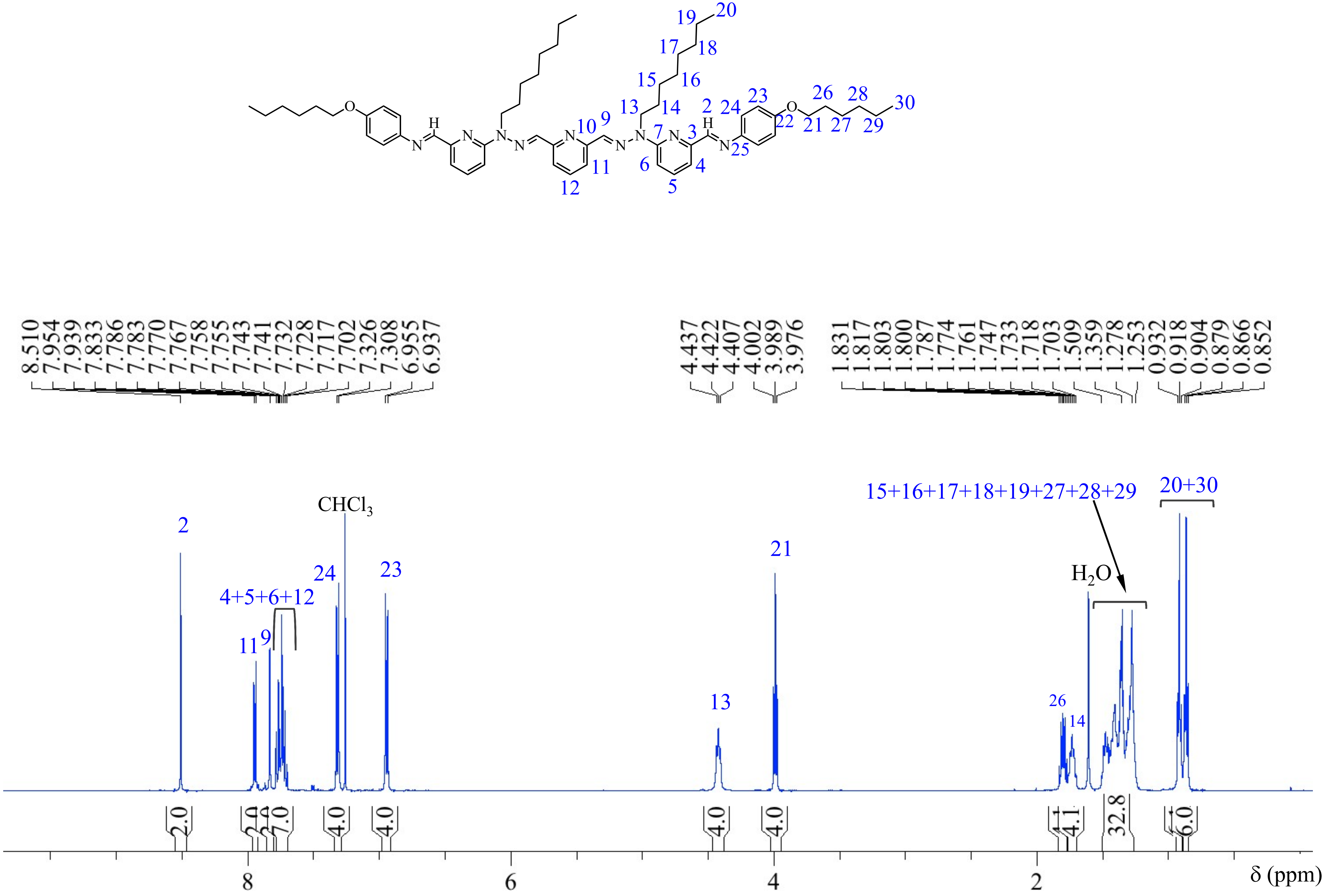

Parts of the ^{1}H NMR spectrum (400 MHz, $CDCl_3$, δ_{ref} = 7.26 ppm) of compound **AR4$_2$**

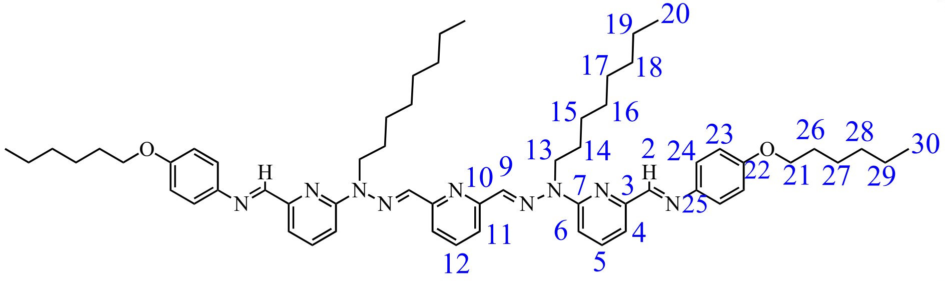

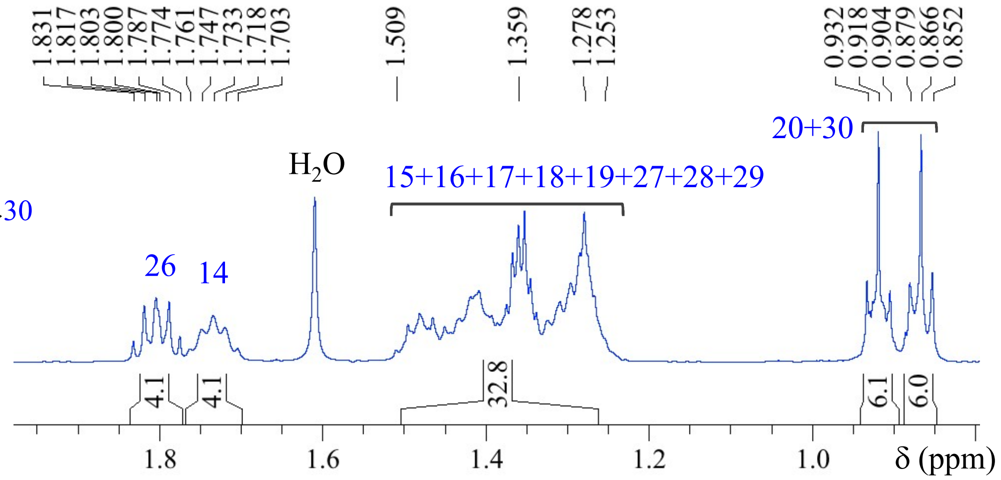

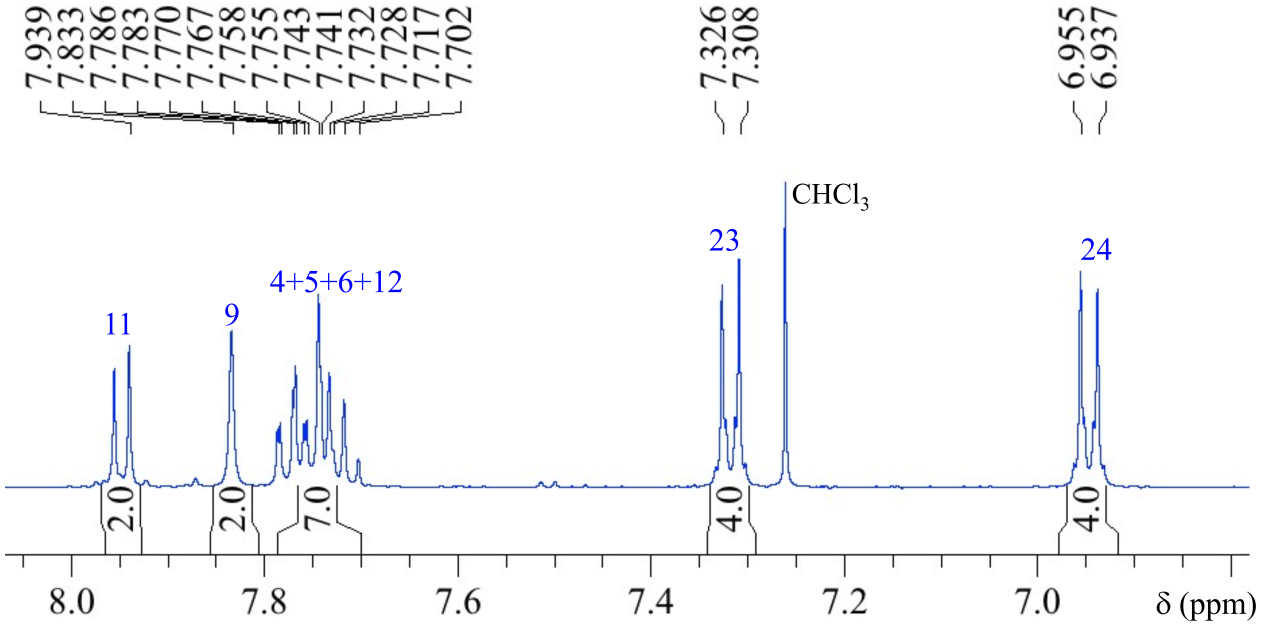

^{13}C NMR spectrum (125 MHz, $CDCl_3$, δ_{ref} = 77.4 ppm) of compound **AR4$_2$**

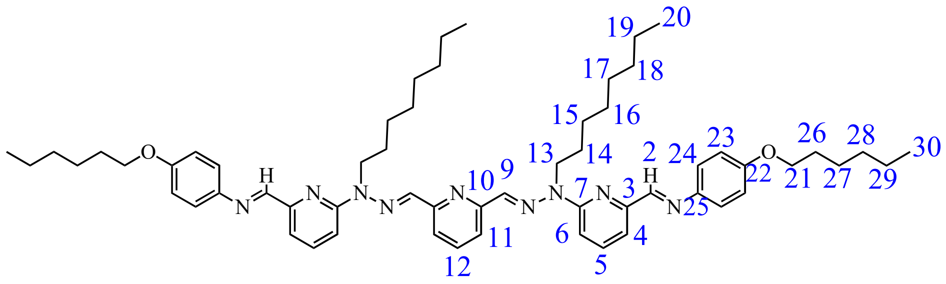

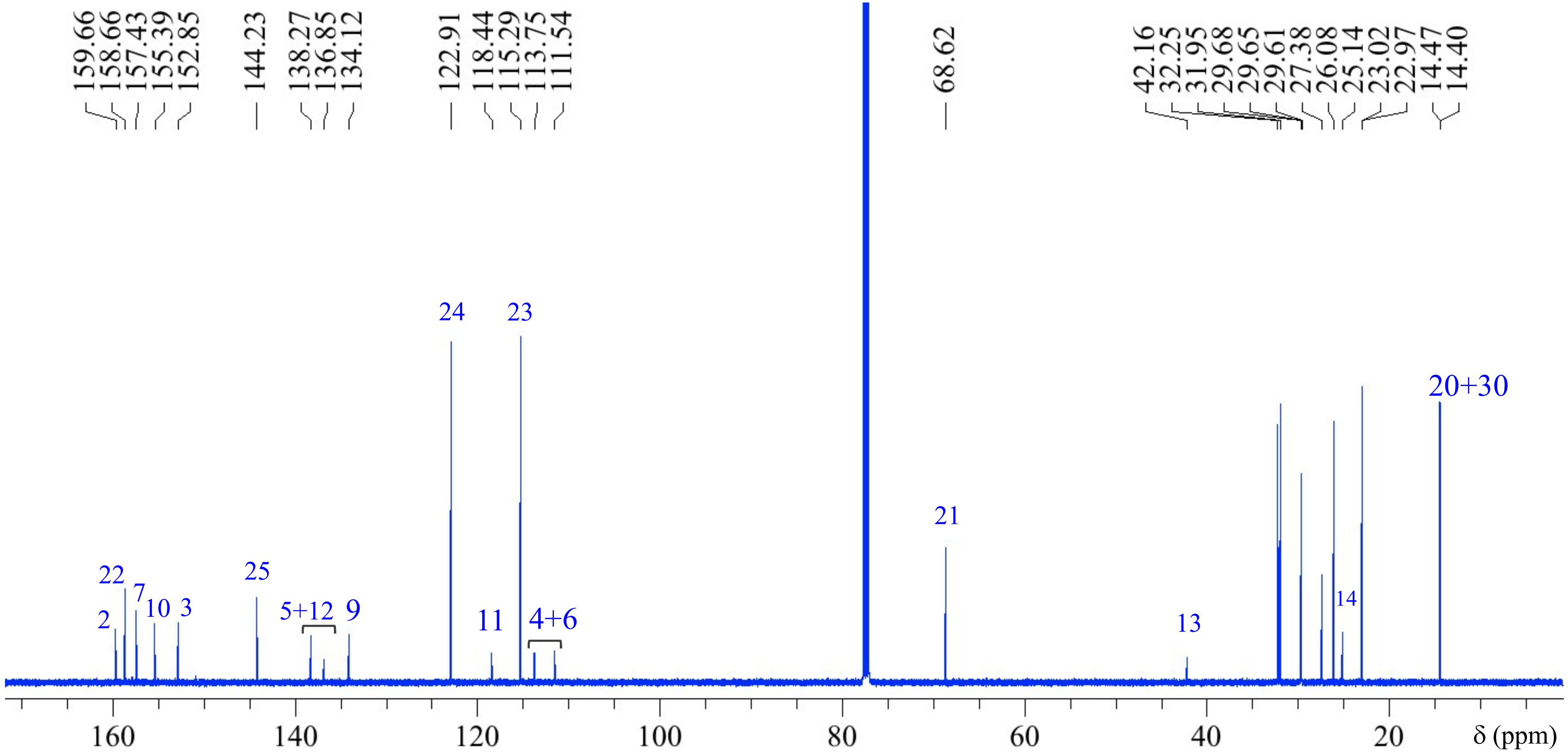

Part of the ^{13}C NMR spectrum (125 MHz, $CDCl_3$, δ_{ref} = 77.4 ppm) of compound **$AR4_2$**

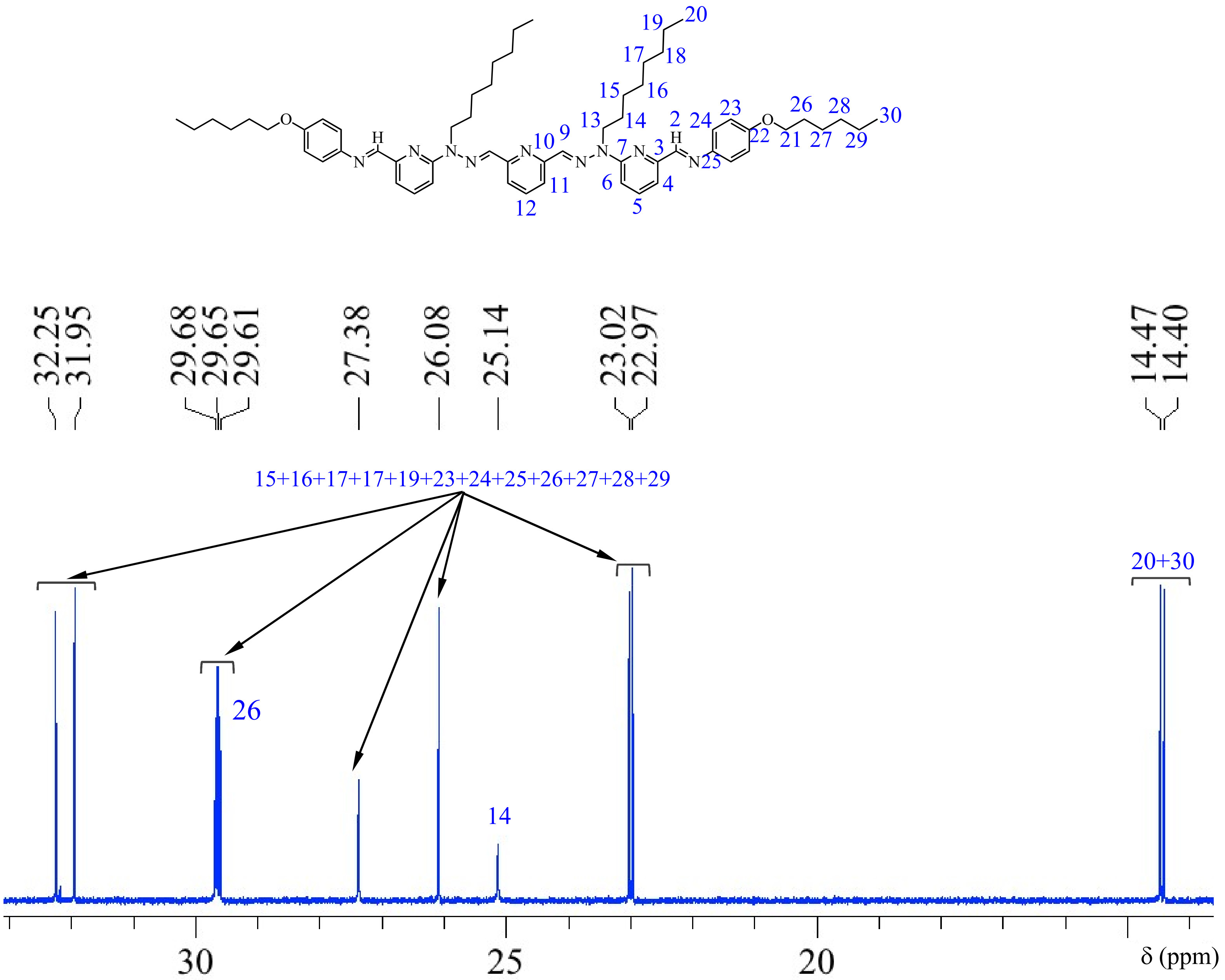

^1H-^1H COSY NMR spectrum (500 MHz, $CDCl_3$) of compound **AR4$_2$**

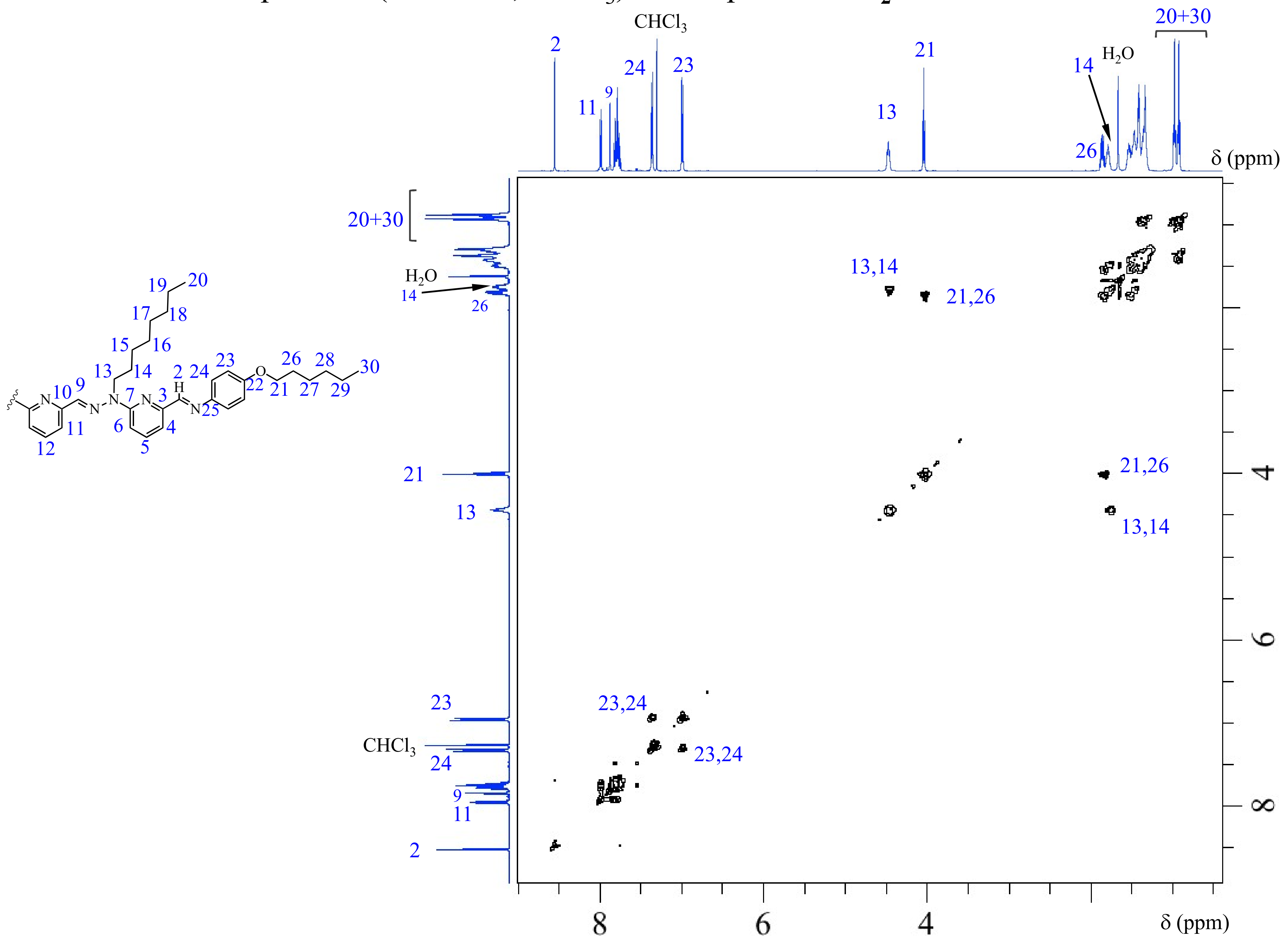

Part of the ^{1}H-^{1}H COSY NMR spectrum (500 MHz, $CDCl_3$) of compound **AR4$_2$**

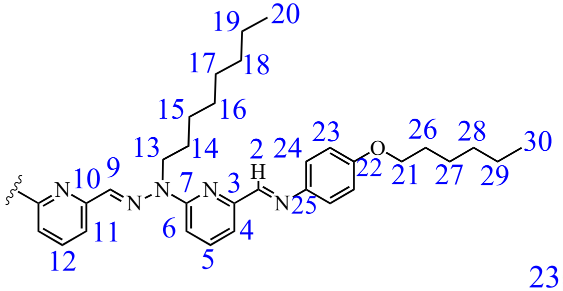

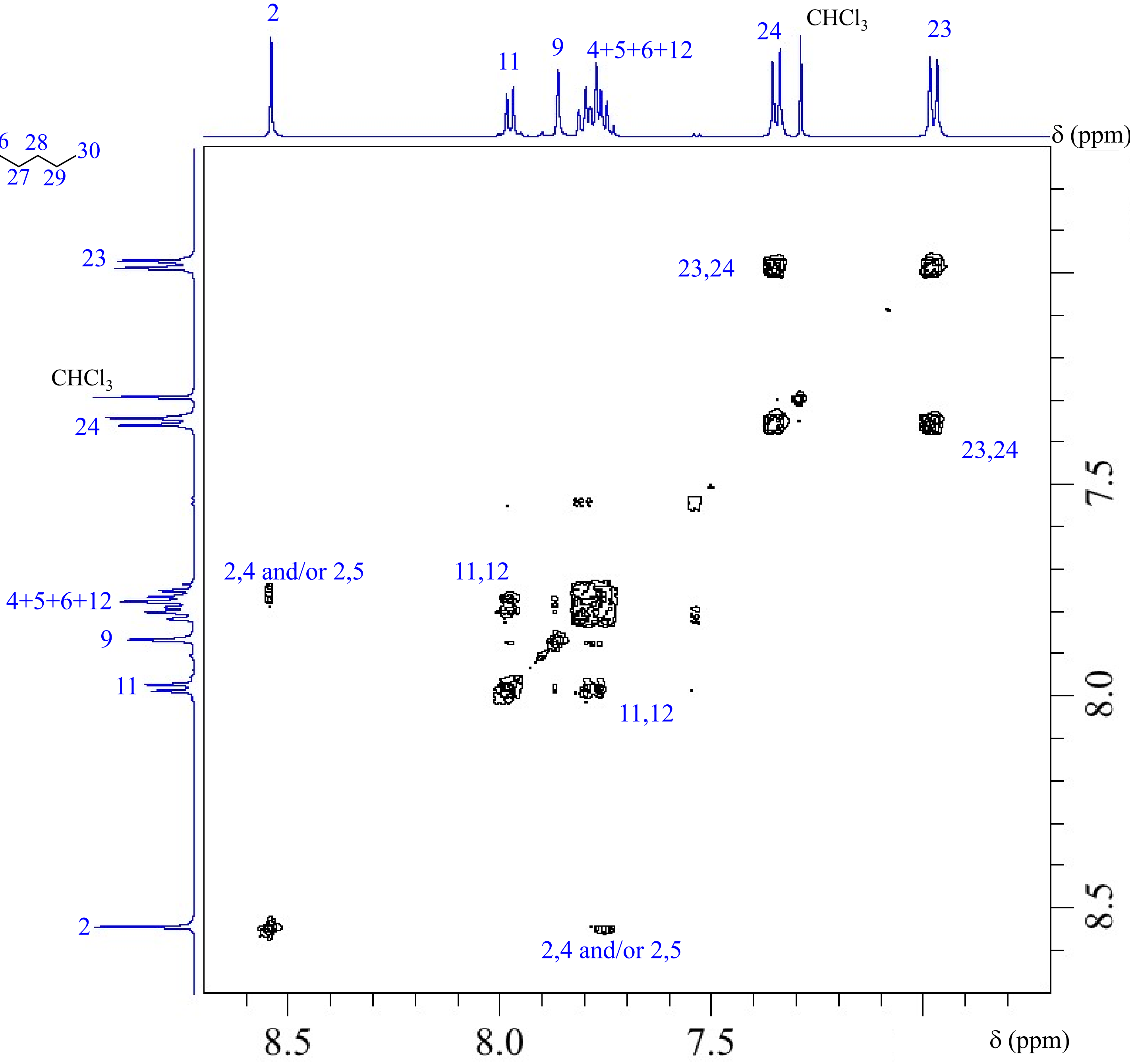

Part of the ^1H-^1H COSY NMR spectrum (500 MHz, $CDCl_3$) of compound **$AR4_2$**

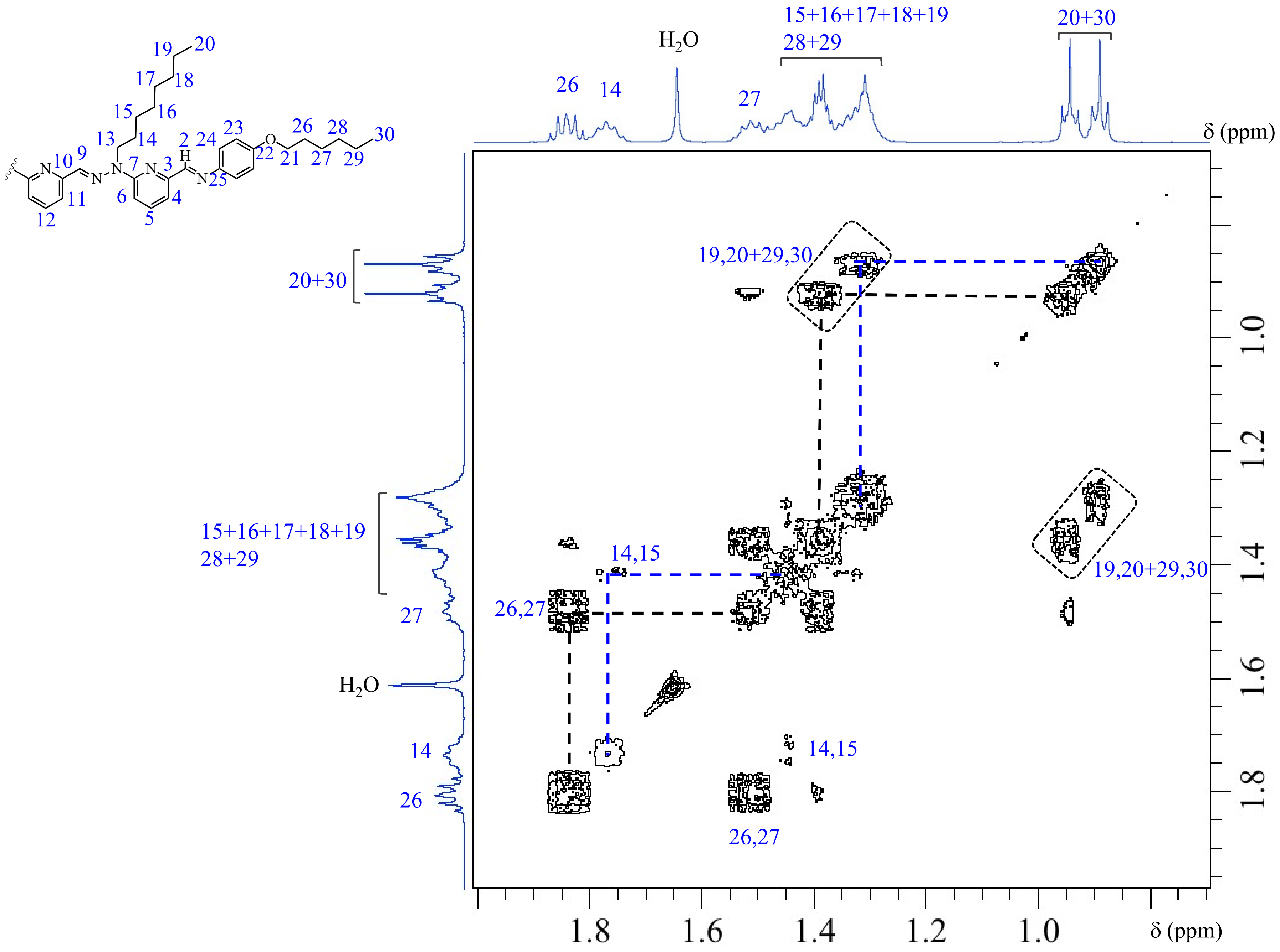

^{1}H-^{1}H ROESY NMR spectrum (500 MHz, $CDCl_3$) of compound **AR4$_2$**

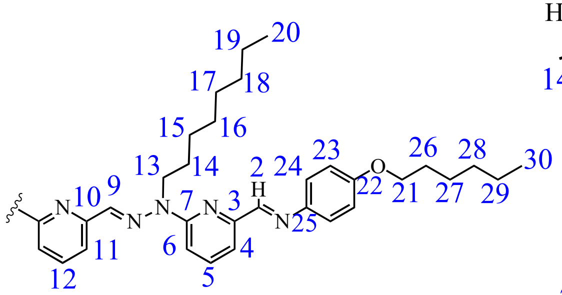

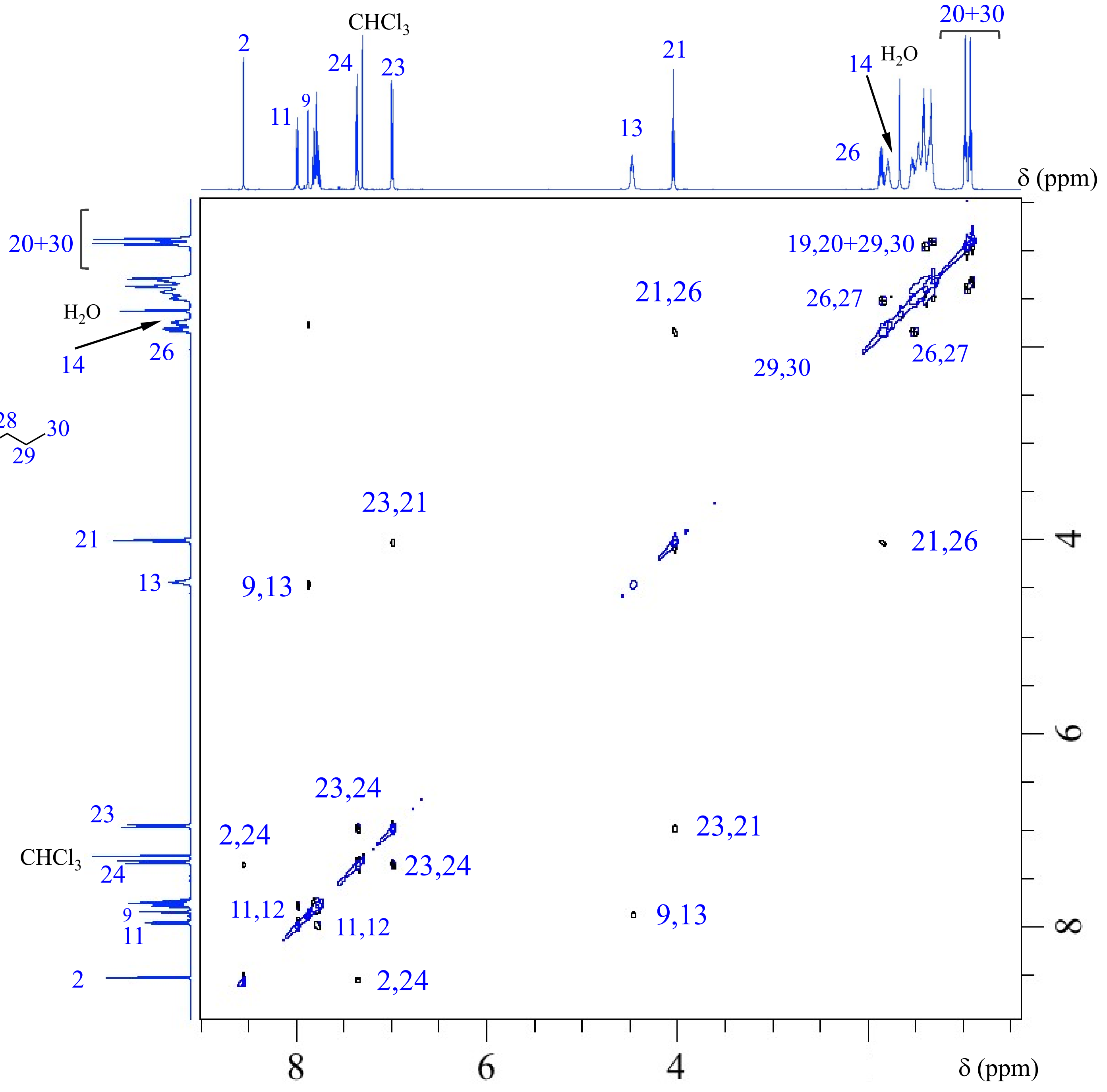

^{1}H-^{13}C HSQC NMR spectrum (500 MHz, $CDCl_3$) of compound **AR4$_2$**

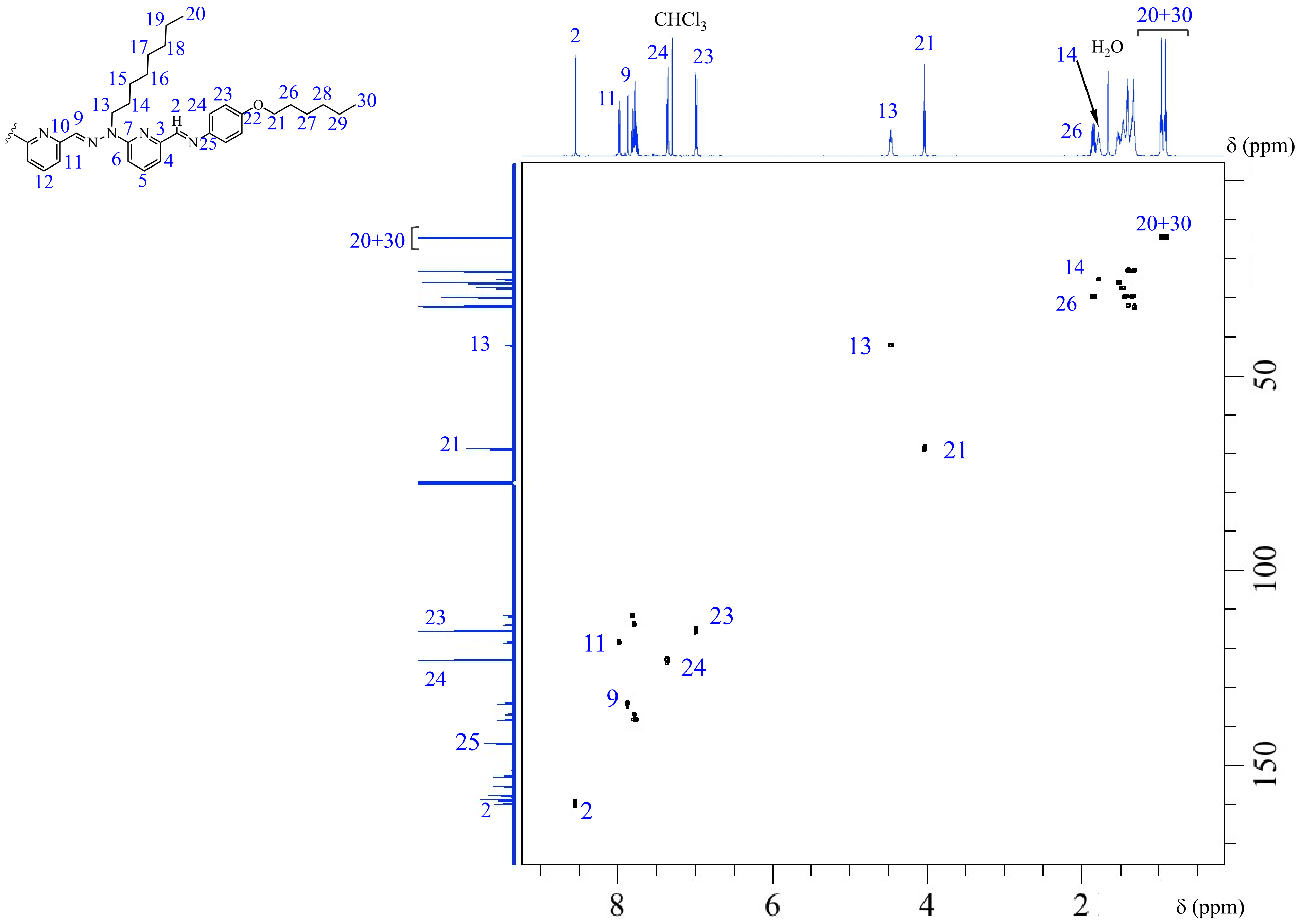

Part of the ^{1}H-^{13}C HSQC NMR spectrum (500 MHz, $CDCl_3$) of compound **$AR4_2$**

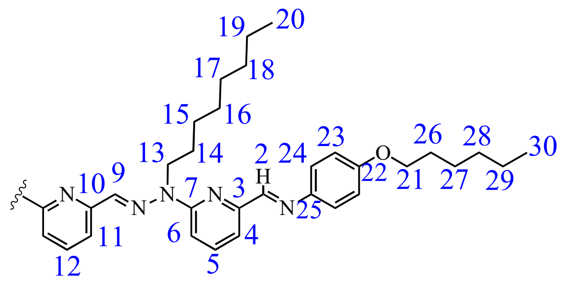

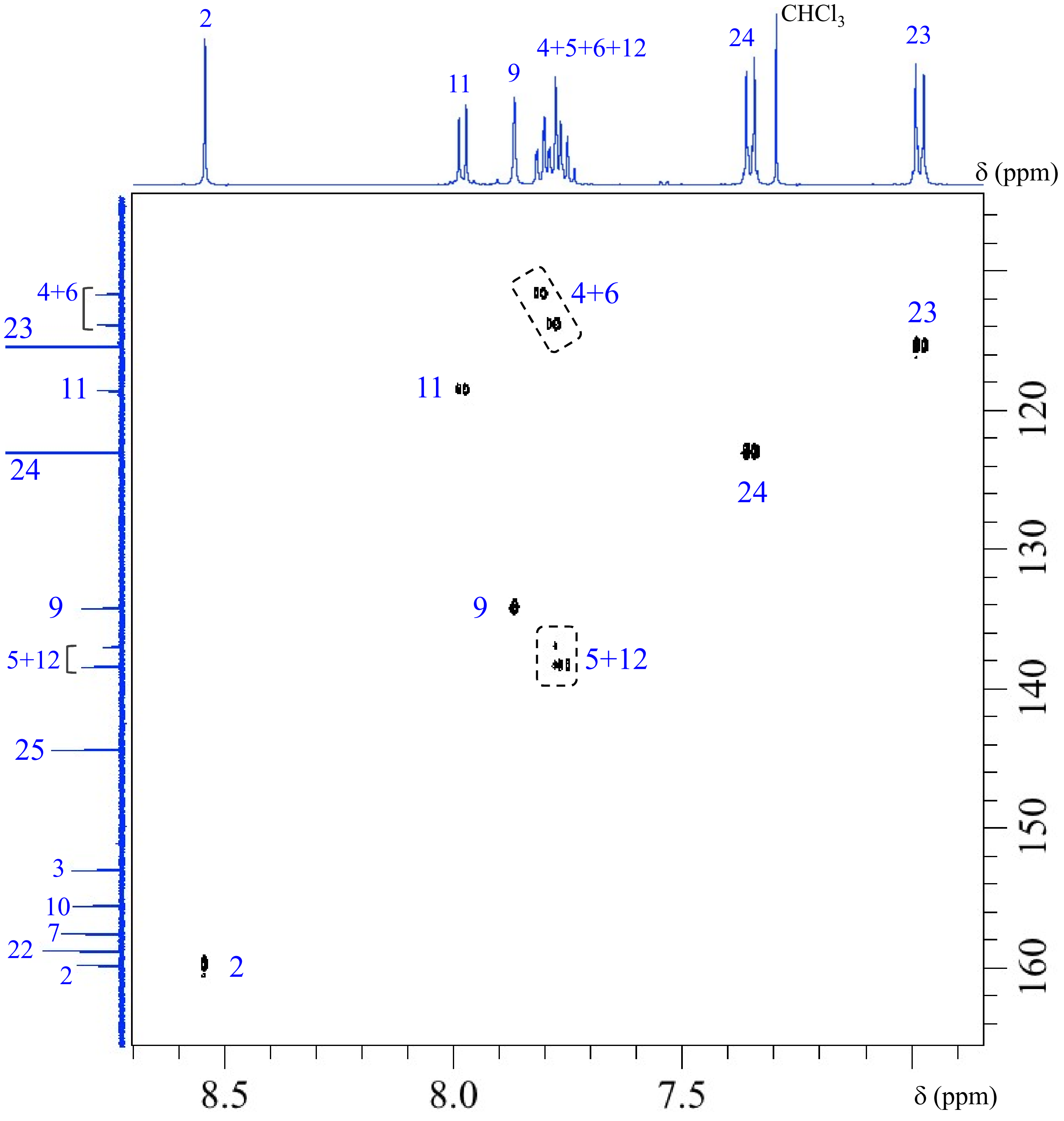

Part of the ^{1}H-^{13}C HSQC NMR spectrum (500 MHz, $CDCl_3$) of compound **AR4$_2$**

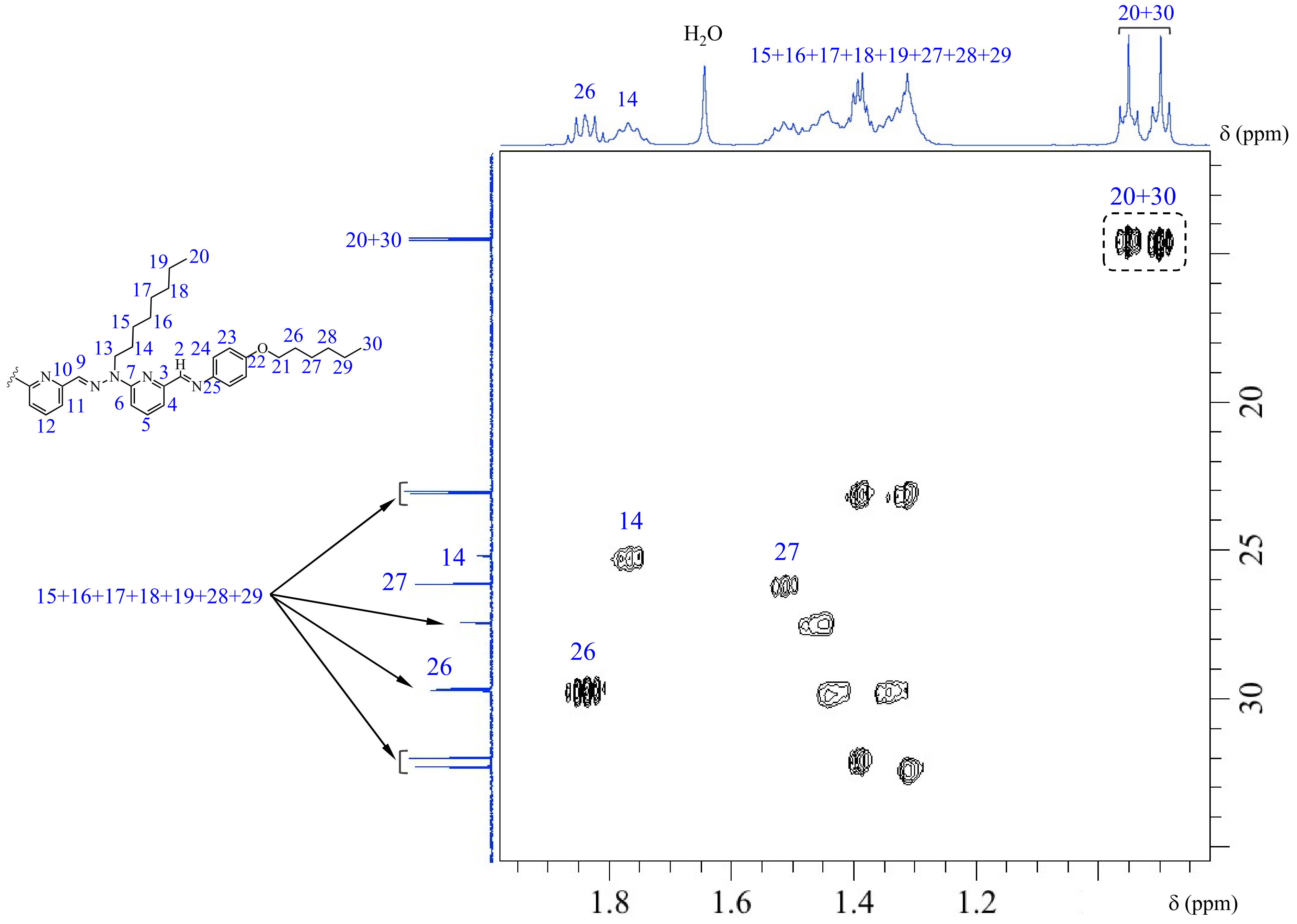

^{1}H-^{13}C HMBC NMR spectrum (500 MHz, $CDCl_3$) of compound **AR4$_2$**

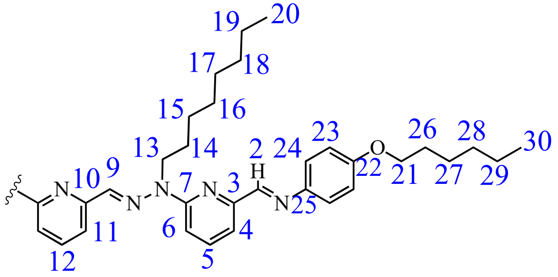

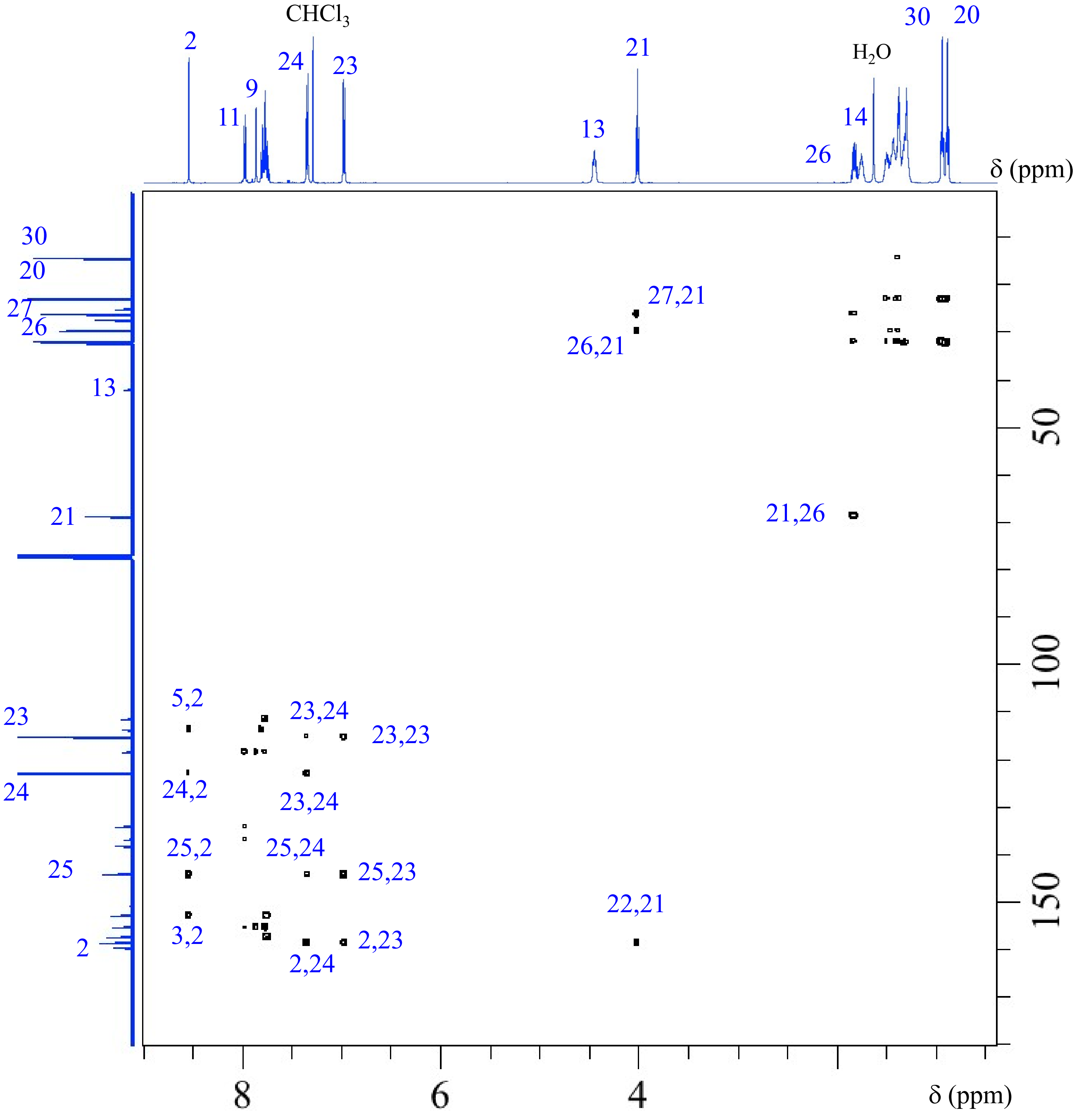

Part of the ^{1}H-^{13}C HMBC NMR spectrum (500 MHz, $CDCl_3$) of compound **$AR4_2$**

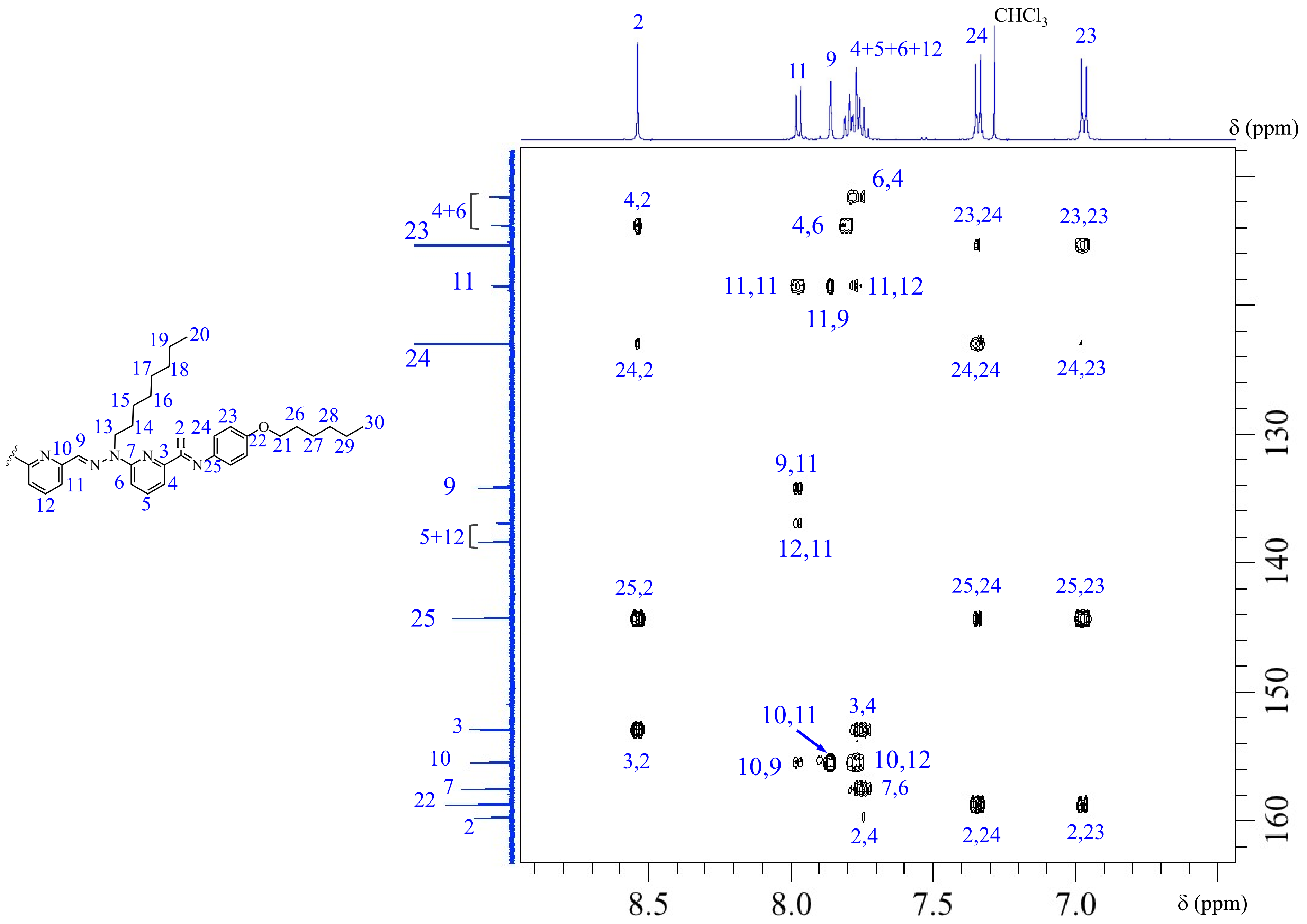

Part of the $^{1}H-^{13}C$ HMBC NMR spectrum (500 MHz, $CDCl_3$) of compound **AR4$_2$**

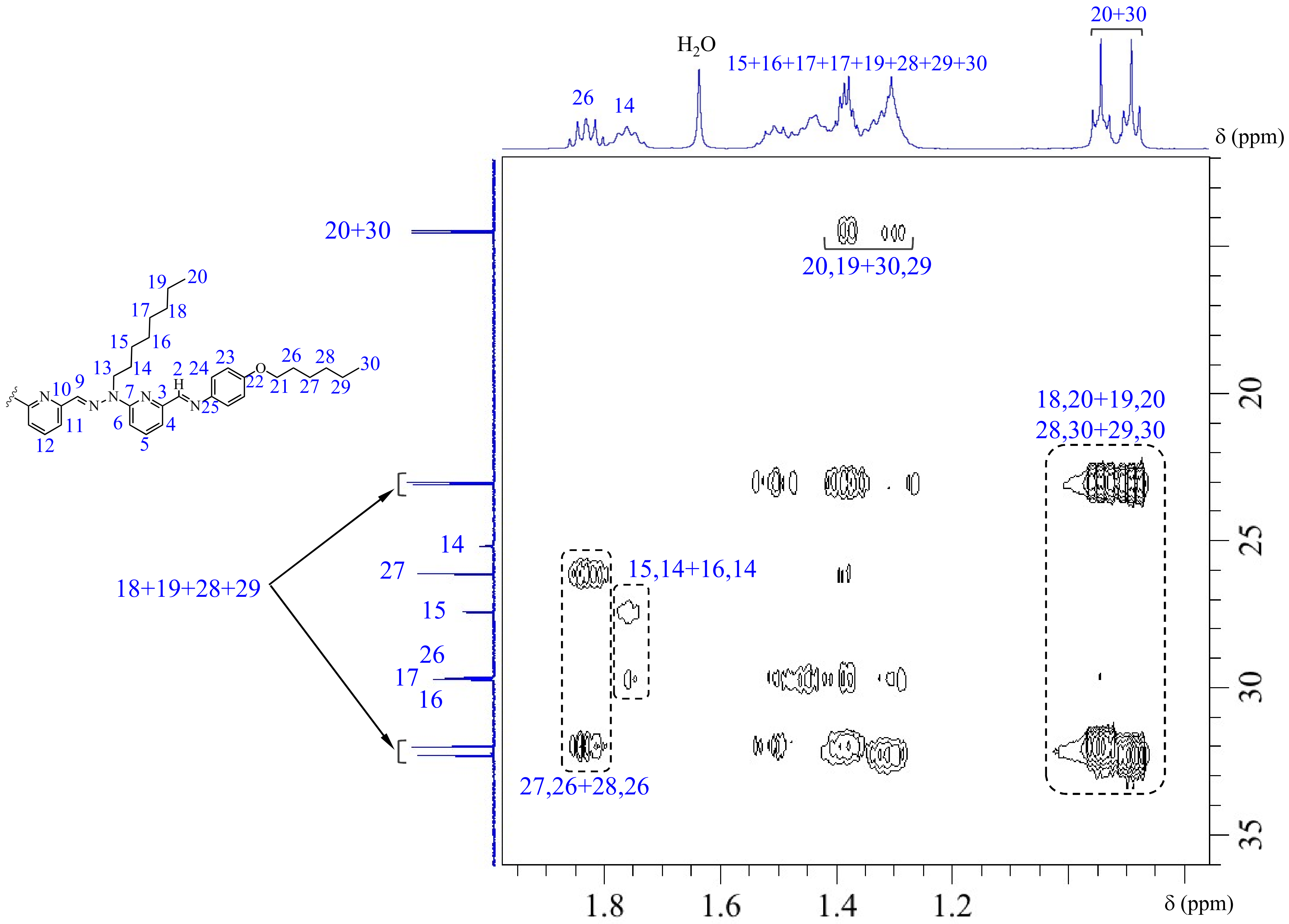

NMR spectra of compound **AL3$_2$**

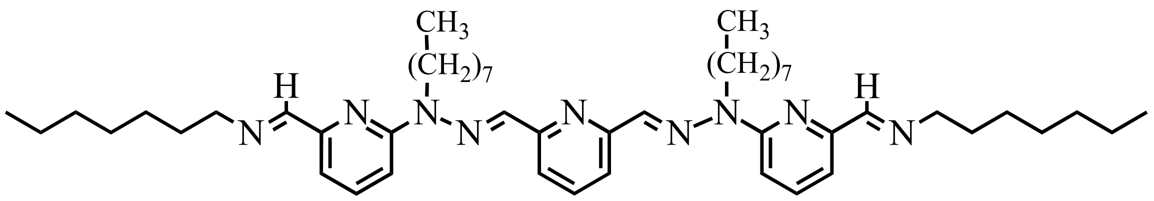

^{1}H NMR spectrum (500 MHz, $CDCl_3$, δ_{ref} = 7.26 ppm) of compound **AL3$_2$**

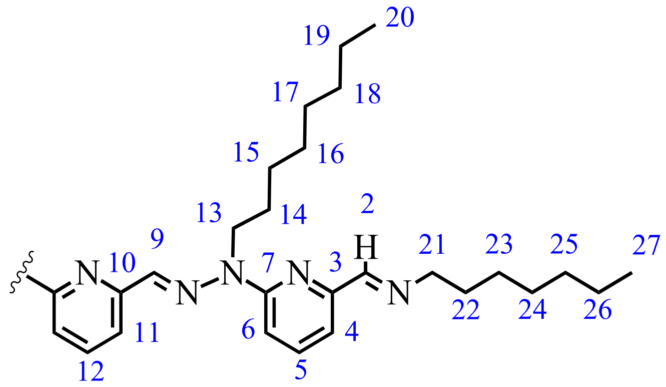

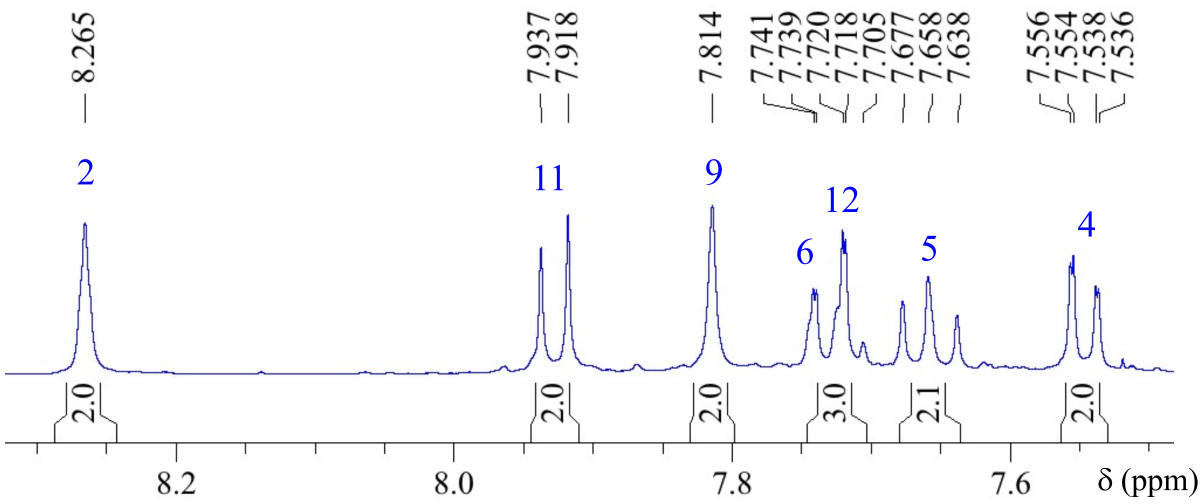

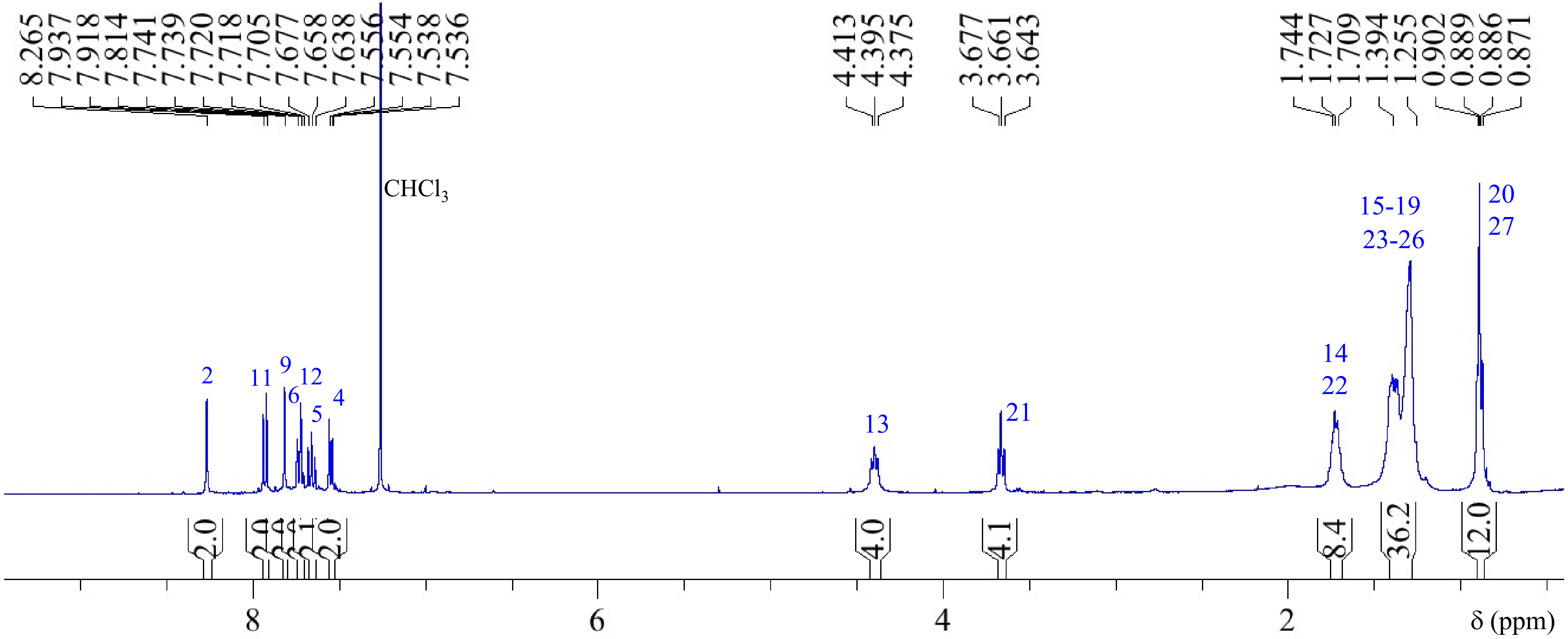

^{13}C NMR spectrum (125 MHz, $CDCl_3$, δ_{ref} = 77.4 ppm) of compound **$AL3_2$**

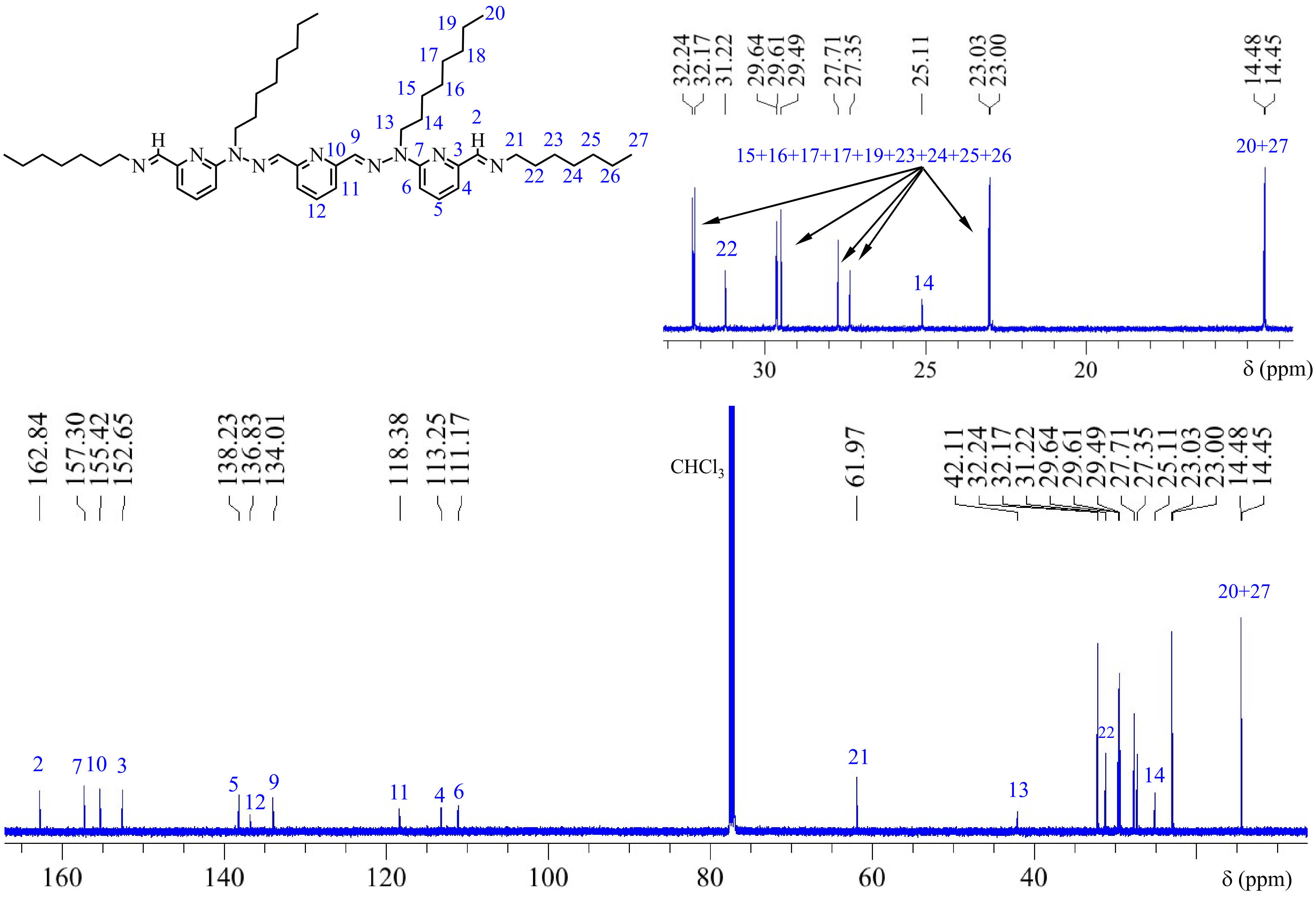

^{1}H-^{1}H COSY NMR spectrum (500 MHz, $CDCl_3$) of compound **AL3$_2$**

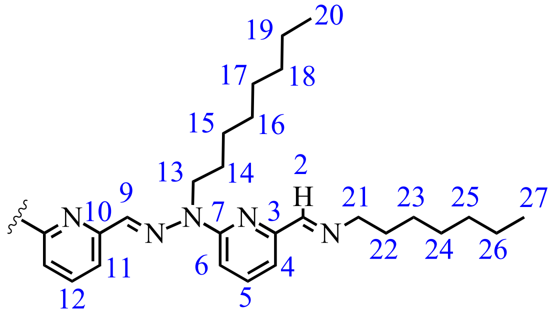

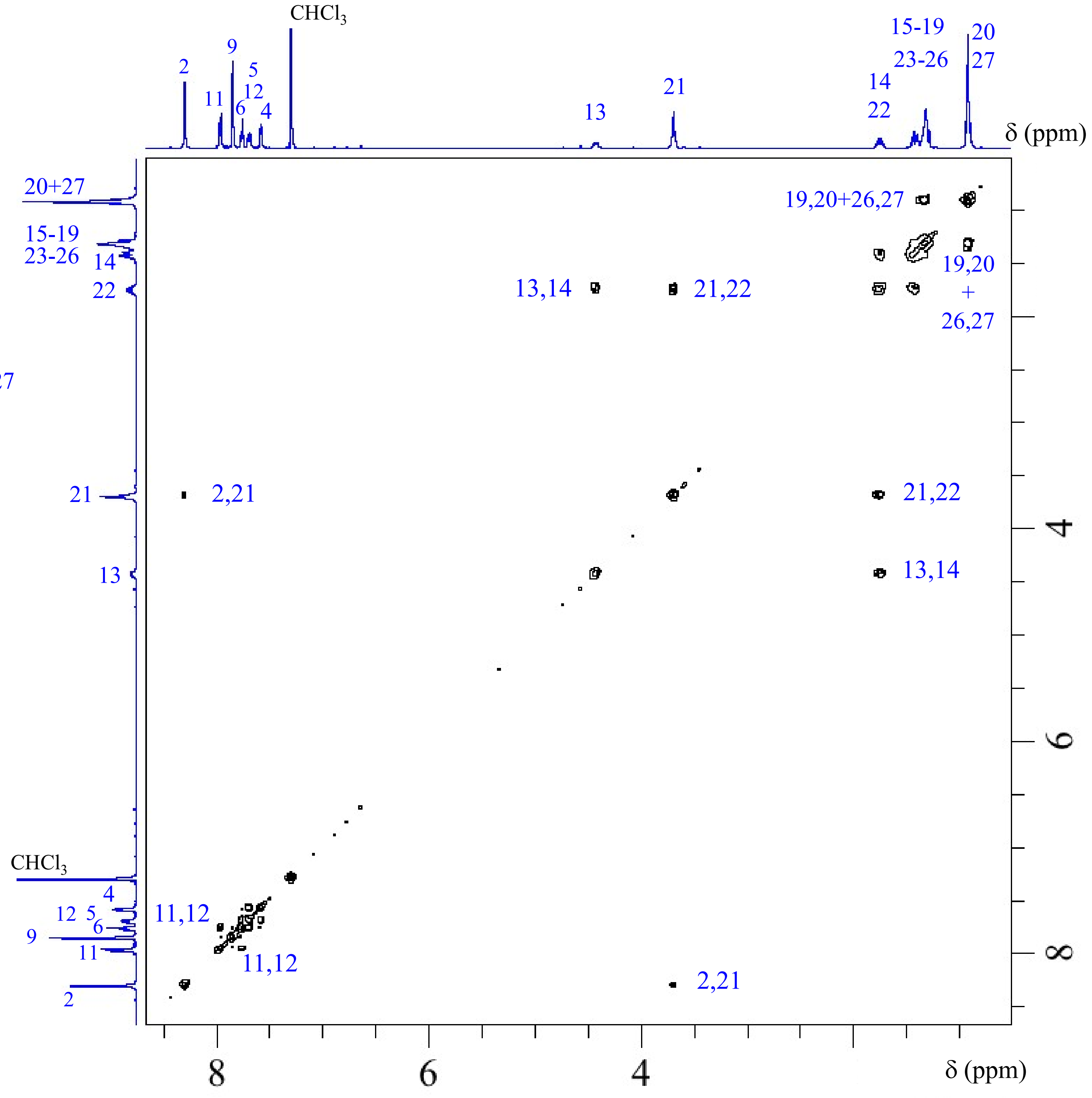

Part of the ^{1}H-^{1}H COSY NMR spectrum (500 MHz, $CDCl_3$) of compound **AL3$_2$**

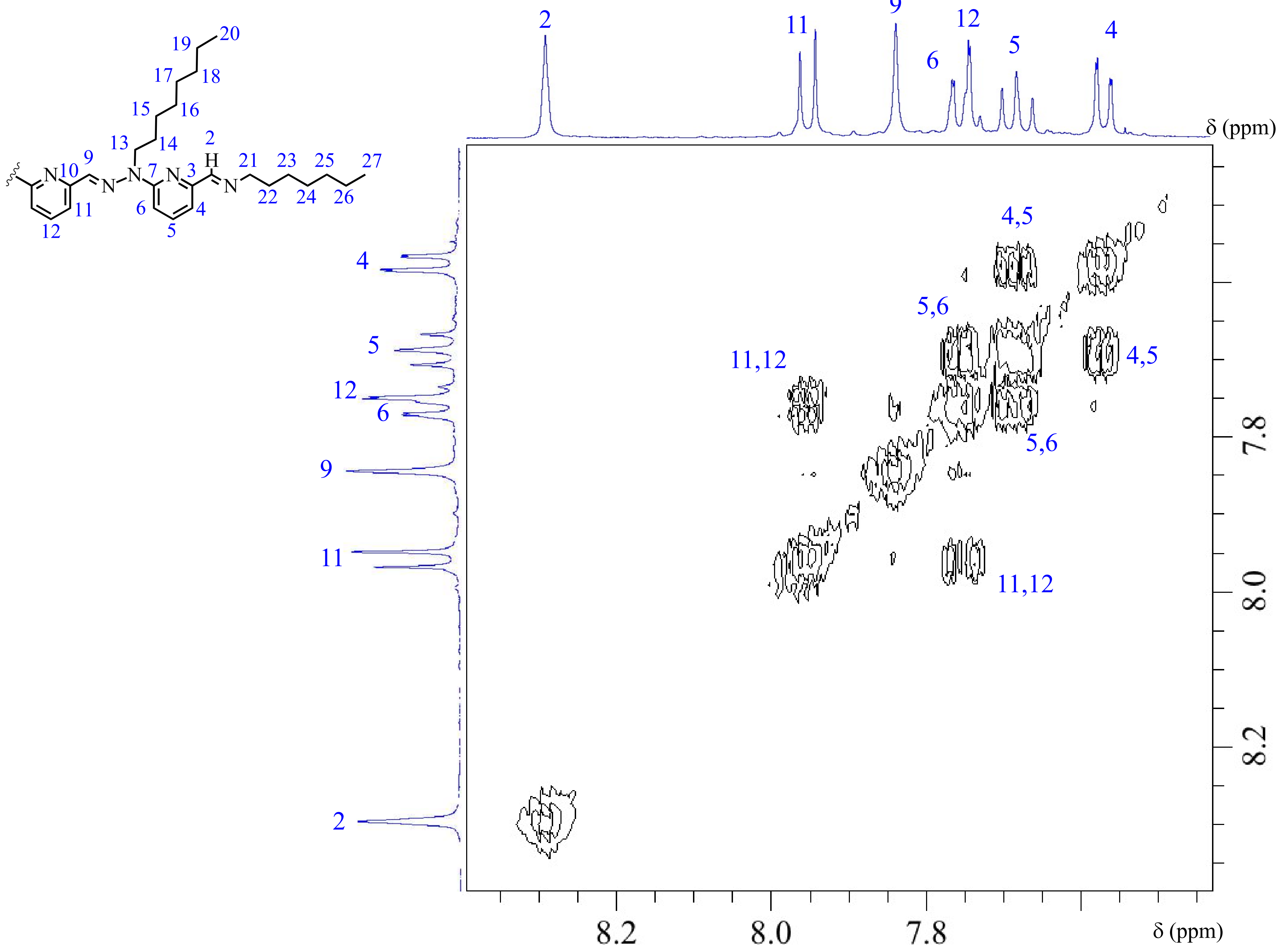

^{1}H-^{1}H ROESY NMR spectrum (500 MHz, $CDCl_3$) of compound **AL3$_2$**

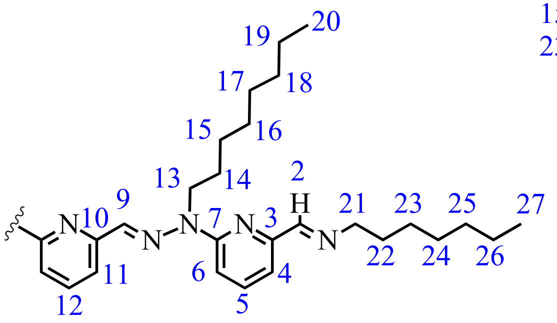

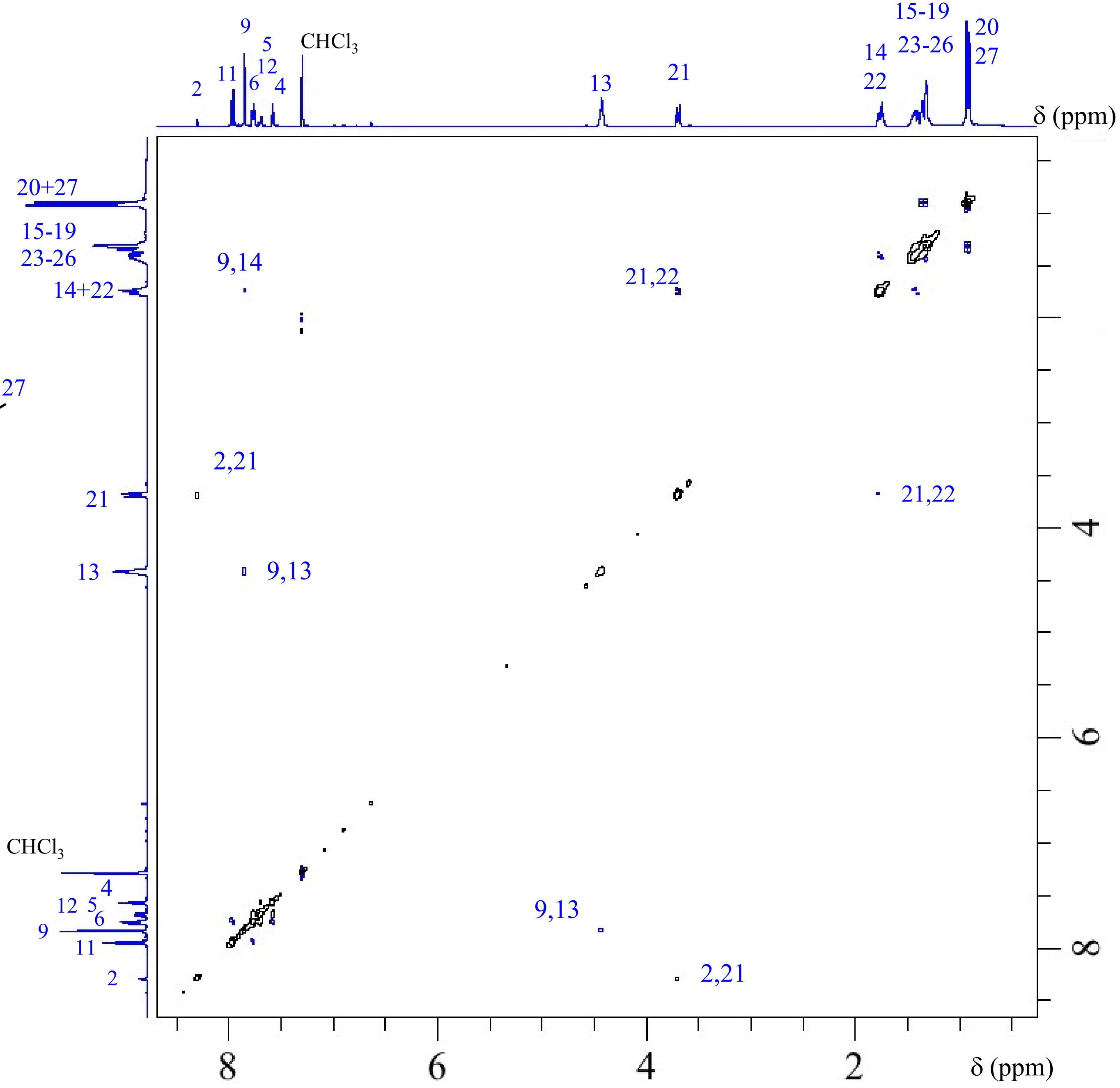

^{1}H-^{13}C HSQC NMR spectrum (500 MHz, $CDCl_3$) of compound **AL3$_2$**

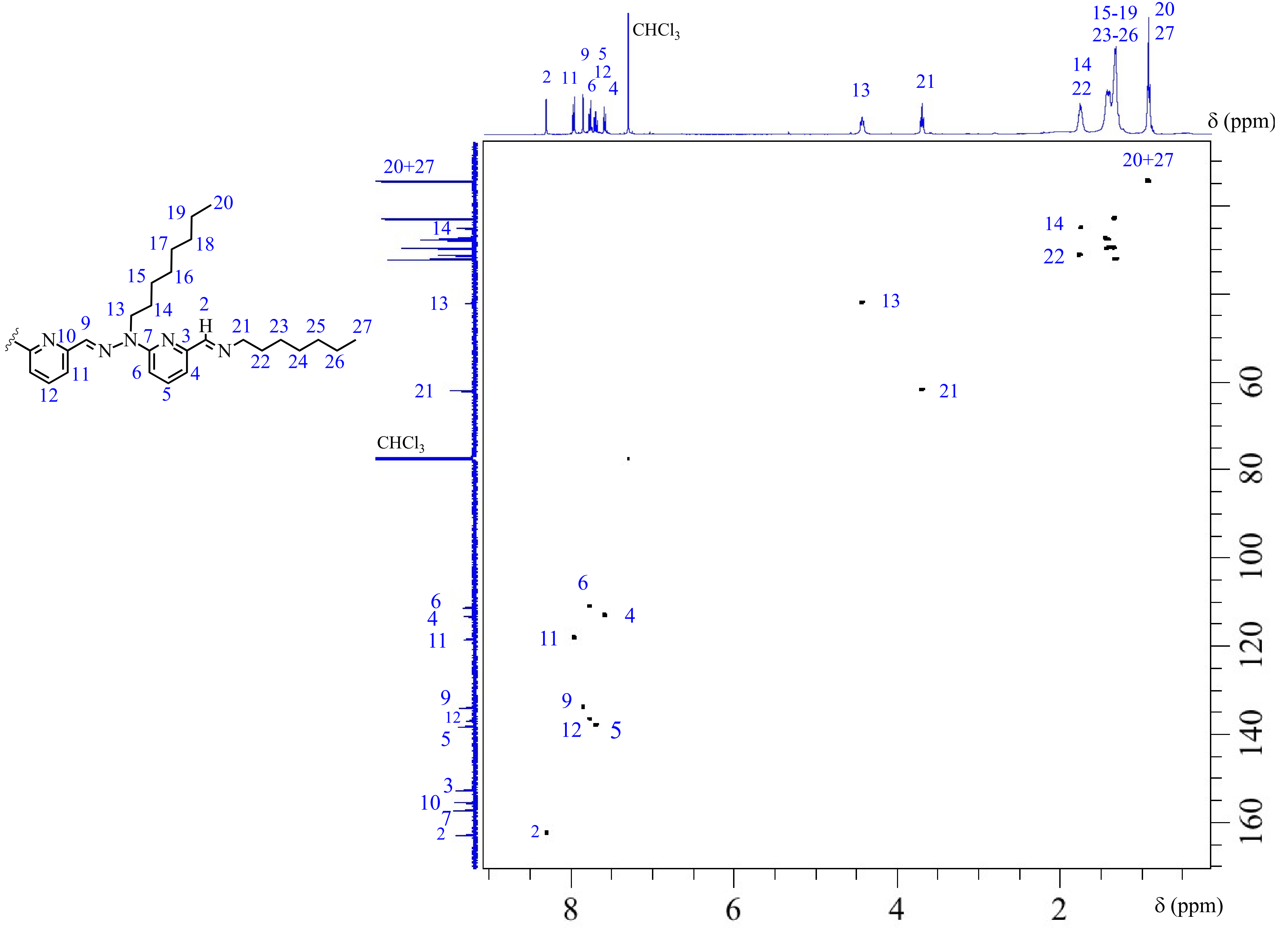

Part of the ^{1}H-^{13}C HSQC NMR spectrum (500 MHz, $CDCl_3$) of compound **AL3$_2$**

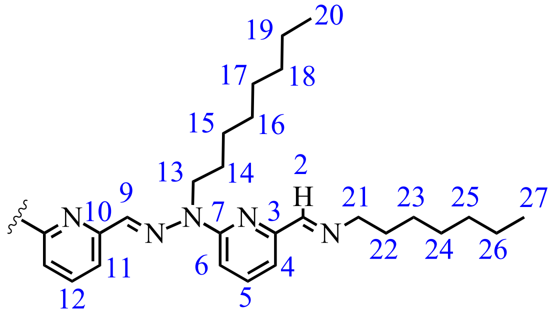

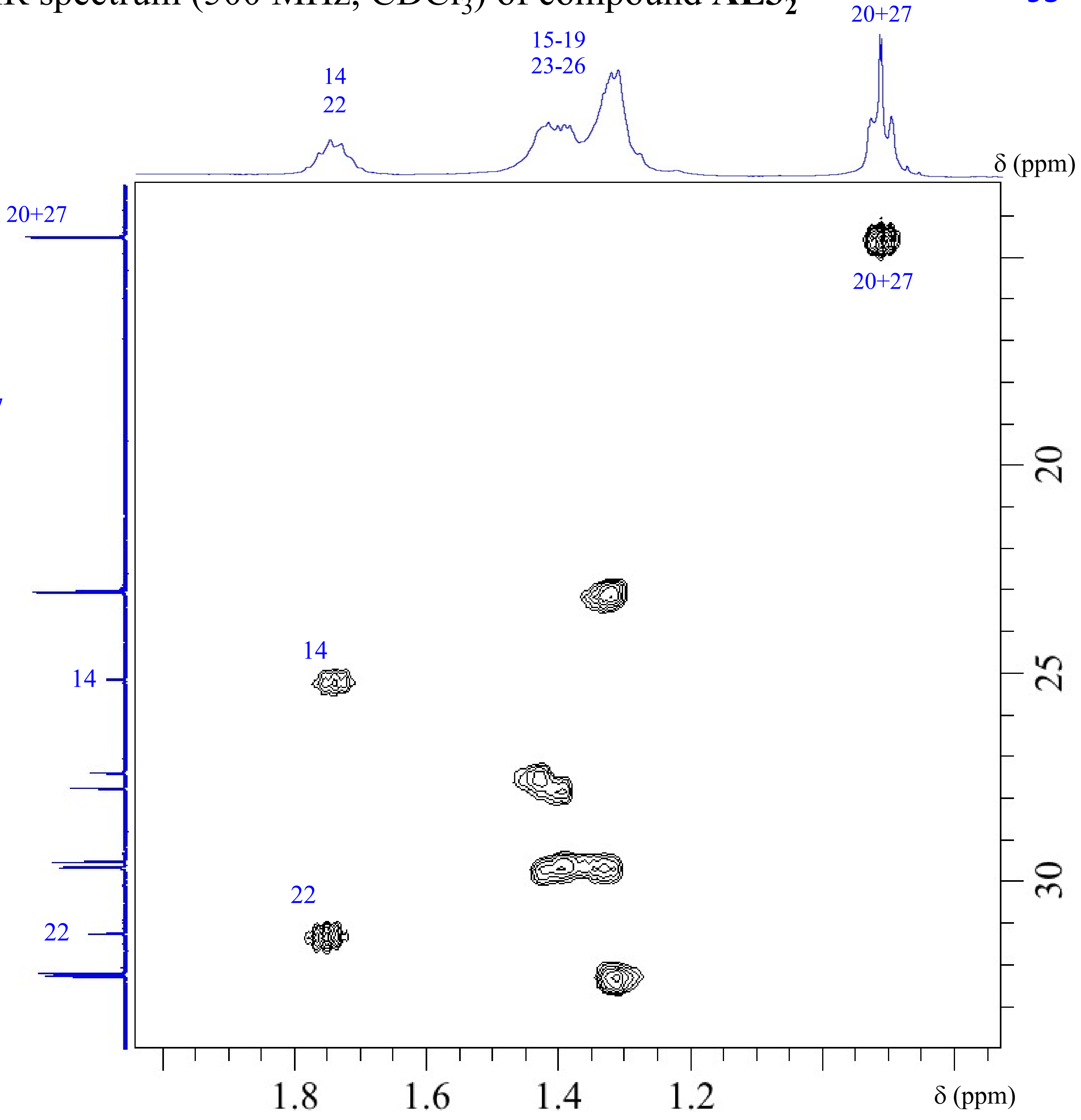

Part of the ^{1}H-^{13}C HSQC NMR spectrum (500 MHz, $CDCl_3$) of compound **AL3$_2$**

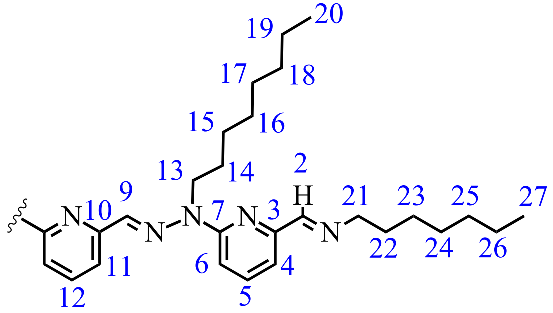

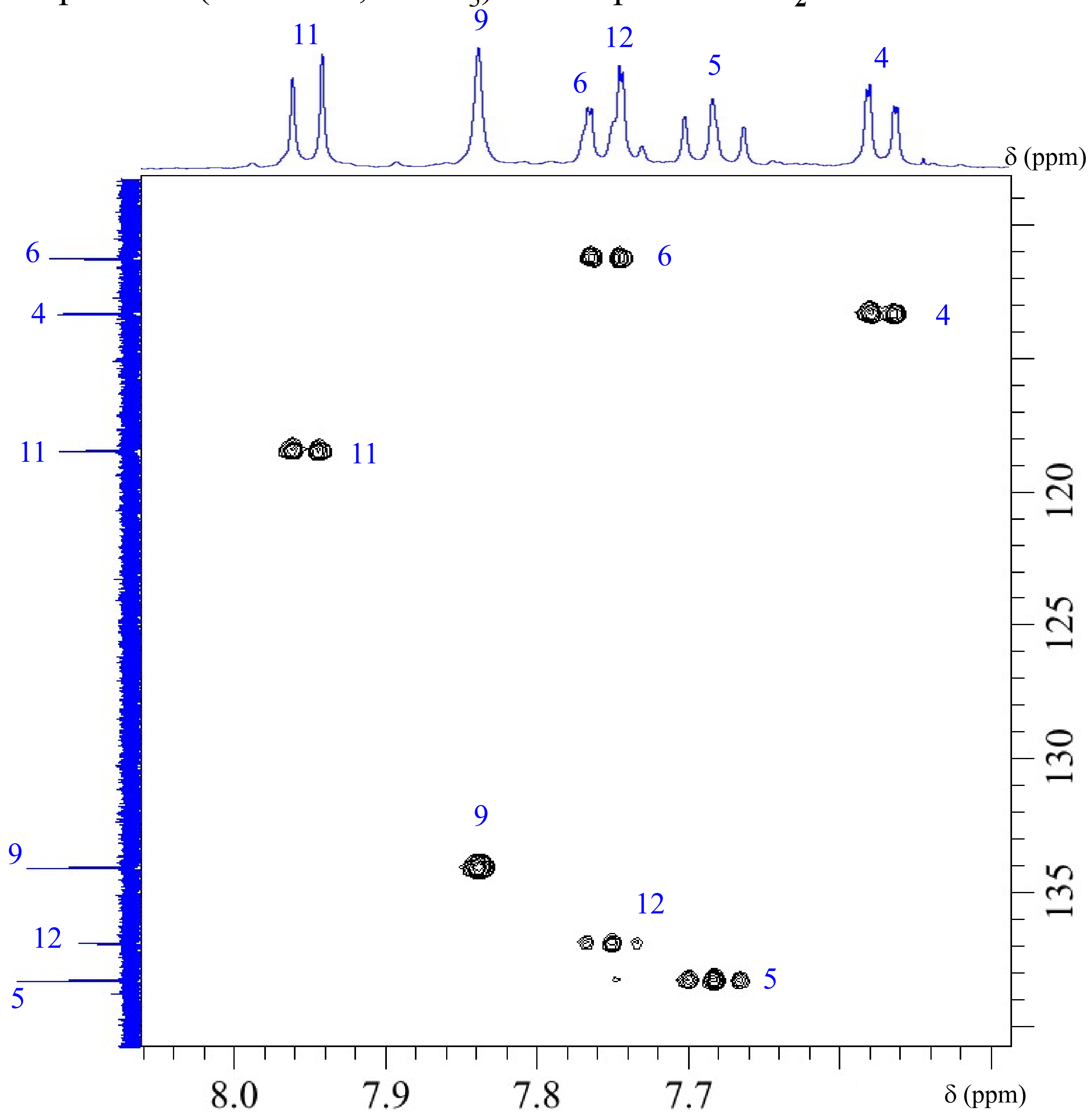

^1H-^{13}C HMBC NMR spectrum (500 MHz, $CDCl_3$) of compound **AL3$_2$**

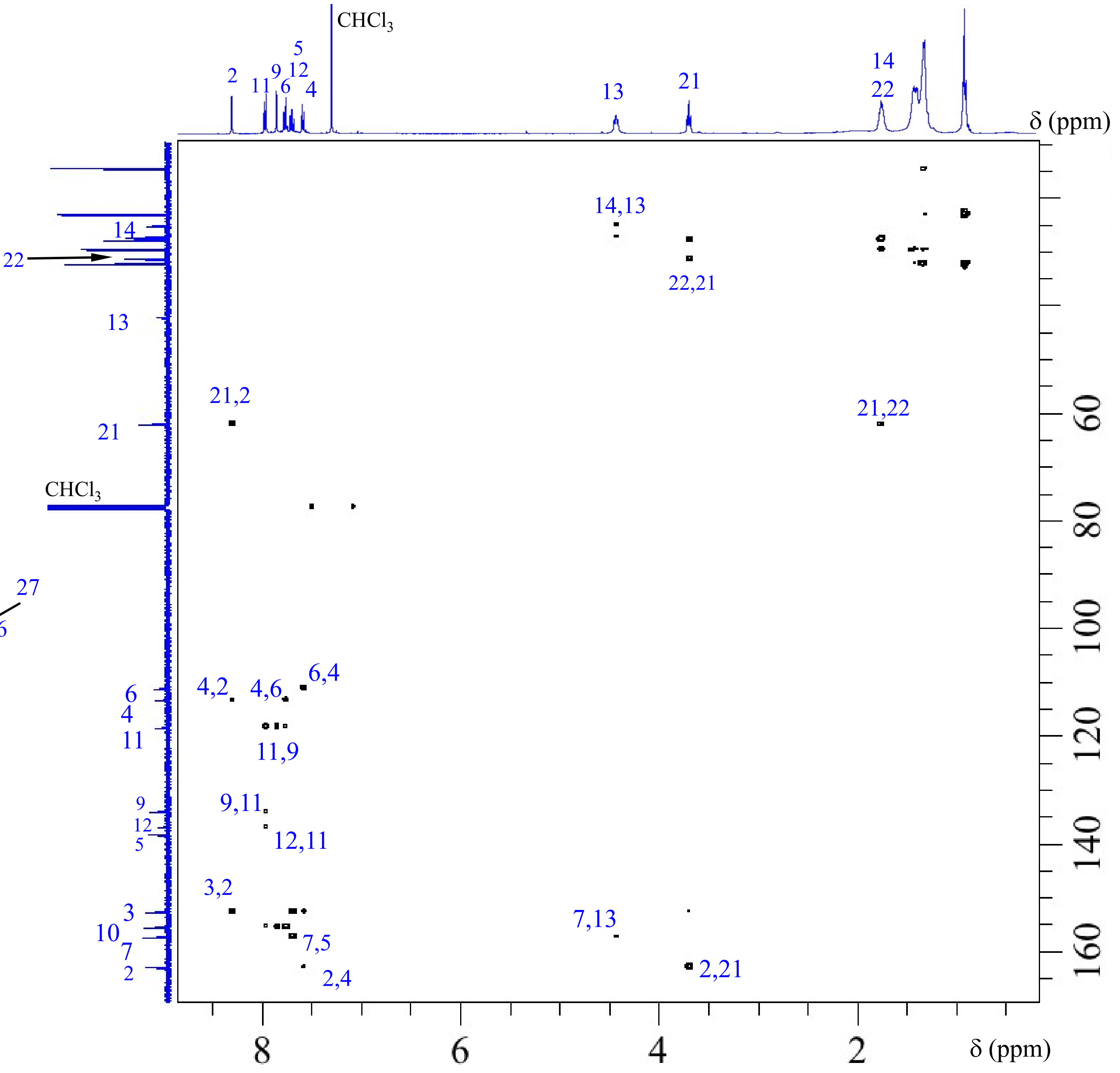

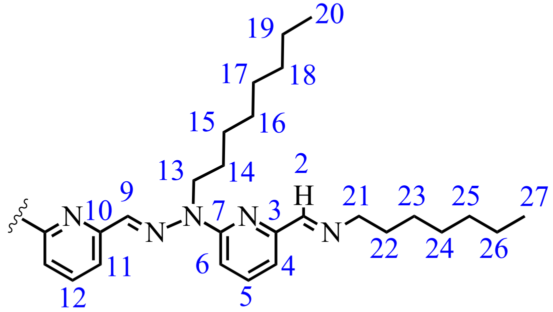

Part of the ^{1}H-^{13}C HMBC NMR spectrum (500 MHz, $CDCl_3$) of compound **AL3$_2$**

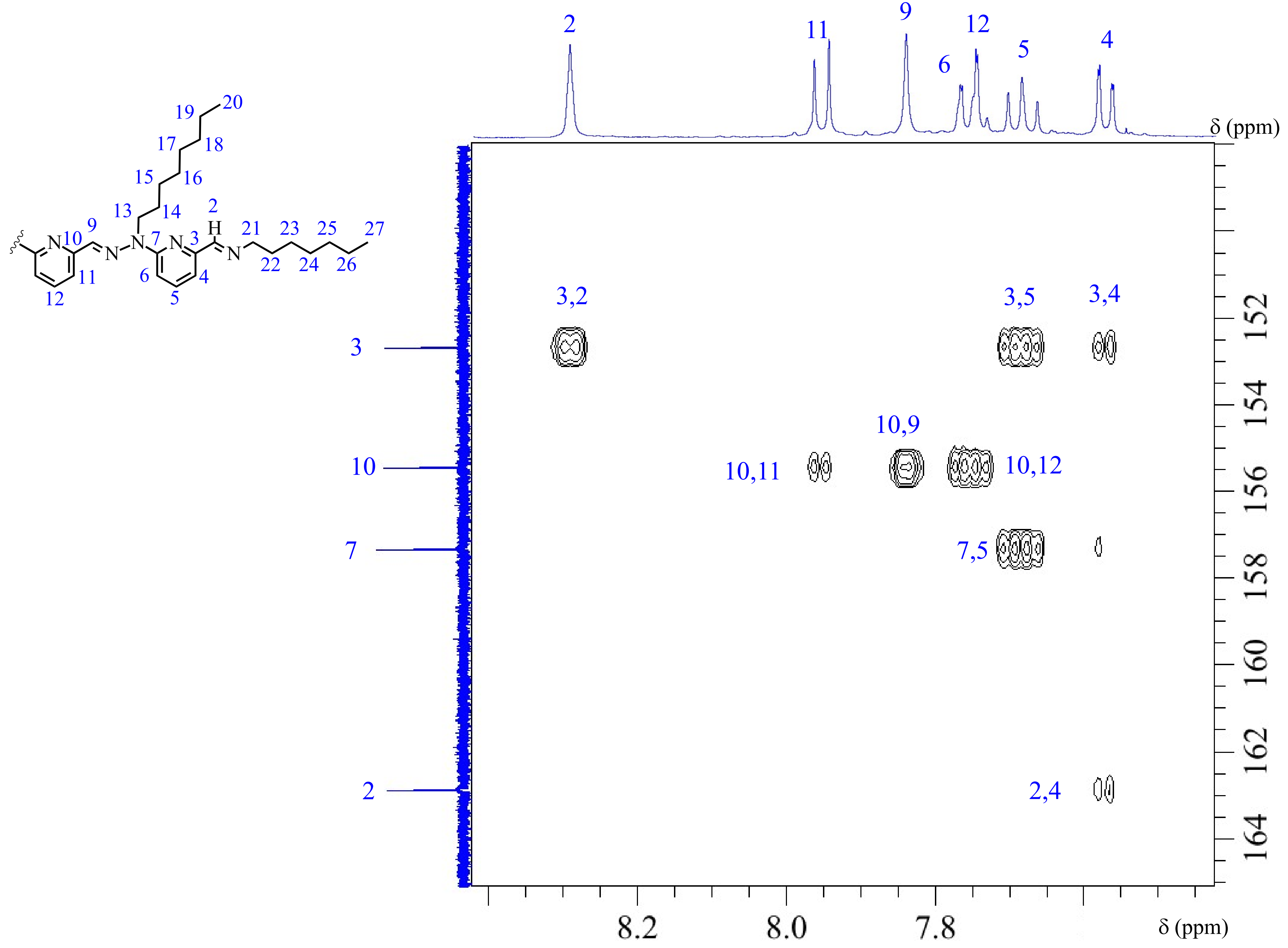

NMR spectra of compound **triim**

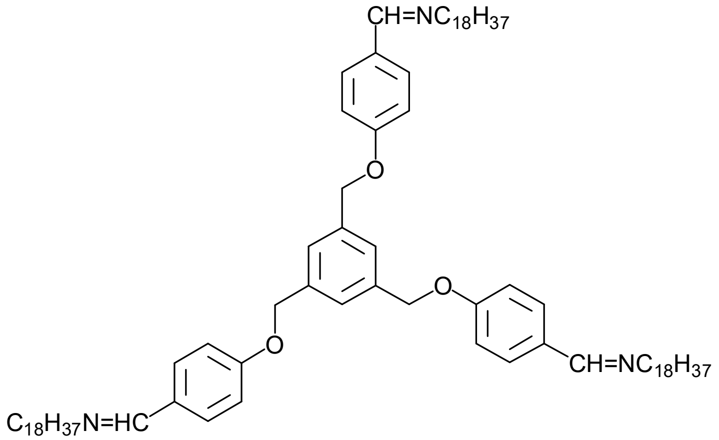

1D ^{1}H NMR spectrum (500 MHz, $CDCl_3$, δ_{ref} = 7.26 ppm) and ^{1}H DOSY NMR spectrum ($CDCl_3$, 500 MHz) of compound **triim**

1D ^{1}H NMR (400 MHz, $CDCl_3$, δ_{ref} = 7.26 ppm): 8.19 (s, 3H, -CH=N-), 7.70-7.62 (m, 6H), 7.47 (s, 3H), 7.03-6.94 (m, 6H), 5.12 (s, 6H, $-CH_2O-$), 3.62-3.50 (m, 6H, $=NCH_2-$), 1.74-1.60 (m, partly overlapped with water peak, 6H), 1.40-1.19 (m, partly overlapped with grease peak, 90H), 0.91-0.84 (m, partly overlapped with grease peak, 9H, $-CH_3$) ppm. ^{1}H DOSY NMR: see below.

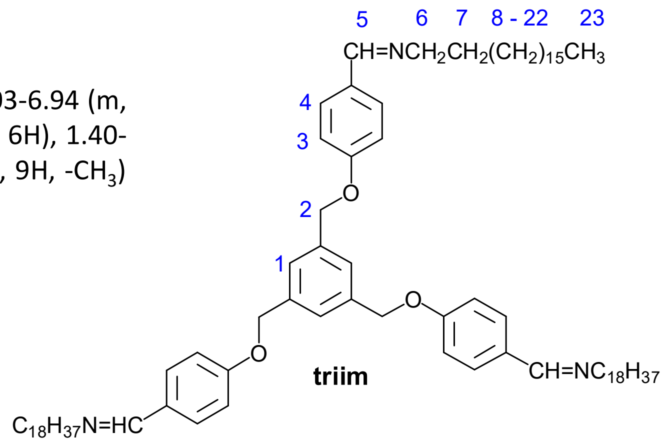

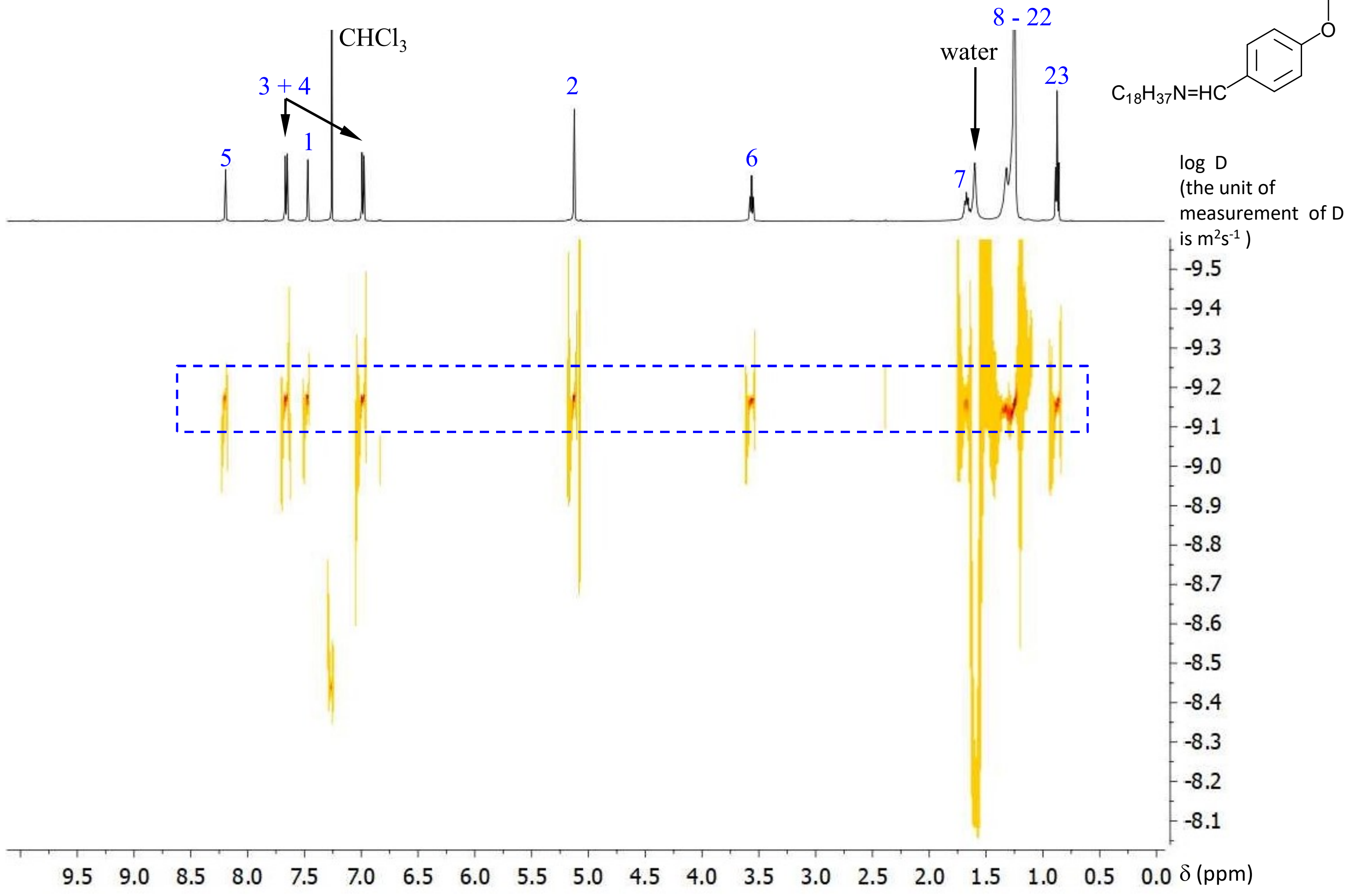

Electrospray ionization mass spectrometry
ESI-MS

ESI spectrum of compound

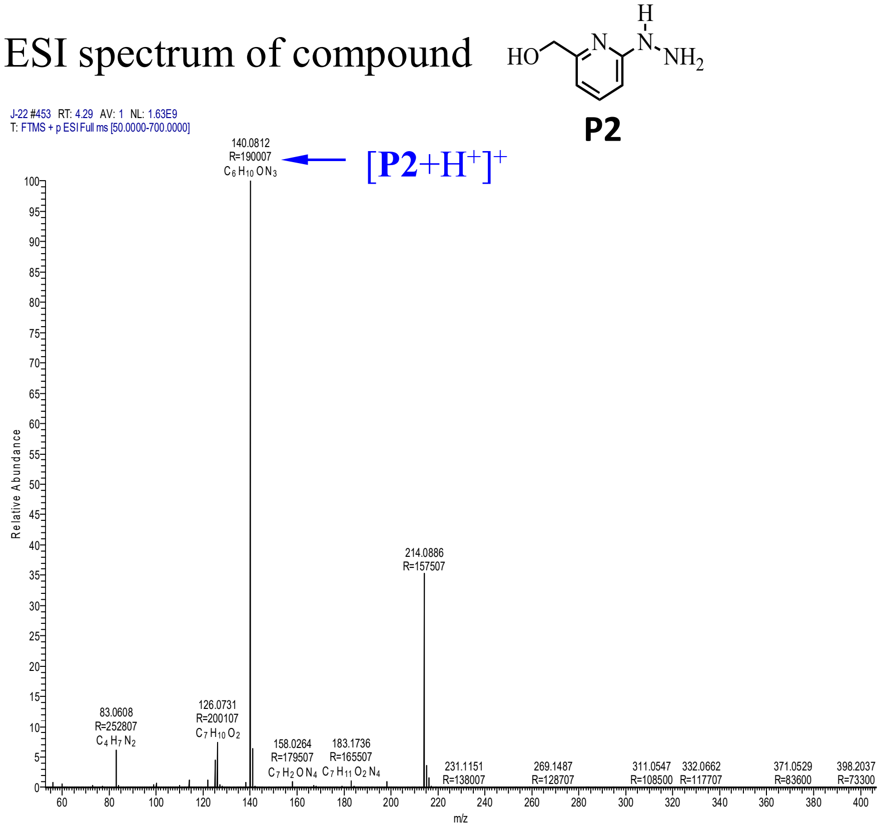

ESI spectrum of compound

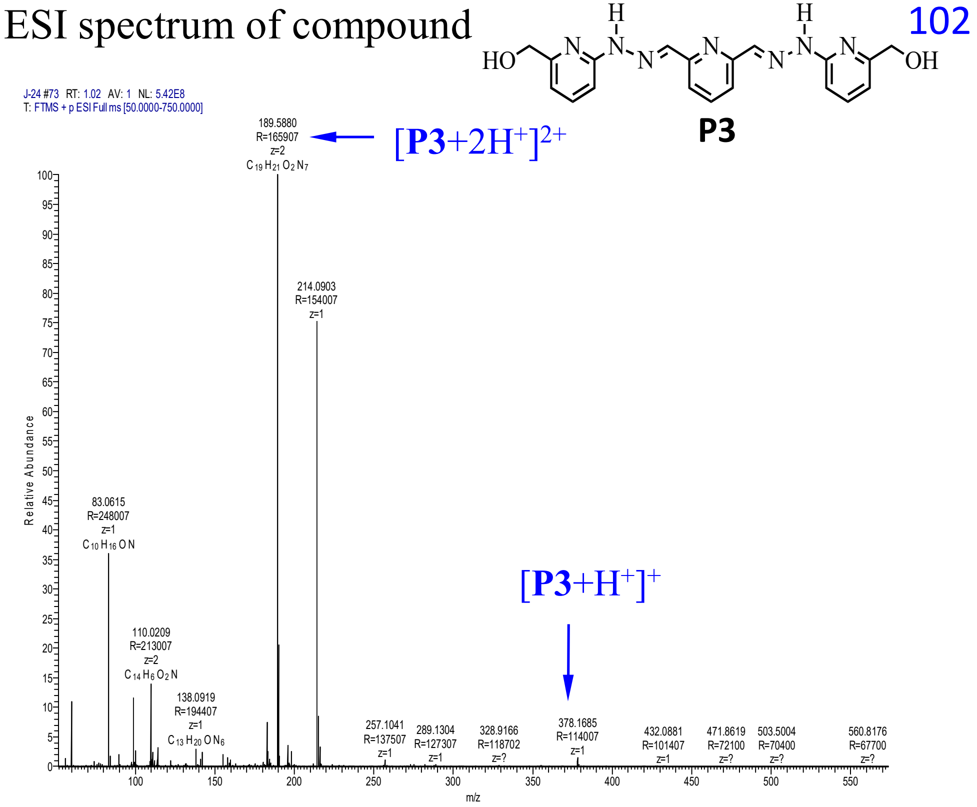

Isotope distribution (measured - top, calculated - bottom)

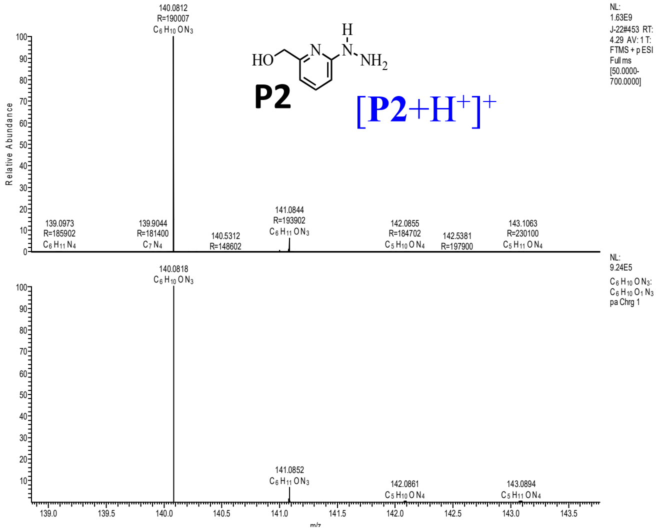

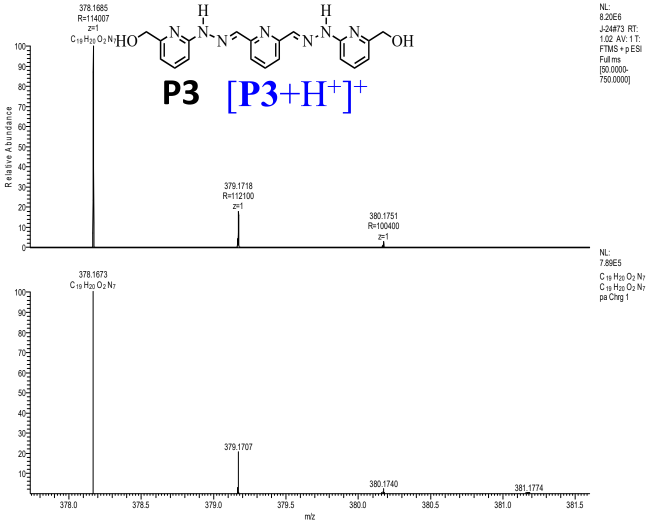

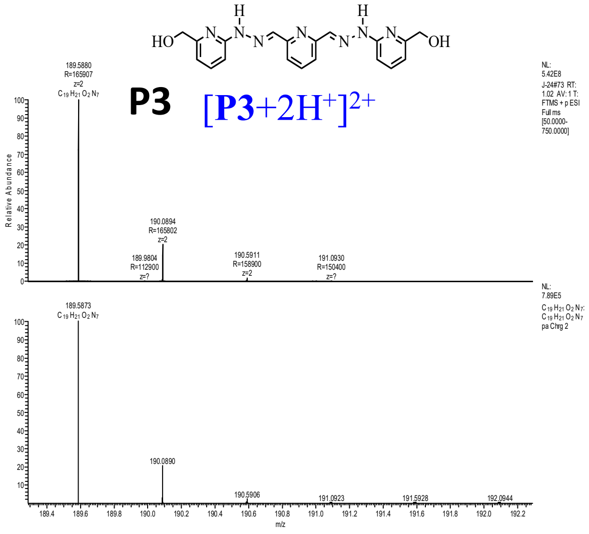

ESI spectrum of compound

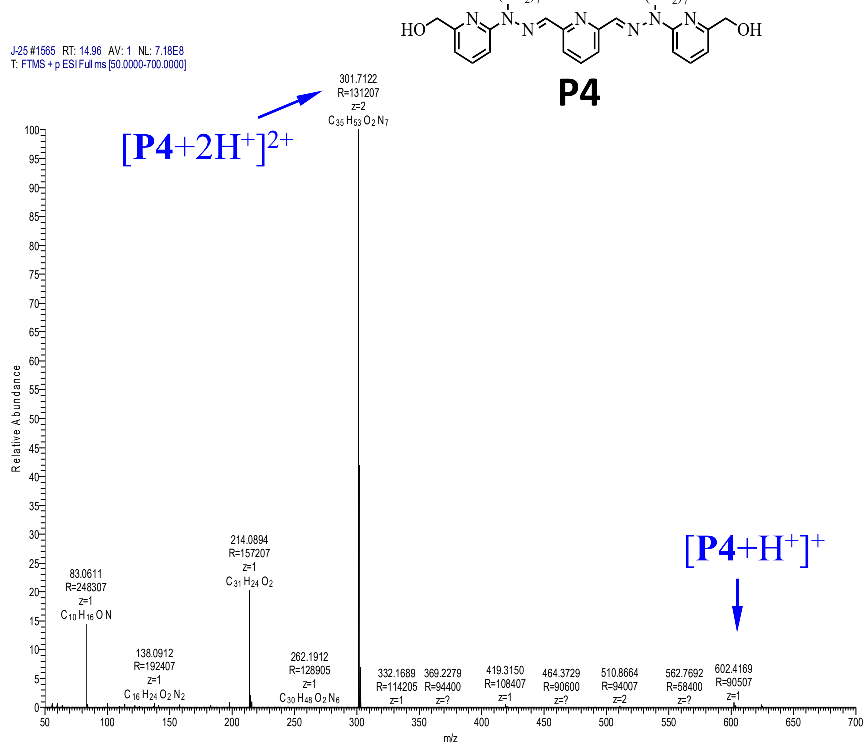

ESI spectrum of cpmpound

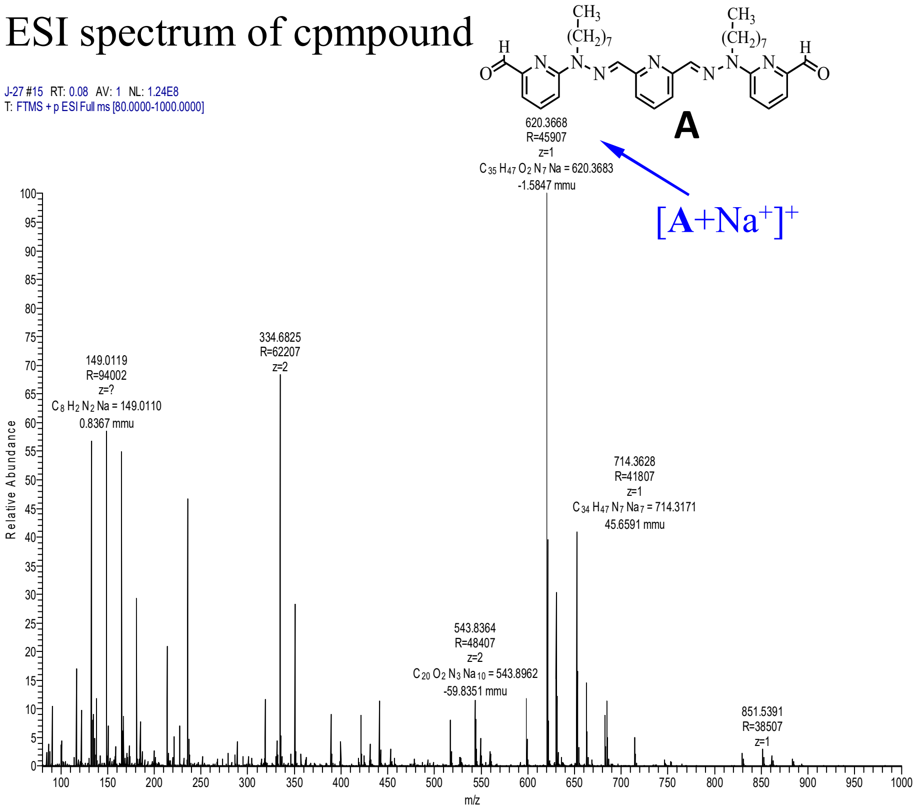

Isotope distribution (measured - top, calculated - bottom)

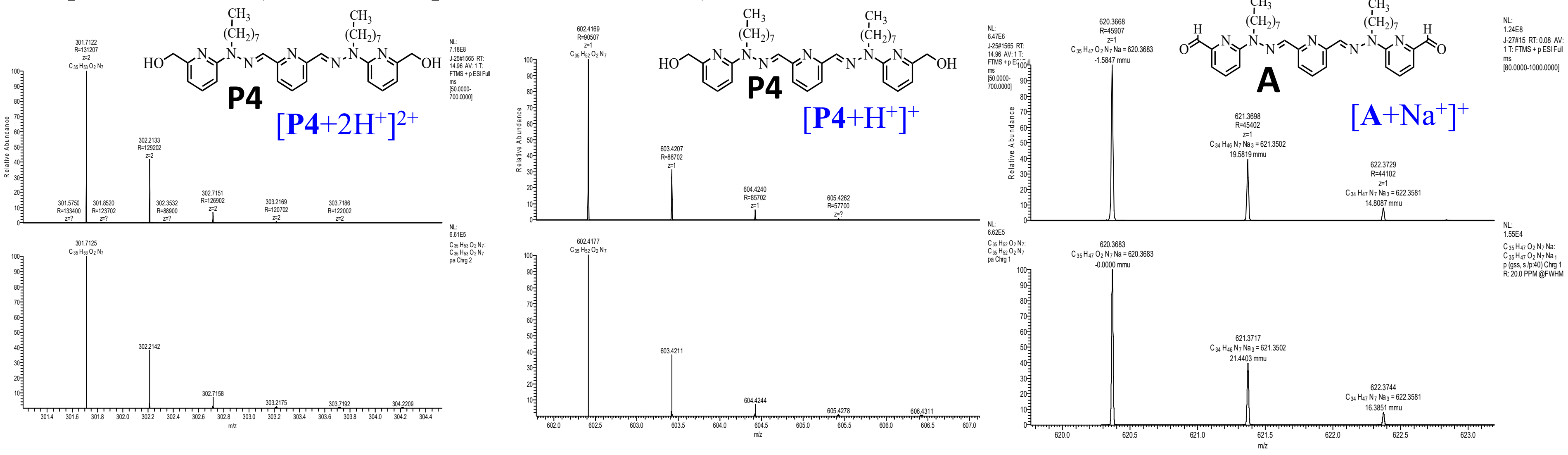

ESI spectrum of

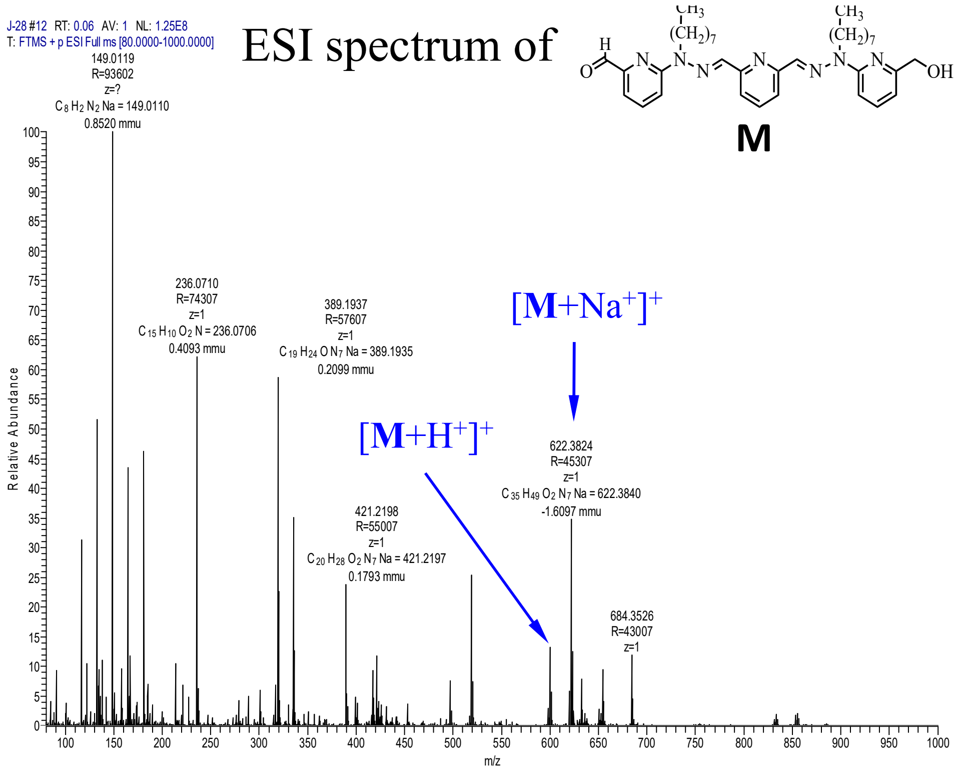

Isotope distribution (measured - top, calculated - bottom)

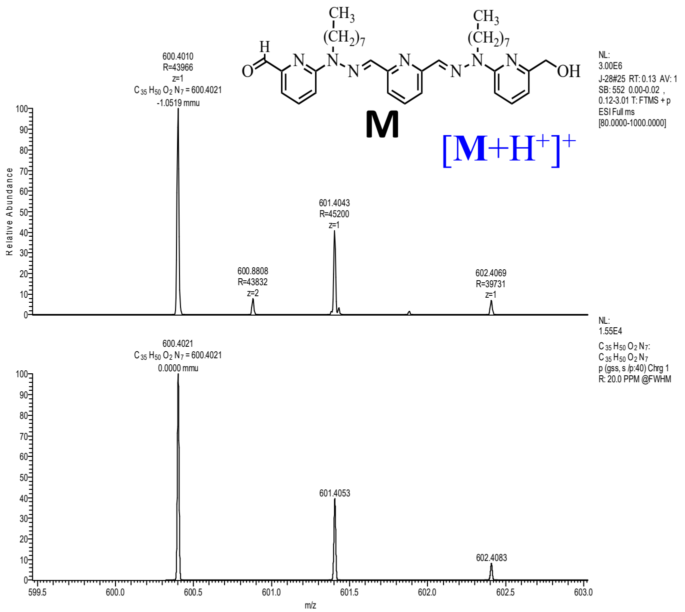

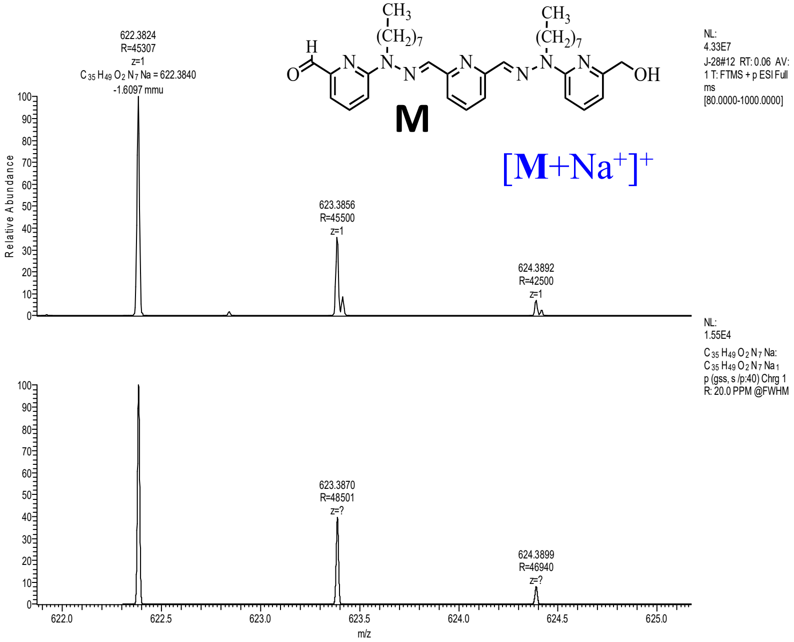

ESI spectrum of amine **R1**

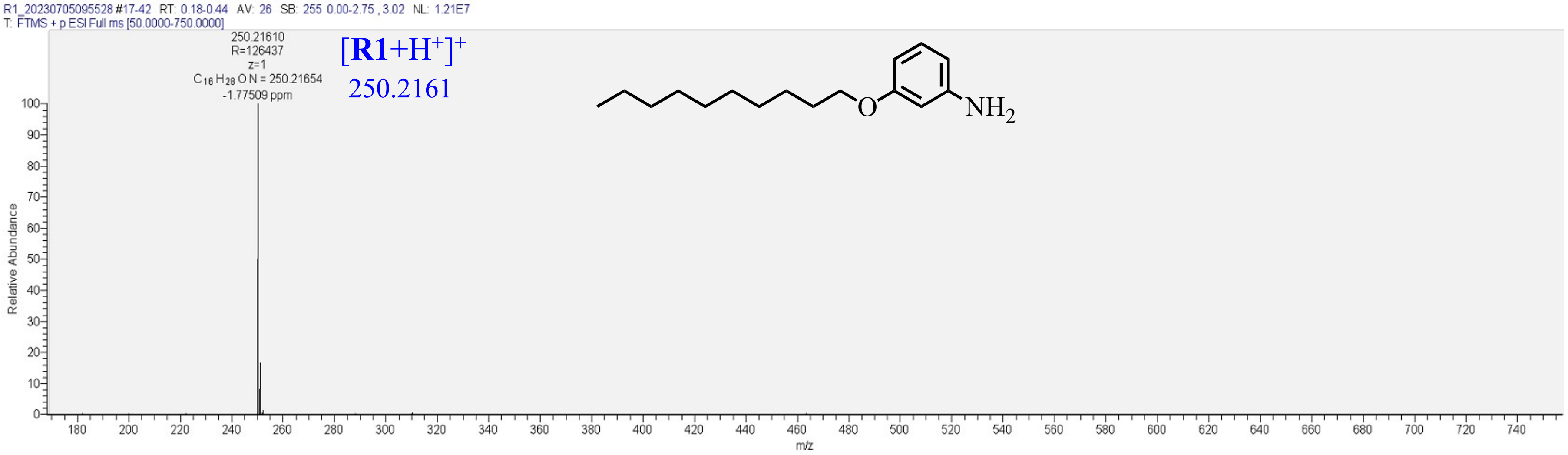

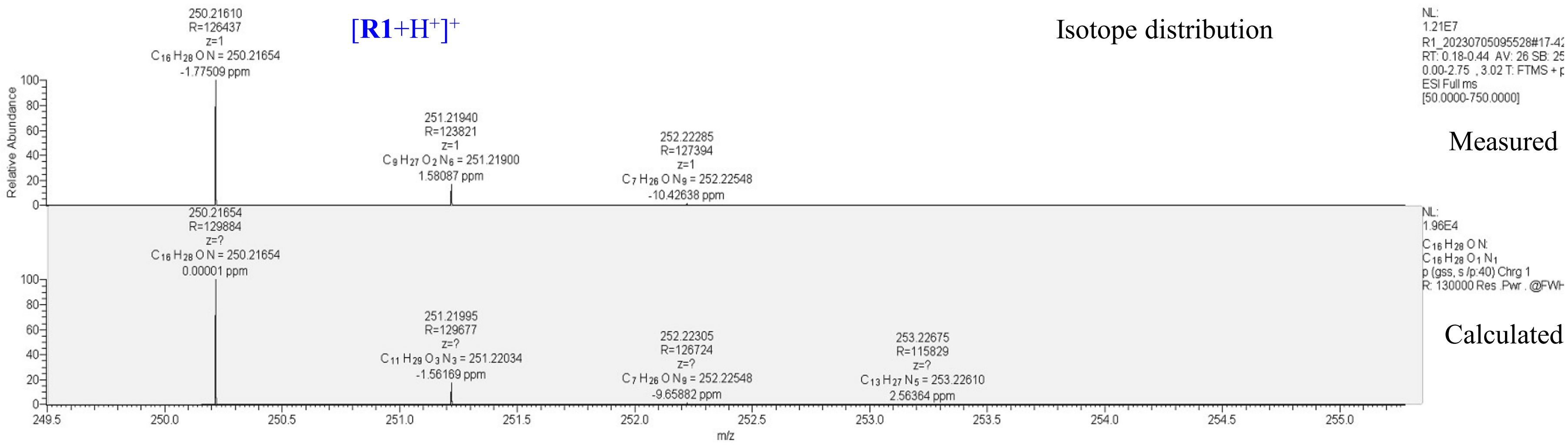

ESI spectrum of amine **R2**

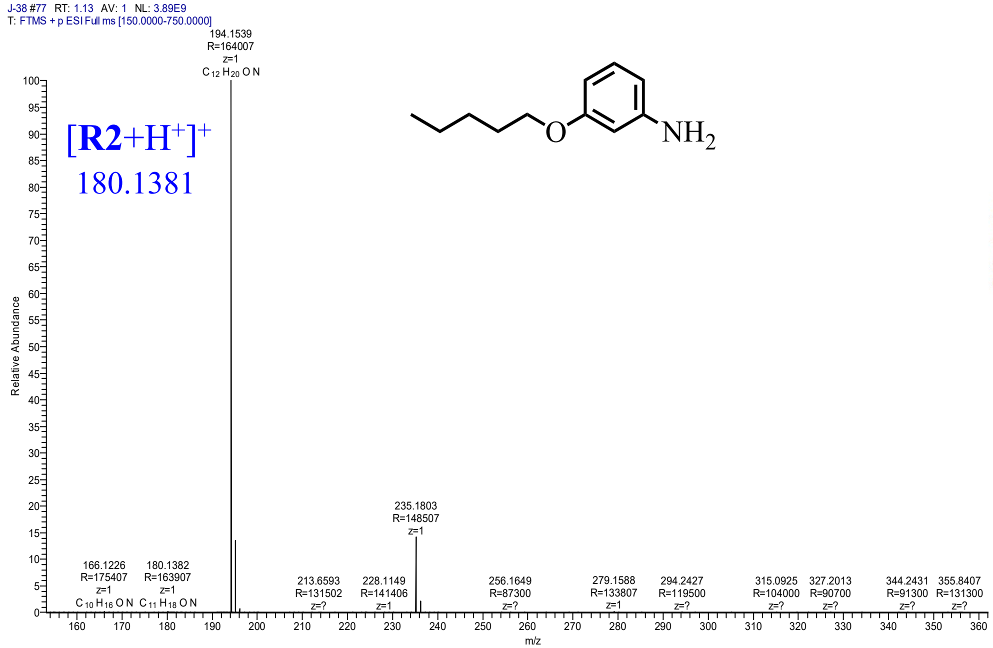

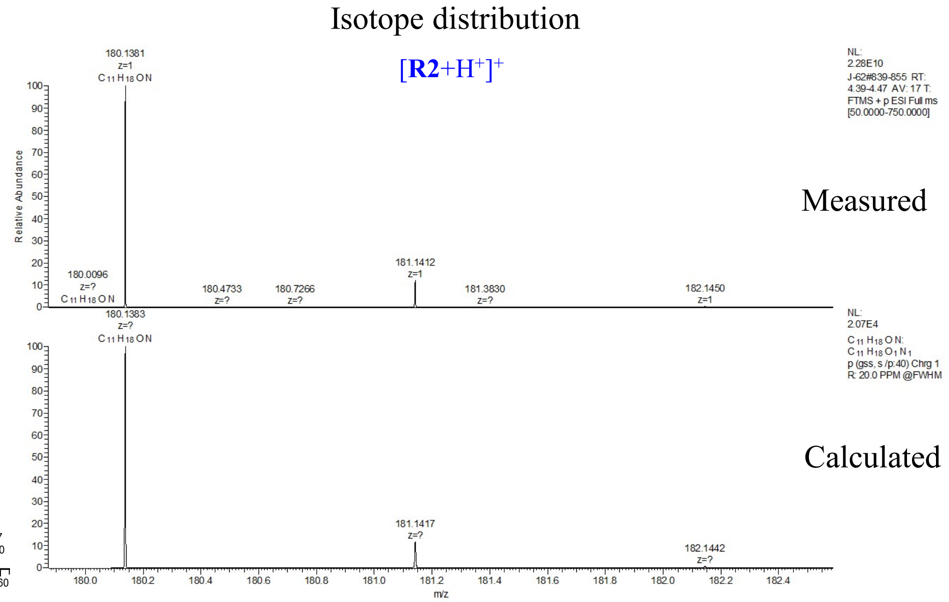

ESI spectrum of amine **R4**

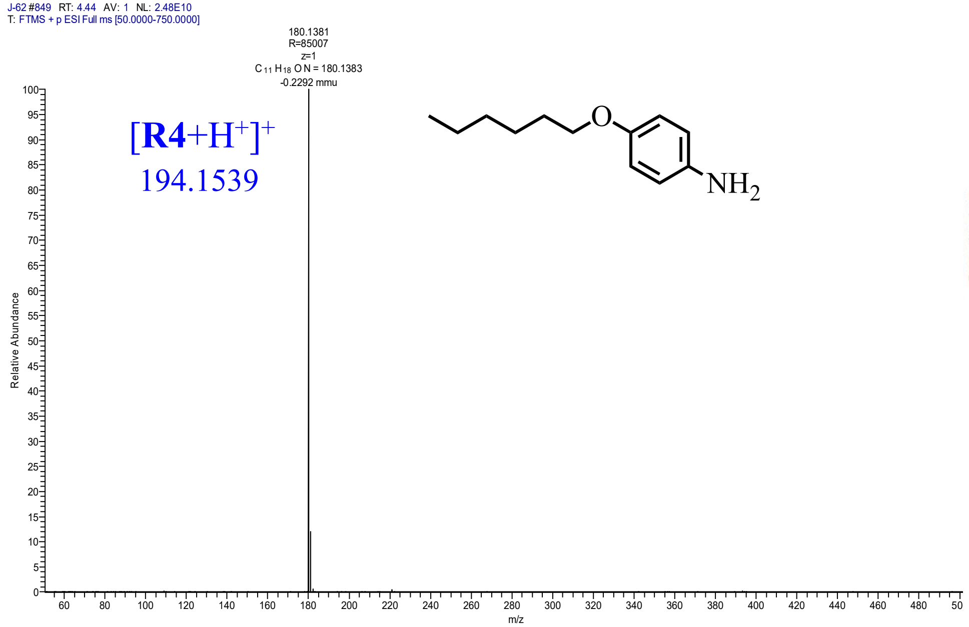

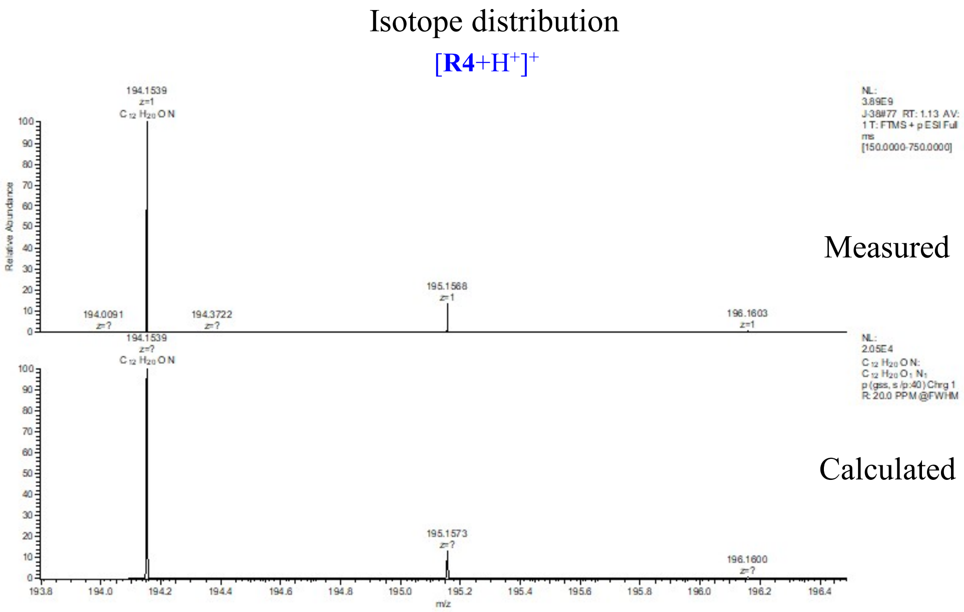

ESI spectrum of compound $\mathbf{AL3_2}$

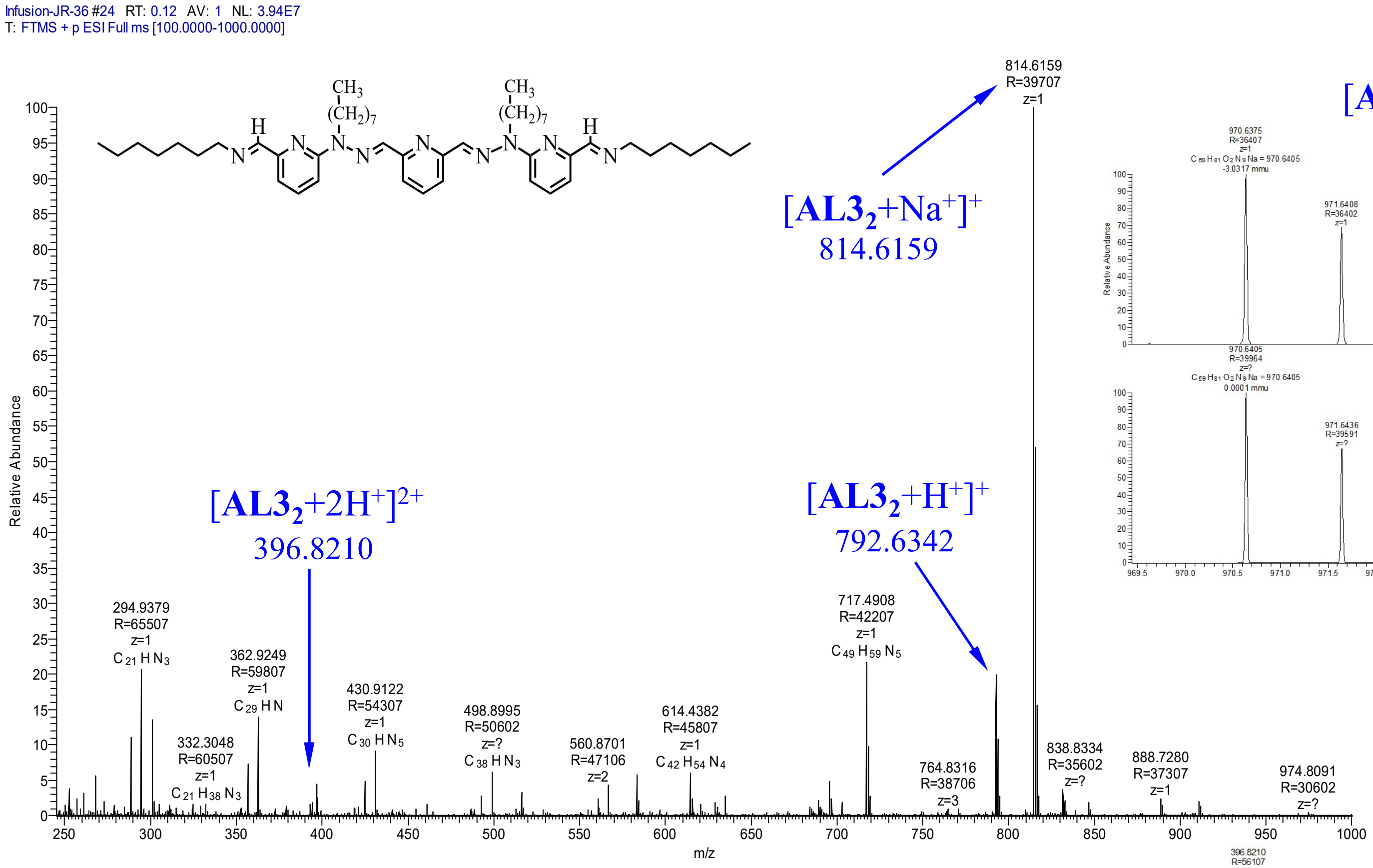

Isotope distribution

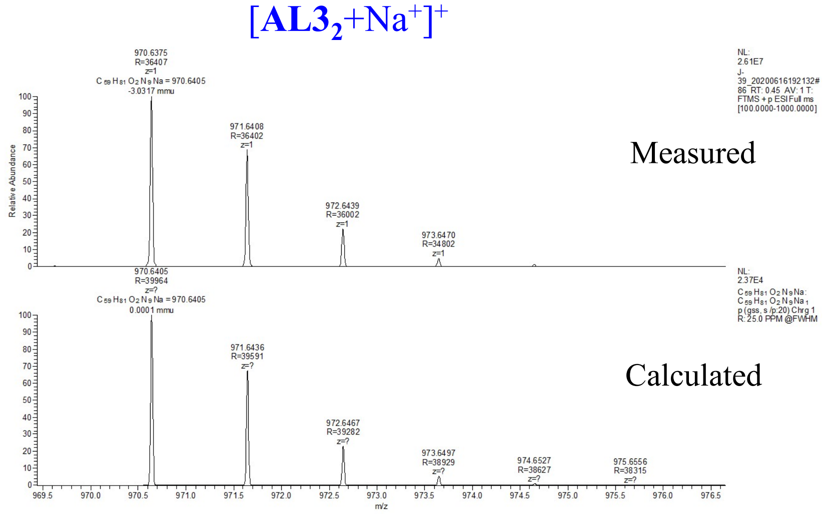

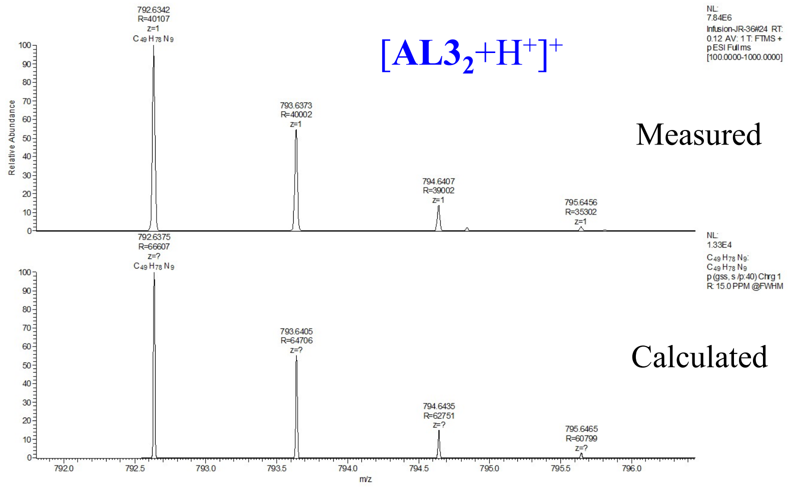

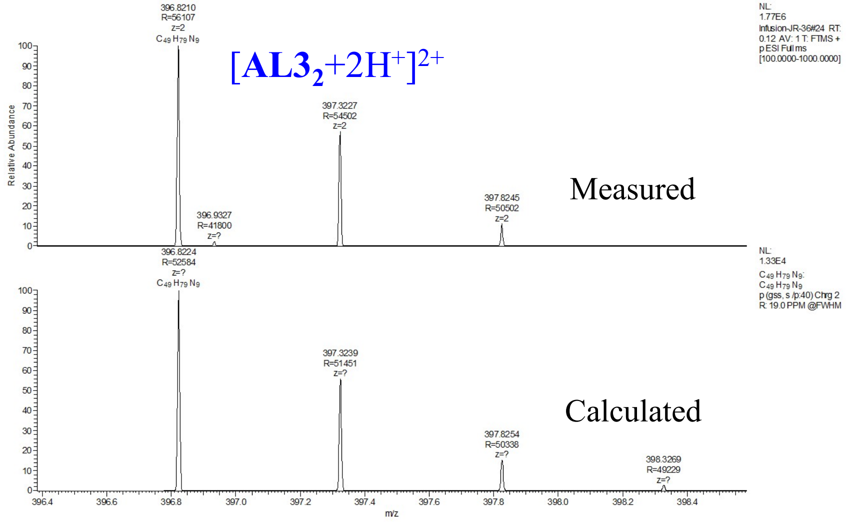

ESI spectrum of compound **AR4$_2$**

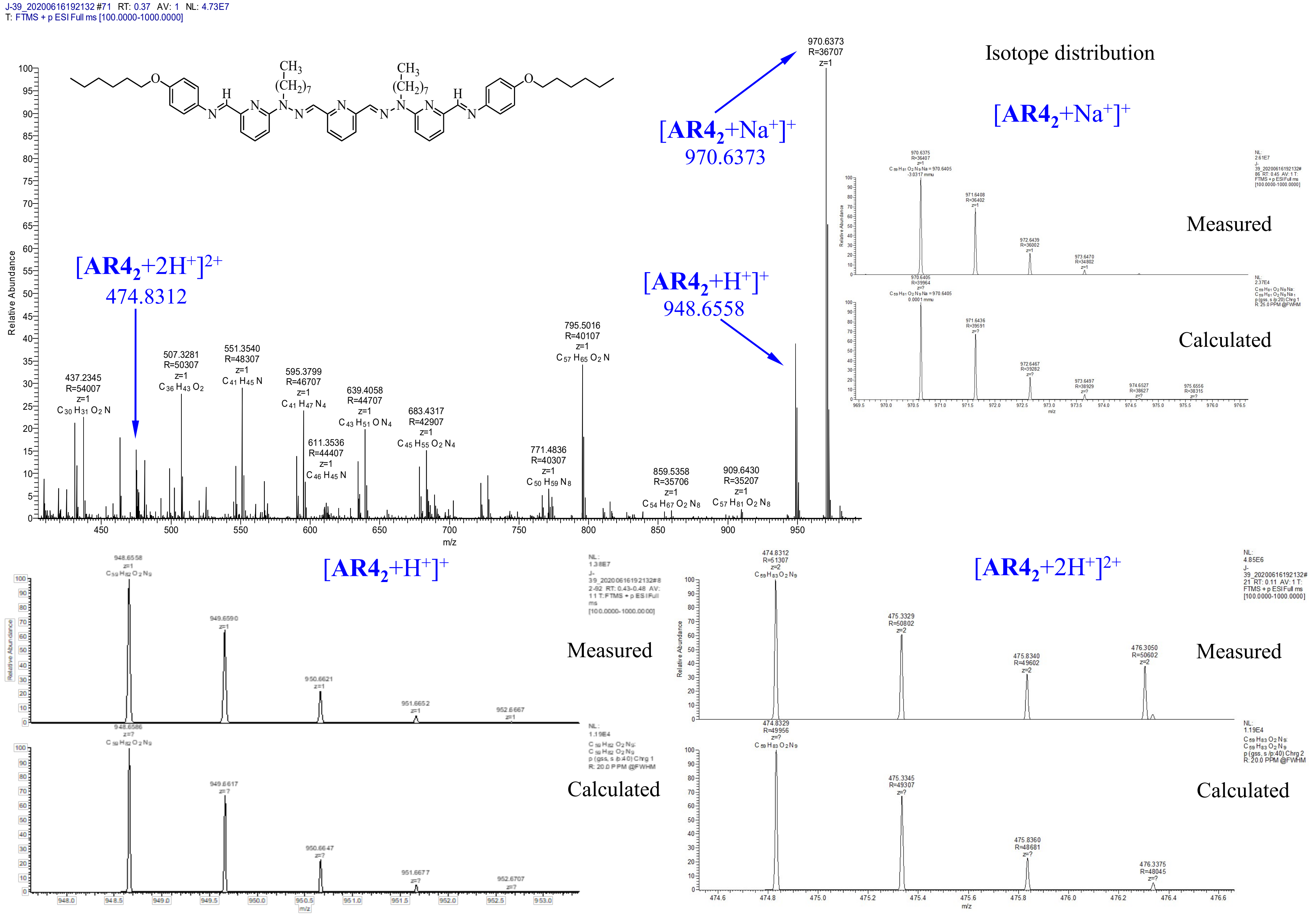

^{1}H DOSY NMR spectra for reactions (500 MHz)

- Formation of bis-imines
- Formation of imines
- bis-imine/amine exchanges in solution
- Imine/aldehyde exchanges in solution
- Solvent-free exchanges

Chemical exchanges may affect the diffusion coefficients and the peaks.

Formation of bis-imines

- 2,6-pyridinedicarboxaldehyde with 2 equiv. of *p*-toluidine
- 2,6-pyridinedicarboxaldehyde with 2 equiv. of 4-(hexyloxy)aniline
- 2,6-pyridinedicarboxaldehyde with 2 equiv. of methyl-4-aminobenzoate
- 2,6-pyridinedicarboxaldehyde with 2 equiv. of 4-aminobenzonitrile
- 2,6-pyridinedicarboxaldehyde with 2 equiv. of 4-acetylaniline

^{1}H DOSY NMR spectrum ($CDCl_3$, 500 MHz) of a reaction mixture (before equilibrium) of 1 equiv. of 2,6-pyridinedicarboxaldehyde with 2 equiv. of *p*-toluidine.

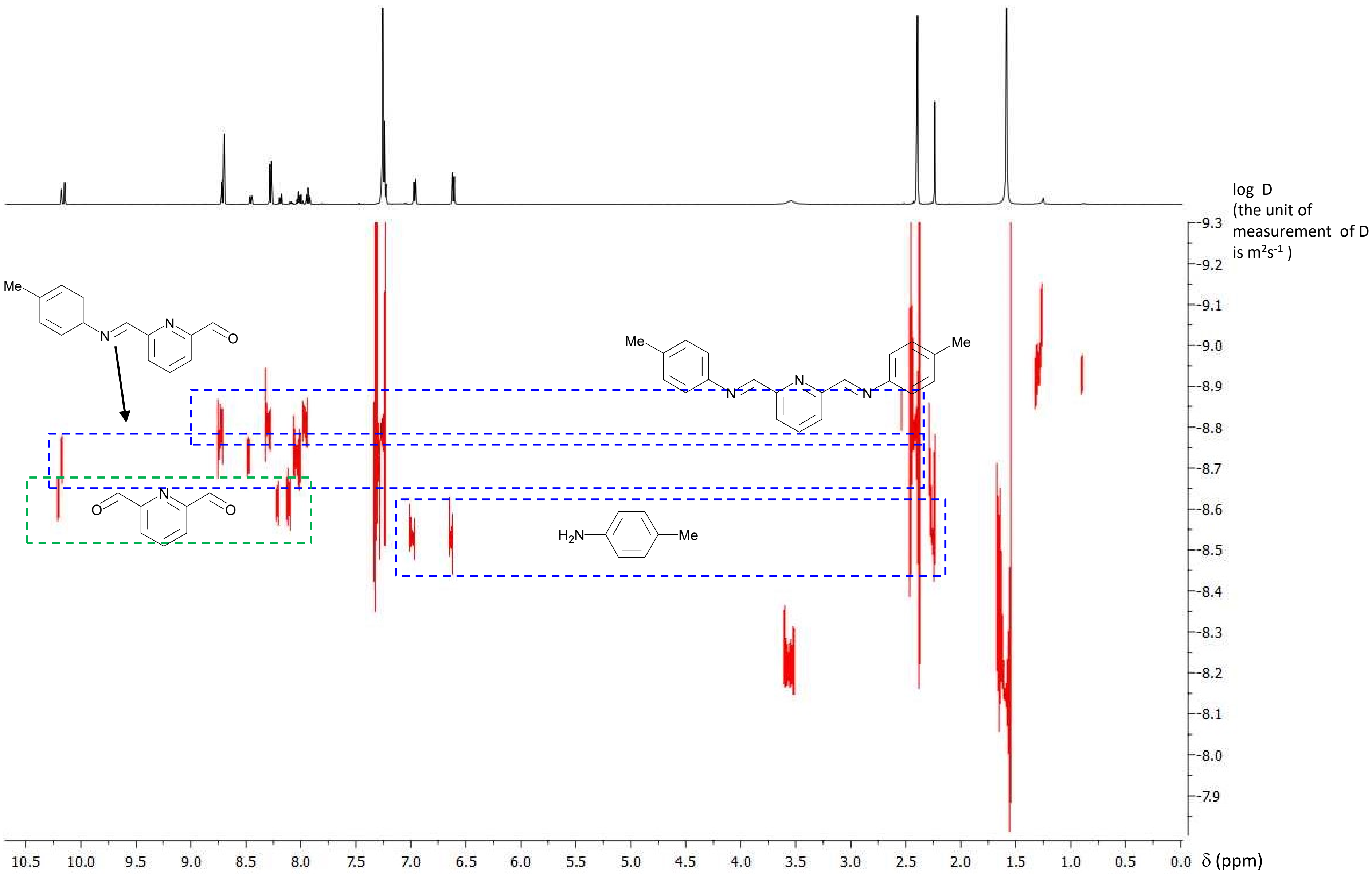

^{1}H DOSY NMR spectrum ($CDCl_3$, 500 MHz) of a reaction mixture (before equilibrium) of 1 equiv. of 2,6-pyridinedicarboxaldehyde with 2 equiv. of 4-(hexyloxy)aniline.

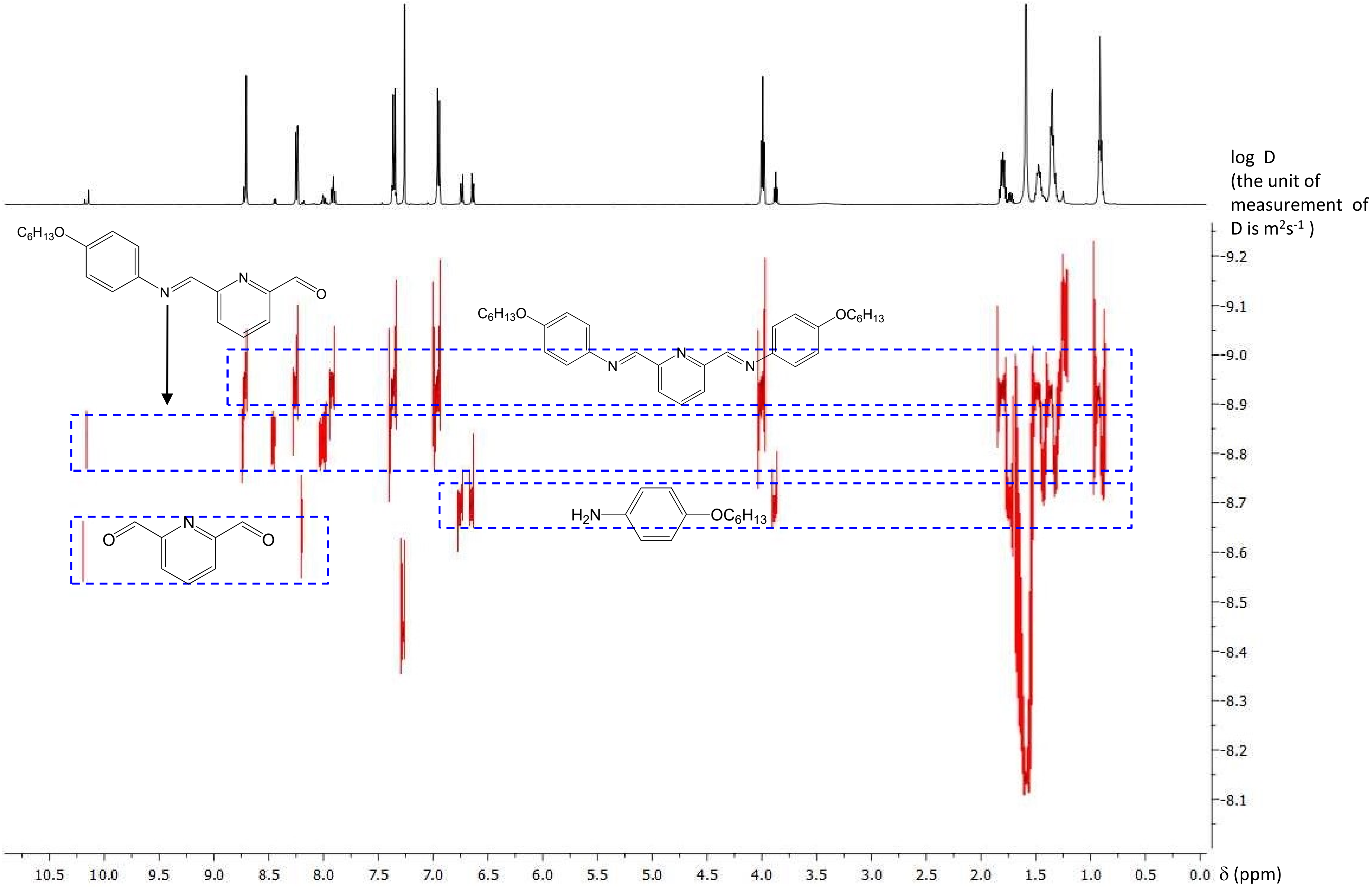

^{1}H DOSY NMR spectrum ($CDCl_3$, 500 MHz) of a reaction mixture of 1 equiv. of 2,6-pyridinedicarboxaldehyde with 2 equiv. of methyl-4-aminobenzoate (equilibrium not reached).

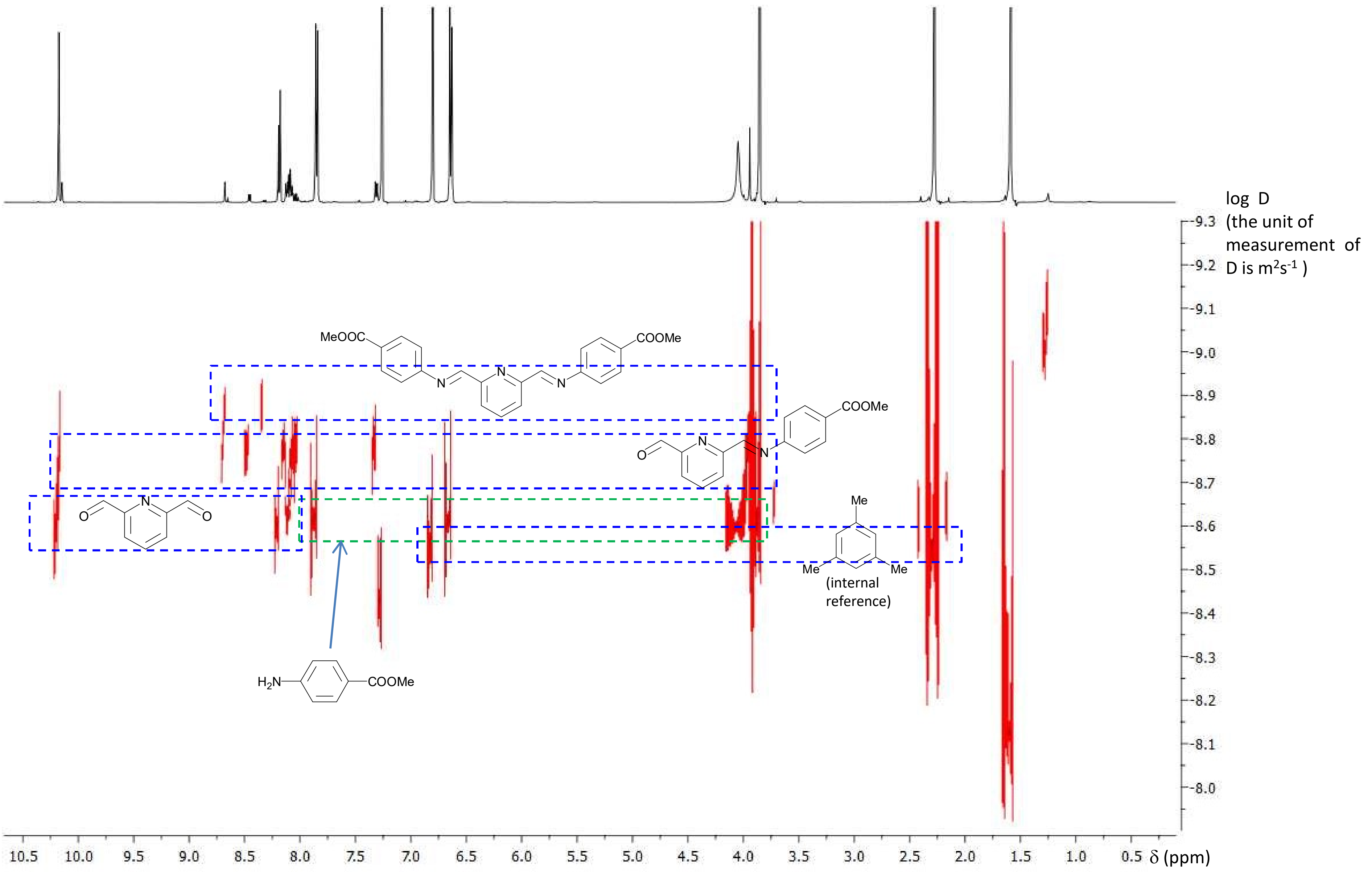

^{1}H DOSY NMR spectrum ($CDCl_3$, 500 MHz) of a reaction mixture (before equilibrium) of 1 equiv. of 2,6-pyridinedicarboxaldehyde with 2 equiv. of 4-aminobenzonitrile.

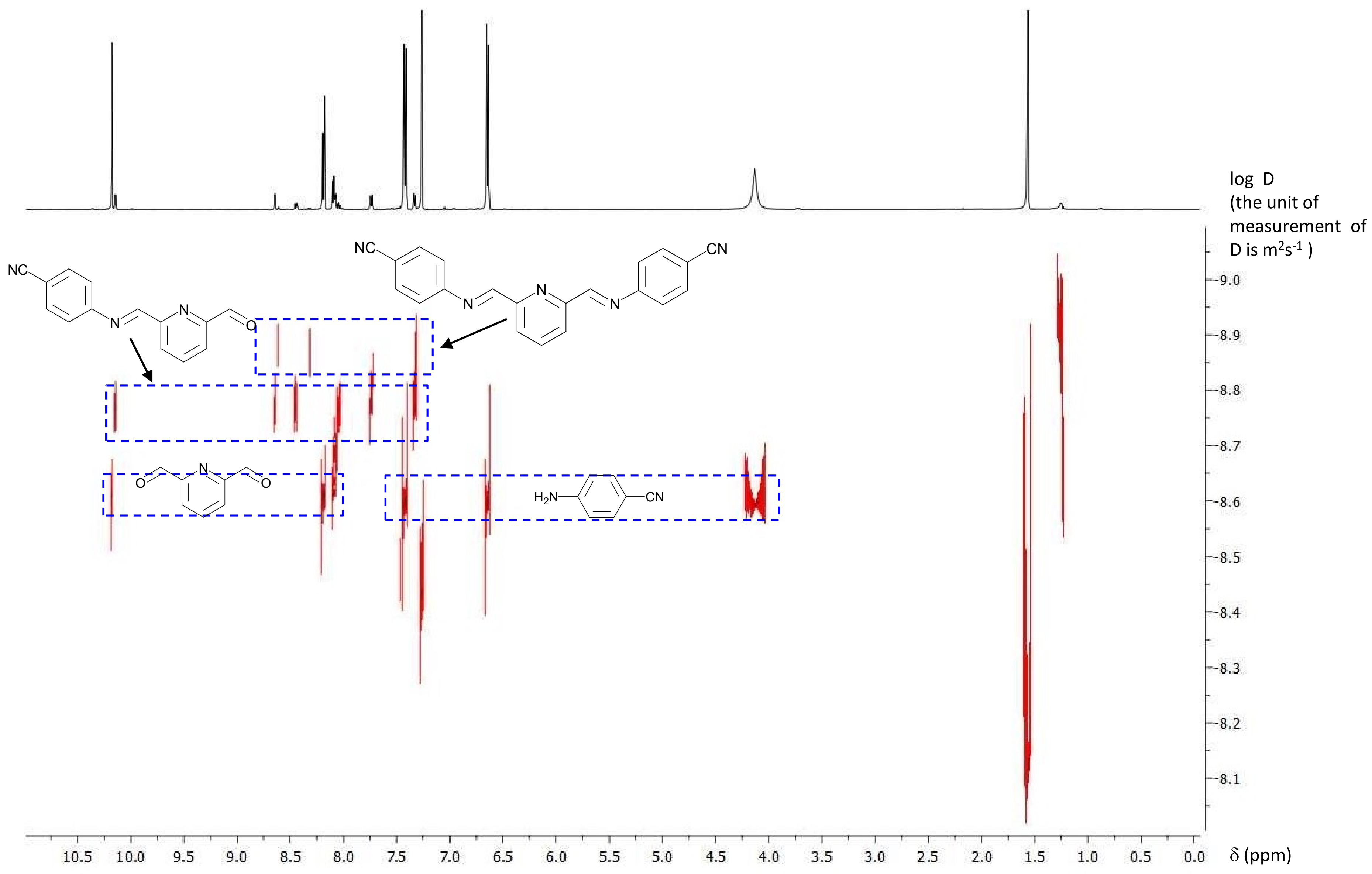

^{1}H DOSY NMR spectrum ($CDCl_3$, 500 MHz) of a reaction mixture (before equilibrium) of 1 equiv. of 2,6-pyridinedicarboxaldehyde with 2 equiv. of 4-acetylaniline.

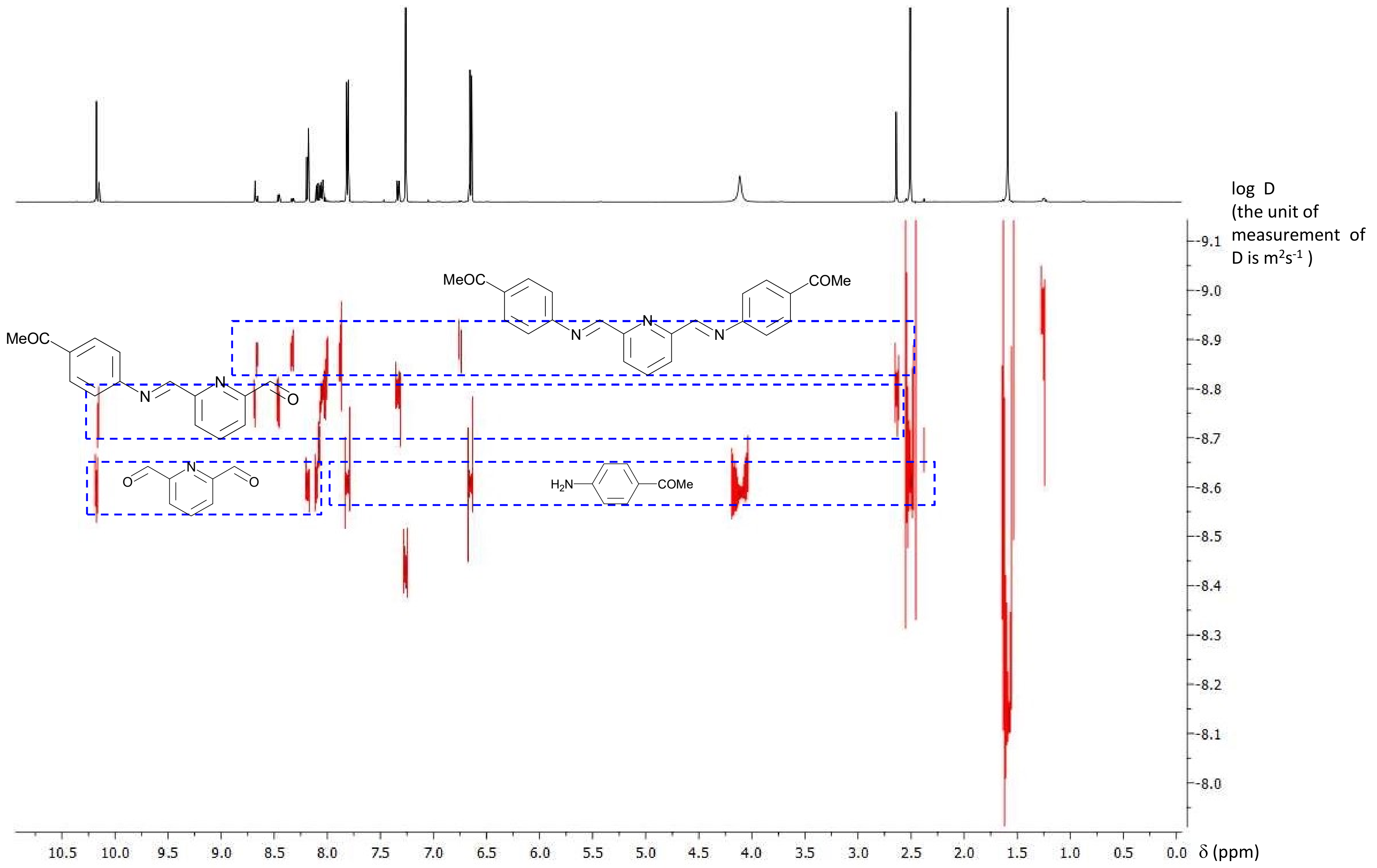

Formation of imines

- 6-bromo-2-pyridinecarboxaldehyde with 1.1 equiv. of 4-(hexyloxy)aniline
- 6-bromo-2-pyridinecarboxaldehyde with 1 equiv. of 4-aminoacetanilide

^{1}H DOSY NMR spectrum ($CDCl_3$, 500 MHz) of a reaction mixture (at equilibrium) of 1 equiv. of 6-bromo-2-pyridinecarboxaldehyde with 1.1 equiv. of 4-(hexyloxy)aniline.

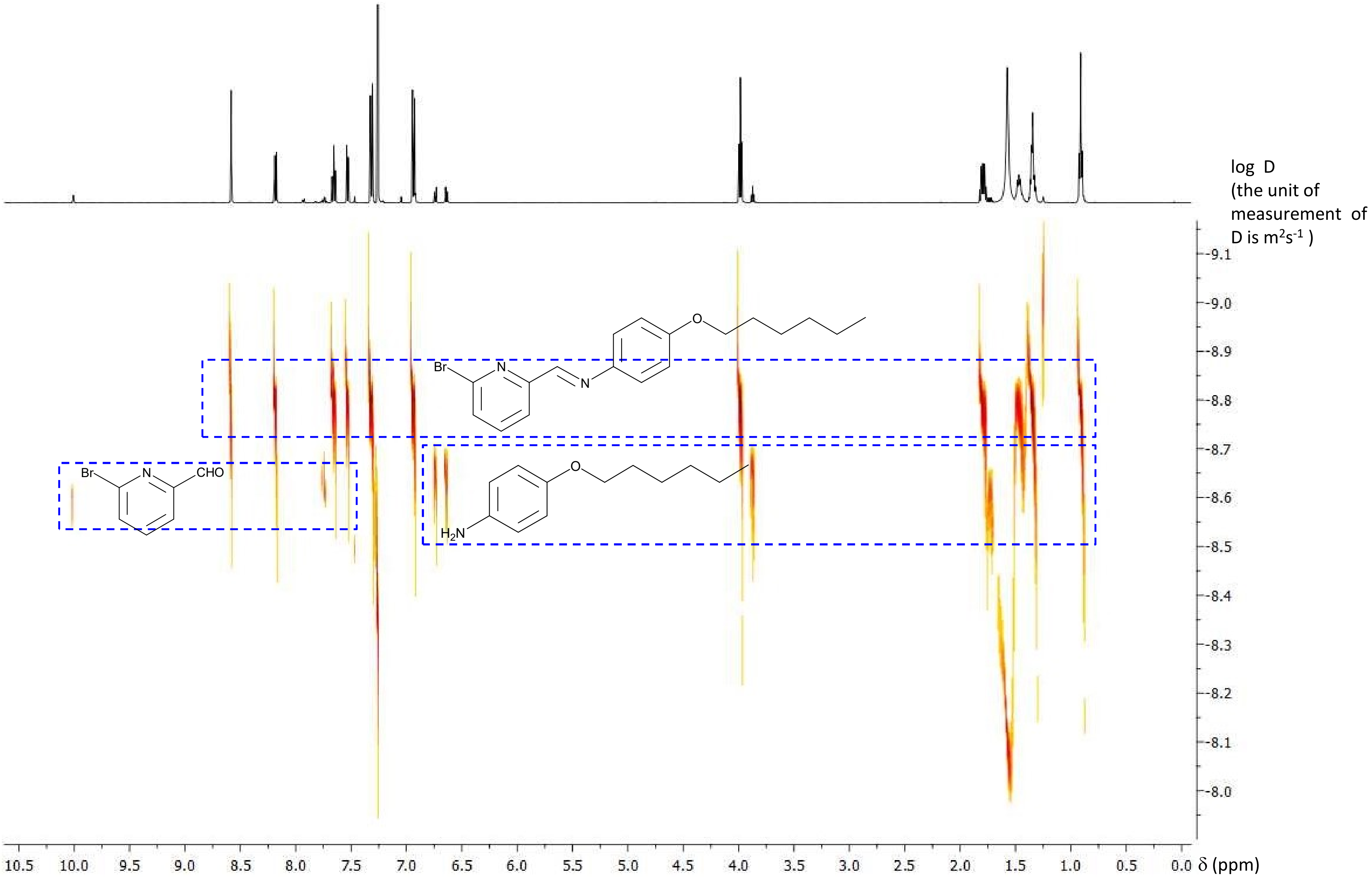

^1H DOSY NMR spectrum (CD_3CN, 500 MHz) of a reaction mixture (at equilibrium) of 1 equiv. of 6-bromo-2-pyridinecarboxaldehyde with 1 equiv. of 4-aminoacetanilide.

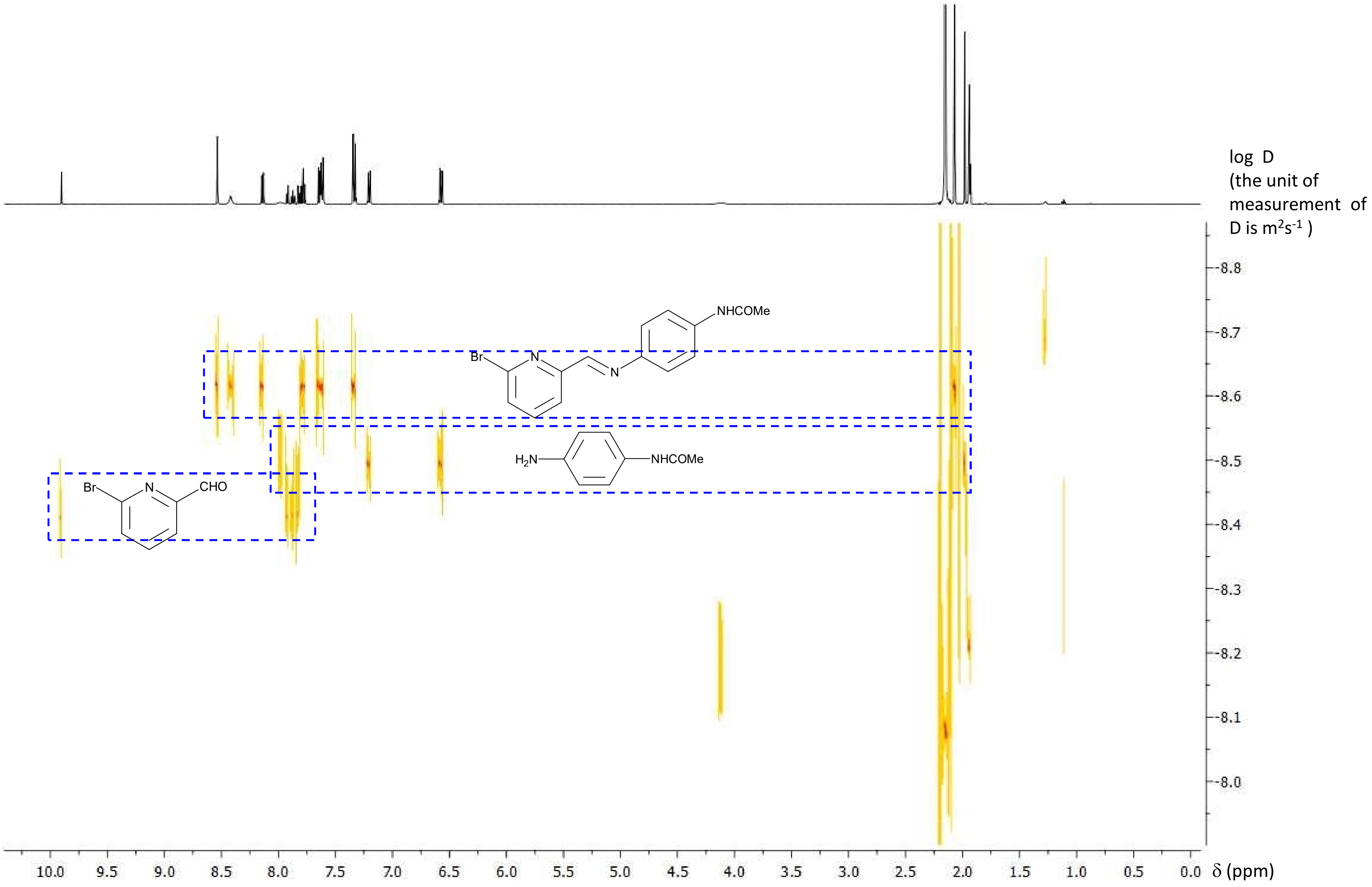

bis-imine/amine exchanges in solution

- 2,6-pyridinedicarboxaldehyde with 4.5 equiv. of *p*-toluidine, followed by addition of 4.5 equiv. of decylamine

- 2,6-pyridinedicarboxaldehyde with 4.5 equiv. of *p*-toluidine, followed, after equilibration, by addition of 4.5 equiv. of decylamine, equilibration and addition of 4.5 equiv. of TFA (= CF_3COOH)

^{1}H DOSY NMR spectrum ($CDCl_3$, 500 MHz) of a reaction mixture (at equilibrium) of 1 equiv. of 2,6-pyridinedicarboxaldehyde with 4.5 equiv. of *p*-toluidine, followed by addition of 4.5 equiv. of decylamine.

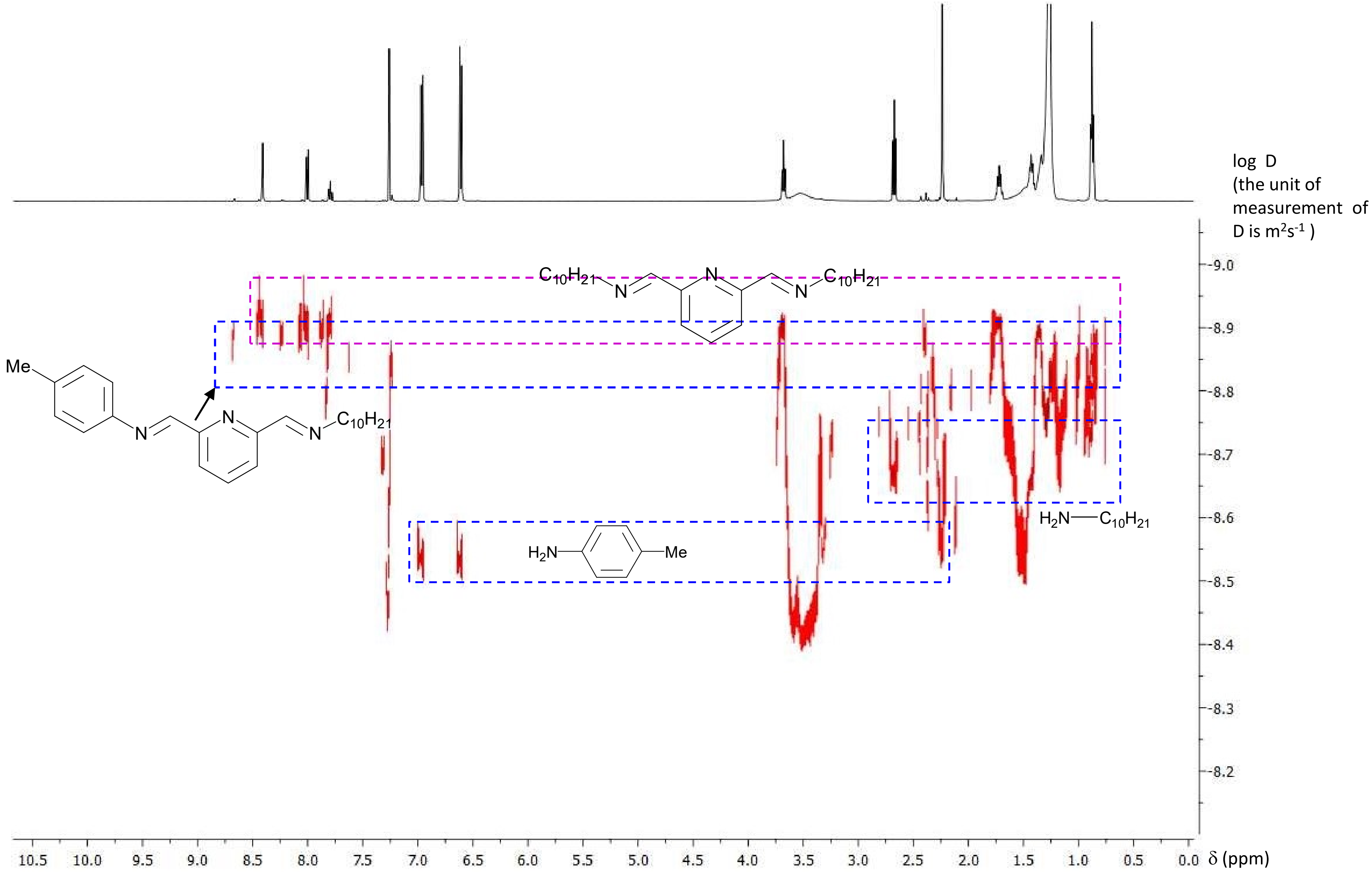

^1H DOSY NMR spectrum ($CDCl_3$, 500 MHz) of a reaction mixture (at equilibrium) of 1 equiv. of 2,6-pyridinedicarboxaldehyde with 4.5 equiv. of *p*-toluidine, followed, after equilibration, by addition of 4.5 equiv. of decylamine, equilibration and addition of 4.5 equiv. of TFA (= CF_3COOH).

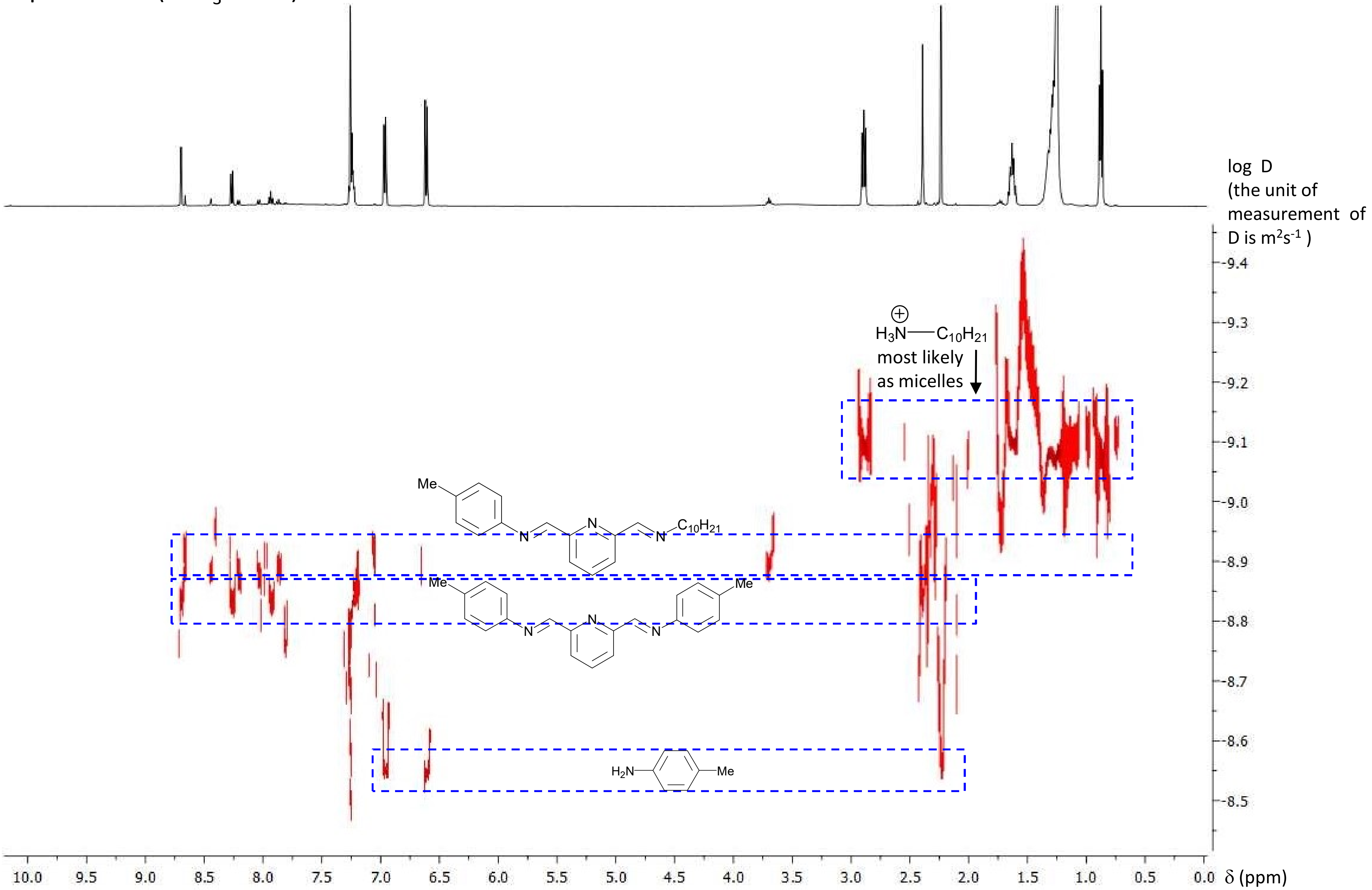

Imine/aldehyde exchanges in solution

- imine from 6-bomo-2-pyridinecarboxaldehyde and octadecylamine, with 1 equiv. of 2,6-dichlorobenzaldehyde

^1H DOSY NMR spectrum ($CDCl_3$, 500 MHz) of the equilibrated reaction mixture of the imine from 6-bomo-2-pyridinecarboxaldehyde and octadecylamine, with 1 equiv. of 2,6-dichlorobenzaldehyde.

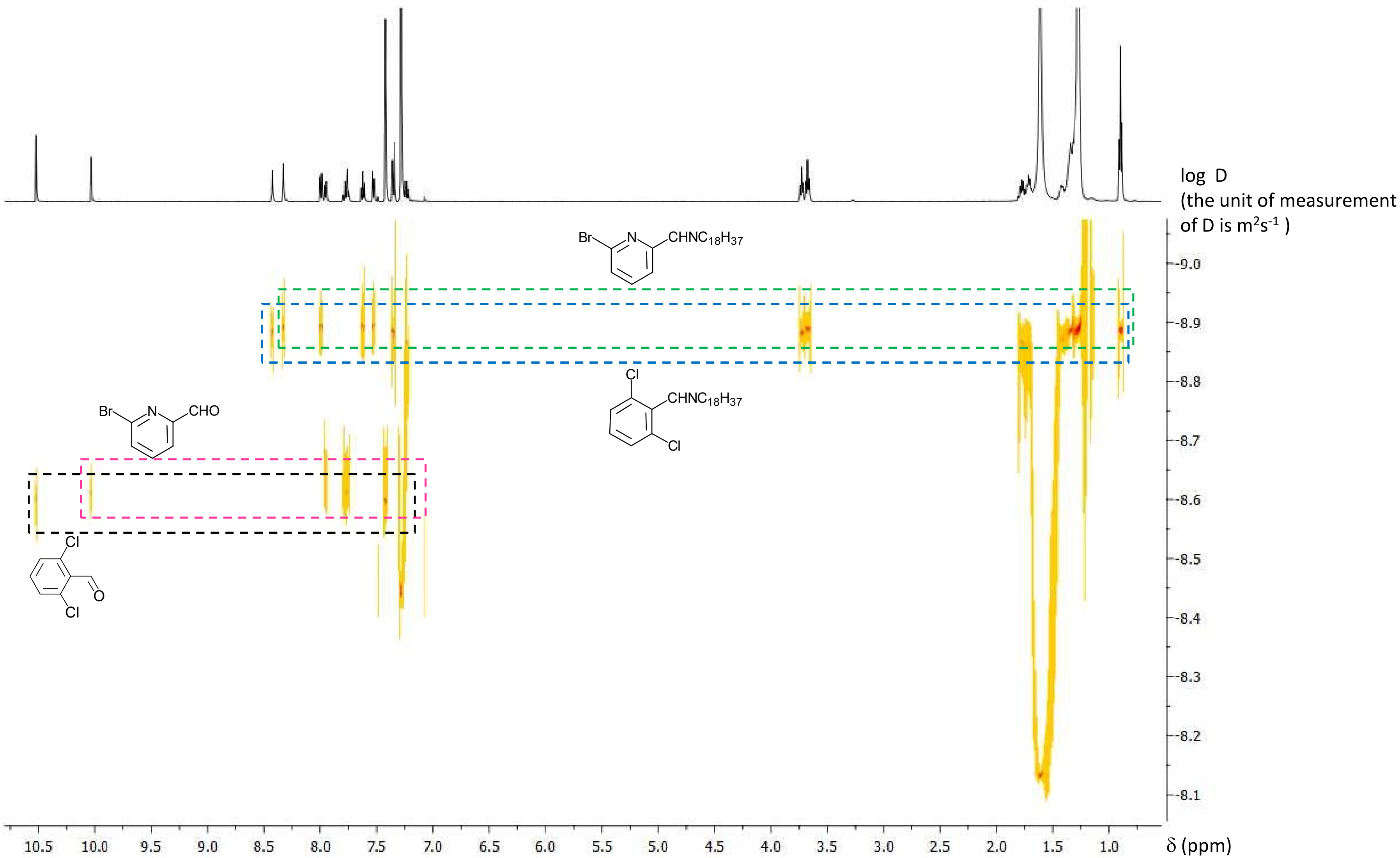

Solvent-free exchanges

- bis-imine from 2,6-pyridinedicarboxaldehyde and octadecylamine, with 4 equiv. of 2,4-dinitrobenzaldehyde
- bis-imine from isophthalaldehyde and octadecylamine with 1 equiv. of 2-phenyl-4,6-pyrimidinedicarboxaldehyde
- 2-phenyl-4,6-pyrimidinedicarboxaldehyde with 2 equiv. of imine from 4-butoxybenzaldehyde and octadecylamine

^1H DOSY NMR spectrum ($CDCl_3$, 500 MHz) of a sample of the solvent-free reaction mixture (19h, 65°C) of 1 equiv. of bis-imine from 2,6-pyridinedicarboxaldehyde and octadecylamine with an excess (4 equiv.) of 2,4-dinitrobenzaldehyde.

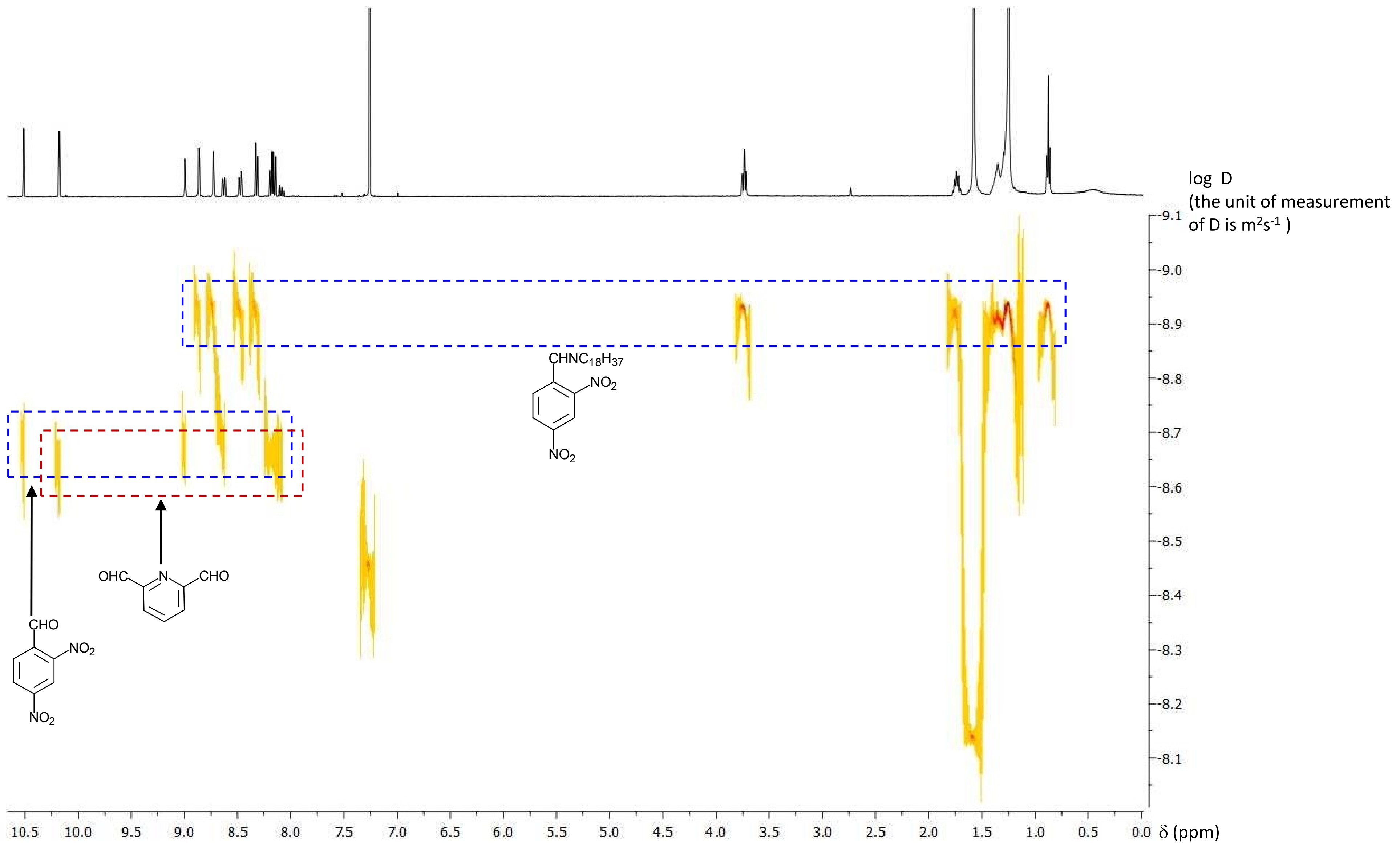

^{1}H DOSY NMR spectrum ($CDCl_3$, 500 MHz) of a sample of the solvent-free reaction mixture (16h, 70°C) of 1 equiv. of bis-imine from isophthalaldehyde and octadecylamine with 1 equiv. of 2-phenyl-4,6-pyrimidinedicarboxaldehyde.

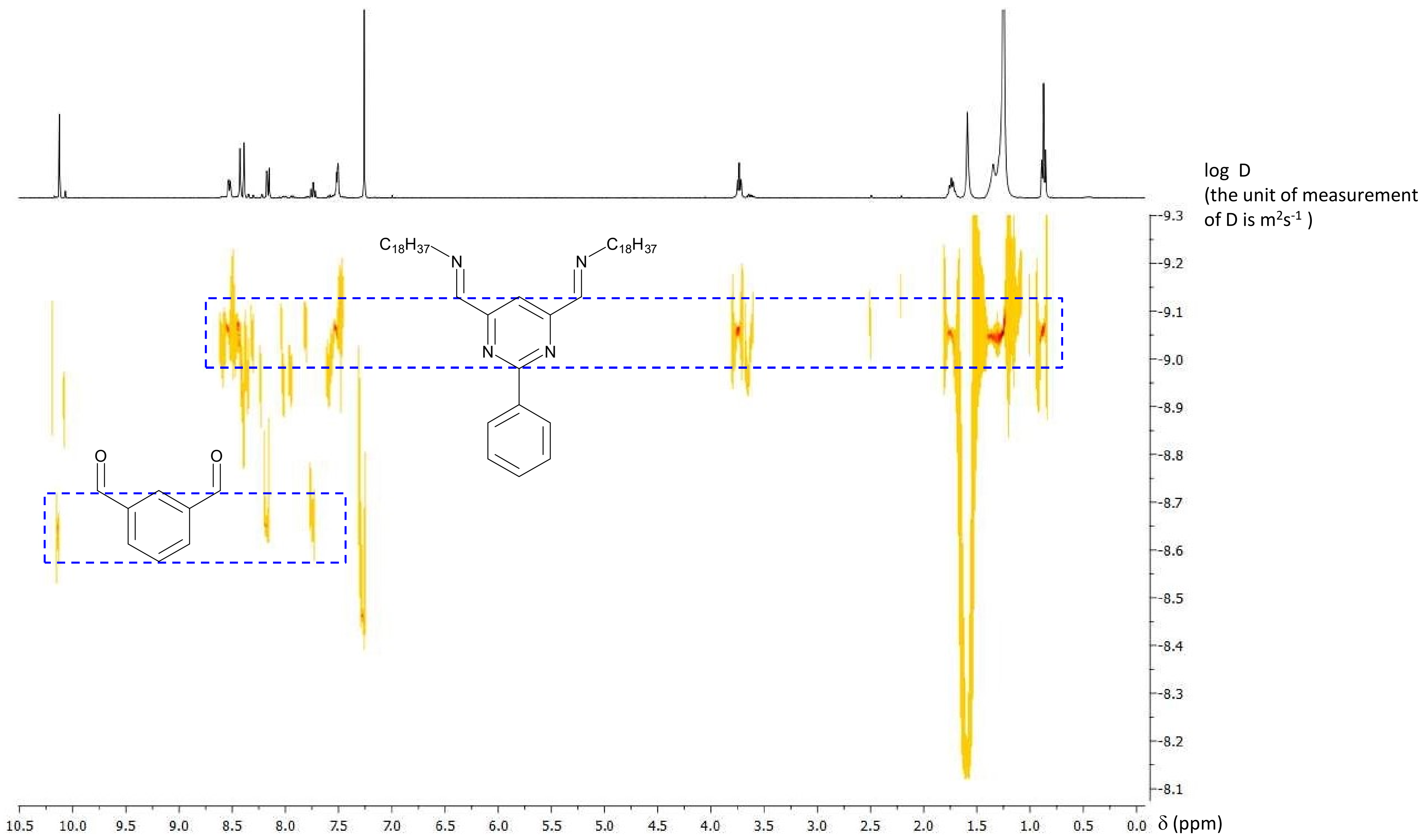

^1H DOSY NMR spectrum ($CDCl_3$, 500 MHz) of a sample of the solvent-free reaction mixture (18h, 70°C) of 2 equiv. of imine from 4-butoxybenzaldehyde and octadecylamine with 1 equiv. of 2-phenyl-4,6-pyrimidinedicarboxaldehyde.

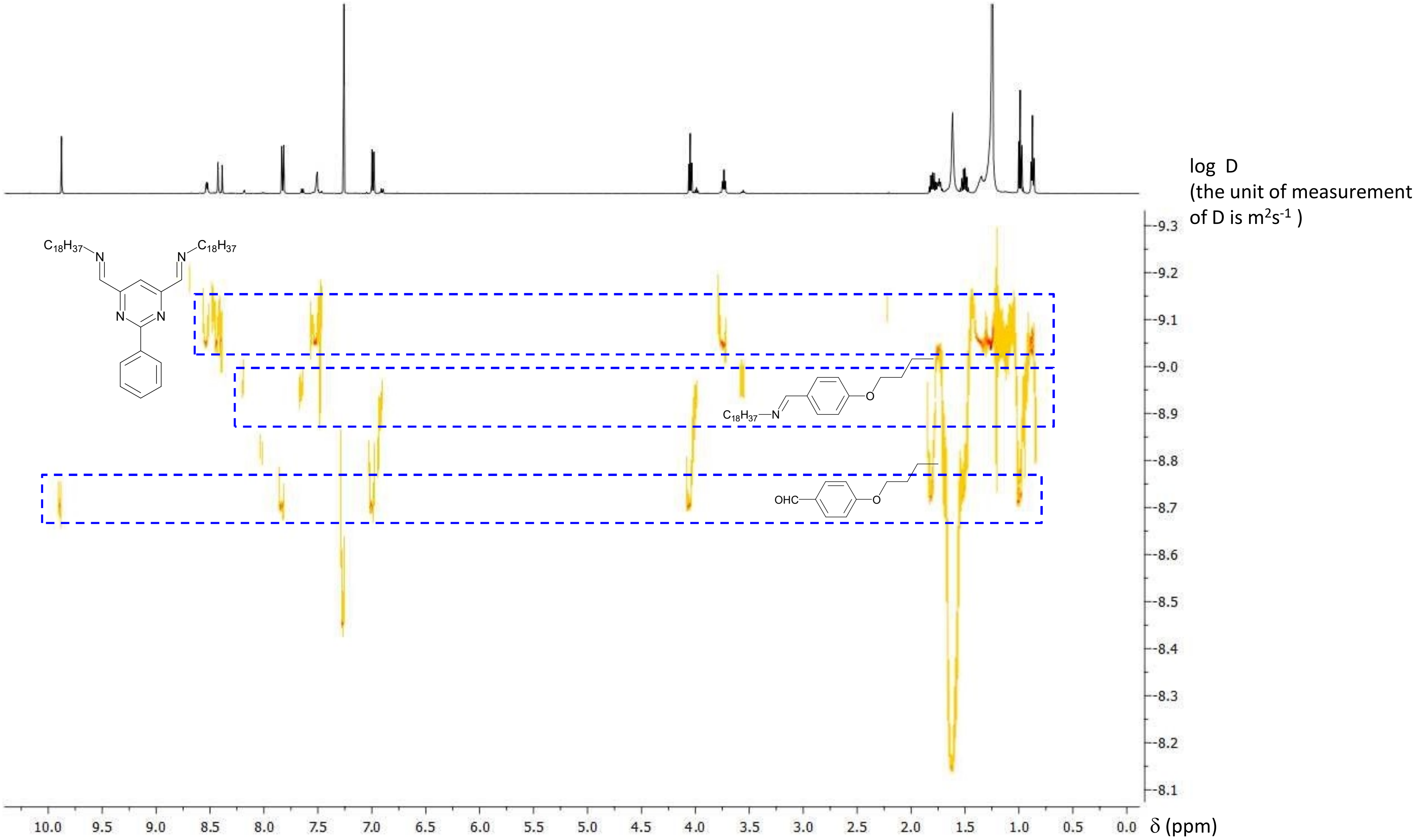

IR spectra (by ATR) for the solvent-free reaction of the bis-imine **dR4**$_2$ (from 2,6-pyridinedicarboxaldehyde **dialpy** and 4-(hexyloxy)aniline **R4**) (a) with 2 equiv. of octadecylamine **L5** (b), then with acid (c) and base (d), together with the spectra of starting materials (f-h) and of the bis-imine **dL5**$_2$ (from 2,6-pyridinedicarboxaldehyde and octadecylamine) (e)

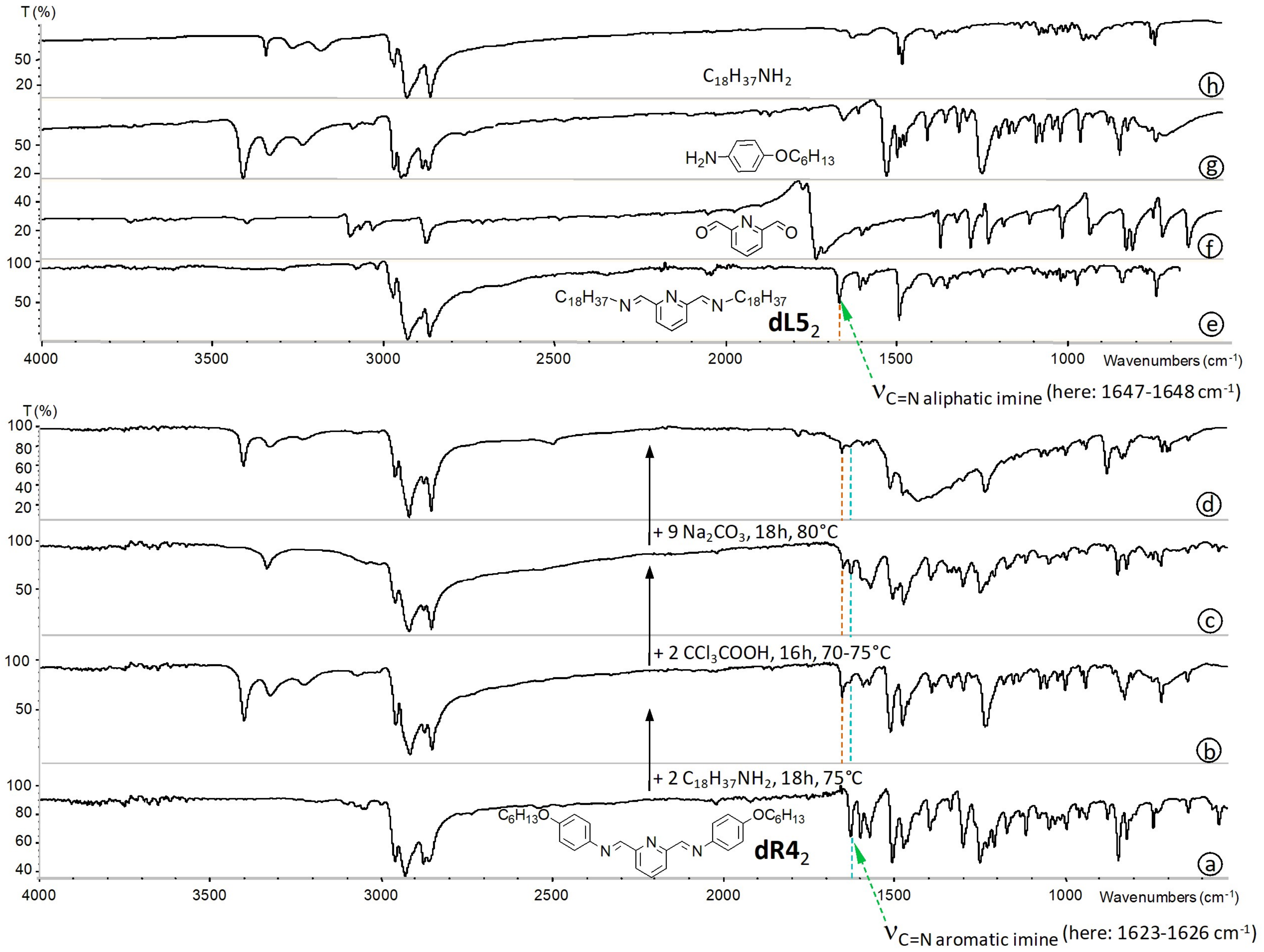

Competition between two monoaldehydes - a benzene-derived one and a pyridine-derived one - for *p*-toluidine

a)

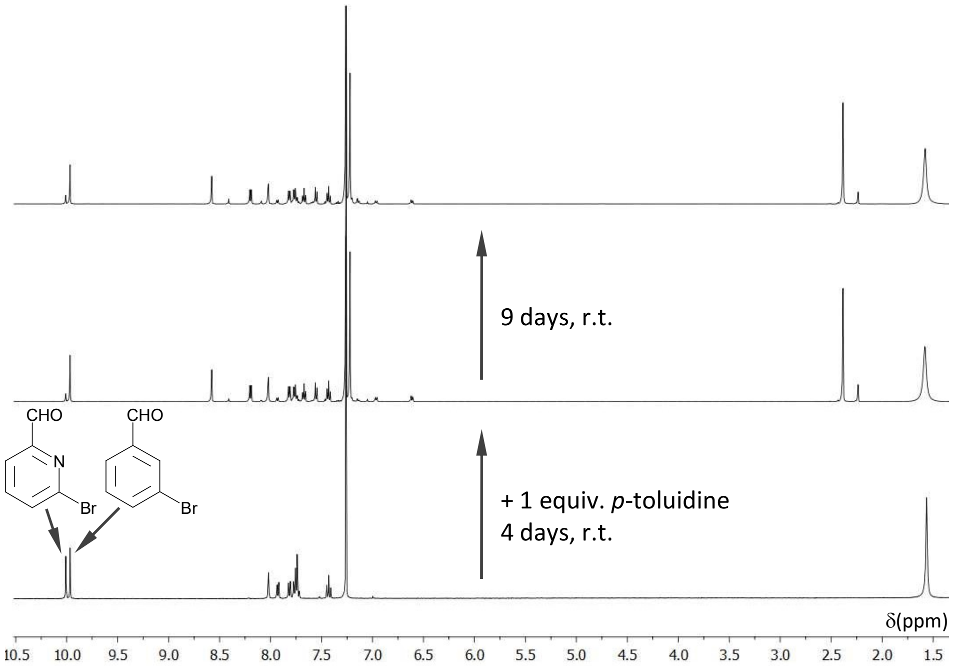

NMR-monitoring (400 MHz, $CDCl_3$) of the reaction of a mixture consisting of 1 equiv. of 3-bromobenzaldehyde and 1 equiv. of 6-bromo-2-pyridinecarboxaldehyde with 1 equiv. of *p*-toluidine.

b)

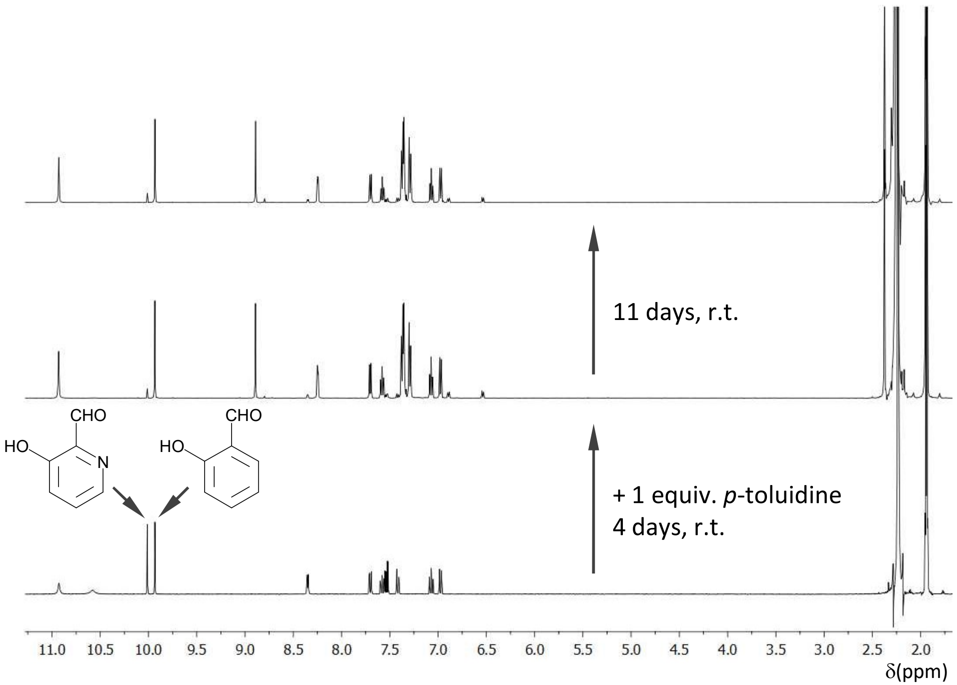

NMR-monitoring (400 MHz, CD_3CN) of the reaction of a mixture consisting of 1 equiv. of salicylaldehyde and 1 equiv. of 3-hydroxy-2-pyridinecarboxaldehyde with 1 equiv. of *p*-toluidine.

Acid/base-modulated transimination-switches between the aromatic bis-imine **AR2**$_2$ and the aliphatic bis-imine **AL2**$_2$ – ^{1}H NMR spectra (400 MHz, $CDCl_3$)

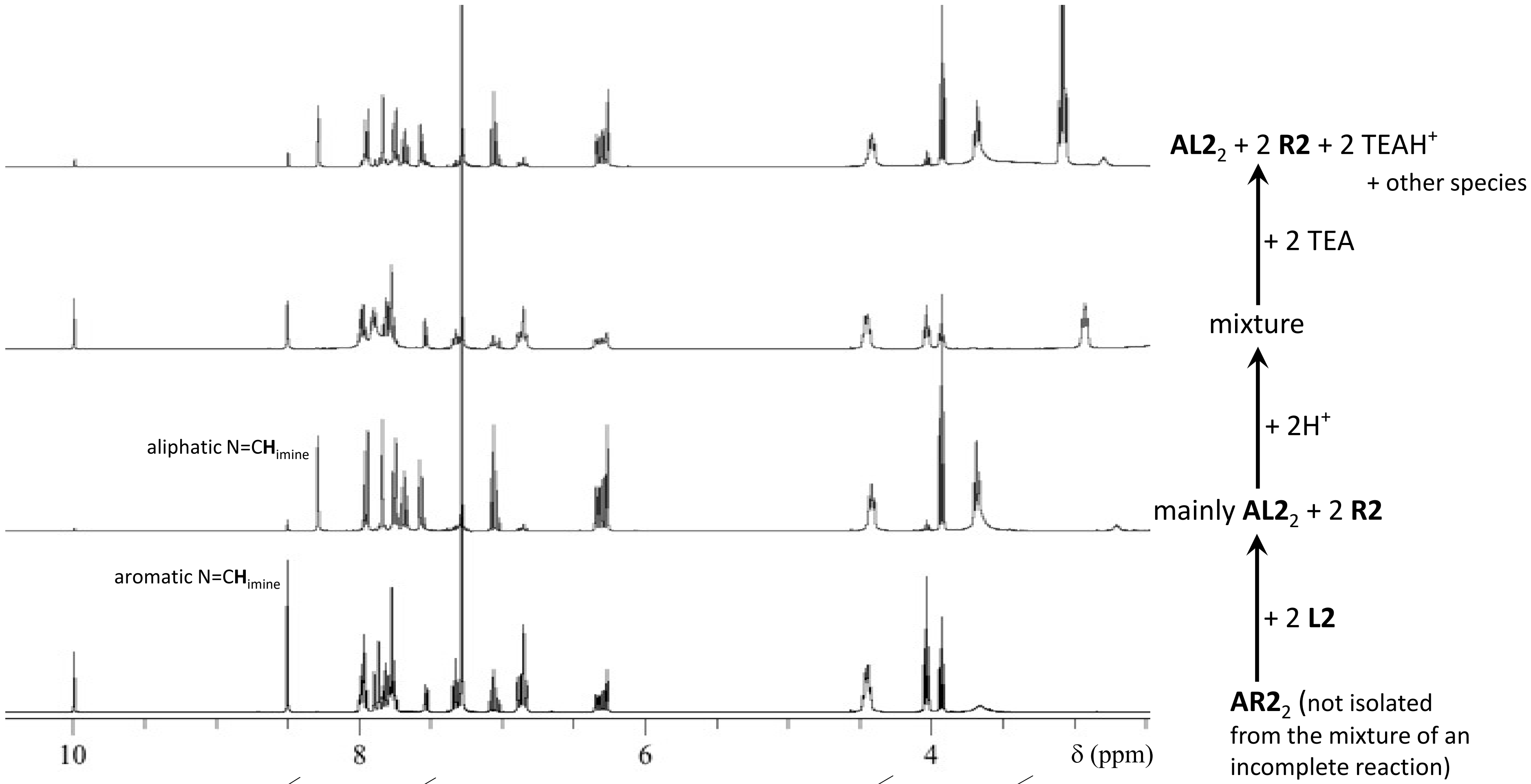

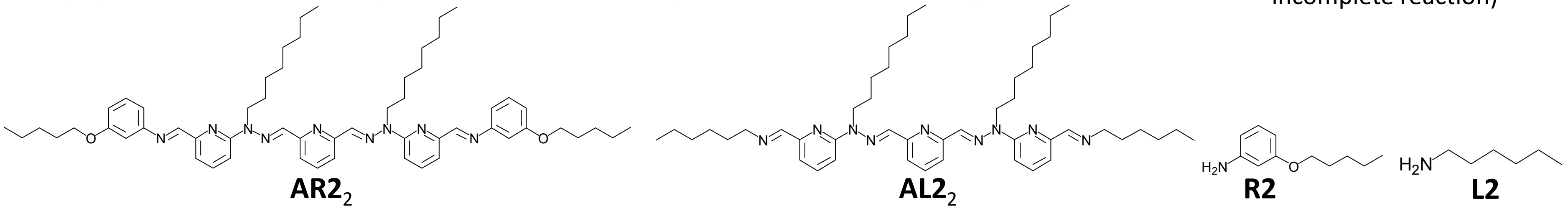

Supplement: Supplementary file 1 [file Supplementaryfile1.zip › Supplementary Material/synt-char.pdf]
